# Spectroscopically Deciphering the Formation and Reactivity of a High-Valent Ni(IV)Cl_2_ Species

**DOI:** 10.1021/jacsau.5c01182

**Published:** 2025-12-16

**Authors:** Ayushi Awasthi, Kiran Bhadauriya, Lucia Velasco, Raju Eerlapally, Asterios Charisiadis, Rakesh Kumar, Maxime Sauvan, Dooshaye Moonshiram, Sharath Chandra Mallojjala, Apparao Draksharapu

**Affiliations:** † Southern Laboratories-208A, Department of Chemistry, 30077 Indian Institute of Technology Kanpur, Kanpur 208016, India; ‡ Instituto de Ciencia de Materiales de Madrid Consejo Superior de Investigaciones Científicas, Sor Juana Inés de la Cruz, 3, Madrid 28049, Spain; § Department of Chemistry, 14787 Binghamton University, Binghamton, New York 13850, United States

**Keywords:** anionic ligands, high-valent nickel, oxygen atom transfer, pseudopeptide ligand

## Abstract

Recent investigations have demonstrated the appeal of using Ni­(II) complexes with redox-active ligands in fields like catalysis, electrochemistry, or materials sciences. Ni­(salen) complexes have particularly been shown to exhibit temperature-dependent equilibrium based on the localization of the unpaired spin. However, the usage of salen as a ligand has always restricted the characterization of a Ni­(IV) species with Ni bearing both the oxidizing equivalents. Hence, the current work aims to develop the biologically relevant pseudopeptide-based Ni complex that enables the formation and trapping of a high-valent Ni­(IV) species from its Ni­(II) precursor. The synthesized [LNi^II^] (**2**) (L = *N*,*N*’-(4,5-dimethyl-1,2-phenylene)­bis­(pyrrolidine-2-carboxamide)) was shown to form a high-valent [LNi^IV^Cl_2_] (**4Cl**) species, depending on the axial coordination, upon the addition of excess ceric ammonium nitrate, in the presence of chloride ions as an exogenous ligand, as supported by X-ray absorption spectroscopic analysis. Favorably, the formed Ni­(IV) species has also demonstrated electron transfer and oxygen atom transfer (OAT) reactions toward thioanisoles. Computational analysis of the mechanism revealed that the oxidation of thioanisoles proceeds via a stepwise pathway involving a single electron transfer from thioanisole, followed by OAT to the subsequent radical cation. The rate of these reactions demonstrated a strong dependence on the electronics of the substituents.

## Graphical abstract

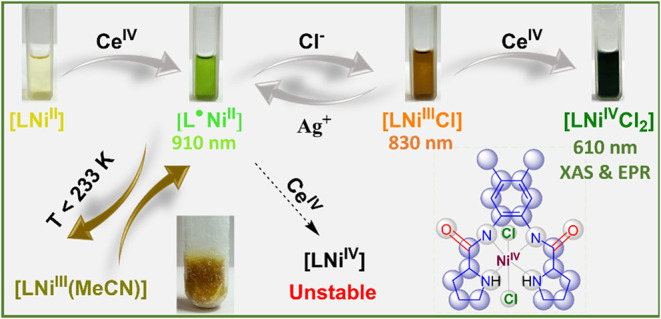

## Introduction

High-valent 3d metal species are critical in biological systems due to their unique ability to participate in electron transfer and catalytic processes.^1^ Their exceptional oxidizing capabilities originate from electron-deficient metal centers capable of multielectron redox reactions, allowing transformations inaccessible to lower-valent metals. Interestingly, the role of nickel in biological systems is more specialized and less widespread compared to iron and manganese, which readily stabilize in higher oxidation states such as Fe^IV/V^, and Mn^IV/V^.^2–4^ Therefore, the most prevalent nickel-dependent enzymes, such as hydrogenases and superoxide dismutase, rely on Ni^2+^ in their active sites.^5–9^ Despite the importance of high-valent Ni-based complexes in catalysis, their intrinsic instability and the challenges associated with isolating these high-valent states hinder a comprehensive understanding of their function within the catalytic cycle. Moreover, synthetically generated high-valent Ni species have been implicated as key intermediates in numerous cross-coupling, C–H activation, and oxidative coupling reactions.^10,11^ Recently, a Ni^IV^-σ-aryl group in the axial position supported by CNN pincer ligand scaffold has been claimed as the active intermediate to cause functionalization of the robust C–H bonds.^12^ A Ni^IV^ species has also been shown to be responsible for highly selective C_sp3_-O, C_sp3_-N, and C_sp3_-S coupling reactions with exogenous nucleophiles and C–H trifluoromethylation reactions.^13^

Only a few of the known groups, such as McDonald,^14–16^ Nam,^17^ Browne,^18^ Anderson,^19^ Mirica,^20,21^ Shanmugam,^22^ Ray, and Company^23,24^ have reported the existence of high valent Ni­(II)-O^•^ and Ni­(III)-O^•^ species, supported by dianionic and neutral ligands (Figure 1). In fact, Mirica and co-workers recently proposed the existence of a Ni­(IV) intermediate responsible for C–C and C–O coupled products.^20^ However, in biological systems, high-valent metal species are often stabilized by specific ligands or cofactors, ensuring that they remain reactive but not overly prone to decomposition. These metal centers are often coordinated with amino acid residues (such as cysteine, histidine, or glutamate) in the enzyme’s active site, which helps to stabilize the high oxidation states of nickel, ensuring that it can participate in catalytic cycles.

**Figure 1:**
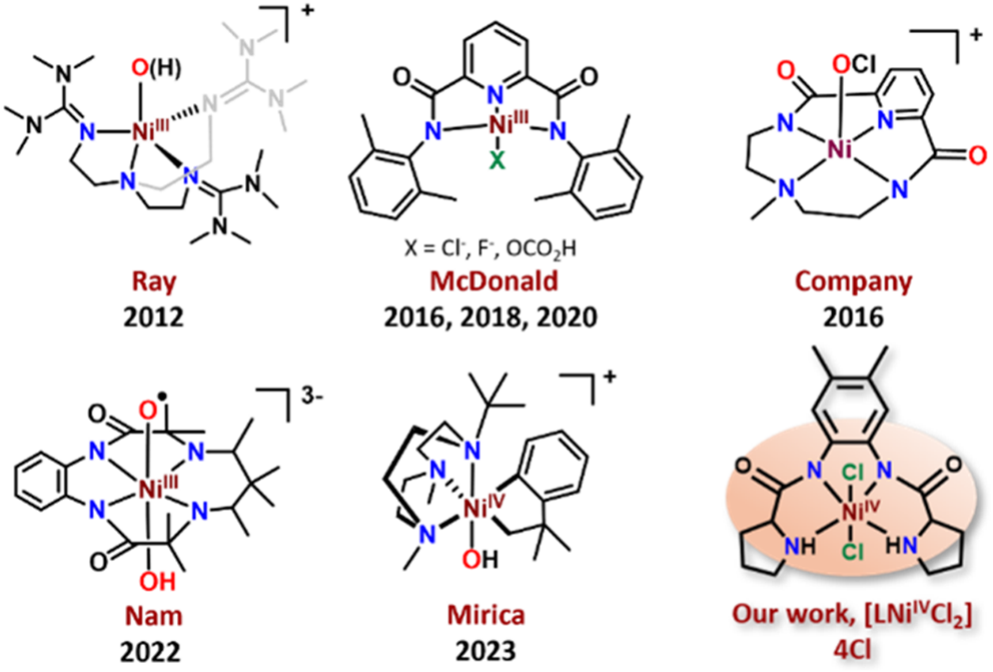
Structures of mononuclear nonheme high-valent Ni intermediates proposed in the literature and the intermediate characterized in the current work [LNi^IV^Cl_2_] (**4Cl**).

Very recently, we reported the action of excess *m*CPBA on Ni^II^(salen) (**1**), leading to the formation of a *formal* Ni­(V), Ni­(III) bisphenoxyl diradical species, Ni^III^(salen^••^), both in MeCN and CH_2_Cl_2_ at −40 °C.^25^ We also demonstrated that the localization of the unpaired spin in this three electron oxidized species can be modulated by adjusting the temperature and tuning the ligand field via different exogenous ligands.^26^ However, the production of a true high valent species that enables Ni to have all the oxidizing equivalents is crucially inhibited by the ligand’s two phenolate rings due to their oxidation susceptibility. Literature suggests that the introduction of amidate nitrogen in combination with the phenolate rings in the ligand framework lowers the M^III/II^ redox potential and can support the generation of high-valent species.^27^ Electrochemical studies of the Ni­(II) complex supported by tetradentate diamide-dipyrrolidine ligands have shown relatively low Ni­(III)/(II) reduction potentials, which indicated that the deprotonated amides were effective at stabilizing Ni in the +3 oxidation state.^28^

The current work aims to further explore the chemically oxidized species from a similar tetradentate diamide-dipyrrolidine ligand-based Ni­(II) complex [(Me_2_OPDPro)­Ni^II^] (**2**) (where Me_2_OPDPro = L = *N,N’*-(4,5-dimethyl-1,2-phenylene)­bis­(pyrrolidine-2-carboxamide)) upon the addition of ceric ammonium nitrate (CAN). We report for the first time the formation of two oxidized equivalent species, i.e., [LNi^IV^Cl_2_] (**4Cl**), from the reaction of **2** with excess CAN and in the presence of a halide source at 233 K in 1:16 MeOH:MeCN. The findings are supported by Density Functional Theory (DFT) calculations and multiple spectroscopies, including Extended X-ray Absorption Fine Structure (EXAFS), where **4Cl** adopts an octahedral geometry with two chlorido ligands (Scheme 1). The formed Ni­(IV) species was also found to be active toward electron transfer (ET) and oxygen atom transfer (OAT) reactions. This makes the proposed intermediate a potential oxidant, especially in the context of developing new therapeutic strategies, and creating novel catalysts for industrial and environmental applications.

**Scheme 1:**
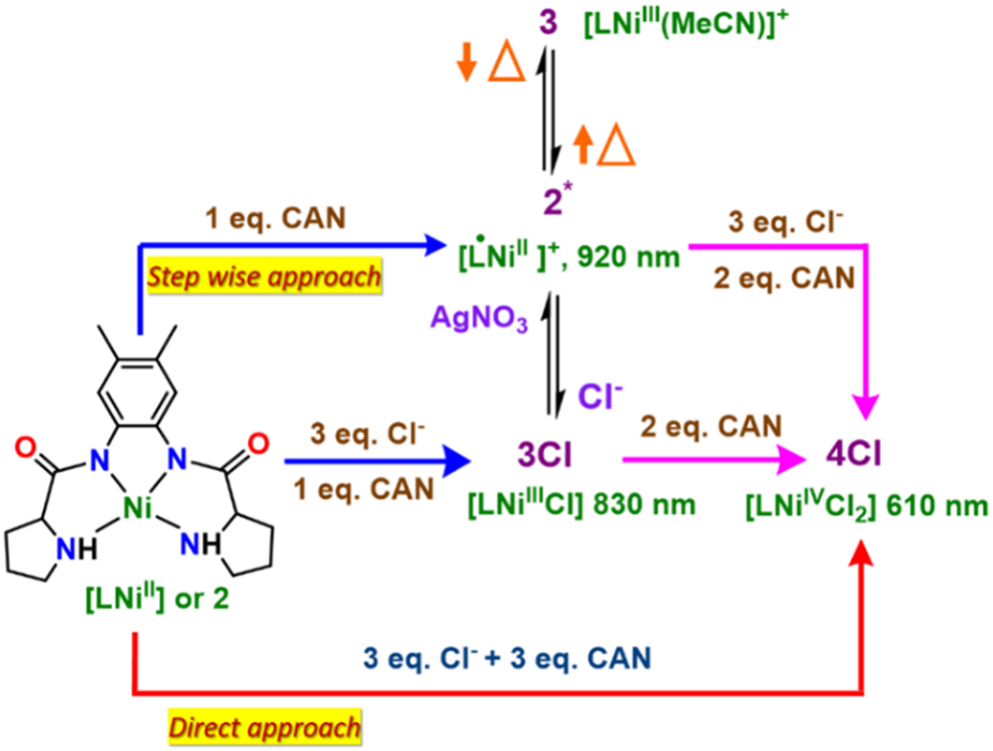
Schematic Diagram Illustrating (Stepwise Approach) the Formation of Ligand-Based One Oxidized Equivalent Species [L^•^Ni^II^]^+^ (2*), and Its Temperature-Dependent Equilibrium (Black Arrows) with Ni-Centered One Oxidized Equivalent Species [LNi^III^(NCMe)]^+^ (**3**)­^a^

a The same reaction in the presence of chloride produces [LNi^III^Cl] (**3Cl**) (blue arrow) directly. Formation of high valent [LNi^IV^Cl_2_] (**4Cl**) from one oxidized equivalent species (pink arrows) or (direct approach) directly from **2** (red arrow).

## Results and Discusson

Complex [(Me_2_OPDPro)­Ni^II^] (**2**) was synthesized using the previously described procedure for [Ni^II^(*S*,*S*-bprolben)]·2H_2_O (*S*,*S*-bprolben = *N*,*N* ^′^-bis­(S-prolyl)-1,2-benzenediamine) but with subtle modifications (Figures S1–S6).^28^ **2** crystallizes in an orthorhombic crystal system with the *‘P2*_1_2_1_2_1_ *’* space group. The yellowish orange crystals of **2** features a distorted square planar coordination environment around the central Ni­(II) ion, coordinated to two anionic nitrogens of the amide moieties and the two prolyl nitrogen atoms (Figure 2a and Tables S1–S3). The Ni–N_amine1_ and Ni–N_amine4_ bond lengths are 1.925(2) Å and 1.926(3) Å, respectively, whereas the Ni–N_amide2_ and Ni–N_amide3_ distances are 1.832(2) Å and 1.833(3) Å, which is 0.094 Å shorter than the Ni–N_amine_ ones (Table S2) due to their anionic coordination. One MeOH solvent molecule is also present in the unit cell as the solvent of crystallization. The crystal details matched the reported dianionic Ni­(II) complex well.^28^ The electrochemical investigation of **2** revealed three quasi-reversible redox waves appearing at E_1/2_^1^ = 0.69 V, E_1/2_^2^ = 1.04 V, and E_1/2_^3^ = 1.22 V vs Ag/AgCl at different scan rates in 1:16 MeOH:MeCN at 298 K (Figures 2b and S7 and Table S4). The anodic-to-cathodic peak current ratios (*i*_p,a_/*i*_p,c_)_1_ = 1.6, (*i*_p,a_/i_p,c_)_2_ = 5.3, and (*i*_p,a_/*i*_p,c_)_3_ = 2.9 observed for each wave in the cyclic voltammogram exhibit significant deviations from unity, confirming that these processes are electrochemically quasi-reversible.

**Figure 2:**
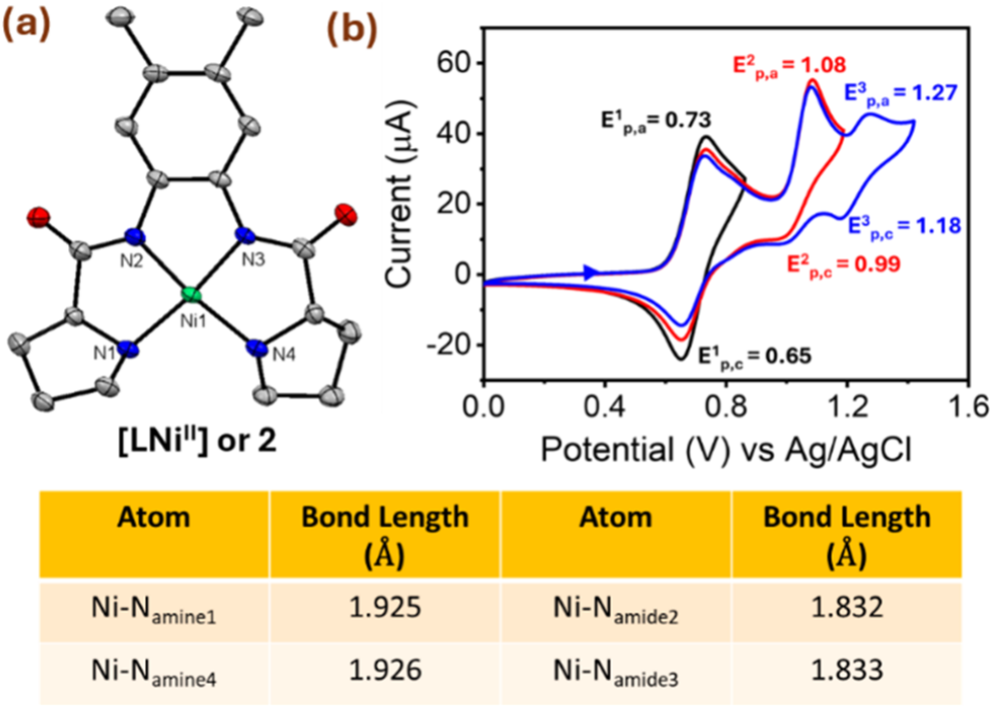
(a) X-ray crystal structure of **2** (Hydrogen atoms and CH_3_OH solvent molecule are removed for clarity) at 50% probability. The table shown below highlights the bond parameters. CCDC number 2419994. (b) Cyclic voltammograms of **2** stopped after each redox event at 100 mV s^–1^ scan rate in 1:16 MeOH:MeCN at 298 K. The arrow indicates the scan direction.

### Reaction of **2** with CAN

The UV/vis absorption spectrum of **2** displayed a d-d absorption at λ_max_ = 418 nm with ε_418 nm_ value of 160 M^–1^ cm^–1^ in 1:16 MeOH:MeCN at 298 K, typical for a Ni­(II) complex (Figure 3).^19^ By the addition of 1 equiv of CAN, a stable green species is produced having distinctive absorption bands at 800 nm (ε_800 nm_ = 4560 M^–1^ cm^–1^) and 920 nm (ε_920 nm_ = 3440 M^–1^ cm^–1^) in 1:16 MeOH:MeCN at 233 K (Figure 3b). The absorption in the NIR region is known to be a characteristic trait of the ligand oxidized radical, which we denoted as [L^•^Ni^II^]^+^ (2*).^29,30^ We also conducted spectroelectrochemical measurements on **2**, where the application of a potential of +0.78 V vs Ag/AgCl resulted in the appearance of an absorption band at 920 nm. This feature is indicative of the formation of a ligand-centered radical species, designated as 2*. These observations demonstrate that, at 298 K, ligand oxidation is favored under these conditions, underscoring the redox-active nature of the ligand framework in this system (Figures S8 and S9).

**Figure 3:**
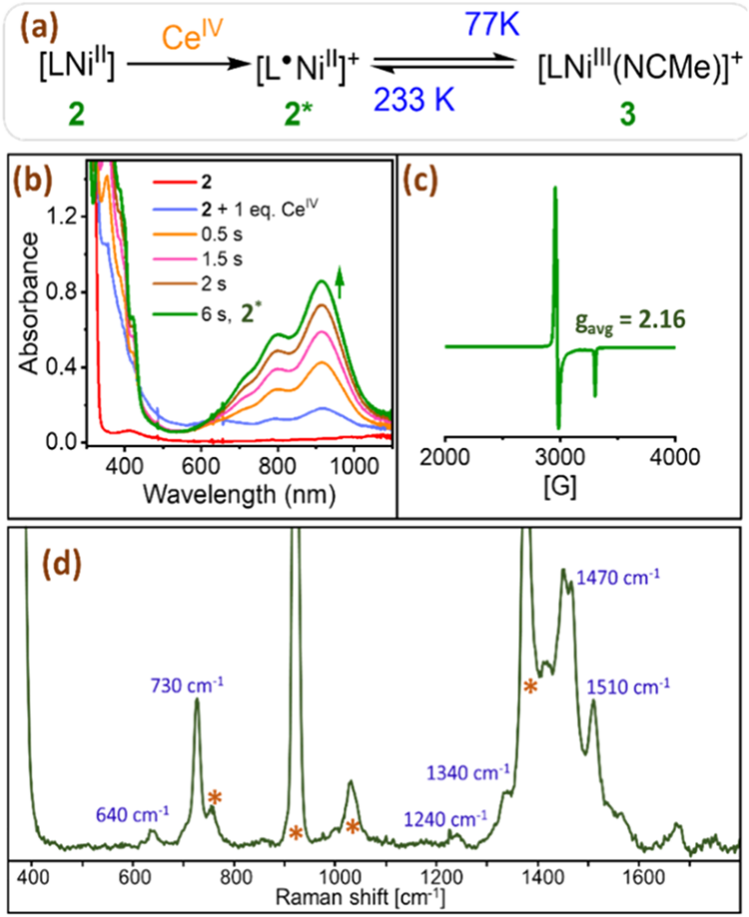
(a) A schematic representation illustrating the reaction of **2** with CAN at 233 K, leading to the formation of 2*, along with its temperature-dependent equilibrium with **3**. (b) UV/vis absorption spectral changes observed upon the addition of 1 equiv of CAN to 0.25 mM **2** in 1:16 MeOH:MeCN at 233 K to form 2*. (c) X-band (9.45 GHz) EPR spectrum of **3** measured at 77 K. *Conditions to generate* **3**: 2 mM **2** in 1:16 MeOH:MeCN + 1 equiv of CAN (in MeCN) at 77 K. Modulation amplitude 1.98 G; modulation frequency 100 kHz, and attenuation 20 dB. (d) Resonance Raman spectrum of 2* in 1:16 MeOH:MeCN at 233 K using λ_exc_ = 405 nm. *Indicate peaks from the solvent. *Conditions to generate* 2* *:* 0.5 mM **2** in 1:16 MeOH:MeCN + 1 equiv of CAN (in MeCN) at 233 K.

To validate our hypothesis, we subjected the species to EPR spectroscopy at 77 K. An axial signal with a g_avg_ of 2.16 indicates the presence of an S = 1/2 spin in the system (Figure 3c).^31^ The resonance Raman (rRaman) analysis performed at 233 K for the same showed a majority of the signals from 1200 to 1700 cm^–1^ region (Figure 3d) attributed to the radical localized on the ligand for 2* similar to the reported Ni^II^(salen^•^).^25,26^ The literature suggests that radical formation is usually accompanied by an isotropic signal in its EPR spectrum.^25,26^ An axial signal in the present case represents the sign of temperature-dependent equilibrium between the metal and ligand-centered one oxidized equivalent species (Scheme 1 and Figure 3a). Moving to 77 K, as known, favors the Ni-based oxidation, i.e., formation of [LNi^III^(MeCN)] (**3**) species.^26^ Given that the species **3** is accessible only at low temperatures (below 77 K) and does not persist in solution at ambient conditions, its characterization was carried out via XAS spectroscopy (*vide infra*). Notably, the green color of 2* at 233 K in solution changes to orange upon freezing at 77 K, providing visual confirmation that the two species exist in a temperature-dependent equilibrium between ligand- and metal-centered oxidized forms (Figure S10d). At temperature of 77 K, the sample converts to a metal-localized species, [LNi^III^(MeCN)], **3** that exhibits an axial signal. The observation that treatment with 1 equiv of magic blue at 233 K also generates 2* effectively excludes the possibility of NO_3_^–^ binding in the case of CAN (Figure S10).

### Effect of Halide Ion

The formation of one oxidized equivalent species, 2*, by the addition of 1 equiv of CAN thus demonstrates that the first wave occurring at 0.69 V vs Ag/AgCl in its cyclic voltammogram corresponds to (L^•^)­Ni^II^/(L)­Ni^II^ (Figure S7). Thereby, in the absence of any extra site of oxidation within the ligand framework, Ni could be assumed to be the epicenter of any further oxidation event happening in **2**.

To investigate this further, when more equivalents of CAN were added to 2*, a decay was observed of the 920 nm band to 990 and 780 nm with the simultaneous generation of a 610 nm absorption band that decays within 10–15 s at 233 K (Figure S11 and S12). Attempts to stabilize the 610 nm absorbing species by increasing the equivalents of CAN or changing the solvent were unsuccessful. This observation indicated the need for an anionic ligand, as such scaffolds are important cofactors in stabilizing high-valent metal species. Hence, a Cl^–^ source (i.e., 3 equiv of tetrabutylammonium chloride (TBACl)) was added to the formed one oxidized equivalent species, 2*, leading to an instant shift from the absorbance at 920 to 830 nm (ε_830 nm_ = 1520 M^–1^ cm^–1^) along with an appearance of a small absorption feature at 570 nm (ε_570 nm_ = 620 M^–1^ cm^–1^) (Figure S13a). The fact that the absorption feature changes in the presence of Cl^–^ indicates its coordination with the Ni center. We denoted the newly formed 830 nm absorbing species as **3Cl**. To verify this, **3Cl** was treated with 5 equiv of AgNO_3,_ where the regeneration of 2* was observed. Further addition of Cl^–^ and AgNO_3_ in a respective manner formed **3Cl** and 2* with the surfacing of a white precipitate of AgCl (Scheme 1 and Figure S14). In addition, treatment of **2** with 4 equiv of Br^–^ and 1 equiv of CAN resulted in the emergence of an absorption band at 850 nm, attributed to the formation of **3Br**. Notably, this band exhibits a 20 nm red shift compared to that of **3Cl** (Figure S15). It was apparent from the literature that Ni^II^(salen^•^) species undergo conversion to Ni^III^(salen) in the presence of exogenous coligands.^25,26^ Similarly, the addition of Cl^–^ to 2* promotes a shift in the oxidation locus to the Ni center, leading to the formation of [LNi^III^Cl] (**3Cl**) in the present study. The EPR spectrum at 77 K shows an axial signal with a g_avg_ value of 2.16 for one oxidizing equivalent Ni^III^ complex (Figure S13b). We additionally recorded excitation wavelength-dependent Raman spectra of **3Cl**, which showed no resonance enhancement in the region associated with ligand oxidation, in CH_3_CN and CD_3_CN, further supporting the formation of a Ni-based oxidized species (Figure S13c and S13d).

Having proven the binding of Cl^–^ to the one oxidized equivalent complex, **3Cl**, more equivalents of CAN were added to access the high valent oxidation state. As expected, that brought enough stability to the transient 610 nm absorbing species, **4Cl** (Figures 4a and S16). Favorably, the same was observed by the subsequent addition of TBACl and excess CAN to **2** (Scheme 1, red arrow, and Figure S17). The optimized condition suggests that introduction of 3 equiv of CAN to the mixture of **2** enriched with 3 equiv of TBACl produces the maximum yield of **4Cl** (ε_610 nm_ = > 6500 M^–1^ cm^–1^), which is stable for more than 30 min (Figures S17 and 18) at 233 K. Addition of 5 equiv of AgNO_3_ to the **4Cl** species causes a decrease in its absorbance signifying the binding of Cl^–^ and proves the stability in **4Cl** (Figure S19). The presence of AgNO_3_ in the mixture takes away the Cl^–^ ligand to give AgCl precipitate, leaving only NO_3_^–^ ions in the mixture that offers markedly lower stabilization of high-valent species as compared to chloride *(Note: In the absence of any Cl* ^*–*^ *source, the addition of excess CAN makes the species extremely unstable)* (Figure S11). Unlike **3Cl**, **4Cl** decays to a low-valent species in the presence of AgNO_3_ and is not regenerated upon the addition of TBACl (Figure S19). The influence of Cl^–^ and NO_3_^–^ as axial ligands on the redox behavior of **2** was also examined (Figures S20 and S21). As shown in Figure S21 and Table S4, the presence of Cl^–^ caused a significant shift in the redox potential of **2** by approximately 250 mV. This observation highlights the strong effect of Cl^–^ coordination on the redox processes, facilitating access to the high-valent species at lower applied potentials. However, in the presence of nitrate ions, no significant shift in the potential of **2** was noticed.

**Figure 4:**
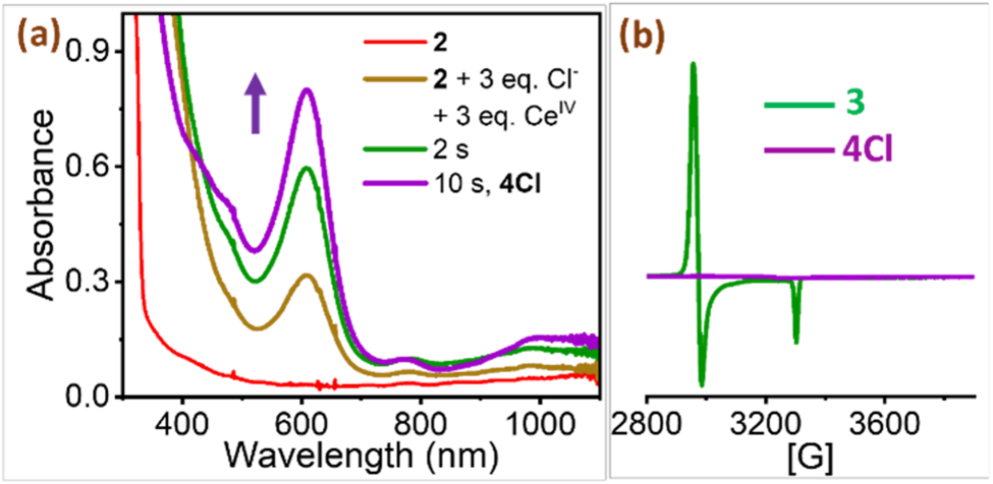
a) UV/vis spectral changes observed upon addition of 3 equiv of CAN to a mixture of 0.125 mM **2** + 3 equiv of TBACl, in 1:16 MeOH:MeCN at 233 K. b) X-band (9.45 GHz) EPR spectrum of **4Cl** measured at 77 K that showed no signal. The EPR spectrum of **3** is shown for comparison purposes. Modulation amplitude 1.98 G; modulation frequency 100 kHz, and attenuation 20 dB. *Conditions to generate**4Cl**:* 2 mM **2** in 1:16 MeOH:MeCN + 3 equiv of TBACl (in MeCN) + 3 equiv of CAN (in MeCN) at 233 K.

To further establish the binding of halides, Br^–^ was employed prior to the addition of excess CAN. Interestingly, a 30 nm red shift was noticed in the absorption spectrum, accompanied by a unique species, **4Br**, which absorbs at 640 nm (Figures S22 and S23). This ascertains the formation of a Br^–^ bound high valent species in **4Br**, which regains the absorption corresponding to **4Cl** when enriched again with the Cl^–^ source (Figure S23). The reverse effect occurs upon the addition of Br^–^ to the formed **4Cl**, which contrarily causes its decay and does not produce **4Br** (Figure S24). This behavior of different halides could be inferred based on their binding tendency, where Cl^–^ being the strongest ligand, is not easily replaced by Br^–^.

To investigate the stability of the ligand under oxidative conditions, a control experiment was conducted by introducing CAN to a solution containing the decayed **4Cl** species. The reappearance of the characteristic absorption band at 610 nm in the UV/vis spectrum upon CAN addition signifies that **4Cl** can be regenerated, with an estimated recovery yield of ∼45%. These findings indicate that the ligand framework is preserved during the oxidation process and does not undergo irreversible degradation under the experimental conditions (Figure S12).

Since **4Cl** can be a high valent species, it was tested for its electron transfer reactivity toward ferrocenes. The quantity of CAN that is added to produce these high-valent Ni species prevents the tracking of the generated ferrocenium concentration, which ultimately limits the computation of the precise oxidizing equivalents that are included in them. Nevertheless, the addition of 5 equiv of ferrocene (Fc) to **4Cl** immediately reacted and caused an instant decay in its absorbance (Figure S25a and S25d). However, it was found that **4Cl** was being reduced to **3Cl** when weaker reducing agents like acetylferrocene (AcFc) (Figure S25b and S25e) and diacetylferrocene (Ac_2_Fc) (Figure S25c and S25f) were used, validating the presence of more than one oxidizing equivalents in **4Cl**. To further unveil the identity of **4Cl**, we resorted to EPR spectroscopy at 77 K. The EPR showed a silent signal for an S = 0 spin, which indicated an intermediate bearing of two oxidizing equivalents (Figure 4b). In addition to UV/vis and EPR, the resonance Raman spectroscopy was conducted at 233 K using a 638 nm excitation wavelength. Unlike 2*, the resonance Raman spectrum of **4Cl** exhibited only a few bands at 400–600, 1000, and 1300 cm^–1^ (Figure S26). The reduced number of bands observed for **4Cl**, compared to **3Cl**, suggests that its formation involves metal-centered oxidation rather than ligand-centered oxidation. The resonance Raman spectra of **4Cl** recorded at 77 and 233 K were essentially indistinguishable (Figure S27), demonstrating that the complex maintains its structural integrity over this temperature range. The observed resonant enhancement of ligand-based vibrational bands further supports that the electronic transition at 610 nm arises from ligand-to-metal charge transfer (LMCT) (Figure S27). No shift was observed in the resonance Raman spectrum of **4Br** compared to **4Cl** (Figure S28), suggesting the charge transfer from the ligand (L) to nickel.

The observation of fewer bands indicates a metal-centered oxidation event during the reaction of CAN with **3Cl**. Low-temperature NMR spectroscopy of the **4Cl** complex revealed sharp signals in the diamagnetic region, consistent with an S = 0 ground state and supporting the assignment of a Ni­(IV) oxidation state. In contrast, 2* exhibited broadened NMR resonances typical of paramagnetic species, indicative of *formal* Ni­(III) character (Figure S29). These observations are in agreement with the results obtained from EPR spectroscopy.

To better understand the electronic structure of these species, we turned toward X-ray absorption near-edge structure (XANES) and EXAFS. Both spectroscopic analyses were carried out on complexes **2**, **3**, and **4Cl** at the Ni K-edge energy to gain comparative insights into their coordination behaviors and structural conformations (Figures 5 and S30–32). The complexes were kept at 15 K in a He atmosphere at ambient pressure and recorded as fluorescence excitation spectra.

**Figure 5:**
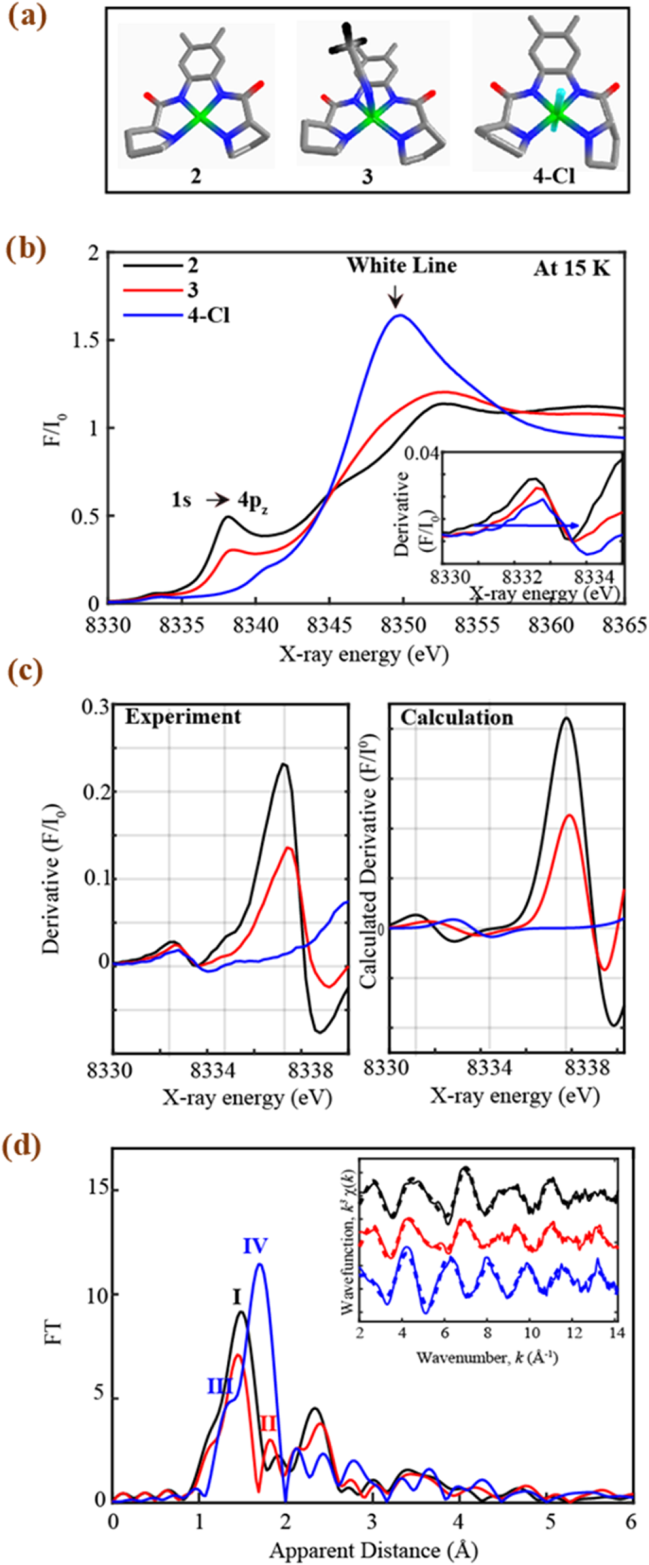
a) Structures of the Ni-based complexes **2** (black), **3** (red), and **4Cl** (blue) measured through XANES and EXAFS at 15 K. b) Experimental normalized Ni K-edge XANES of **2** (black), **3** (red), and **4Cl** (blue). Inset. Zoom-in of the derivative of the pre-edge regions. c). Left. Derivative of the experimental pre-edge and rising edge regions. Right. TD-DFT simulated XANES derivative spectra corresponding to the formation of a square planar **2** (in black), square pyramidal **3** (in red), and an octahedral **4Cl** (in blue) d). Fourier transforms of k^3^-weighted Ni EXAFS of **2**, **3**, and **4Cl**. Inset. *k* ^3^[χ­(*k*)]-weighted traces as a function of *k*, the photoelectron wavevector (solid lines), and fitted (dashed lines) of **2**, **3**, and **4Cl**. Experimental spectra were calculated for *k* values of 2–14.15 Å^–1^.

**2** displays an intense main peak at 8338.06 eV along the rising edge from 8335 to 8350 eV, assigned as a 1s → 4p_*z*_ transition^32,33^ as previously observed in square planar Ni­(II) complexes.^34^ Both **3** and **4Cl** additionally display a clear edge energy shift of 0.67 eV from 8343.56 to 8344.23 eV compared to **2** at normalized 0.5 absorption, reflecting the higher ionization energy required for ejecting a core 1s electron from more positively charged ions (Figure 5b),^34^ which confirms the metal-based oxidation behavior detected at 15 K. On the one hand, a small shift in the pre-edge region of 0.97 eV from 8332.90 to 8333.10 is observed upon oxidation of **2** to **3**. On the other hand, a shift in the pre-edge of 0.12 eV from 8333.10 to 8333.22 eV is obtained upon oxidation of **3** to **4Cl** (Figure 5b inset, Figure S30, Table S5). The presence of pre-edge features corresponds to 1s to 3d quadrupole transitions and dipole excitations of the core electrons into the valence 3d states hybridized with ligand p orbitals.^29,30^ Such small shifts in the pre-edge peak transitions, ranging within less than 0.5 eV for Ni-based complexes upon oxidation, have been consistently demonstrated by Sarangi,^34^ Solomon,^35^ and co-workers.

It is also important to remark that **4Cl**, unlike **2** and **3**, has a higher metal–ligand covalency with two bound chlorido ligands (*vide infra*). The higher coordination of **4Cl** with six bound atoms (Figure 5a) leads to a greater charge neutralization of the Ni atom,^34^ thereby leading to a decrease in its effective nuclear charge. This also accounts for a small shift in energy in the pre-edge and rising edge transitions of **4Cl** compared to **2** and **3** (Figure 5b, inset). The intensity of the rising edge spectrum, together with a more smeared or lack of the 1s → 4p_*z*_, has also been known to correlate proportionally to its covalency.^32^ In this case, a more intense white line (Figure 5b) in **4Cl** at 8349 eV versus **3** and **2** at 8352.37 eV confirms the higher coordination environment of **4Cl** in comparison to **3** and **2**. Furthermore, an octahedral geometry in **4** with two chlorido ligands results in a degeneracy among the 4p orbitals, with the 1s → 4p_*z*_ transition becoming smeared and almost indistinguishable from the white line transition.^32^

**3** by contrast shows a weaker and slightly shifted 1s → 4p_*z*_ transition at 8338.33 eV vs **2** at 8338.02 eV. The more intense white line of **3** and its weaker 1s → 4p_*z*_ transition suggests that **3** maintains a penta-coordinated geometry, probably with a bound acetonitrile molecule upon oxidation (Figure 5a). A distorted square planar geometry in **3** would have resulted in a similarly intense 1s → 4p_*z*_ transition and white line feature as **2**, as previously demonstrated.^32,36^ In contrast, an octahedral geometry in **3** would have led to a smeared 1s → 4p_*z*_ transition as that observed in **4Cl**. The formations of square-planar coordination in **2**, square pyramidal geometry in **3** with a loosely bound solvent acetonitrile, and octahedral geometry in **4Cl** with two chlorido ligands were further supported through time-dependent Density Functional theoretical (TD-DFT XANES) simulations, which showed good agreement in the pre-edge region with the experimental derivative XANES features (Figures 5c, S31, and Table S5).

The EXAFS spectra of the three Ni complexes are shown in Figure 5d. A prominent peak **I** observed in the EXAFS spectrum of **2** corresponds to the averaged contribution of the Ni–N bond distances, respectively (black trace, Figure 5d). By contrast, two peaks, **I** and **II**, representing the Ni–N and Ni-solvent contribution, can be seen in **3**′s EXAFS spectrum (red trace, Figure 5d). **4Cl**, compared to **2** and **3**, shows a weaker peak **III** corresponding to the averaged contributions of the shortened Ni–N distances (Figure 5d, blue trace) and a prominent peak **IV** corresponding to elongated Ni–Cl bond distances, respectively (blue trace). The higher amplitude of peak **IV** (Figure 5d) in **4Cl** is due to the presence of its two distinct Ni–Cl bond distances, which bear larger atomic weight versus light nitrogen atom scatterers as previously illustrated in Co tetramacrocyclic ligands with axial chlorido ligands.^37,38^

The EXAFS fits for the extraction of actual bond distances for all three complexes, which are shown in Figure 5d inset, Figure S32, and Table S6. Analysis of the EXAFS spectrum of **2** resolves 4 averaged Ni–N distances at 1.87 Å (Table S7, fit 2, Figure S32a) within 0.01 and-0.04 Å of the averaged Ni–N distances of 1.88 and 1.91 Å derived from XRD analysis and DFT optimizations respectively (Table S8) whereas EXAFS fits of **3** illustrates 4 Ni–N bond distances at 1.86 Å and an elongated Ni-NCCH_3_ solvent distance at 2.54 Å (Table S7, Fit 5, Figure S32b). Similarly, EXAFS analysis of **4Cl** reveals 4 Ni–N distances at 1.87 Å and 2 Ni–Cl bond distances at 2.15 Å (Table S7, fit 7, Figure S32c). The average theoretically determined Ni–N and Ni-NCCH_3_ solvent distance for **3** are 1.90 and 2.39 Å, whereas **4Cl** shows 4 Ni–N distances at 1.93 Å and 2 Ni–Cl bond distances at 2.35 Å (Table S8), respectively, within 0.06 and 0.20 Å from the experimentally determined distances from EXAFS data analysis (Table S7). The experimentally fitted Ni–N distances for **4Cl** are not significantly shortened compared to those of **3** due to the presence of its two chlorido ligands and distorted octahedral geometry. It is also important to note that metal-chlorido complexes have been reported to exhibit metal-Cl bond distances ranging from 2.19 to 2.41 Å,^39^ which closely aligns with the values obtained from both EXAFS and DFT calculations in our study. The observed 0.20 Å deviation in the Ni–Cl bond distance between the DFT and EXAFS results can be attributed to the known tendency of DFT methods in overestimating metal–ligand bond lengths, particularly in labile metal-chlorido interactions. Furthermore, the **4Cl** species analyzed via EXAFS was rapidly frozen and measured within 30 s of formation in a helium cryostat, as detailed in the Experimental section. This likely inhibited further elongation and the dynamic lability typically observed in Ni–Cl bonds.

Combined theoretical methods and XAS analysis thus suggest a square pyramidal geometry for **3**, featuring a loosely bound CH_3_CN ligand coordinated to Ni­(III) [LNi^III^(NCMe)] whereas, intermediate **4Cl** adopts an octahedral geometry with two chlorido ligands [LNi^IV^Cl_2_] (Table S8). A XANES and EXAFS comparison of **3** and **3Cl** was further conducted (Figure S33). Although **3** and **3Cl** bear a square bipyramidal geometry with a respective coordinated solvent and chloride bond, **3Cl** displays a slightly more intense more pre-edge feature at 8330.10 eV due to its more distorted structure and noncentrosymmetric geometrical configuration compared to **3** (Figure S33b). Indeed noncentrosymmetric complexes have been shown to have an increased intensity in their pre-edge features due to an increase in the metal 4p mixing into the 3d orbitals contributing toward the electric dipole 1s to 4p character of this transition EXAFS Fits of **3Cl** further reveal 4 Ni–N bond distances at 1.86 Å and 1 Ni–Cl distance at 2.54 Å (Fit 10, Table S7, Figure S33b), similar to that of **3** (Fit 5, Table S7). It is important to note that EXAFS shows an improvement in the overall fit quality of **3Cl** upon the inclusion of two chloride interactions compared to one (Fits 11 vs 10, Table S6). Moreover, unlike in **4Cl**, where the Ni–Cl interaction constitutes a true bond, the Ni–Cl contact in **3Cl** is a weak interaction at 2.54 Å. As a result, the corresponding backscattering amplitude is significantly weaker and comparable to that of the Ni-solvent interaction observed in **3** (Figure S33c and Table S8).

### Density Functional Theoretical Studies

Furthermore, TD-DFT computations were performed on the lowest-lying complexes to identify the predominant species responsible for the observed UV/vis absorption at the PBE0/zora-def2-tzvp^40^ level of theory using the CPCM solvation model with acetonitrile as the solvent. The unscaled predicted absorbance maxima for complex **3** with single axial acetonitrile coordination were at 864 and 736 nm, and in good agreement with the experimental values of 920 and 800 nm bands. The predominant transition for the 864 nm excitation was found to be the HOMO–LUMO transition (Figure S34), and for the 736 nm, there were numerous transitions to the LUMO+1 orbital. The Raman vibrations computed at PCM-M06L/def2-TZVP^41–44^ level of theory for this species were found to match the experimental values closely (Figure S35).

These frequencies and the UV/vis spectra were not scaled owing to a lack of rigorously benchmarked scaling factors for this functional. Changing the axial coordination to chlorine or methanol altered the UV/vis absorbance by about 60 nm, and a more complex Raman vibrational spectrum was noticed for these species (Figure S47). The formations of a distorted square planar coordination in **2** and square pyramidal geometry in **3** with a loosely bound acetonitrile were further corroborated through TD-DFT XANES simulations, as elaborated above, which showed good agreement in the pre-edge region with the experimental derivative XANES features (Figure 5c). Intrinsic bond orbital analysis for this species further confirmed that the first oxidation indeed occurs at the ligand, as demonstrated by the orbital occupancy.

TD-DFT computations on the lowest-lying conformations for various two-oxidized species demonstrated the importance of axial coordination. The lack of axial coordination resulted in a complete mismatch with the experimental absorbance maxima, while having two chloride ions as the axial coordination yielded an absorption maximum of 610 nm for the two oxidized species, along with maxima at 1204 and 1012 nm. Investigating the transition responsible for 610 nm absorbance revealed an excitation from the HOMO to the LUMO level (Figure 6). The scaled Raman vibrational modes for these complexes were in good agreement with the experimental values (Figures S27 and S36). However, the computed Raman modes showed much less intensity for the mode around 990 cm^–1^ for both hydroxyl and acetonitrile than for the other axial coordination group.

**Figure 6:**
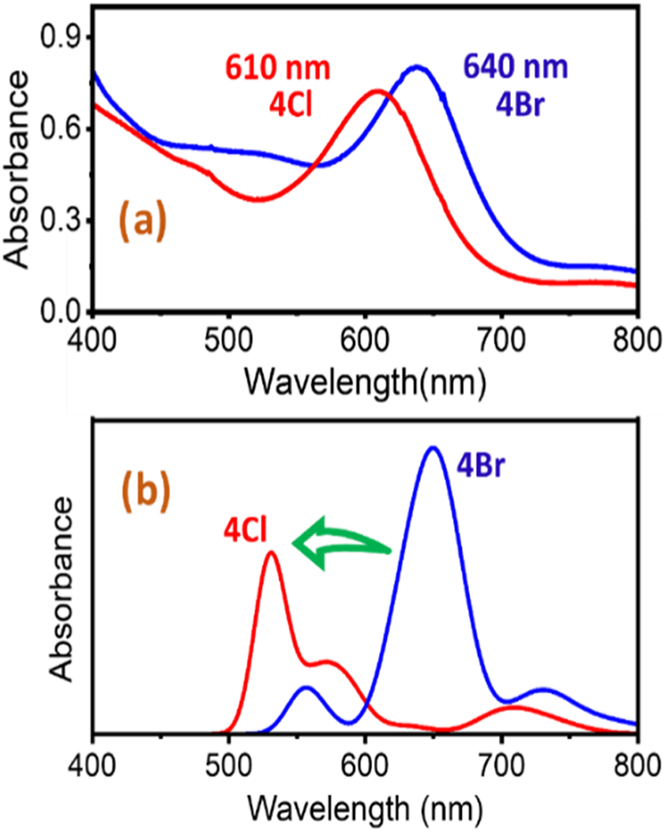
(a) UV/vis absorption spectra observed on adding 3 equiv of TBACl to the 640 nm, **4Br** (blue), resulting in the generation of 610 nm, **4Cl** (red) absorption band. Condition to generate **4Br**: 0.125 mM **2** + 4 equiv of TBABr + 5 equiv of CAN in 1:16 MeOH:MeCN at 233 K. (b) Computed UV/vis absorption spectra for complexes **4Cl** (red) and **4Br** (blue) using TD-DFT at TD-PBE0/ZORA-def2-TZVP in acetonitrile with CPCM model.

Mulliken spin densities shown in Figure 7A indicate that nickel and the ligands have equal but opposite spin densities, indicating that it is an open shell singlet species. Moreover, the open shell singlet species is lower in energy than the closed shell singlet geometry by 7.9 kcal/mol. Intrinsic bond analysis revealed that the electronic configuration of this species is, in fact, **4Cl** (Figure 7) and not [L^•^Ni^III^Cl_2_]. By counting the valence orbitals containing substantial metal contribution (>75%) with d-symmetry within the IBO localization, one can identify the intrinsic d-configuration as described by Knizia and co-workers.^45^ We identified 6 orbitals with δ-symmetry for **4Cl** after applying the intrinsic bond localization schema^45^ on the densities obtained at UM06L/def2-TZVP level of theory indicating that the intrinsic d-configuration is indeed d^6^ for nickel (Figure S37). However, inspecting the electronic configuration using Lowdin population indicated a total of 8 electrons on nickel. However, computing the σ-gain and π-loss as defined by Klein and co-workers^46^ under the intrinsic bond orbital scheme indicated a σ-gain of 2.34 electrons for this complex by the metal center based on the occupancy numbers and a π-loss of 0.26 electrons. Even though the metal center has a d^8^ electron count according to the Lowdin and IPA analysis, the net gain of nearly two electrons (σ-gain is 2.34) by the metal center indicates that the electronic configuration is d^6^ and is consistent with the 6 δ-symmetry IBOs indicated in Figure S37. These values, together with the literature precedence of high-valent metal complexes demonstrating a high σ-gain and a low π-loss, indicate that the electronic configuration is indeed d^6^ for the nickel center.^47^ A similar analysis of complex **3** indicated an intrinsic configuration (Figure S37) as indicated by the 7 valence orbitals with high metal contribution and with δ-symmetry. Finally, second order perturbation analysis in NBO basis also indicates that there is significant electron donation from both chloride ions and the peptide ligand framework (Tables S9–S13).

**Figure 7:**
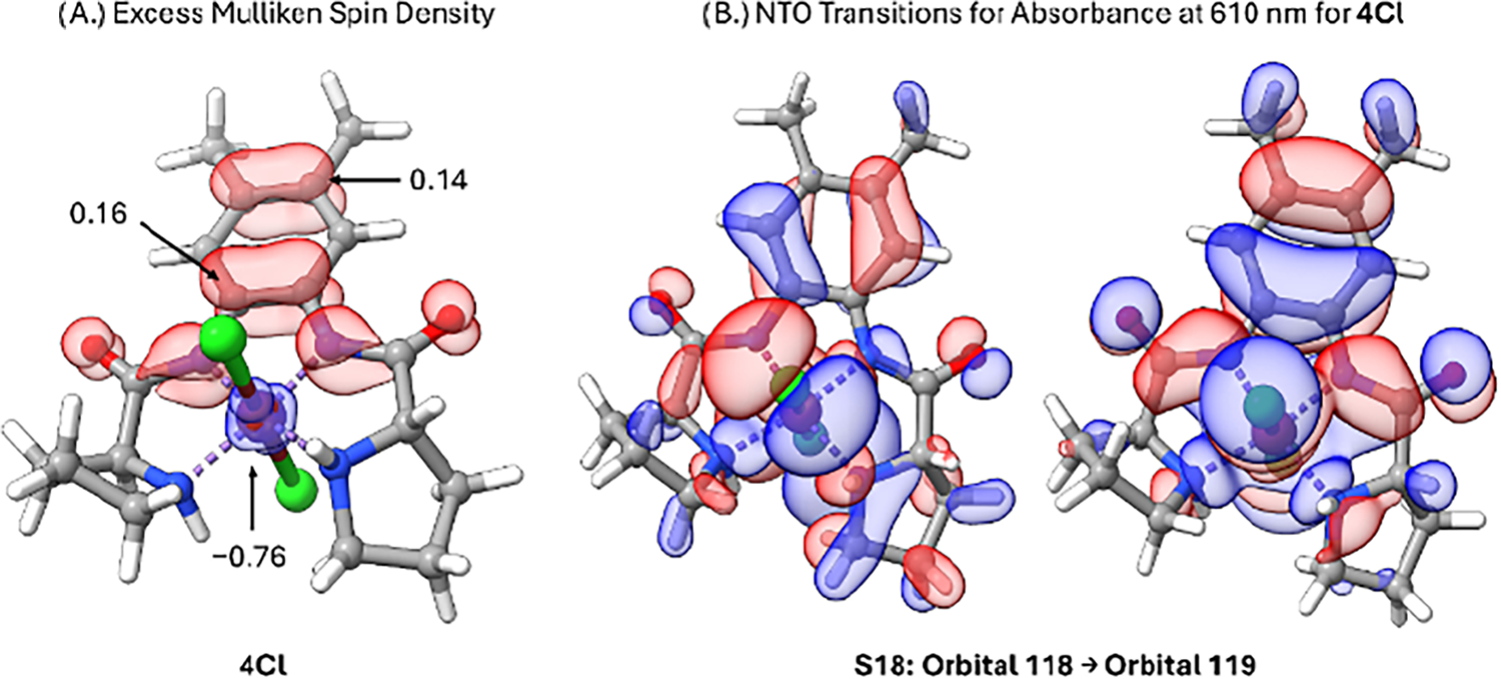
(A) The Mulliken spin densities for **4Cl** and the α-β spin difference plot computed at PCM-acetonitrile: UM06L/def2-TZVP level of theory. (B) Natural transition orbitals for the observed transition for species **4Cl**. The orbitals are computed at TD-PBE0/ZORA-def2-TZVP//UM06L/def2-TZVP in acetonitrile with the CPCM model.

### Reactivity Studies

The reactivity of the high-valent intermediate **4Cl** toward potential oxygen atom transfer (OAT) substrates, including thioanisole and its derivatives, was investigated (Figures 8 and S38). The reaction of **4Cl** with thioanisole and its para-substituted derivatives caused the pseudo first order decay of its characteristic 610 nm absorption band, accompanied by a concomitant increase in the 830 nm band corresponding to the formation of the **3Cl** species (Figure 8a). The formation of **3Cl** rules out the concerted electron transfer and oxygen atom transfer mechanism. The linear decay in the absorbance with the addition of different equivalents of thioanisoles gave the first order rate constants (*k*_obs_), which, plotted against the substrate concentrations, provide the second-order rate constant (*k*_2_). Further, the Hammett studies obtained *k*_2_s delivered the negative ρ value for **4Cl** as −8.3 (Figure 8b), depicting the high electrophilic character of the Ni­(IV) species.

**Figure 8:**
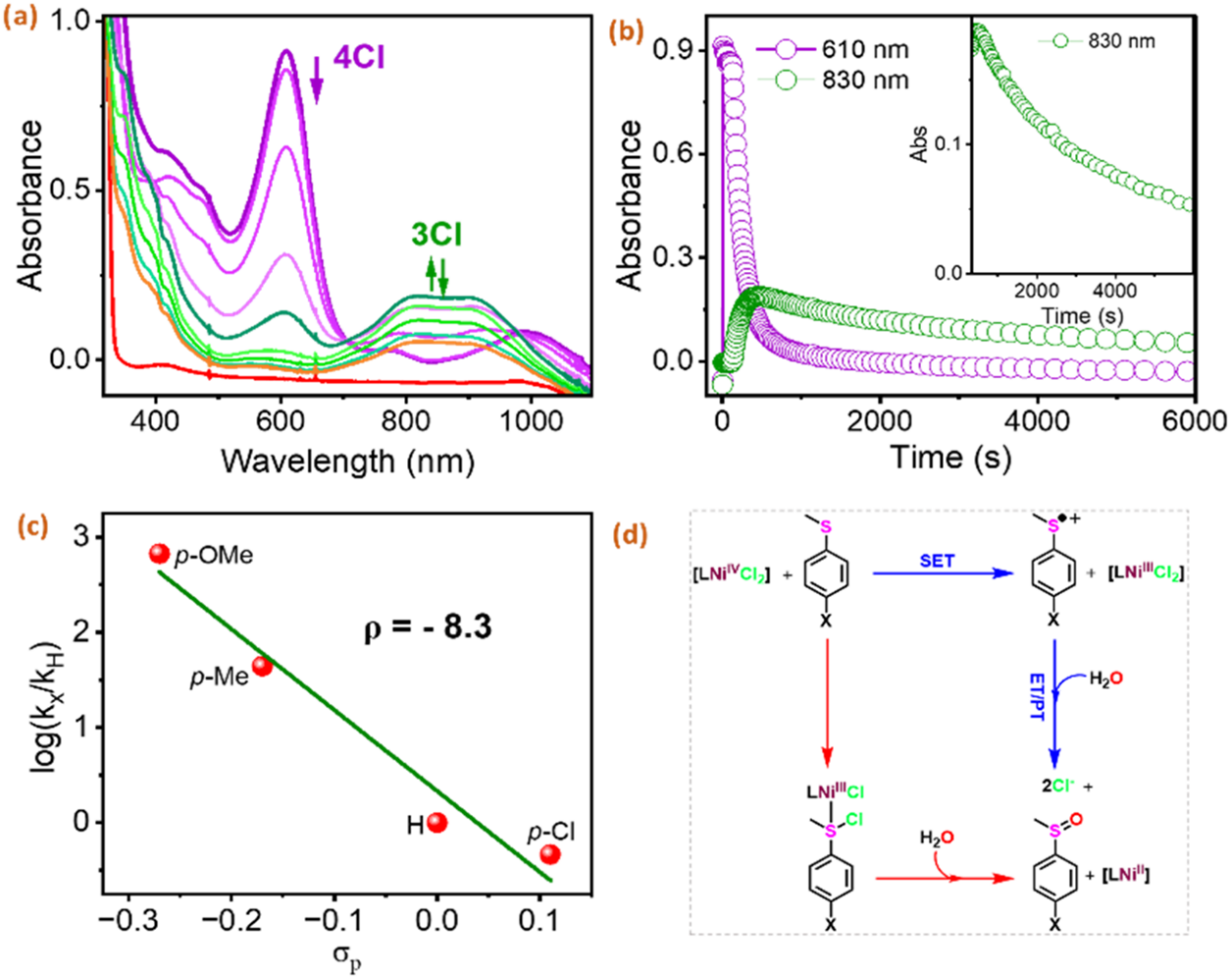
(a) UV/vis spectral changes observed upon the addition of 200 equiv of thioanisole to **4Cl**. *Conditions to generate* **4Cl**: 0.125 mM **2** + 3 equiv of TBACl + 3 equiv of CAN in 1:16 MeOH:MeCN at 233 K. (b) The corresponding changes in the absorbance at 610 and 830 nm. Inset: Zoom in of decay of **3Cl** (followed at 830 nm). (c) Hammett plots of log­(*k_X_*/*k_H_*) against σ_p_ of para-X-thioanisole derivatives by **4Cl** at 233 K. *Hammett constants have been taken from literature.^51^ Relative rate constants (*k_X_*/*k_H_*) have been obtained by dividing *k*_2_ for the reaction with para substituted thioanisole by *k*_2_ for the reaction with thioanisoles. (d) Scheme depicting mechanistic possibilities for the OAT activity demonstrated by **4Cl**.

Two mechanistic possibilities were explored for the oxidation of thioanisoles, as indicated in (Figure 8c). The first route contains a single electron transfer from thioanisole to complex **4Cl**, initially forming the **3Cl** species and the oxidized thioanisole, which then reacts with the in situ water, followed by an electron transfer/proton transfer pathway to yield the sulfoxide. Alternatively, **4Cl** directly transfers the chlorine atom to form the thioanisole complex of [LNi^III^Cl]. This intermediate can then undergo hydrolysis to yield sulfoxide as the final product (Figure S39). Recently, Nam and co-workers reported a similar OAT reactivity with Ni^III^(PaPy3*) (PaPy_3_* = *N*,*N*-bis­(4-methoxy-3,5-dimethyl-2-pyridylmethyl)-amine-*N*-ethyl-2-pyridine-2-carboxamidate) species, where water acts as an oxygen source.^48^ Notably, when the reaction between **4Cl** and thioanisole was performed in the presence of deliberately added water, UV–vis analysis revealed a significant rate enhancement of up to 3.6-fold (Figure S40). These findings underscore the crucial role of H_2_O in facilitating the OAT process in this system. Furthermore, ESI-MS analysis of the reaction conducted with **4Cl** and thioanisole in the presence of minimal addition of H_2_^18^O showed approximately 20% incorporation of ^18^O into the thioanisole oxide product (Figures S39 and S41a–c). However, increasing the amount of added H_2_^18^O resulted in approximately 70% incorporation of ^18^O into the product (Figure S41d,e), supporting the mechanism proposed in Figure 8d. The rate of consumption of **4Cl** (monitored by UV/vis spectroscopy) scales linearly with substrate concentration, indicating that although excess CAN is present, **4Cl** does exhibit some oxidation reactivity with these substrates. S42 illustrates that while the decay of **4Cl** in the presence of 200 equiv of thioanisole requires approximately 300 s to generate **3Cl**, the decay of **3Cl** under identical conditions proceeds significantly more slowly. However, when the reactivity of independently synthesized **3Cl** with thioanisole was examined under the same conditions, **3Cl** exhibited a second-order rate constant of 0.06 M^–1^ s^–1^ (Figure S43). This difference between the two **3Cl** species, one formed *in situ* during the reaction of **4Cl** with thioanisole and the other independently synthesized, supports the mechanistic pathway proposed in Figure 8d. According to this mechanism, the initial reaction of **4Cl** with thioanisole generates a thioanisole cation radical, which then reacts with **3Cl** to yield the oxidized products.

Evaluating both the mechanistic scenarios at UM06L/def2TZVPP// UM06L/def2TZVP level of theory with acetonitrile as the solvent and PCM as the solvent model indicated that the direct chlorine atom transfer has a barrier of 21.3 kcal/mol for the highly activated *p*-methoxythioanisole, demonstrating that the direct chlorine atom transfer pathway might be prohibitive under the experimental conditions. However, the electron transfer pathway was found to be facile for all thioanisole substrates with barriers ranging from 8 to 12 kcal/mol for various thioanisole substrates as indicated by the Marcus theory computations (see SI, Tables S8–S11, Figures S44–S47).^49–53^ The four-point model was used to evaluate the inner sphere reorganization energies of the species involved, while the modified two sphere model was used to evaluate the solvent reorganization energy. The reaction of this oxidized thioanisole radical cation intermediate with water to form the sulfur–oxygen bond was found to be barrierless. Further mechanistic studies for this oxygen atom transfer activity are underway.

## Conclusions

While high-valent Ni compounds are not common, they are increasingly studied and utilized in synthetic chemistry, catalysis, and energy applications. Their rarity in nature and their relatively short-lived nature in synthetic conditions make them unusual. Here **2** is supported by a redox-active ligand that can be oxidized when mixed with one equivalent of CAN, yielding a ligand-centered one-oxidized equivalent species, 2* in 1:16 MeOH:MeCN, with an absorption at 920 nm. However, in the presence of exogenous ligands like Cl^–^, Ni-based oxidation is preferred to form **3Cl** and the λ_max_ shifts to 830 nm (Scheme 1). The occurrence of this Ni localized one oxidized equivalent species, **3**, is also facilitated at a temperature lower than 233 K (used for UV/vis and resonance Raman spectroscopies) to 77 and 15 K, as in EPR and EXAFS, respectively. In the absence of any external exogenous ligand, solvent MeCN acts as one, favoring the formation of [LNi^III^(NCMe)]. This connotes the existence of a temperature-dependent equilibrium in one oxidized equivalent species similar to what is observed with the Ni^II^(salen) complexes.^22,23^ Furthermore, the addition of CAN to **3Cl** generates a high valent species having λ_max_ at 610 nm. Interestingly, only the presence of exogenous ligands like Cl^–^ or Br^–^ makes this high-valent species stable enough to be characterized. EPR at 77 K gives a silent signal for an S = 0 species, implying the formation of either [LNi^IV^Cl_2_] or [L^•^Ni^III^Cl]. This perplexity is analogous to the recently reported *formal* Ni­(V) species, where the presence of imidazole in the system aids the generation of an octahedral species with the formation of [Ni^III^-MI_2_L^••^].^26^ Favorably in the current study, the EXAFS of the 610 nm absorbing species performed at 15 K reveals the formation of a high-valent Ni^IV^ complex bound to two chloride ligands, [LNi^IV^Cl_2_], **4Cl**. The findings from EXAFS are also assisted by DFT calculations, making this discovery a first.

The study thus underscores the complex behavior of nickel complexes with a biologically relevant pseudo peptide ligand under different conditions as a concluding remark. The knowledge is precisely adjusted to fit applications by modifying the ligand structure and coordination environment. This makes them adaptable options for a variety of scientific and technological projects. These results yield new perspectives on the genesis of high-valent Ni species, enhancing our comprehension of their stability and reactivity when exogenous ligands are present.

## Experimental Section

All commercially available chemicals and reagents used in the present study were used as received. HPLC grade dry MeCN, MeOH, and DMF from Thomas Baker were used in the spectroscopic studies.

UV/vis absorption spectroscopic studies were performed by an Agilent 8453 diode-array spectrophotometer to carry out kinetics experiments spectrophotometrically in 1 cm quartz cells (λ = 190–1100 nm range). A low temperature of 233 K was maintained with a cryostat from a CoolSpeK USP-203-B Unisoku cryostat. Sample cooling was performed using a CoolSpeK USP-203-B Unisoku cryostat. X-band EPR spectra were recorded at 77 K using a Bruker EMX 1444 spectrometer with a temperature controller. Electrochemical analyses were done at room temperature in MeCN through cyclic voltammetry experiments using the CH instrument, Electrochemical Analyzer M-600B series. A three-electrode system, where a glassy carbon (CHI 104 Glassy Carbon Disk Working Electrode) was used as the working electrode, a Pt wire as the counter electrode, and an aqueous Ag/AgCl electrode was used as the reference electrode. The solutions used were 2 mM **2** and 100 mM of tetra-*n*-butylammonium perchlorate (TBAClO_4_) as the supporting electrolyte. Elemental analysis (C, H, N) of **2** was obtained using a PerkinElmer CHNS/O 2400 series II Analyzer. Raman spectra were obtained using a λ_exc_ of 405 and 638 nm from Cobolt Lasers (75 mW at source). Spectra were recorded using a 180° backscattering arrangement. Raman scattering was collected by a plano-convex lens (2.5 cm diameter, *f* = 7.5 cm). The collimated Raman scattering was then passed through a long-pass edge filter from Semrock and focused by a second plano-convex lens (2.5 cm diameter, *f* = 7.5 cm) into a Shamrock300i spectrograph from Andor Technology with a 1200 L/mm grating blazed at 500 nm, acquired with an iDus-430-BV CCD camera from Andor Technology. The spectral slit width was set to 100 μm.

### X-ray Absorption Spectroscopy (XAS) Methods

X-ray absorption spectra were collected at an SSRL light source at Stanford University (USA) on a wiggler beamline at an electron energy of 8.33 keV and an average current of 100 mA. The radiation was monochromatized by Si(220) crystal monochromator. The intensity of the X-rays was monitored by three ion chambers (I_0_, I_1,_ and I_2_) filled with 70% nitrogen and 30% argon and placed before the sample (I_0_) and after the sample (I_1_ and I_2_). Ni metal was placed between ion chambers I_1_ and I_2,_ and its absorption was recorded with each scan for energy calibration. Ni XAS energy was calibrated by the first maxima in the second derivative of the Nickel′s metal foil’s X-ray absorption near edge structure (XANES) spectrum. The samples were kept at 15 K in a He atmosphere at ambient pressure and recorded as fluorescence excitation spectra using a 26-element energy-resolving Ge detector. The solution complexes were measured in the continuous helium flow cryostat in fluorescence mode. Around 10 XAS spectra of each sample were collected. Care was taken to measure at several sample positions on each sample, and no more than 5 scans were taken at each sample position. In order to reduce the risk of sample damage by X-ray radiation, 80% flux was used (beam size 6000 μm­(Horizontal) × 1000 μm­(Vertical)), and no damage was observed scan after scan to any samples. All samples were also protected from the X-ray beam during spectrometer movements by a shutter synchronized with the scan program. Ni XAS energy was calibrated by the first maxima in the second derivative of the Nickel′s metal X-ray Absorption Near Edge Structure (XANES) spectrum.

### Extended X-ray Absorption Fine Structure (EXAFS) Analysis

Athena software^54^ was used for data processing. The energy scale for each scan was normalized using the Nickel metal standard. Data in the energy space were pre-edge corrected, normalized, deglitched (if necessary), and background corrected. The processed data were next converted to the photoelectron wave vector (*k*) space and weighted by *k*. The electron wavenumber is defined as *k* = [*2m­(E–E*_0_ *)*/*ℏ* ^2^]^1/2^, *E*_0_ is the energy origin or the threshold energy. *K*-space data were truncated near the zero crossings, *k* = 2 to 14.151 Å^–1^ in Ni EXAFS, before Fourier transformation. The *k*-space data were transferred into Artemis Software for curve fitting. To fit the data, the Fourier peaks were isolated separately and grouped together, or the entire (unfiltered) spectrum was used. The individual Fourier peaks were isolated by applying a Hanning window to the first and last 15% of the chosen range, leaving the middle 70% untouched. Curve fitting was performed using *ab initio*-calculated phases and amplitudes from the FEFF8 ^52^ program at the University of Washington. *Ab initio*-calculated phases and amplitudes were used in EXAFS eq 1.

*where Nj* is the number of atoms in the *j*th shell; *R*_*j*_ is the mean distance between the absorbing atom and the atoms in the *j*th shell; *f*_*eff*_*j*__ (π,*k*,*R*_*j*_) is the *ab initio* amplitude function for shell *j*, and the Debye–Waller term $e^{-2\sigma_{j}^{2}k2}$ accounts for damping due to static and thermal disorder in absorber-backscatterer distances. The mean free path term $e^{-2R_{j}/\lambda_{j}\left(k\right)}$ reflects losses due to inelastic scattering, where λ*j­(k)*, is the electron mean free path. The oscillations in the EXAFS spectrum are reflected in the sinusoidal term sin­(2*kR*_*j*_ + ϕ_*ij*_ *(k))*, where ϕ_*ij*_ *(k)* is the *ab initio* phase function for shell *j*. This sinusoidal term shows the direct relation between the frequency of the EXAFS oscillations in *k*-space and the absorber-backscatterer distance. *S*_0_^2^ is an amplitude reduction factor.

1 $$\chi \left(k\right)=S_{0}^{2}\sum_{j}\frac{N_{j}}{kR_{j}^{2}}f_{{e\mathrm{ff}}_{j}}\left(\pi ,k,R_{j}\right)e^{-2\sigma_{j}^{2}k^{2}}e^{-2R_{j}/\lambda_{j}\left(k\right)}\sin\left(2kR_{j}+\phi_{\mathrm{ij}}\left(k\right)\right)$$

The EXAFS equation^55^ (eq 1) was used to fit the experimental Fourier isolated data (*q*-space) as well as unfiltered data (*k*-space), and Fourier transformed data (*R*-space) using *N*, *S*_0_^2^, *E*_0_, *R*, and *s* ^2^ as variable parameters. *N* refers to the number of coordination atoms surrounding Ni for each shell. The quality of fit was evaluated by the *R*-factor (eq 2) and the reduced Chi^2^ value. The deviation in *E*_0_ ought to be less than or equal to 10 eV. *R*-factor less than 2% denotes that the fit is good enough^56^ whereas *R*-factor between 2 and 5% indicates that the fit is correct within a consistently broad model. The reduced Chi^2^ value is used to compare fits as more absorber-backscatter shells are included to fit the data. A smaller reduced Chi^2^ value implies a better fit. Similar results were obtained from fits done in *k*, *q*, and *R*-spaces.

2 $$R‐\mathrm{factor}=\frac{\sum_{i}{\left(\mathrm{difference}\;\mathrm{between}\;\mathrm{data}\;\mathrm{and}\;{\mathrm{fi}t}_{i}\right)}^{2}}{\sum_{i}{\left(\mathrm{data}\right)}^{2}}$$

### Pre-Edge Area Fits

The near-edge and pre-edge peak fits were carried out with an error function and Gaussian functions, respectively. The formulas for the error (erf) and Gaussian functions (gauss) are as follows:

3 $$e\mathrm{rror function}:A\left[\mathrm{erf}\left(\frac{e-E_{0}}{w}\right)+1\right]$$

4 $$\mathrm{Gaussian function}:\left(\frac{A}{w\sqrt{2\pi }}\right)\exp\left[\frac{-{\left(e-E_{0}\right)}^{2}}{\left(2w^{2}\right)}\right]$$

Where *A* corresponds to the amplitude; *w*, the width; *E*_0_, the centroid of the pre-edge and near-edge peaks; and *e*, the X-ray energy. The parameters *E*_0_, *A*, and *w* used for each set of functions for the experimental and theoretical fits, together with their uncertainties, are tabulated below (Table S4).

The pre-edge area peaks fitting were further recarried out in the Fityk^55^ software, and as previously demonstrated,^57^ and the same pre-edge peak areas of 5.2, 4.5, and 4.0 were obtained for **2, 3**, and **4Cl**, thus confirming the fit procedure employed in the Athena software.^54^

### DFT Calculations

The DFT optimization calculations were performed using the ORCA (Version 5.0) program package developed by Neese^39^ and co-workers. The geometry optimizations were carried out using the solid-state (XRD) as a starting point. The calculations were carried out using the BP86 exchange-correlation functional^58^ in combination with the triple-ζ valence polarization functions (def2-TZVP),^41^ and the atom-pairwise dispersion correction with the Becke-Johnson damping scheme (D3BJ)^59,60^ and the CPCM solvent polarization model.

The RI approximation was used to accelerate Coulomb and exchange integrals for the ground and excited state calculations, respectively.^61^ The default GRID settings were further used for the self-consistent field iterations and the final energy evaluation. The calculated structures were confirmed to be minima based on a check of the energies and the absence of imaginary frequencies from frequency calculations carried out on the optimized geometries.

### Time-Dependent (TD)-DFT XANES Calculations

Time-dependent DFT (TD)-DFT calculations for the XANES spectra of the Ni complexes were carried out using the hybrid-DFT functional. The TD-DFT XANES simulations were, in this case, performed with the B3LYP^62,63^ as functional with the def2-TZVP triple-ζ^42^ basis sets together with the ZORA approximation and D3BJ dispersion correction effects with dense integration grids. The def2-TZVP/J auxiliary basis set was also employed. The XANES absorption spectra from the TD-DFT calculations were shifted in energy by +180.1 eV relative to the experimental data, as previously demonstrated,^64,34,65^ and a broadening of 2.0 eV was applied to all calculated spectra. Up to 150 roots were calculated. The calculated XANES spectrum contains contributions from electric quadrupole, electric dipole, and magnetic dipole transitions. All spectra were broadened with a Gaussian line shape of 2.0 eV (fwhm).

## Supplementary Material



## Notes

Synthetic procedure for Ligand (L) and complex **2**; cyclic voltammetry of **2**; EPR, rRaman, XAS of **2**, **3**, **4Cl**; Reactivity of **4Cl** with thioanisole and DFT calculations (PDF)
