# Supplementary material for: Spectroscopically Deciphering the Formation and Reactivity of a High-Valent Ni(IV)Cl2 Species
Source: JACS Au. 2025 Dec 16;6(1):245–57. doi: 10.1021/jacsau.5c01182 (PMC12848696; doi:10.1021/jacsau.5c01182)
Supplement: Supplementary file 1 [file au5c01182_si_001.pdf]

Supporting Information

Spectroscopically Deciphering the Formation and Reactivity of a High-Valent $Ni(IV)Cl_2$ Species

Ayushi Awasthi,[†] Kiran Bhadauriya,[†] Lucia Velasco,[¥] Raju Eerlapally,[†] Asterios Charisiadis,[¥] Rakesh Kumar,[†] Maxime Sauvan,[¥] Dooshaye Moonshiram,[*,¥] Sharath Chandra Mallojjala[*,‖] and Apparao Draksharapu[*,†]

[†]Southern Laboratories-208A, Department of Chemistry, Indian Institute of Technology Kanpur, Kanpur-208016, India.

[¥]Instituto de Ciencia de Materiales de Madrid Consejo Superior de Investigaciones Científicas Sor Juana Inés de la Cruz, 3, Madrid 28049, Spain.

[‖]Department of Chemistry, Binghamton University, Binghamton, New York 13850, United States.

Experimental Section

Synthesis of (Me_2OPDProline) (L)

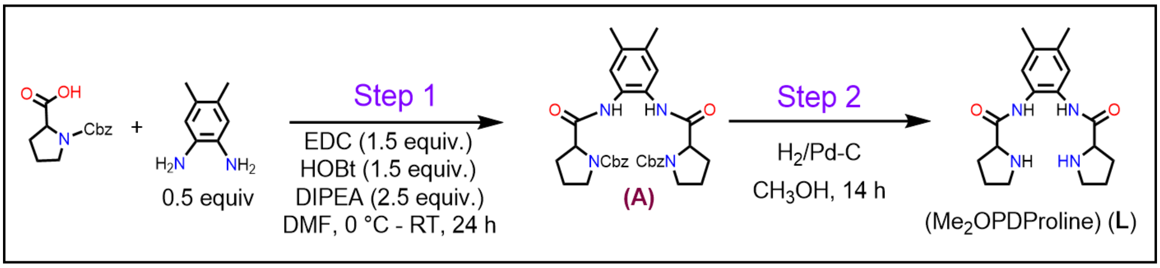

Figure S1. Schematic outline representing the synthesis of (Me_2OPDProline) ligand (**L**).

Step 1: *Synthesis of dibenzyl 2,2'-(((4,5-dimethyl-1,2 phenylene)bis(azanediyl))bis(carbonyl))bis(pyrrolidine-1-carboxylate)* **(A)**

Synthesis of **(A)** was carried out using a modified literature procedure.[1] N-Cbz-L-Proline (1 g, 4.01 mmol) was dissolved in 3 ml DMF (N,N-Dimethylformamide) followed by the addition of coupling reagent EDC (1-Ethyl-3-(3-dimethylaminopropyl)carbodiimide) (1.16 g, 6.02 mmol) in the presence of additive 1-hydroxybenzotriazole (HOBt) (813 mg, 6.02 mmol) in a 50 ml round bottom flask equipped with a magnetic stirring bar at 0 °C. After 20 minutes, base N,N-Diisopropylethylamine (DIPEA) (1.75 ml, 10.02 mmol) was added to the reaction mixture, followed by the addition of 4,5-Dimethyl-1,2-phenylenediamine (273.1 g, 2 mmol) and the reaction was allowed to reach the ambient temperature for the next 24 hours. After 24 hours water (200 ml) was added to the reaction mixture and extracted with ethyl acetate (150 ml). The collected organic layer was washed with saturated $NaHCO_3$ (200 ml), 1 N HCL (200 ml), and Brine solution (200 ml). The organic layer was collected, dried over anhydrous Na_2SO_4, and filtered. The solvent from the filtrate was removed on a rotary evaporator to give a white solid. The white solid is called (**A**) and is a precursor to (Me_2OPDProline) and has a yield of 42%.

Step 2: *Synthesis of N,N'-(4,5-dimethyl-1,2-phenylene)bis(pyrraolidine-2-carboxamide)* ***[(Me_2OPDProline] (L)***

The carbobenzoxy protecting groups were removed using the modified literature procedure method.[2] dibenzyl 2,2'-(((4,5-dimethyl-1,2-phenylene)bis(azanediyl))bis(carbonyl))bis(pyrrolidine-1-carboxylate) (500 mg, 0.84 mmol) was dissolved in 20 ml of methanol into a 100 ml round bottom flask equipped with hydrogenation setup. To the mixture, (8.90 mg, 0.08 mmol) of palladium on charcoal (10%) catalyst was added, and the hydrogen gas was purged into the mixture through a t-shaped three-way hollow plug connected with a hydrogen balloon. As the reaction was air-sensitive,

the solution was degassed with N_2 and the N_2–atmosphere was exchanged for an H_2–atmosphere (1 bar). The reaction was stirred vigorously for 14 h. The catalyst was filtered, and the solvent was removed on a rotatory evaporator to give a white solid with 87% yield.).^{1}H NMR (500 MHz, $CDCl_3$): d (ppm) 9.58 (s, 2H), 7.39 (s, 2H), 3.87 (dd,2H, J = 5.35, 3.8 Hz), 3.45 (s, 2H), 3.07-2.95 (m, 4H), 2.20 (s, 6H), 2.07-1.99 (m, 2H), 1.82-1.70 (m, 4H). ^{13}C NMR (125 MHz, $CDCl_3$): d (ppm) 173.79, 135.19, 128.18, 126.21, 61.62, 47.97, 31.49, 26.66, 20.18.

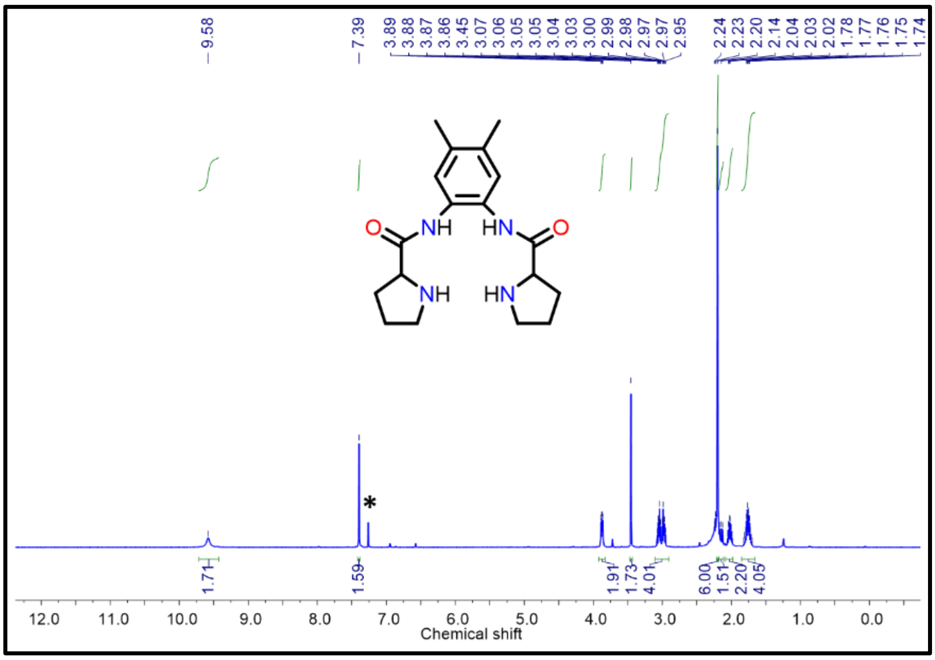

Figure S2. ^{1}H NMR spectrum of (Me_2OPDProline) in $CDCl_3$ at 500 MHz.

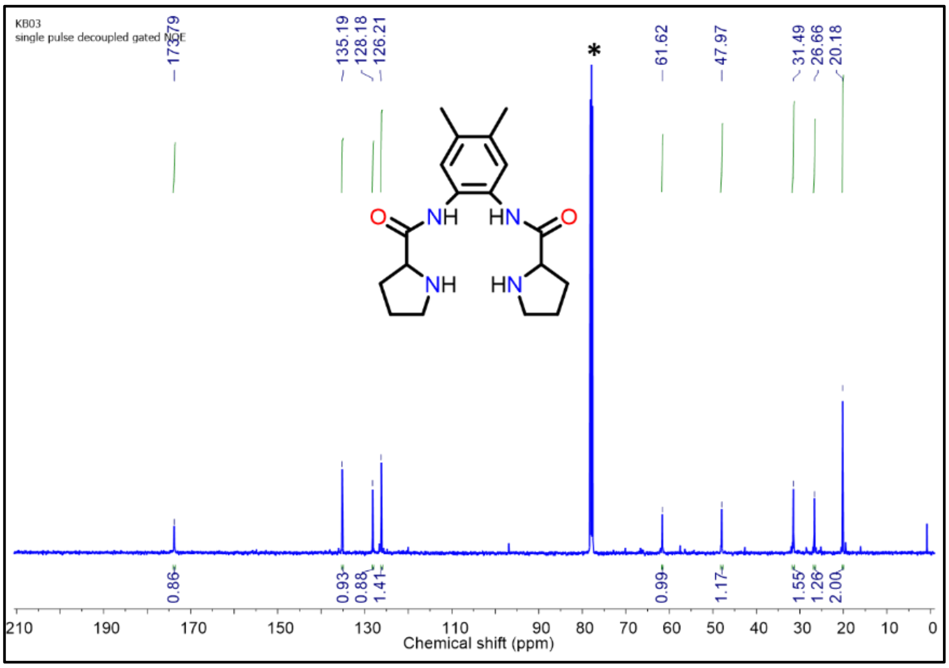

Figure S3. ^{13}C NMR spectrum of (Me_2OPDProline) in $CDCl_3$ at 125 MHz.

Synthesis and characterization of [(Me$_2$OPDProline)Ni^{II}], [LNi^{II}] (2):

Synthesis of **2** was carried out by a modified literature procedure.[1] Ni(OAc)$_2$ ·4H$_2$O was dissolved in 3.5 ml HPLC water and added dropwise to the 3.6 ml methanolic solution of (Me$_2$OPD)proline) (100 mg, 0.303 mmol) in a 50 ml round bottom flask equipped with magnetic stirring in an oil bath at 70 °C. The brown solution of the ligand turned pale yellow immediately, and a precipitate was formed. The reaction was kept on stirring for 4 h, and after that, the solvent was removed on a rotatory evaporator, and yellow crystals were obtained in the round bottom. The above crystals were recrystallized in methanol under ether vapor diffusion. (20 mg **2** was taken in 2 ml methanol solution and kept in a diethyl ether bath for slow vapor diffusion. Within 2-3 days, yellow crystals of **2** suitable for X-ray analysis were obtained. Yield: 93%. UV/vis: 418 nm (160 M^{-1} cm^{-1} in 1:16 MeOH: CH$_3$CN). ^{1}H NMR (400 MHz, DMSO-d_6): δ (ppm) 7.83 (s, 2H), 4.63 (q, 2H, J = 6.78 Hz), 3.53 (q, 2H, J = 7.7 Hz), 3.30 - 3.24 (m, 2H) 2.79 - 2.72 (m, 2H), 2.04 - 1.88 (m, 2H), 2.00 (s, 6H), 1.81 - 1.73 (m, 2H), 1.65 - 1.55 (m, 2H). Anal. Calc. for NiC$_{18}$H$_{24}$N$_4$O$_2$.H$_2$O: C, 53.37; N, 13.83; H, 6.47. Found: C, 52.78; N, 13.68; H, 6.18.

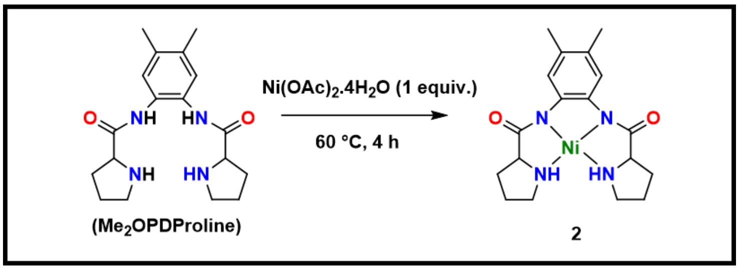

Figure S4. Schematic outline representing the synthesis of **2**.

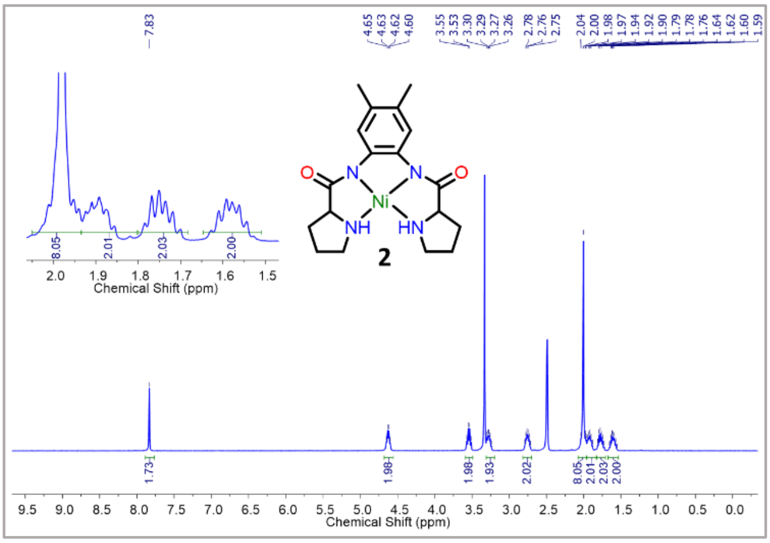

Figure S5. ^{1}H NMR spectrum of **2** in DMSO-d_6 at 400 MHz. This spectrum suggests that **2** is a diamagnetic S = 0 species (*i.e.*, Square planar complex).

Crystal data of [(Me_2OPDProline)Ni^{II}] (2)

The single crystal suitable for X-ray diffraction (XRD) was grown via diffusion method in the methanol (MeOH) and diethyl ether (Et_2O) for **2** at room temperature. Complex **2** exhibits an *orthorhombic* crystal system with the '$P2_12_12_1$' space group. The structure features a distorted square planer coordination environment around the central Ni(II) ion, which is coordinated by two anionic nitrogen of amide moiety and two pyrrole nitrogen in the ligands. The amide bonds in the ligands coordinate to the nickel center through the nitrogen atoms, forming a five-membered chelate ring, with the bond lengths of Ni(1)–N(2) and Ni(1)–N(3) being 1.833(3) Å and 1.832(2) Å, respectively. The pyrrole's nitrogen coordinate to the nickel center with bond lengths of Ni(1)–N(1) and Ni(1)–N(4) being 1.925(2) Å and 1.926(3) Å, respectively. These distances were found to be slightly longer compared to amide bonds as expected. One MeOH solvent molecule was also found in the unit cell as a solvent of crystallization. The two amide anions present in the ligand framework neutralize the charge of the Ni(II) center.

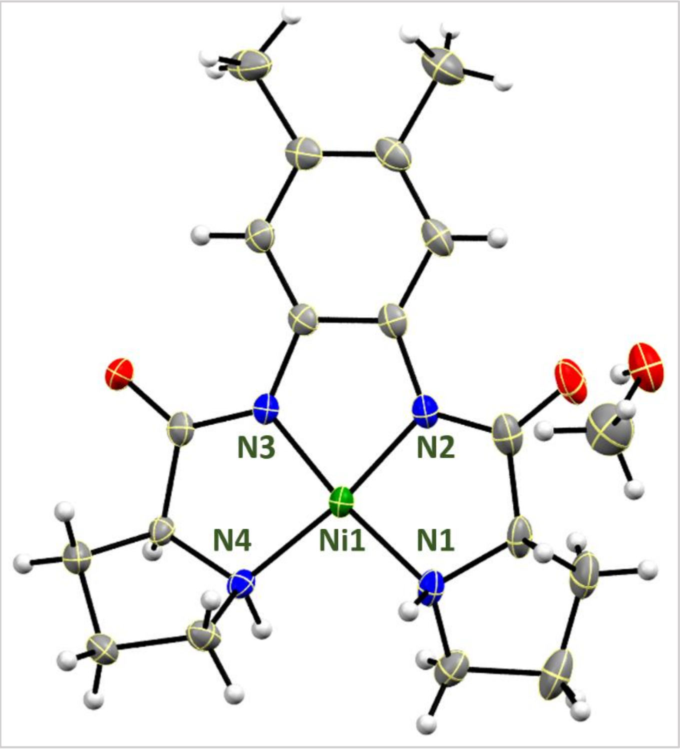

Figure S6. X-ray crystal structure of **2** collected at 100 K. Thermal ellipsoids are drawn at the 50% probability level.

Table S1. Crystal data and structure refinement for **2**.

Empirical formula	$C_{38}H_{56}N_8Ni_2O_6$
Formula weight	838.32
Temperature/K	100(2)
Crystal system	orthorhombic
Space group	$P2_12_12_1$
a/Å	7.9170(2)
b/Å	21.0927(7)
c/Å	23.0175(7)
α/°	90
β/°	90
γ/°	90
Volume/Å^3	3843.7(2)
Z	4
ρ_{calc}g/cm^3	1.449
μ/mm^{-1}	1.037
F(000)	1776.0
Crystal size/mm^3	0.22 × 0.2 × 0.18
Radiation	MoKα (λ = 0.71073)
2Θ range for data collection/°	5.442 to 56.698
Index ranges	-10 ≤ h ≤ 10, -28 ≤ k ≤ 28, -30 ≤ l ≤ 30
Reflections collected	62902
Independent reflections	9600 [R_{int} = 0.0592, R_{sigma} = 0.0407]
Data/restraints/parameters	9600/0/496
Goodness-of-fit on F^2	1.030
Final R indexes [I>=2σ (I)]	R_1 = 0.0321, wR_2 = 0.0706
Final R indexes [all data]	R_1 = 0.0373, wR_2 = 0.0732
Largest diff. peak/hole / e Å^{-3}	0.53/-0.42
Flack parameter	0.037(11)

Table S2. Bond Lengths for **2**.

Bond	Length/Å
Ni1-N1	1.925(2)
Ni1-N2	1.833(3)
Ni1-N3	1.832(2)
Ni1-N4	1.926(3)

Table S3. Bond Angles for **2**.

	Angle/°
N1-Ni1-N2	86.84(11)
N1-Ni1-N4	100.06(11)
N1-Ni1-N3	172.77(11)
N4-Ni1-N2	173.03(11)
N3-Ni1-N2	86.12(11)
N3-Ni1-N4	87.01(11)

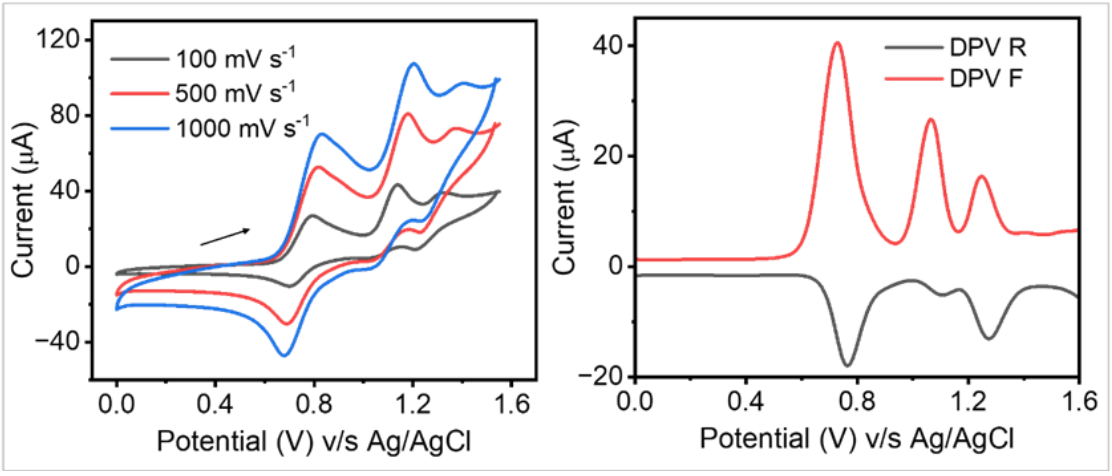

Figure S7. Cyclic voltammograms at different scan rates (left) and differential pulse voltammetry (DPV) (right) of 1 mM **2** in 1:16 MeOH:MeCN.

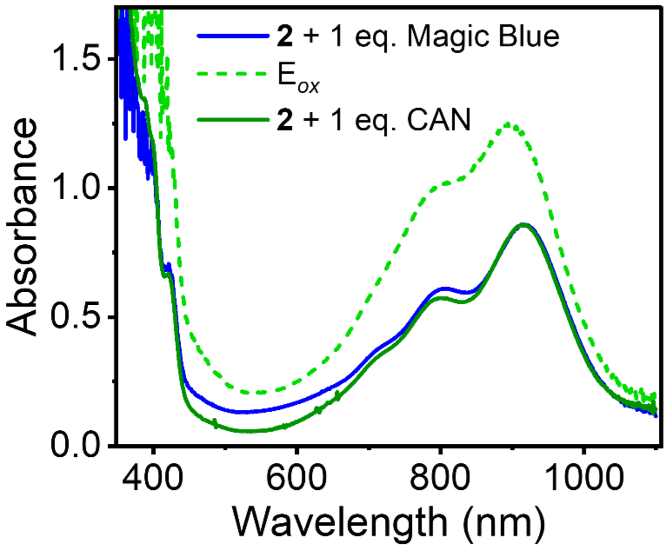

Figure S8. UV/vis absorption spectra of one-electron oxidized species (**2***), generated by the reaction of 0.25 mM **2** in 1:16 MeOH:MeCN with magic blue (blue trace) and CAN (green trace) at 233 K. The spectrum of **2*** (light green, dashed line) generated by electrochemical oxidation of **2** in 100 mM $TBAClO_4$ in 1:2 MeOH:MeCN at 0.78 V vs Ag/AgCl at 298 K.

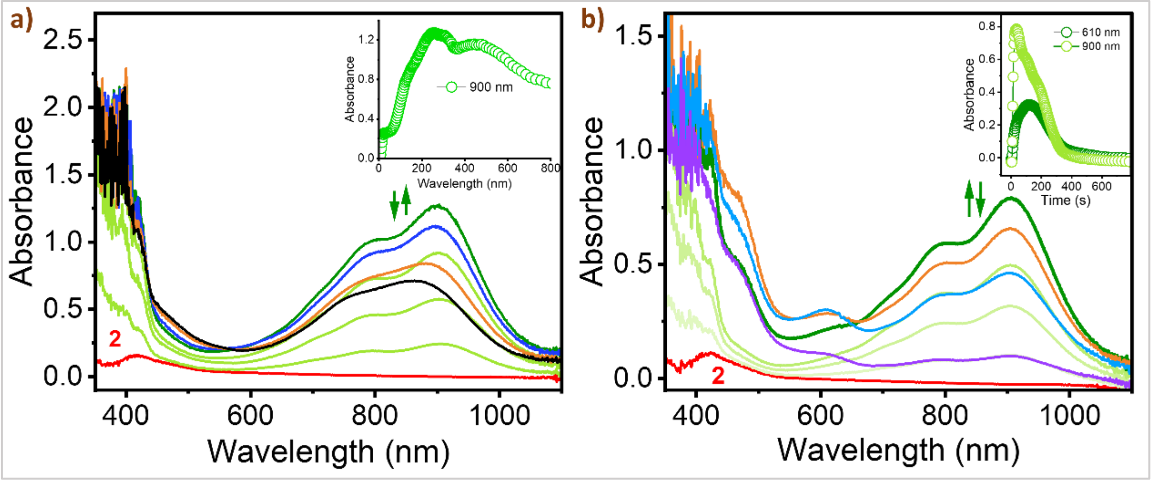

Figure S9. Spectroelectrochemical oxidation of 1 mM of **2** in 100 mM of $TBAClO_4$ in 1:3 MeOH:MeCN with the applied potentials of a) 0.78 V, and b) 1.2 V vs Ag/AgCl at 298 K.

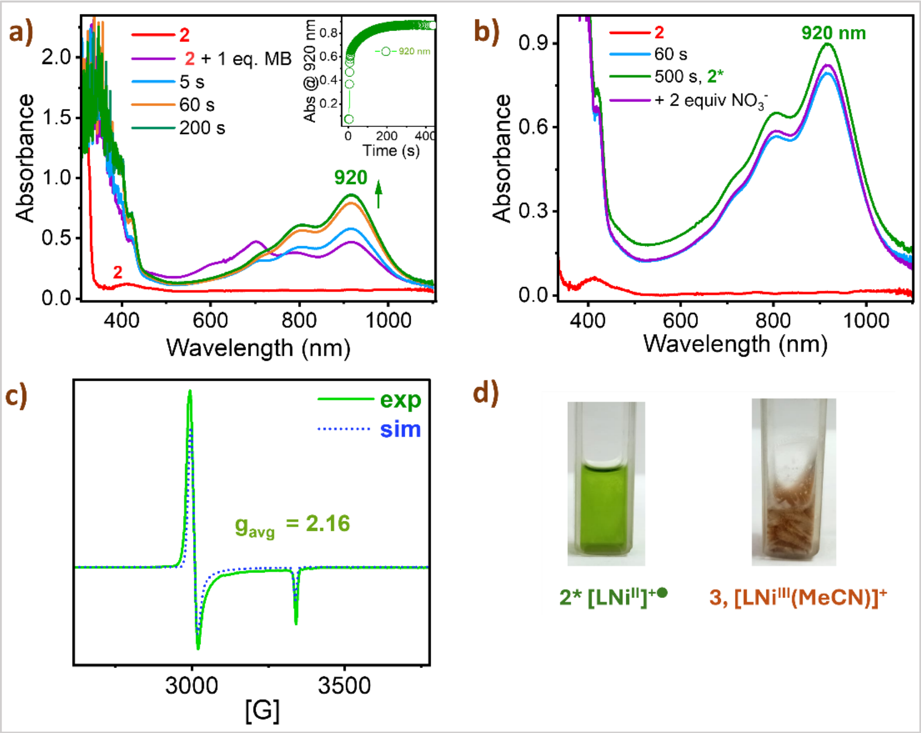

Figure S10. a) UV/vis absorption spectral changes observed upon reacting 0.125 mM **2** in 1:16 MeOH:MeCN with 1 equiv of MB at 233 K to produce **2***. b) Effect of tetra butyl ammonium nitrate (TBANO$_3$) addition to **2*** (generated with magic blue). *Condition to generate* ***2****: 0.125 mM **2** in 1:16 MeOH:MeCN + 1 equiv of MB in CH_3CN at 233 K. c) X-band EPR of **2*** generated by adding of 1 equiv of MB to 2 mM **2** (green: experimental; blue: simulated). d) Picture representation of the green color of **2*** (1 mM **2** + 1 eq. CAN) in solution changes to orange upon freezing at 77 K in 1:16 MeOH:MeCN.

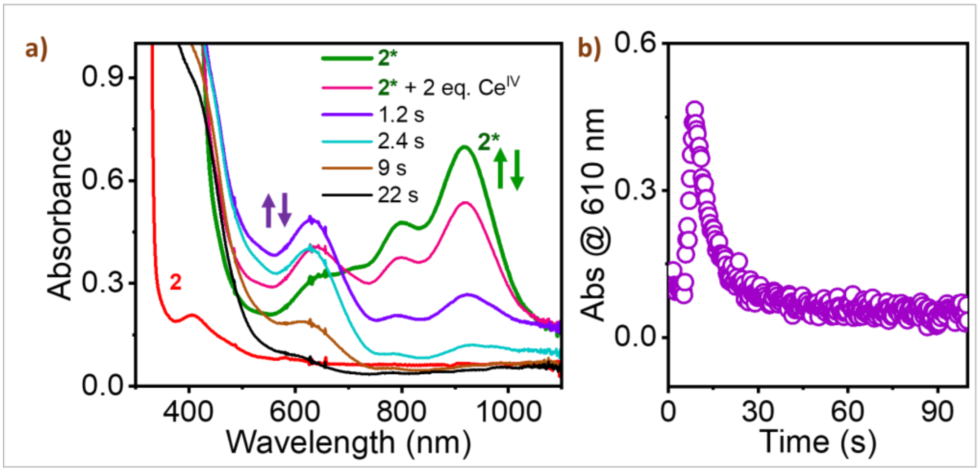

Figure S11. a) UV/vis absorption spectra observed on adding 2 equiv of CAN to one oxidized equivalent species, **2***. b) Absorption changes corresponding to immediate formation and decay of the 610 nm absorption band that dies in the course of 10-15 s at 233 K in 1:16 MeOH:MeCN. *Conditions to generate* **2***: 0.25 mM **2** + 1 equiv of CAN in 1:16 MeOH:MeCN at 233 K.

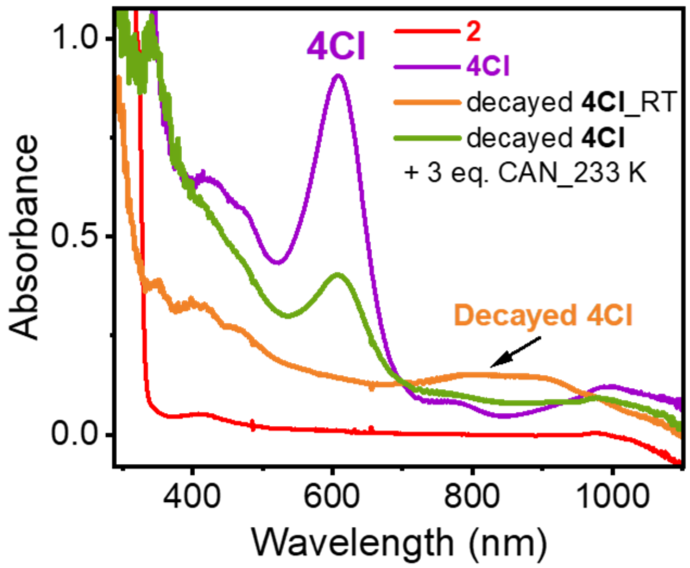

Figure S12. a) UV/vis absorption spectra of **4Cl** at 233 K (purple), and its decayed spectrum (orange) at 298 K. To the decayed species of **4Cl**, adding 3 equiv of CAN regenerates **4Cl** (green) in 45% yield at 233 K. Conditions to generate **4Cl**: 0.125 mM **2** in 1:16 MeOH:MeCN + 3 equiv of TBACl + 3 equiv of CAN at 233 K.

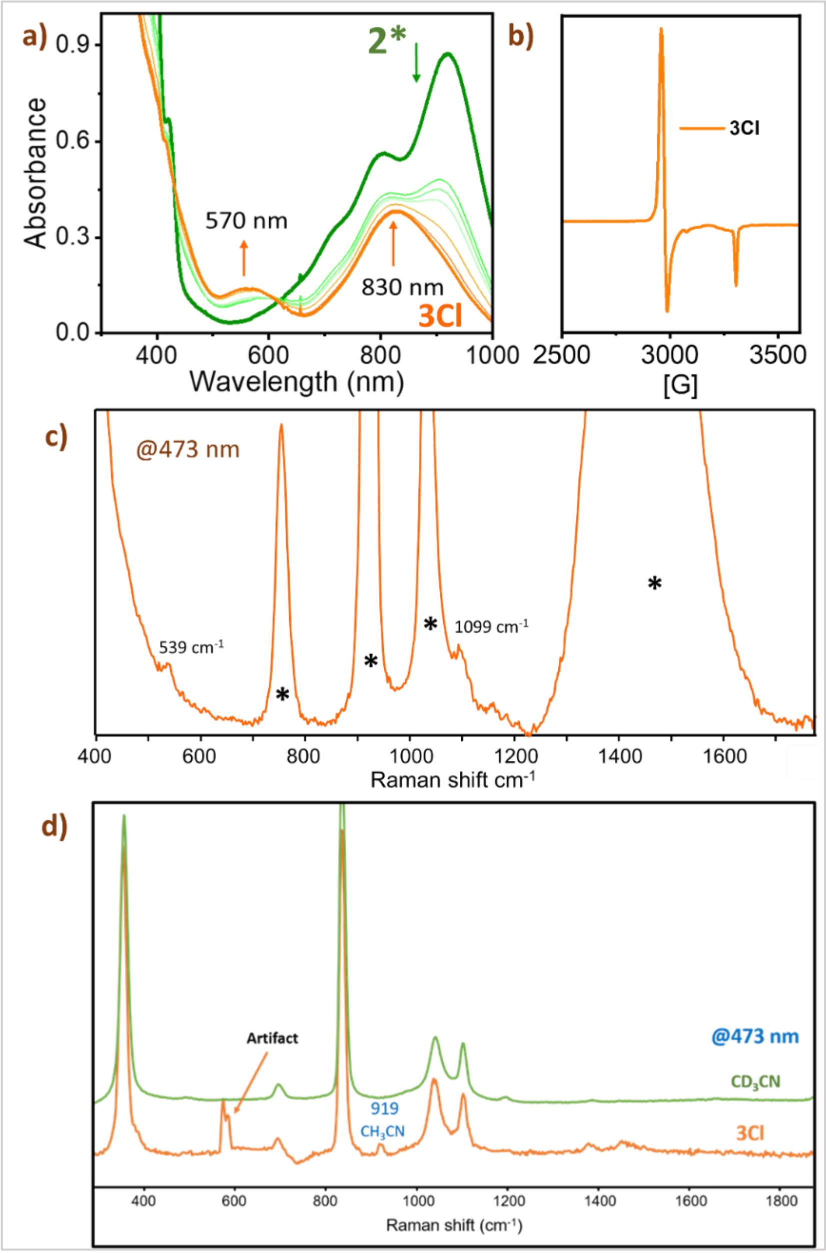

Figure S13. a) UV/vis absorption spectra observed on adding 3 equiv of TBACl to one oxidized equivalent species (**2***). *Conditions to generate **2****: 0.25 mM **2** + 1 equiv of CAN in 1:16 MeOH : MeCN at 233 K. b) X band (9.45 GHz) EPR spectrum of **3Cl** measured at 77 K. *Conditions to generate **3Cl***: 2 mM **2** in 1:16 MeOH:MeCN + 1 equiv of CAN (in MeCN) + 3 equiv of TBACl (in MeCN) at 233 K. Modulation amplitude 1.98 G; modulation frequency 100 kHz, and attenuation 20 dB. c) Resonance Raman spectrum of **3Cl** in 1:16 MeOH:MeCN at 233 K using λ_{exc} = 473 nm. *Indicate peaks from the solvent. *Conditions to generate **3Cl**:* 0.125 mM **2** in 1:16 MeOH:MeCN + 1 equiv of CAN (in MeCN) + 3 equiv of TBACl (in MeCN) at 233 K. d) Resonance Raman spectrum of **3Cl** in 1:16 MeOH:CD_3CN at 233 K using λ_{exc} = 473 nm. *Indicate peaks from the solvent. *Conditions to generate **3Cl**:* 0.125 mM **2** in 1:16 MeOH: CD_3CN + 1 equiv of CAN (in MeCN) + 3 equiv of TBACl (in MeCN) at 233 K.

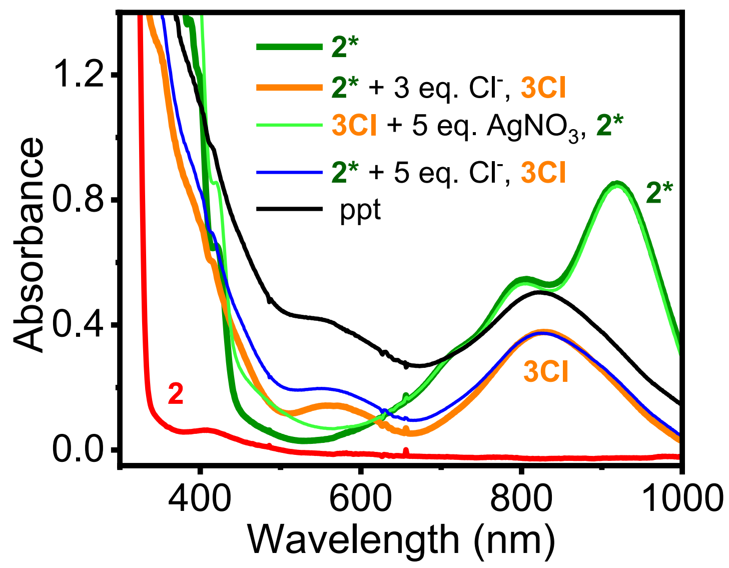

Figure S14. a) UV/vis absorption spectra observed on adding 5 equiv of $AgNO_3$ to the 830 nm species (**3Cl**) that gives 920 nm, **2***. To the regenerated 920 nm, 5 equiv of TBACl is added to form the **3Cl** again which regenerates **2***, on addition of 5 equiv of $AgNO_3$ after which precipitate appears.

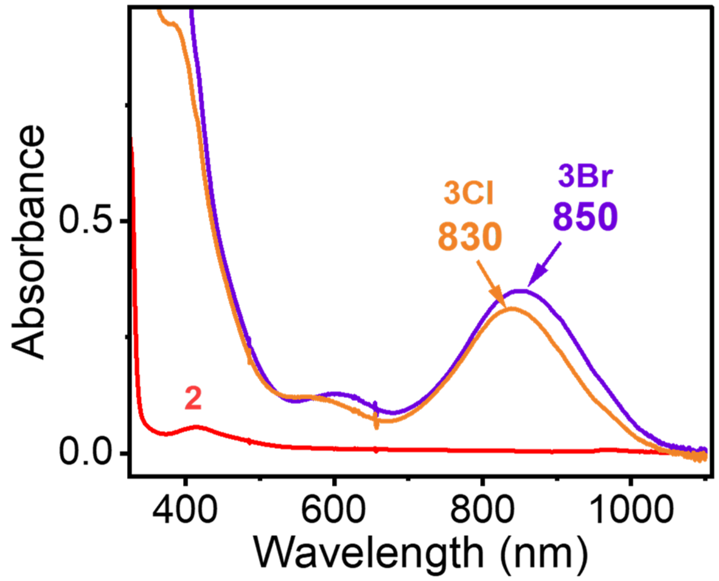

Figure S15. UV/vis absorption spectra observed on adding 3 equiv of TBACl to the one equiv absorbing species, **3Br** at 850 nm, which shows a blue shift and forms 830 nm (**3Cl**). *Condition to generate* **3Br**: 0.25 mM **2** in 1:16 MeOH:MeCN + 4 equiv of TBABr + 1 equiv CAN in CH_3CN at 233 K.

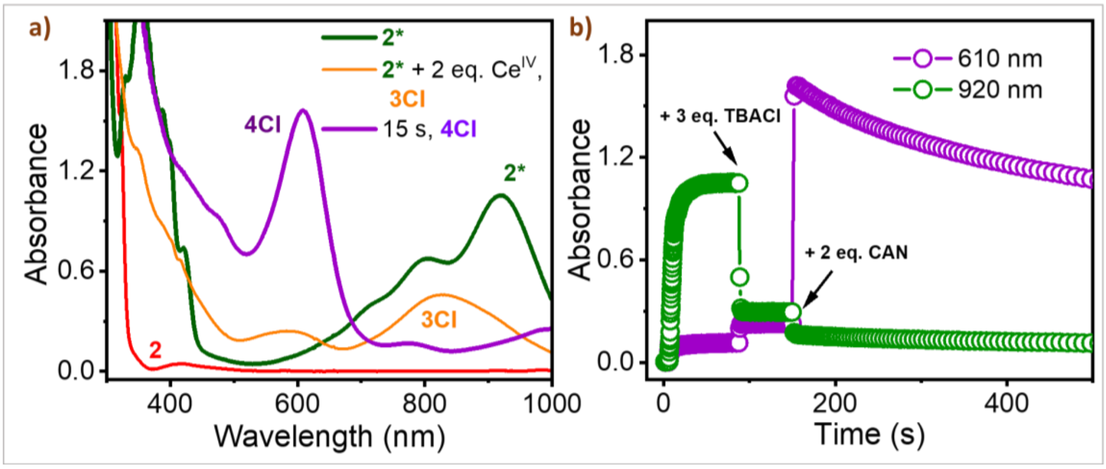

Figure S16. a) UV/vis absorption spectra observed on adding 3 equiv of TBACl to **2*** to form **3Cl** which on addition of 2 equiv of CAN form 610 nm in 1:16 MeOH:MeCN at 233 K. b) The corresponding absorbance changes observed at 610 nm and 920 nm.

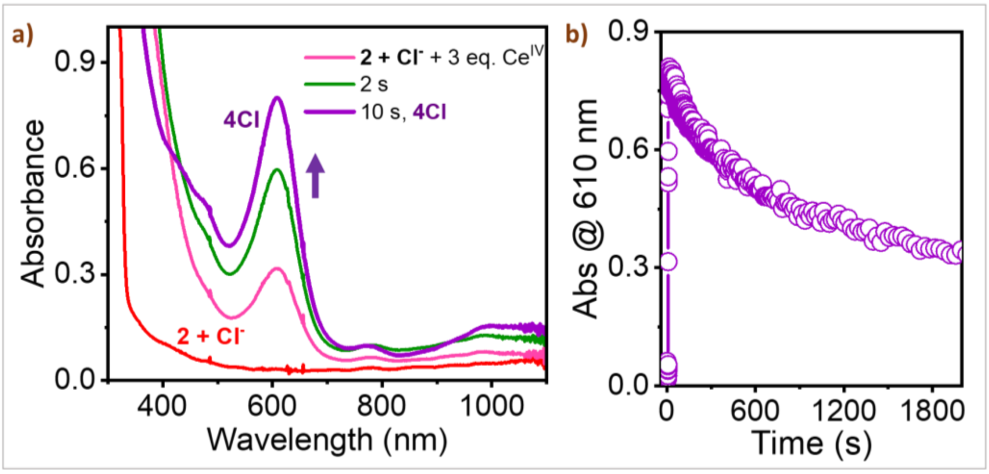

Figure S17. a) UV/vis absorption spectra observed on directly adding 3 equiv of CAN to the mixture of 0.125 mM **2** + 3 equiv of TBACl in 1:16 MeOH:MeCN at 233 K. b) The corresponding absorbance changes at 610 nm.

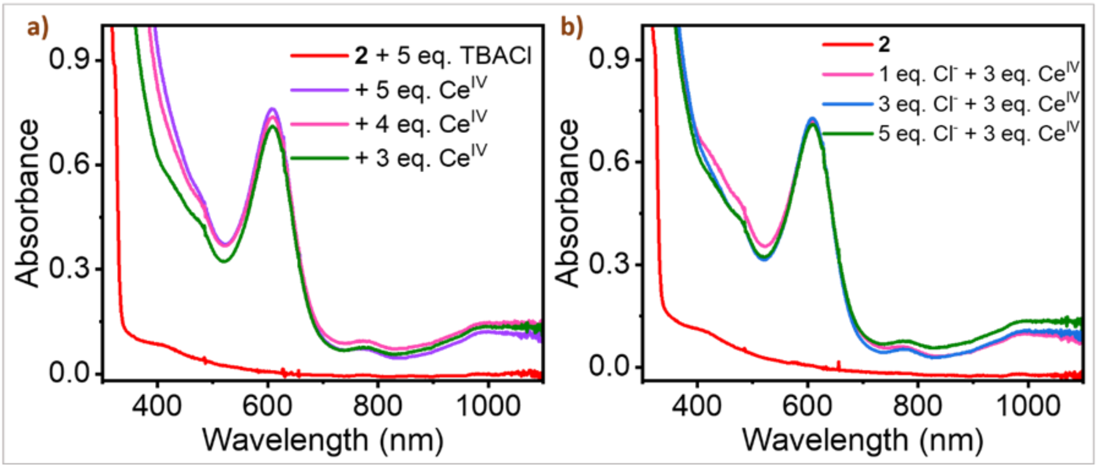

Figure S18. a) UV/vis absorption spectral changes at 610 nm from 0.125 mM of **2** a) by keeping equivalents of TBACl fixed and adding various equivalents of CAN (as mentioned in the legends); b) by keeping equivalents of CAN fixed and varying amount of TBACl (as mentioned in the legends) in 1:16 MeOH:MeCN at 233 K.

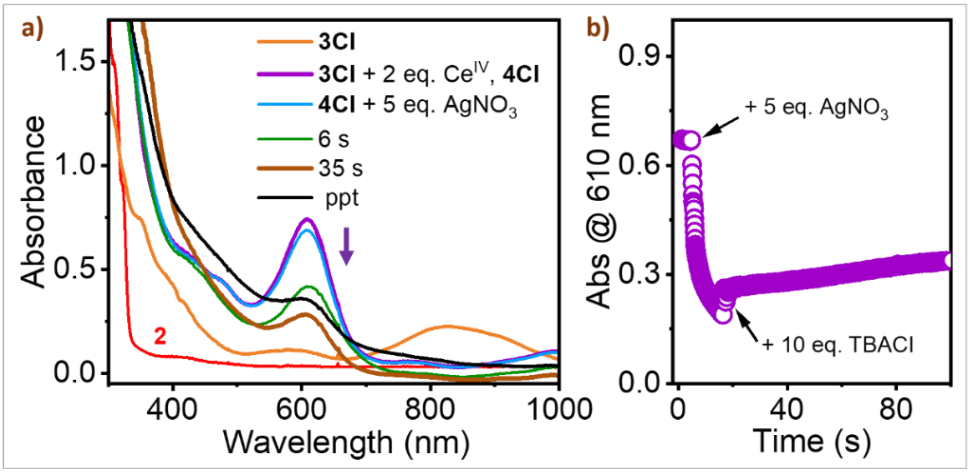

Figure S19. a) UV/vis absorption spectral changes in **4Cl** on addition of 5 equiv of $AgNO_3$ that leads to the decay in its absorbance. This is followed by the addition of 10 equiv of TBACl, which doesn't regenerate the **4Cl** as depicted in the corresponding time trace (b). *Conditions to generate* ***4Cl:*** 0.125 mM **2** + 3 equiv of Cl^- + 3 equiv of CAN in 1:16 MeOH: MeCN at 233 K.

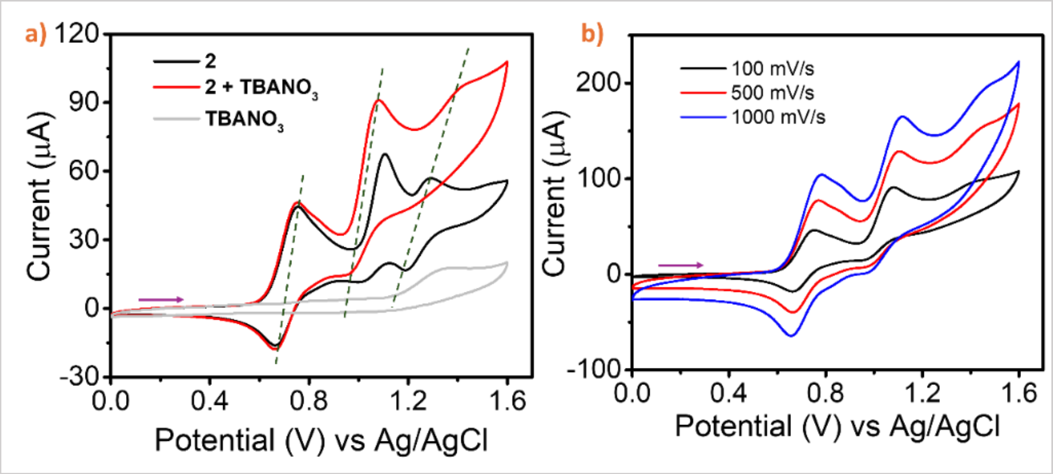

Figure S20. a) Cyclic voltammograms of 2 mM **2** (black), **2** + 3 equiv of $TBANO_3$ (red) and 6 mM $TBANO_3$ (grey) at 100 mV s^{-1} scan rate in 1:16 MeOH:MeCN at 298 K. b) Cyclic voltammograms of **2** in the presence of 3 equiv of $TBANO_3$ in 1:16 MeOH: CH_3CN at 100 mV/s (black), 500 mV/s (red) and 1000 mV/s (blue) scan rates at 298 K.

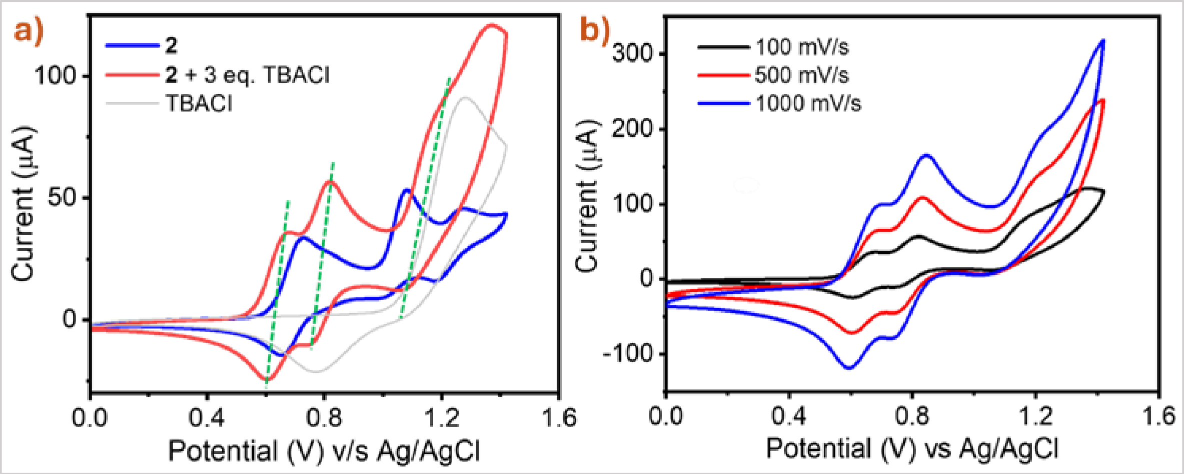

Figure S21. a) Cyclic voltammogram of 1 mM **2** (blue), 3 mM TBACl (grey), and 1 mM **2** in the presence of 3 mM TBACl (red) at 100 mV s^{-1} scan rate in 1:16 MeOH:MeCN at 298 K. b) Cyclic voltammograms of 1 mM **2** in the presence of 3 mM TBACl in 1:16 MeOH:MeCN at 100 mV/s (black), 500 mV/s (red) and 1000 mV/s (blue) scan rates at 298 K.

Table S4. Potential values vs. Ag/AgCl measured for **2** in the presence and absence of 3 equiv of TBACl in 1:16 MeOH:MeCN at 298 K.

	V (mV) vs Ag/AgCl For **2**	V (mV) vs Ag/AgCl For **2** + 3 equiv of Cl^-
$E^1_{p,a}$	0.73	0.67
$E^1_{p,c}$	0.65	0.60
$E^2_{p,a}$	1.08	0.81
$E^2_{p,c}$	0.99	0.74
$E^3_{p,a}$	1.27	1.16
$E^3_{p,c}$	1.18	1.07

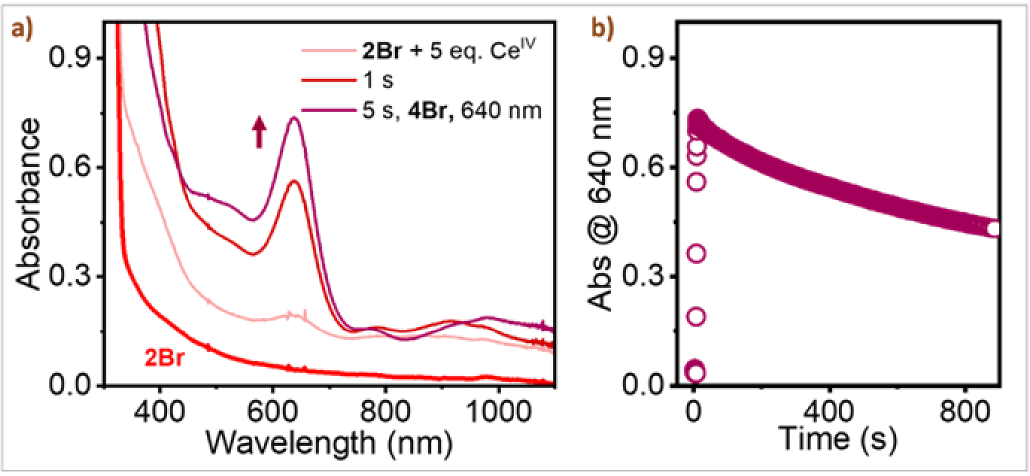

Figure S22. a) UV/vis absorption spectra observed on directly adding 5 equiv of CAN to a mixture of 0.125 mM **2** + 4 equiv of Br^-, **2Br** in 1:16 MeOH:MeCN at 233 K. b) The corresponding absorbance changes observed at 640 nm with respect to time.

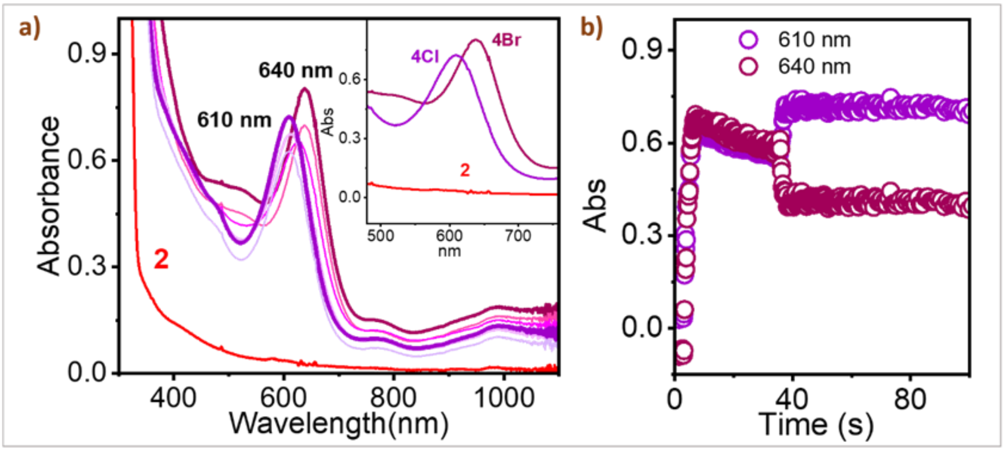

Figure S23. UV/vis absorption spectra observed on adding 3 equiv of TBACl to the 640 nm species (**4Br**), resulting in the generation of a 610 nm absorption band due to **4Cl**. b) The corresponding absorbance changes observed at 640 nm with respect to time. *Condition to generate **4Br***: 0.125 mM **2** + 4 equiv of TBABr + 5 equiv of CAN in 1:16 MeOH:MeCN at 233 K.

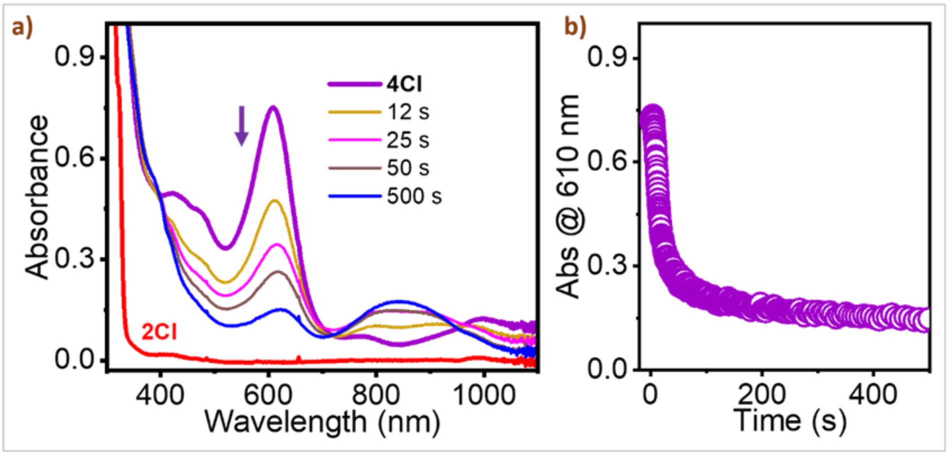

Figure S24. UV/vis absorption spectral changes at 610 nm (due to **4Cl**) on the addition of 4 equiv of TBABr, which leads to the decay in its absorbance. *Condition to generate **4Cl***: 0.125 mM **2** + 3 equiv of TBACl + 3 equiv of CAN in 1:16 MeOH:MeCN at 233 K.

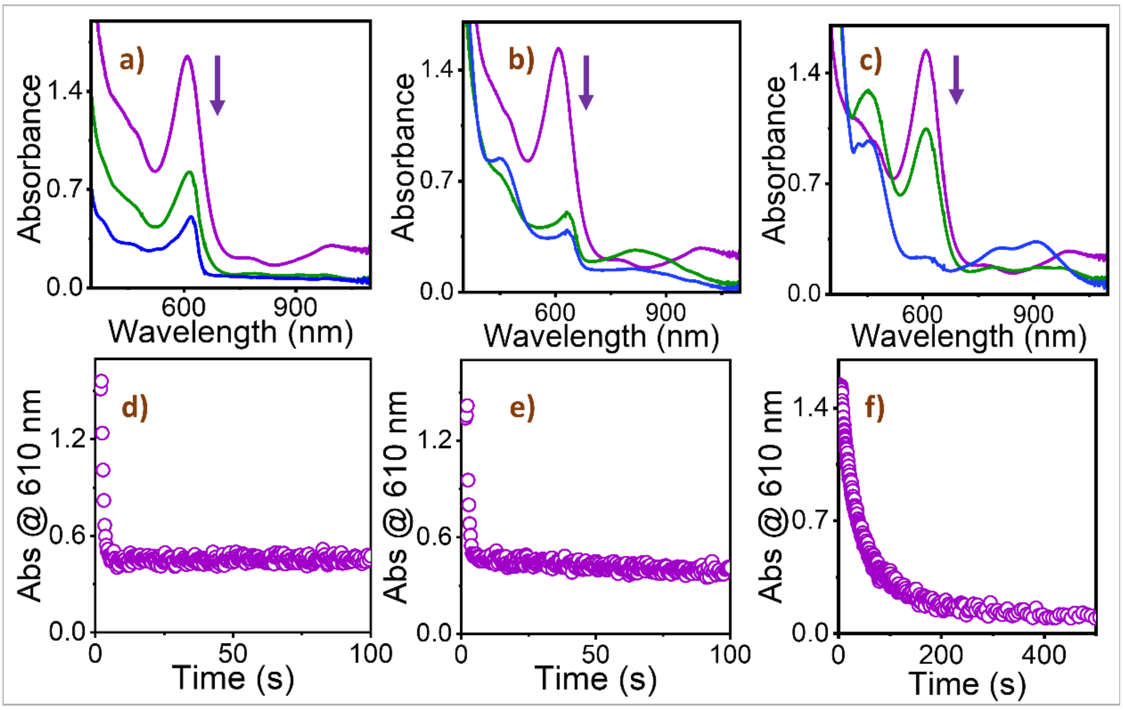

Figure S25. UV/vis absorption spectral changes depicting the reaction of **4Cl** with a) Ferrocene (Fc), b) Acetyl ferrocene (AcFc), and c) Diacetyl ferrocene (Ac_2Fc). Decay in the absorbance at 610 nm for **4Cl** in the presence of d) Ferrocene (Fc), e) Acetyl ferrocene (AcFc) and f) Diacetyl ferrocene (Ac_2Fc). *Conditions to generate **4Cl**:* 0.25 mM **2** + 3 equiv of TBACl + 3 equiv of CAN in 1:16 MeOH:MeCN at 233 K.

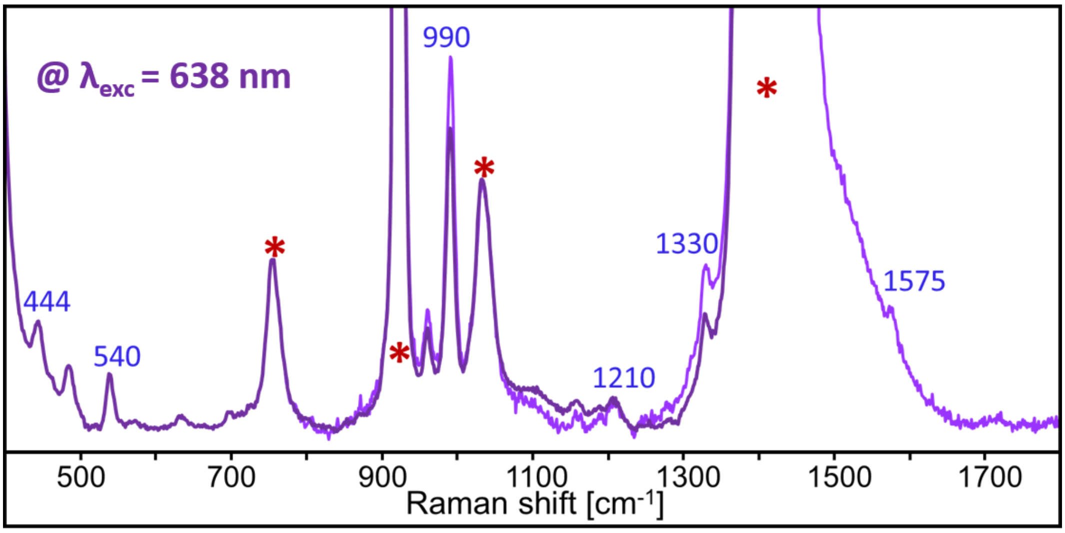

Figure S26. Full-scale resonance Raman spectrum of two electron oxidized species 610 nm species (**4Cl**) at λ_{exc} = 638 nm in 1:16 MeOH:MeCN at 233 K. *Conditions to generate **4Cl***: 0.25 mM **2** in 1:16 MeOH: MeCN + 3 equiv of TBACl (30 µl, 50 mM) + 3 equiv of CAN (30 µl, 50 mM) in MeCN at 233 K.

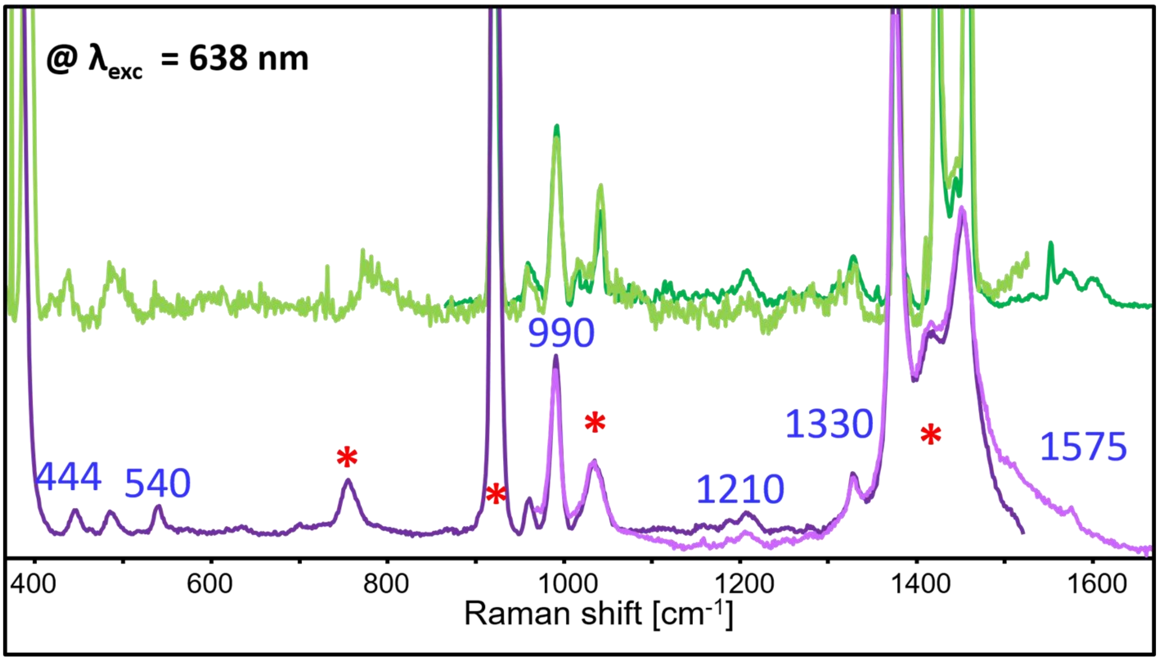

Figure S27. Resonance Raman spectra of species **4Cl** at λ_{exc} 638 nm (green at 77 K) and (purple at 233 K). Condition to generate **4Cl** at 77 K: 2 mM **2** in 1:16 MeOH:MeCN + 3 equiv of TBACl + 3 equiv of CAN in MeCN. Conditions to generate **4Cl** at 233 K: 0.25 mM **2** in 1:16 MeOH:MeCN + 3 equiv of TBACl + 3 equiv of CAN in MeCN. Bands marked with * arise from acetonitrile.

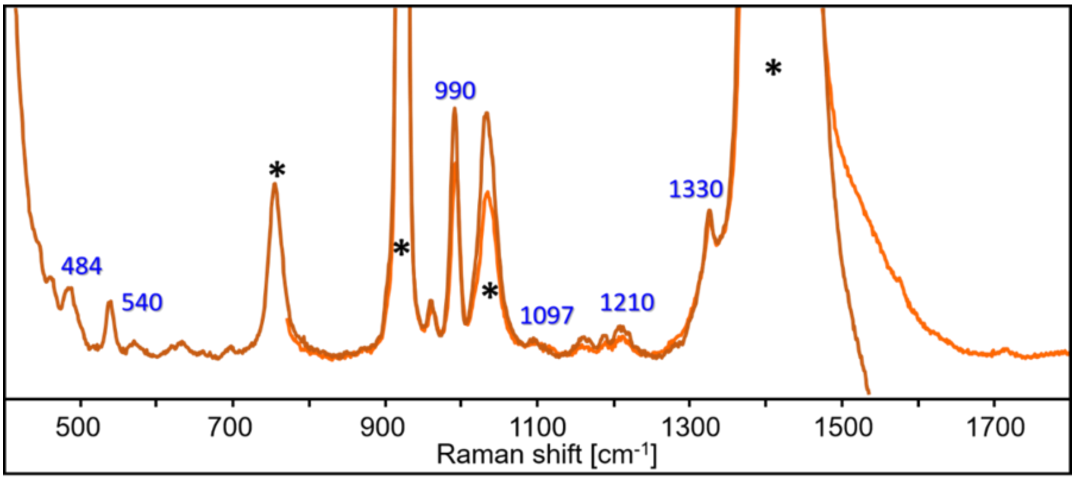

Figure S28. Full-scale resonance Raman spectrum of two electron oxidized species 640 nm species (**4Br**) at λ_{exc} = 638 nm in 1:16 MeOH:MeCN at 233 K. *Conditions to generate **4Br***: 0.25 mM **2** in 1:16 MeOH: MeCN + 4 equiv of TBABr (40 µl, 50 mM) + 5 equiv of CAN (50 µl, 50 mM) in MeCN at 233 K.

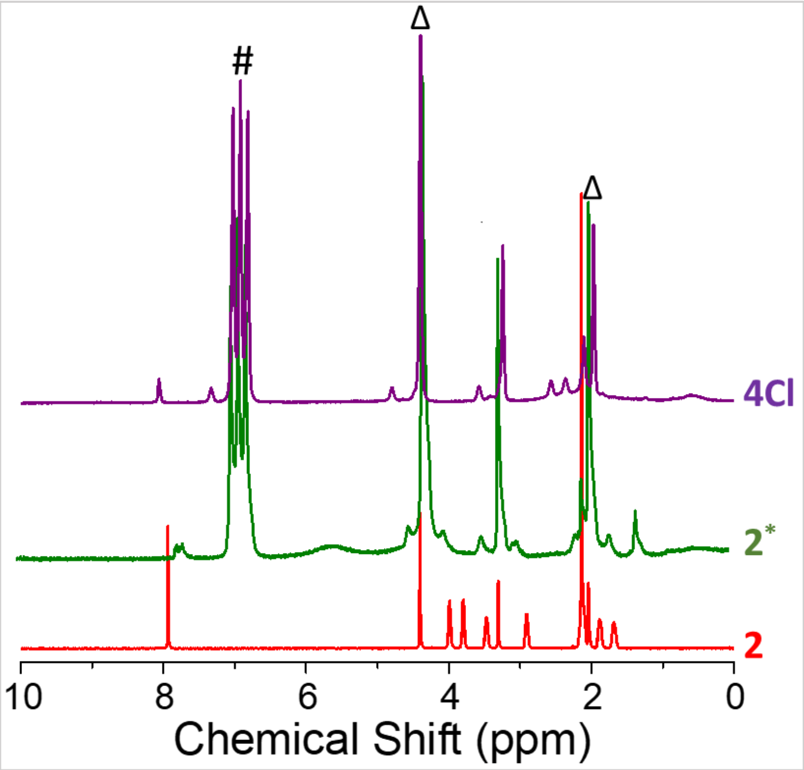

Figure S29. ^{1}H NMR (500 MHz) spectra of species **2**, **2***, and **4Cl** in 1:2.4 CD_3OD:CD_3CN at 228 K. *Conditions to generate* **4Cl**: 10 mM **2** in 1:2.4 CD_3OD:CD_3CN) + 3 equiv of NaCl (in CD_3OD) + 3 equiv of CAN (CD_3CN) at 228 K. *Conditions to generate* **2***: 10 mM **2** in 1:2.4 CD_3OD:CD_3CN) + 1 equiv of CAN (CD_3CN) at 228 K. (#) triplet signal due to NH_4^+ ion derived from CAN and (Δ) signals from CD_3CN and CD_3OD.

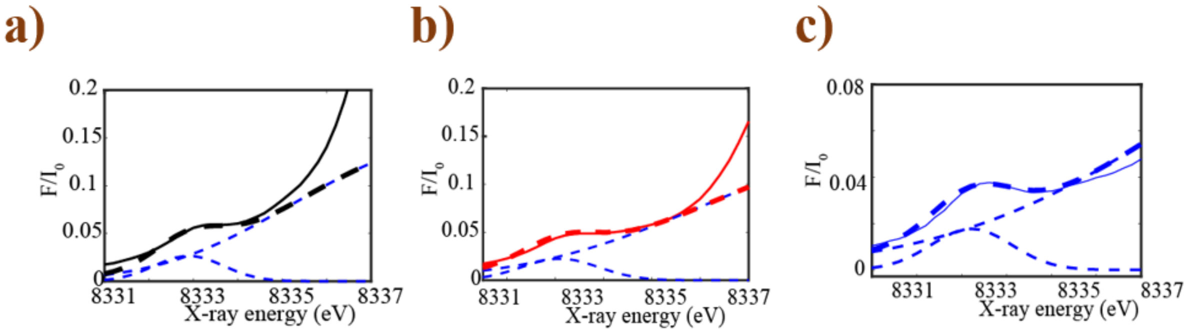

Figure S30. A zoom-in of the pre-edge regions of a) **2**, b) **3**, and c) **4Cl** with the respective fits shown in cyan and orange dashed lines, respectively. The blue lines correspond to the step and Gaussian functions used to fit the pre-edge peak as elaborated in Table S6.

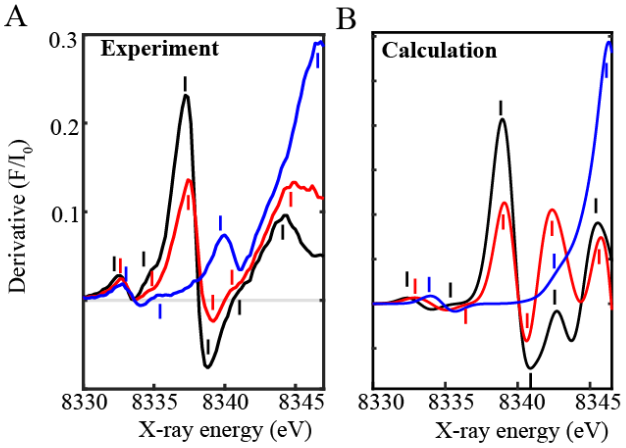

Figure S31 A. Derivative of the experimental pre-edge and rising edge regions within a broader energy range. **B.** TD-DFT simulated XANES derivative spectra corresponding to the formation of a square planar **2** (in black), square pyramidal **3** (in red), and an octahedral **4Cl** (in blue)

Table S5. Comparisons between the experimental features observed from the pre-edge to rising edge regions of **2**, **3** and **4Cl** with TD-DFT XANES simulations

**Complex**	**Experimental Features from pre-edge to rising edge regions in comparison with TD-DFT XANES simulations**	
	Experiment	Theory
**2**	8332.40	8332.42
	8334.59	8335.22
	8337.21	8338.88
	8338.80	8340.80
	8341.00	8342.62
	8344.00	8345.48
**3**	8332.79	8332.98
	8334.80	8336.14
	8337.40	8339.00
	8339.20	8340.64
	8340.60	8342.38
	8344.99	8345.74
**4Cl**	8333.00	8333.94
	8335.41	8337.30
	8340.00	8342.36
	8346.61	8346.20

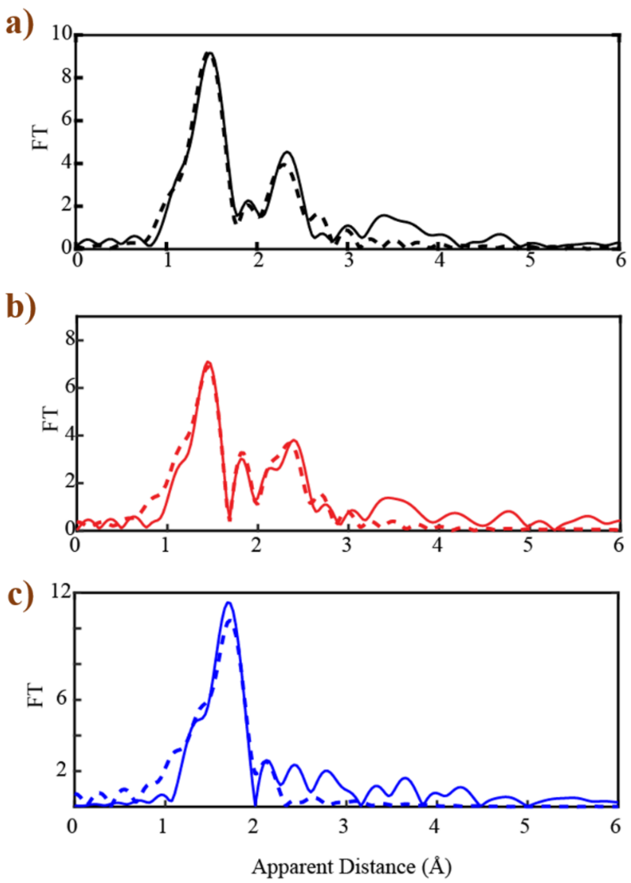

Figure S32. Fourier transforms of k^3-weighted Ni EXAFS of **2** (black), **3** (red), and **4Cl** (blue) in solid lines together with their respective fits 2, 5, and 7 in Table S5 shown in dashed lines.

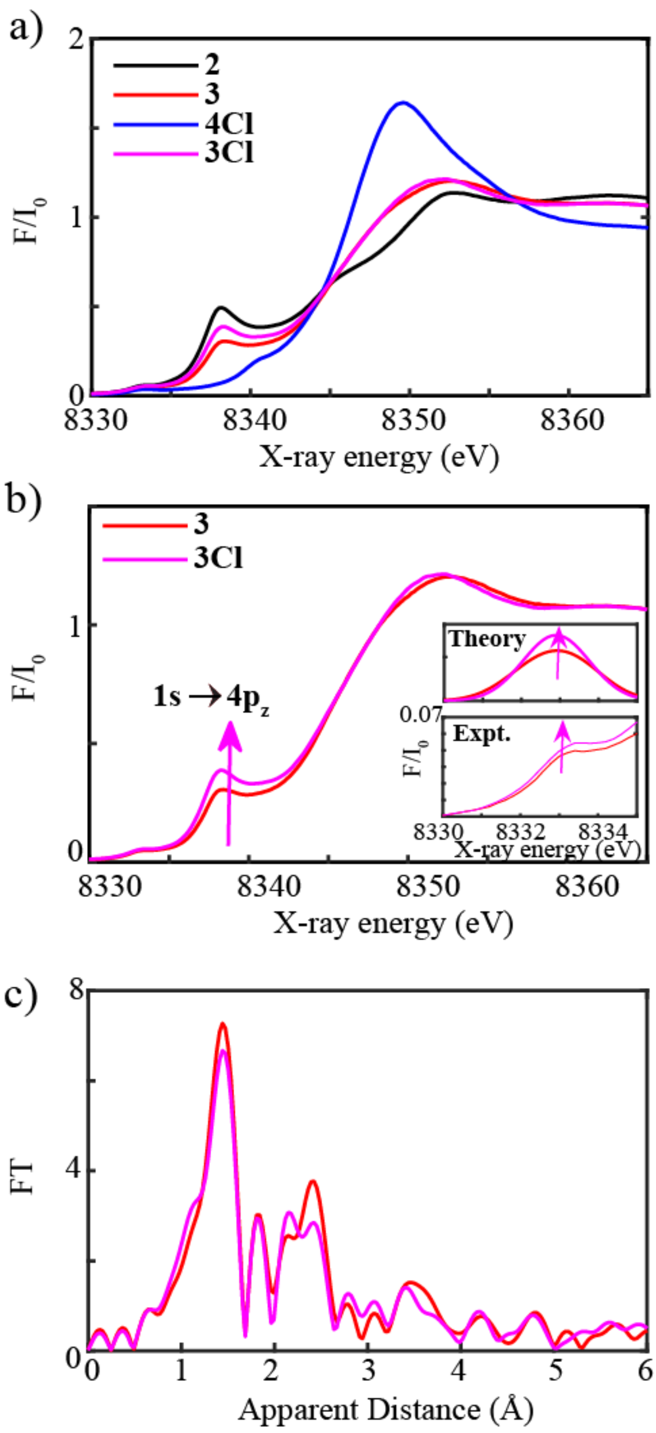

Figure S33. a) Experimental normalized Ni K-edge XANES of **2** (black), **3** (red), **3Cl** (magenta), and **4Cl** (blue) b). Experimental normalized Ni K-edge XANES of **3** (red) and **3Cl** (magenta). Inset. Top. Zoom-in of the Calculated and Bottom. Experimental pre-edge regions c). Fourier transforms of k^3-weighted Ni EXAFS of **3**, and **3Cl**. Experimental spectra were calculated for *k* values of 2−14.15 $Å^{-1}$.

Table S6. Pre-edge parameter fits for **2**, **3**, and **4Cl**.

**2**			
**Function**	**Centroid (eV)**	**Amplitude**	**Width**
**Erf**	8335.20	0.080	3.40
**Gauss**	8332.90	0.052	0.78
**Pre-edge area**	5.2 units		
**3**			
**Function**	**Centroid (eV)**	**Amplitude**	**Width**
**Erf**	8336.17	0.080	4.30
**Gauss**	8333.10	0.045	0.800
**Pre-edge area**	4.5 units		
**[$LNi^{IV}Cl_2$]** (**4Cl**)			
**Function**	**Centroid (eV)**	**Amplitude**	**Width**
**Erf**	8338.99	0.080	6.80
**Gauss**	8333.22	0.040	0.900
**Pre-edge area**	4.0 units		

Table S7. EXAFS Fits parameters.

**Sample**	**Fit**	**Region**	**Shell,N**	**R, Å**	**E_0**	**ss.^{2} (10^{-3})**	**R-factor**	**Reduced Chi-square**
**2**	1	I	Ni-N,4	1.87	2.3	4.6	0.0009	115
	**2**	**I,II**	**Ni-N,4**<br>**Ni-C,4**	**1.87**<br>**2.76**	**4.3**	**4.6**<br>**2.8**	**0.0005**	**31**
**3**	3	I	Ni-N,4	1.84	-8.2	6.3	0.0043	192
	4	I,II	Ni-N,4<br>Ni-N,1	1.84<br>2.42	-4.1	6.7<br>3.5	0.0064	1056
	**5**	**I,II**	**Ni-N,4**<br>**Ni-N,1**<br>**Ni-C,4**	**1.86**<br>**2.54**<br>**2.75**	**0.72**	**7.2**<br>**2.5**<br>**3.8**	**0.0083**	**189**
**[$LNi^{IV}Cl_2$]** (**4Cl**)	6	I	Ni-N,4<br>Ni-Cl,2	1.85<br>2.15	-12	3.9	0.0034	2399
	7	I,II	Ni-N/O,5<br>Ni-Cl,1<br>Ni-C,4	1.88<br>2.14<br>2.64	-11	21<br>0.8<br>10	0.008	2214

	**8**	**I,II**	**Ni-N,4**<br>**Ni-Cl,2**<br>**Ni-C,4**	**1.87**<br>**2.15**<br>**2.64**	**-10**	**18**<br>**4.0**<br>**12.3**	**0.0022**	**435**
**[LNi^{III}Cl$_2$]$^-$**<br>(**3Cl**)	9	I	Ni-N, 4<br>Ni-Cl, 1	1.86<br>2.55	2.6	8.6<br>14	0.0401	1319
	10	I,II	Ni-N,4<br>Ni-Cl,2<br>Ni-C,4	1.86<br>2.54<br>2.64	4.3	9.9<br>13<br>9.7	0.034	723
	**11**	**I,II**	**Ni-N,4**<br>**Ni-Cl,1**<br>**Ni-C,4**	**1.86**<br>**2.54**<br>**2.67**	**2.8**	**9.3**<br>**10**<br>**11**	**0.0207**	**436**

* The amplitude reduction factor S_0^2 was fixed to 1. Region I refers to the EXAFS spectra region between 1.1-2.0/2.1 Å Regions I, II refer to that between 1.1-3 Å. We note that the data resolution, the ability to distinguish between 2 bond distances, given by π/2Δk is ~ 0.129 Å.

Table S8. Summary of the bond distances of calculated Ni-based complexes **2**, **3**, **3Cl**, and **4Cl** bond distances are in Å.

**Complexes**	**Ni-N$_1$**	**Ni-N$_2$**	**Ni-N$_3$**	**Ni-N$_4$**	**Ni-CH$_3$CN(1)**	**Ni-Cl$_1$**	**Ni-Cl$_2$**	**Ni-N (avg.)**	**Ni-Cl (avg.)**
**2**	1.84913	1.84296	1.97315	1.96725				1.90812	
**3**	1.84501	1.84501	1.94443	1.94928	2.39014			1.89590	
**[LNi^{IV}Cl$_2$] (4Cl)**	1.86880	1.87206	1.98794	1.98971		2.25237	2.45079	1.92962	2.35158
**[LNi^{III}Cl] (3Cl)**	1.84228	1.85041	1.95197	1.97270		2.43568		1.90434	
**[LNi^{III}Cl$_2$]**	1.86226	1.86560	1.97020	1.97381		2.55237	2.58960	1.91797	

Computational methods:

All geometry optimizations and frequency computations were performed in Gaussian16, with M06L as the functional using the unrestricted formalism and def2-TZVP as the basis set. Single points were computed using M06L functional and def2-TZVPP basis set. Solvent effects were accounted for using integral equation formalism of polarized continuum model as implemented in Gaussian 16. Acetonitrile was used as the solvent for all computations. Ultrafine grid was used for all computations. Stability analysis was performed to verify the stability of the wave function. XQC and YQC algorithms were used when necessary for scf convergence for both geometry optimizations and single points. TDDFT computations were performed using Orca 5.0.4. Unrestricted formalism was used for TDDFT computations. PBE0 functional with ZORA-def2-TZVP basis set and CPCM solvent model with acetonitrile as the solvent were used for TDDFT computations.

Key words used:

For optimizations:

#P M06L def2tzvp opt freq=noraman scrf=(pcm,solvent=acetonitrile)

For stability analysis,

Stable = opt

For single points: #P M06L gfinput IOP(6/7=3) def2tzvpp scrf=(pcm,solvent=acetonitrile) 6D 10F

For TDDFT

! UKS PBE0 ZORA ZORA-DEF2-TZVP SARC/J CPCM(CH3CN) RI-SOMF(1X)

%PAL NPROCS 8 END

%TDDFT NROOTS 10

DoNTO TRUE

NTOSTATES 1,2,3,4,5,6,7,8,9,10

NTOThresh 1e-4

DOSOC FALSE

TDA FALSE

END

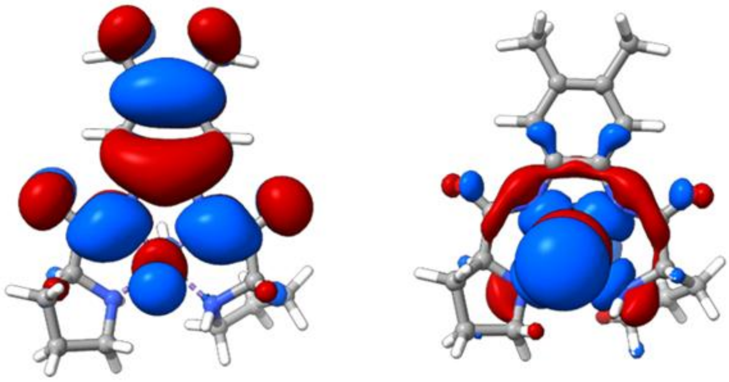

Figure S34. HOMO (left) and LUMO (right) orbitals for complex **3** computed at CPCM (CH_3CN)-uPBE0/ZORA-def2-TZVP//IEFPCM (CH_3CN)-uM06L/def2-TZVP level of theory.

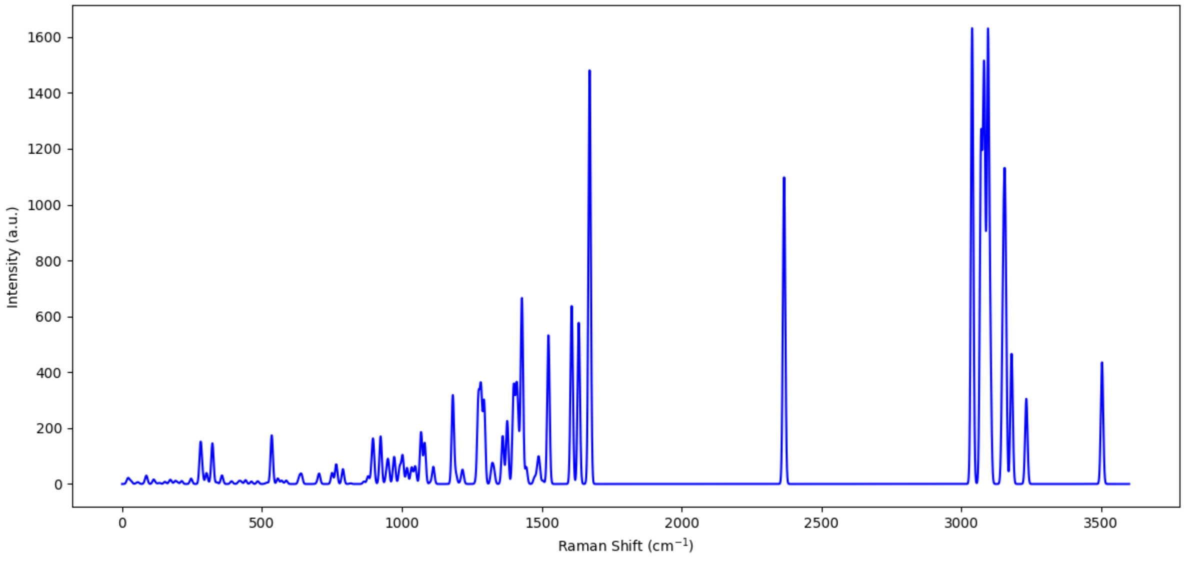

Figure S35. Computed Raman spectrum for **3** coordinated to two acetonitrile molecules at PCM-acetonitrile-UM06L/def2-TZVP level of theory with a Gaussian line broadening of 10 cm^{-1}. The peak at 2500 cm^{-1} corresponds to the stretching of the nitrile group

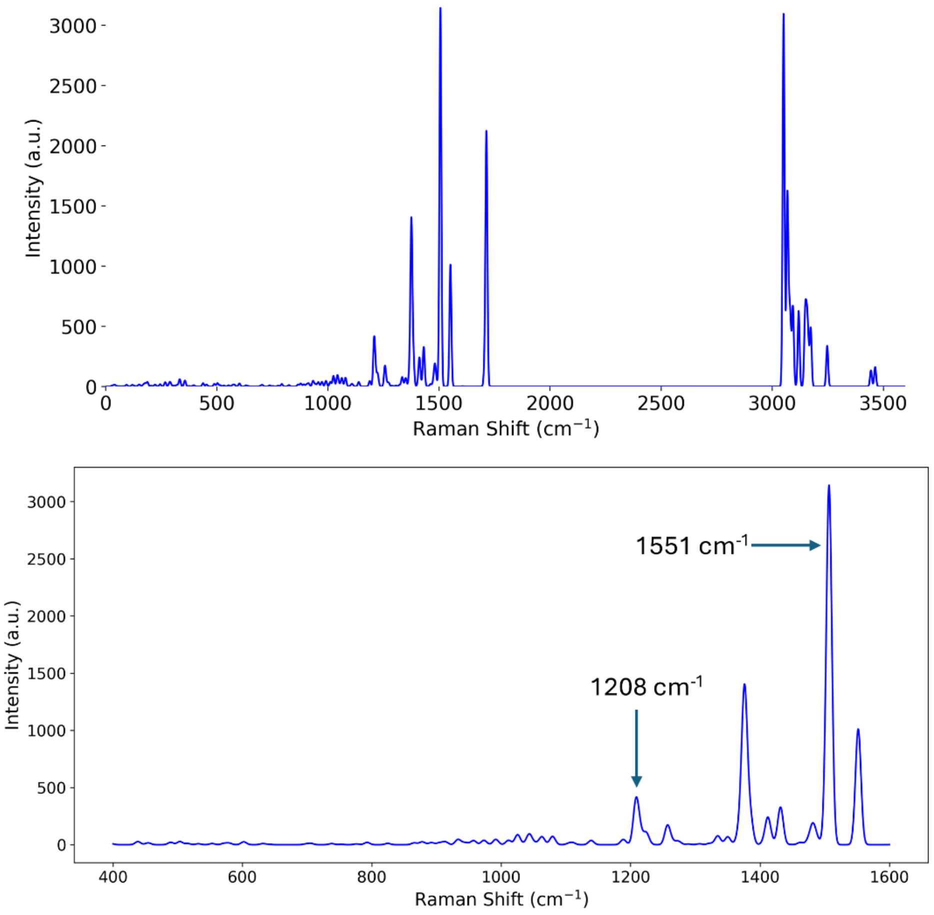

Figure S36. Computed Raman spectrum for complex **4Cl** at PCM-acetonitrile-UM06L/def2-TZVP level of theory with a Gaussian line broadening of 10 cm^{-1}. The lack of a peak at 2500 cm^{-1} indicates the absence of nitrile stretch and thereby a lack of nitrile coordination. The figure also shows the zoomed in view in the range of 400 to 1600 cm^{-1}. In the zoomed in figure, two peaks at 1208 cm^{-1} and 1551 cm^{-1} are highlighted to show the agreement with the experimental data discussed in Figure S27.

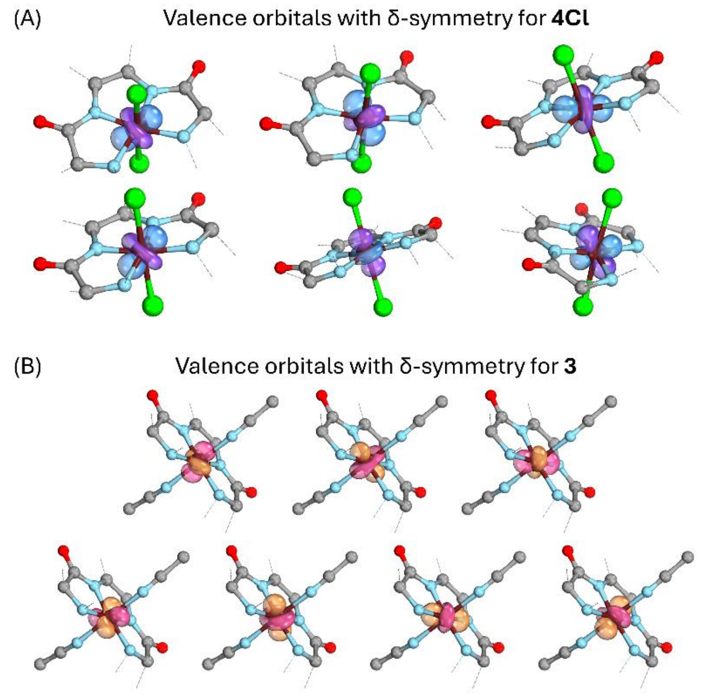

Figure S37. (A) Intrinsic bond localized orbitals for the **4Cl** complex centered around nickel and (B) Intrinsic bond localized orbitals for complex **3** centered around nickel computed at PCM(CH_3CN)-uM06L/def2-TZVPP level of theory.

Oxygen atom reactivity with thioanisole and its derivatives:

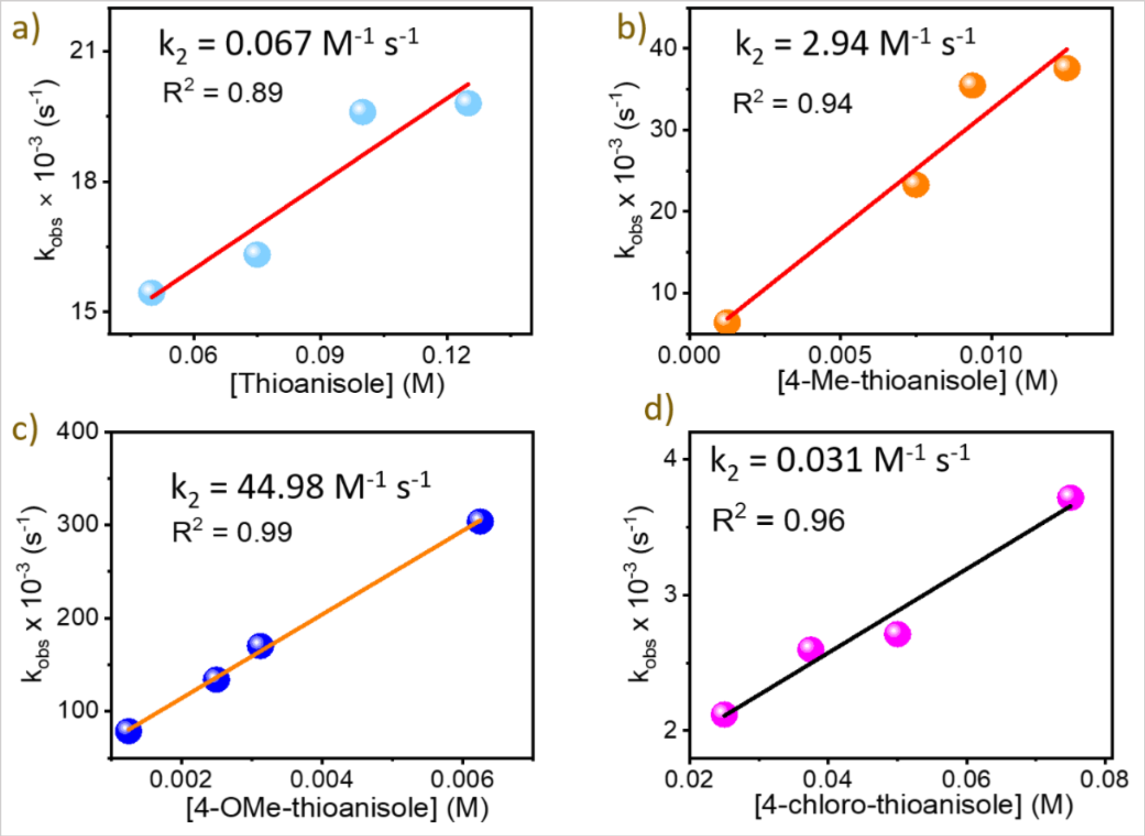

Figure S38. The plot of k_{obs} against the concentration of thioanisole derivatives 4-*X*-thioanisole (a) X =H, (b) X = Me, (c) X= OMe, (d) X= Cl; to get the corresponding k_2 at 233 K for **4Cl**. *Conditions to generate **4Cl**: 0.25 mM **2** + 3 equiv of Cl^- + 3 equiv of CAN in 1:16 MeOH:MeCN at 233 K.*

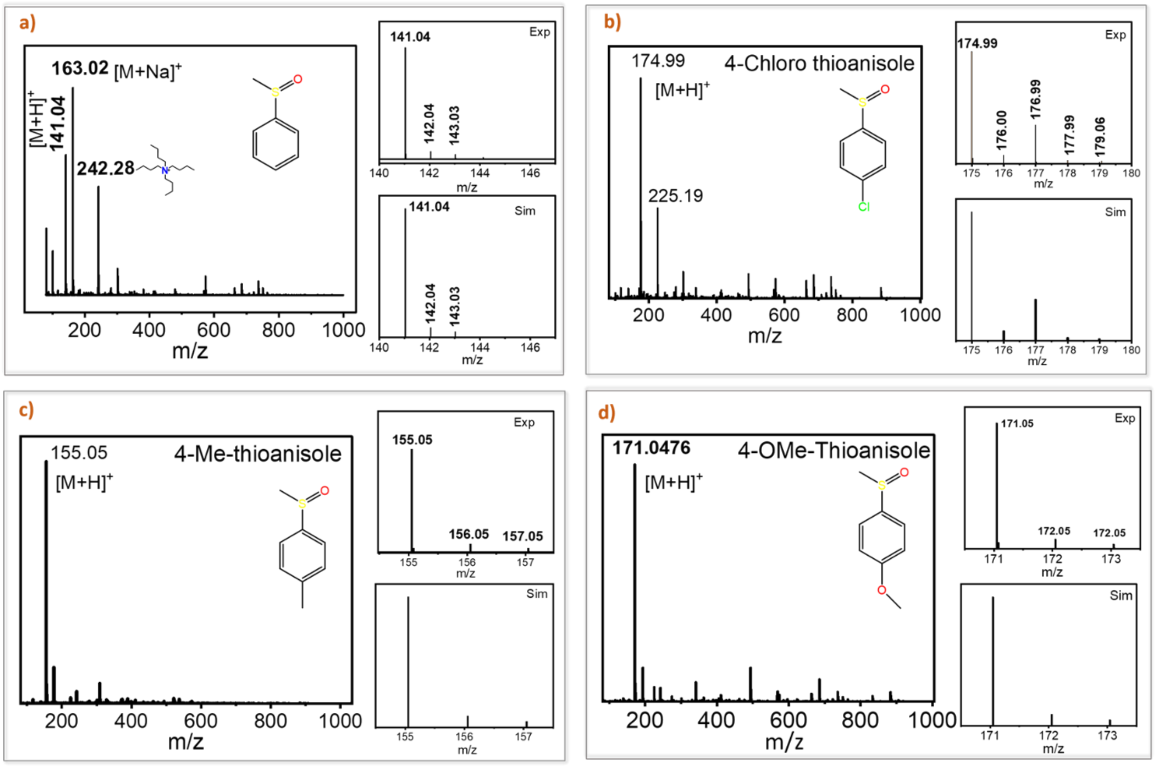

Figure S39. Product analysis by ESI-MS. The reaction of **4Cl** with 40 equiv of (a) thioanisole, (b) 4-*Cl*-thioanisole, (c) 4-Me-thioanisole and (d) 4-OMe-thioanisole. Conditions to generate **4Cl**: 1 mM **2** + 10 equiv of TBACl + 10 equiv of CAN in 1:16 CH_3OH:CH_3CN at 233 K.

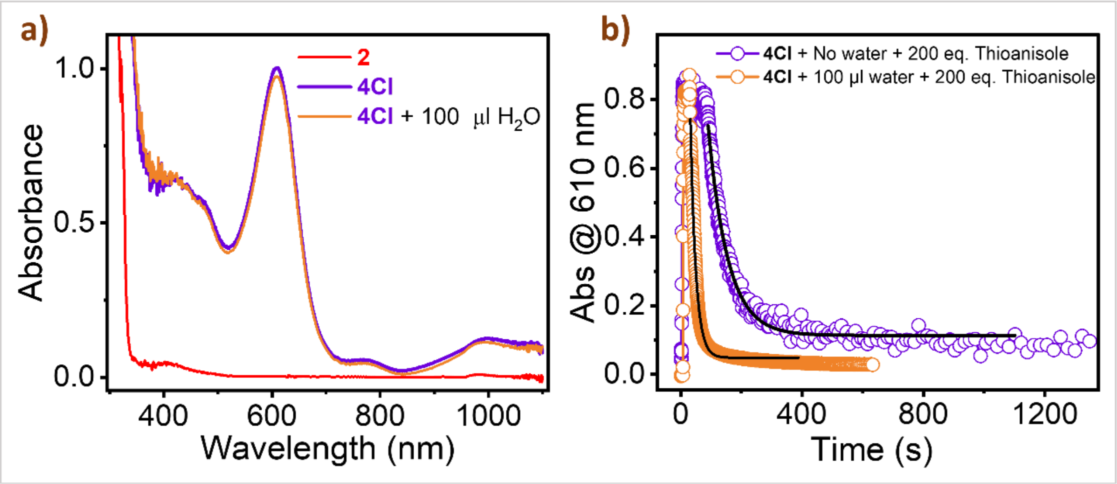

Figure S40. a) UV/vis absorption spectra of **4Cl** (purple) and **4Cl** with 100 µL water (orange) at 233 K, and b) the corresponding absorbance changes observed on the reaction of **4Cl** and 200 equiv thioanisole with (orange) and without (purple) the presence of 100 µL water at 233 K. *Conditions to generate **4Cl***: 0.125 mM **2** in 1:16 MeOH:MeCN + 3 equiv of TBACl + 3 equiv of CAN in CH_3CN at 233 K.

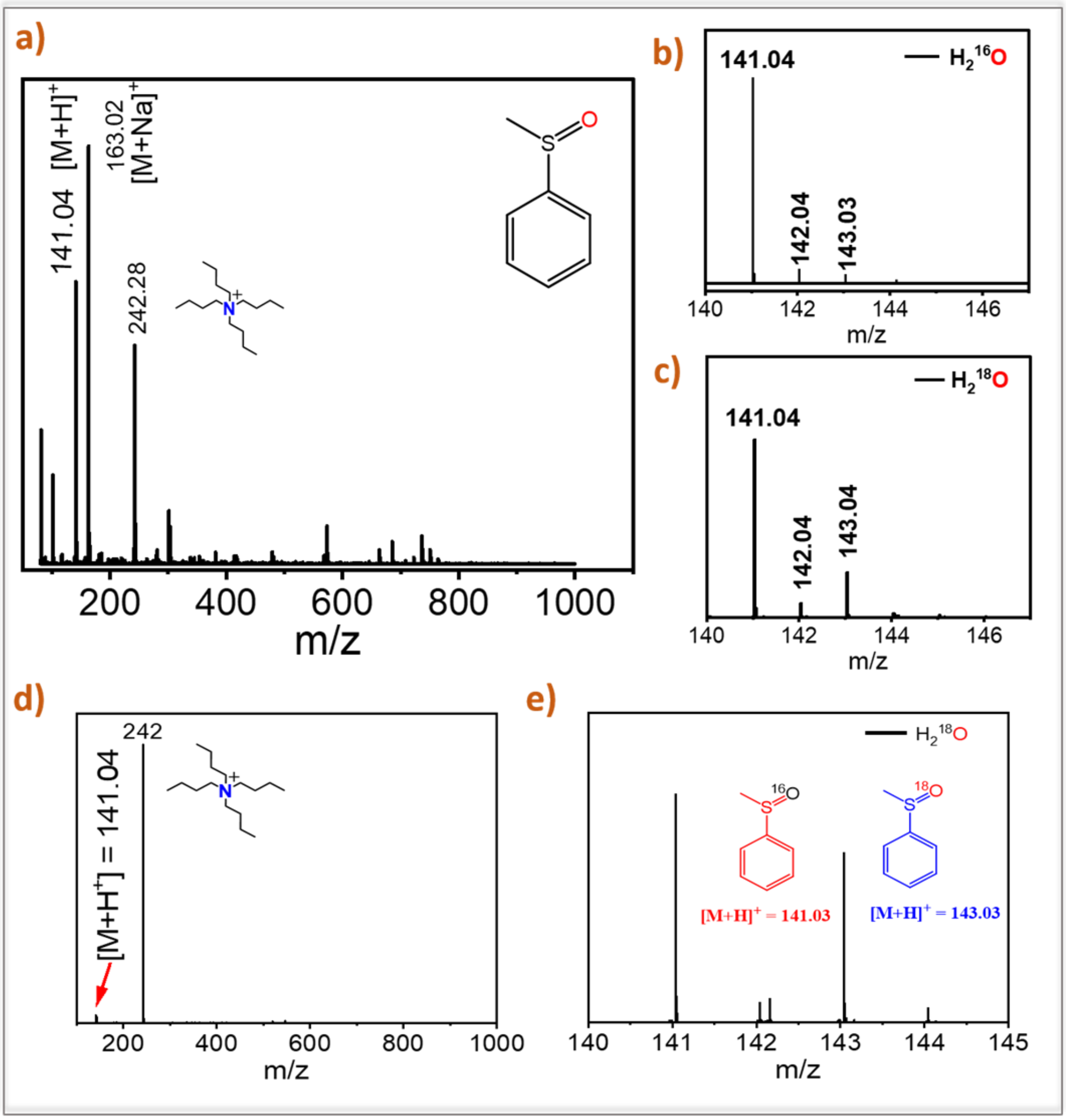

Figure S41. a) Product analysis of the reaction of **4Cl** with 40 equiv of thioanisole in the presence of 10 µl $H_2{}^{18}O$ in 1:16 MeOH:MeCN at 298 K by ESI-MS. Zoom in spectra with the b) peak at 141.04 corresponding to the ^{16}O sulfoxide product formed without the addition of H_2O. c) peak at 143.03 with sulfoxide product formed in the presence of 10 µl $H_2{}^{18}O$. *Conditions to generate* **4Cl**: 1 mM **2** in 1:16 MeOH:MeCN + 10 equiv of TBACl + 10 equiv of CAN in MeCN at 298 K. (d) ESI-MS analysis of the reaction between **4Cl** and 40 equiv of thioanisole in the presence of 100 µL of $H_2{}^{18}O$ in a 1:16 MeOH:MeCN mixture at 298 K. (e) Expanded view of the signal at m/z 141.04.

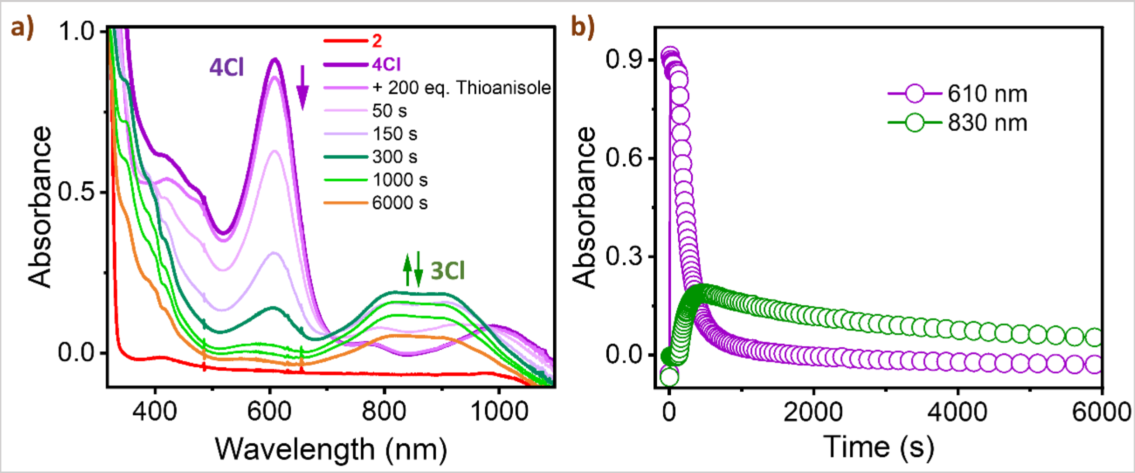

Figure S42. a) UV/vis absorption spectra depicting changes of **4Cl**, on the addition of 200 equiv of thioanisole to form **3Cl**, and b) the corresponding absorption changes at 610 nm (for **4Cl**) and 830 nm (for **3Cl**). *Conditions to generate* **4Cl**: 0.125 mM **2** in 1:16 MeOH:MeCN + 3 equiv of TBACl + 3 equiv of CAN in MeCN at 233 K.

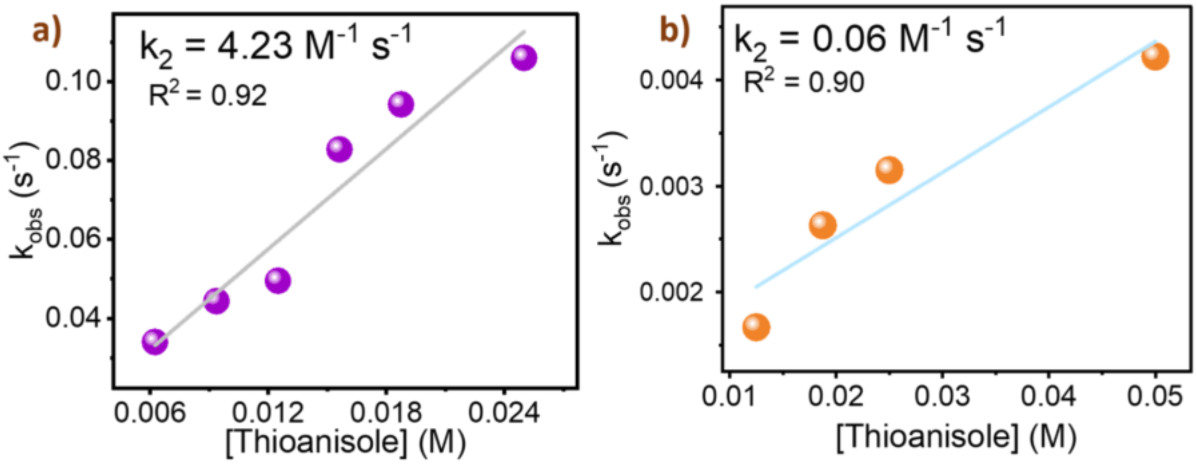

Figure S43. a) The plot of k_{obs} against the concentration of thioanisole for species (a) **4Cl**, (b) **3Cl**; to get the corresponding k_2 at 273 K. *Conditions to generate* **4Cl**: 0.125 mM **2** in 1:16 MeOH:MeCN + 3 equiv of TBACl + 3 equiv of CAN in MeCN at 273 K. *Conditions to generate* **3Cl**: 0.25 mM **2** in 1:16 MeOH:MeCN + 3 equiv of TBACl + 1 equiv of CAN in MeCN at 273 K in MeCN at 273 K.

Free energy barrier for electron transfer:

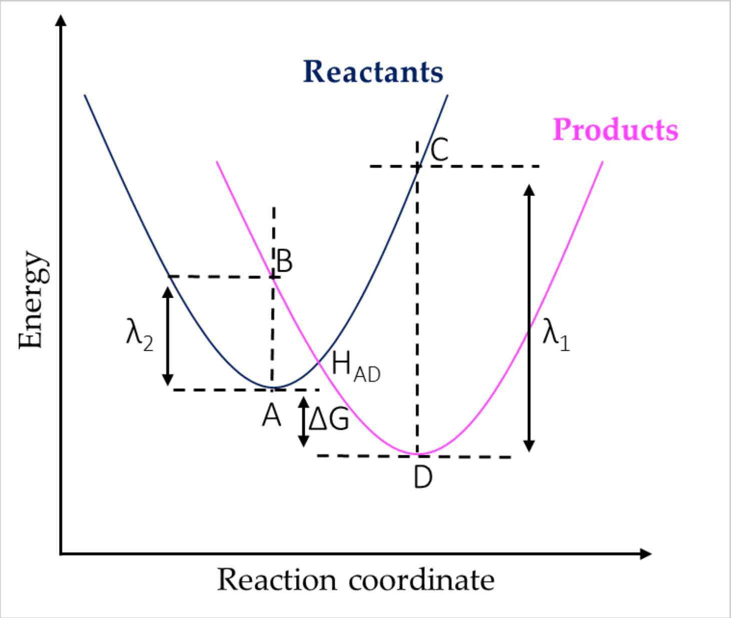

Figure S44. Marcus theory approach for computing the SET barrier.

Marcus theory approach was used for computing the electron transfer free energy barrier by Equation S1. In a traditional Marcus computation, ΔG, the driving force, is computed by taking the free energy difference of points A and D (Figure S44). However, it is important to note that the electron transfer free energies obtained via Marcus theory will have different errors associated with it compared to the free energy barriers obtained via semiclassical transition state theory, owing to the assumptions centered around the reorganizational energy computations.

Equation S1. $\Delta G^{\ddagger}_{ET} = (\Delta G + \lambda)^2/(4\lambda)$

Reorganization energy (λ) was computed in two parts, *viz.*, inner sphere reorganization energy (λ_i) and outer sphere reorganization energy (λ_o). Inner sphere reorganization energy is associated with the molecular rearrangement associated with the electron transfer, while outer sphere reorganization energy is associated with the reorganization of the solvent sphere surrounding the solutes.

Equation S2. $\lambda = \lambda_i + \lambda_0$

Inner sphere reorganization energy (λ_i):

The four-point model was used to estimate the inner sphere reorganization energy for both the nickel complex, **[LNi^{IV}Cl$_2$]**, and the thioanisole derivatives.

$$\lambda_i = (\lambda_1 + \lambda_2)/2$$

Equation S3. Equation used for the computation of inner sphere reorganization energy, where λ_1 and λ_2 were obtained as shown in Figure S44.

Table S9. Table computing the inner sphere reorganization energy.

Molecule	E_R(G_P) (Hartree)	E_R(G_R) (Hartree)	E_P(G_R) (Hartree)	E_P(G_P) (Hartree)	λi (kcal/mol)
**[LNi^{IV}Cl$_2$]**	-3498.9566	-3499.0222	-3499.1613	-3499.2085	35.4
**Thioanisole**	-669.6099	-669.8230	-669.8196	-669.6133	2.1
***p*-OMe Thioanisole**	-784.1801	-784.3774	-784.3719	-784.1855	3.4
***p*-Me Thioanisole**	-708.9421	-709.1489	-709.1446	-708.9462	2.6
***p*-Cl Thioanisole**	-1129.2181	-1129.4309	-1129.4269	-1129.2220	2.5

For example,

- E_R(G_P) is the energy of **[LNi^{IV}Cl$_2$]** in the geometry of **[LNi^{III}Cl$_2$]** (C in Figure S44)
- E_R(G_R) is the energy of **[LNi^{IV}Cl$_2$]** in the geometry of **[LNi^{IV}Cl$_2$]** (A in Figure S44)
- E_P(G_R) is the energy of **[LNi^{III}Cl$_2$]** in the geometry of **[LNi^{IV}Cl$_2$]** (B in Figure S44)
- E_P(G_P) is the energy of **[LNi^{III}Cl$_2$]** in the geometry of **[LNi^{III}Cl$_2$]** (D in Figure S44).

Outer Sphere Reorganization Energy (λ_o):

Outer sphere reorganization energy for both the catalyst and the thioanisole was computed using the modified two sphere model proposed by Liu and coworkers.[3] This model attempts to fix the overestimation of the outer sphere reorganization energy from the original model by incorporating a constant that is dependent on the dielectric constants of the solvent.

$$\lambda_o = \frac{\Delta q^2}{2}\left(\frac{1}{r_D} + \frac{1}{r_A} - \frac{2}{d}\right)\frac{(\varepsilon_{op}^{-1} - \varepsilon_s^{-1})^2}{(1-\varepsilon_s^{-1})}$$

Equation S4. Modified two-sphere model used to compute λ_o. r_D and r_A stand for the radius of the donor and acceptor molecules, and d is the sum of these radii. ε_{op} (1.34 for DCE) stands for the optical dielectric constant of the solvent used, and ε (36.60 for DCE) stands for the dielectric constant. Δq stands for the total number of electrons involved in the SET step, which is 1 in the systems investigated.

Table S10. Cavity volume data for the molecules of interest in $Å^3$ and radius in Å.

Species	Volume ($Å^3$)
**[$LNi^{IV}Cl_2$]**	526.134
**[$LNi^{III}Cl_2$]**	525.785
Thioanisole	184.655
Radical Cation Thioanisole	184.474
p-methyl thioanisole	210.765
Radical Cation p-methyl thioanisole	210.062
p-methoxy thioanisole	223.164
Radical Cation *p*-methoxy thioanisole	222.469
p-chloro thioanisole	207.383
Radical Cation *p*-Chloro thioanisole	206.371

Table S11. Solvent reorganizational energy in kcal/mol.

Species	Solvent reorganizational energy (kcal/mol)	Scaled
Thioanisole	45.2	25.2
p-methyl thioanisole	44.6	24.6
p-methoxy thioanisole	44.4	24.4
p-chloro thioanisole	44.7	24.7

Outer sphere reorganizational energy is typically overestimated by 20 kcal/mol as shown by Hammes-Schiffer and coworkers.[4] So, we have subtracted another 20 kcal/mol from the outer sphere reorganization energy. The barriers obtained for electron transfer computed with this scaled outer sphere reorganization energy were found to be more reasonable compared to the unscaled ones under the experimental conditions, further validating the assumption.

Table S12. The computed electron transfer barriers.

Species	Scaled Electron transfer (kcal/mol)
Thioanisole	12.8
p-methyl thioanisole	11.0
p-methoxy thioanisole	8.6
p-chloro thioanisole	12.6

We have computed the UV/Vis spectra for **4Cl** at the TD-DFT by turning off TDA approximation and increasing the total roots to 30. This resulted in a much better fit with the experimental data for **4Cl** as shown in the figure below.

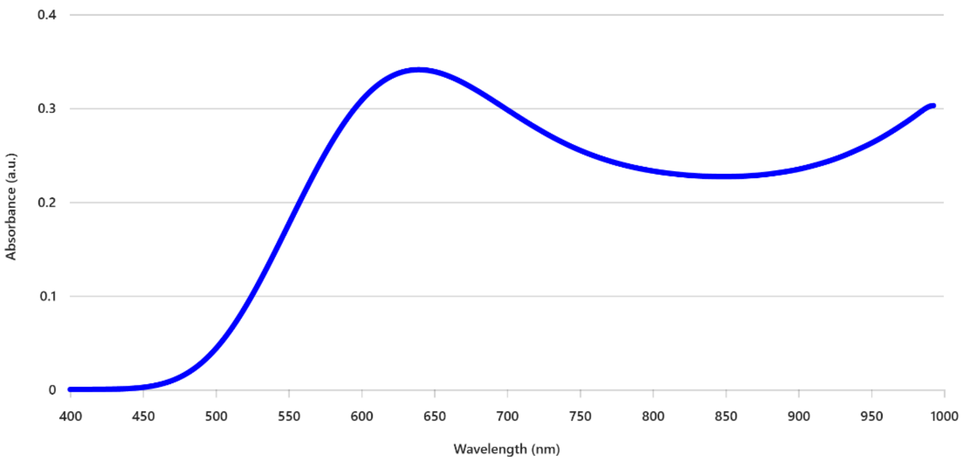

Figure S45. Computed UV/Vis spectrum for **4Cl** with increased total roots and TDA approximation turned off.

The natural transition orbitals are much better at representing the transitions involved in the UV/vis bands. Relevant NTOs for **3Cl** are shown in Figure S46.

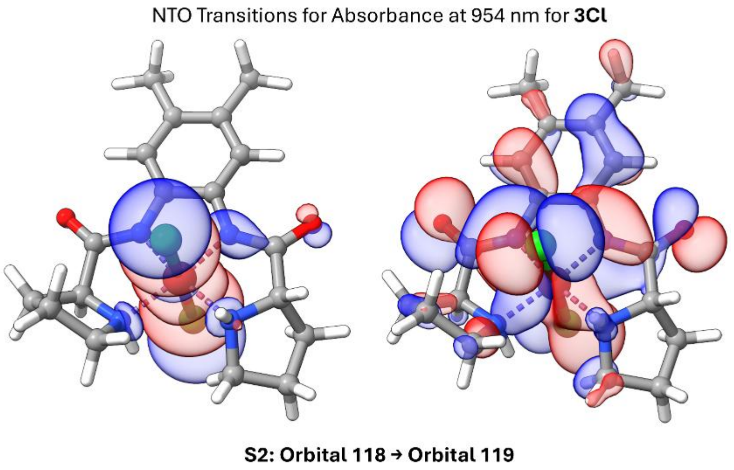

Figure S46. Computed NTOs for UV/Vis spectrum for complex **3Cl** with increased total roots and TDA approximation turned off.

Furthermore, we also computed the UV/Vis spectra for complexes **3Cl**, **4Cl**, and $[LNi^{IV}Cl(MeOH)]^{+}$ to investigate the effects of axial ligation state. As shown in Figure S41, there were notable differences in the UV/Vis spectra. Removing an axial chloride from **4Cl** red shifted the UV/Vis spectrum by >80 nm, while substituting one of the chloride ions with a neutral methanol molecule from the solvent also red shifted the spectrum but by a much smaller amount, by 30 nm. These changes were found to predominantly affect the NTOs shown in figure S46 (for e.g. the corresponding transition between orbitals 118 and 119 in **4Cl**). The intensities were normalized to show the differences more clearly.

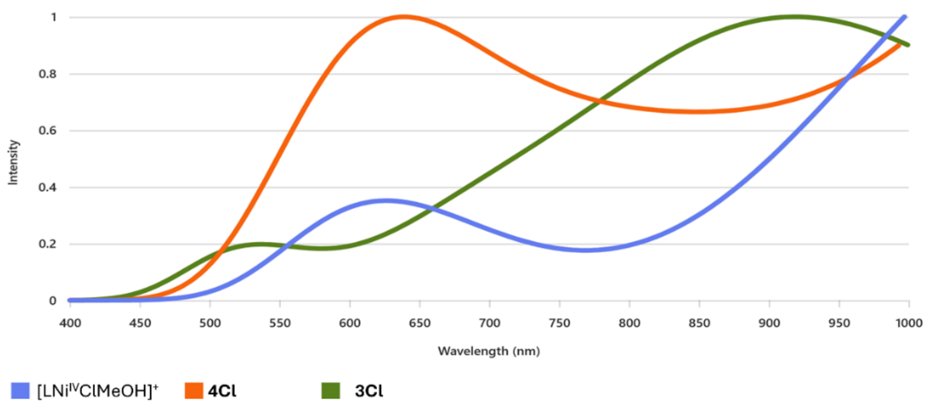

Figure S47. Computed UV/Vis spectra for various axial ligation states.

Finally, Table S13 demonstrates the significant electron donation of the peptide backbone to nickel as indicated in the second order perturbation analysis in NBO basis. The relevant transitions are also mentioned for **4Cl**. It is surprising to see that transition 1 is stronger than transition 2.

Table S13. Second order perturbation energies for various transitions in **4Cl**.

S. No.	Transition	Energy (kcal/mol)
1.	LP amide N1 -> BD* of Ni- amide N2	9.2
2.	LP amide N1 -> BD* of C=O attached to amide N1	3.8
3.	LP of pyrrolidine N3 -> BD* of Ni – amide N1	3.8
4.	BD of pyrrolidine N3-H -> LP* of Ni	5.4
5.	LP of Cl1 -> LP* of Ni	32.8
6.	LP of Cl2 -> LP* of Ni	25.1

References:

1. Kumar, S.; Munjal, M.; Singh, J.; Gupta, R. Nickel and Copper Complexes of Pyrrolecarboxamide Ligands -Stabilization of M^{3+} Species and Isolation of Ni^{3+} Complexes. *Eur. J. Inorg. Chem.* **2014**, *2014* (29), 4957–4965. https://doi.org/10.1002/ejic.201402361.
2. Weeks, C. L.; Turner, P.; Fenton, R. R.; Lay, P. A. Nickel(II) Complexes with Amide Ligands: Oxidative Dehydrogenation of the Amines in a Tetradentate Diamide-Diamine Ligand. *J. Chem. Soc. Dalt. Trans.* **2002**, No. 6, 931–940. https://doi.org/10.1039/b107378h.

3. Wu, H. Y.; Ren, H. S.; Zhu, Q.; Li, X. Y. A Modified Two-Sphere Model for Solvent Reorganization Energy in Electron Transfer. *Phys. Chem. Chem. Phys.* **2012**, *14* (16), 5538–5544. https://doi.org/10.1039/c2cp23759h.
4. Sayfutyarova, E. R.; Goldsmith, Z. K.; Hammes-Schiffer, S. Theoretical Study of C-H Bond Cleavage via Concerted Proton-Coupled Electron Transfer in Fluorenyl-Benzoates. *J. Am. Chem. Soc.* **2018**, *140* (46), 15641–15645. https://doi.org/10.1021/jacs.8b10461.

Coordinates:

51

[LNi^{IV}Cl$_2$]

Ni	-0.81110500	0.01451700	-0.20034000
N	-2.00515200	1.55889100	-0.55500000
N	-2.13165800	-1.36900300	0.31740500
C	-1.15716300	2.76166400	-0.85560500
C	-3.02026800	1.96299600	0.47247800
H	-2.46775500	1.26645700	-1.41024200
N	0.49210300	-1.20320000	0.29170400
N	0.58234300	1.22650400	-0.16774800
C	-1.41270100	-2.66517700	0.48061900
C	-3.33223100	-1.60894000	-0.53672400
H	-2.36571800	-1.00788100	1.24809600
C	-1.82963000	3.91839000	-0.13166500
C	0.28077200	2.55693900	-0.40258000
H	-1.11600700	2.90523700	-1.93646100
C	-2.46214500	3.23398000	1.06862700
H	-3.14865300	1.16637800	1.20145600
H	-3.96738200	2.14097900	-0.03842300
C	1.76450000	-0.74232100	0.19215400
C	0.08555600	-2.49804400	0.57053700
C	1.81700700	0.67221100	-0.06568400
C	-1.89458700	-3.50781900	-0.69295800
H	-1.73717000	-3.12606900	1.41746300
H	-4.21788100	-1.22873000	-0.03267000
H	-3.20786400	-1.06345100	-1.47183600
H	-1.10360600	4.68763500	0.12147500
H	-2.59156000	4.36622300	-0.76861800
O	1.05601800	3.47536500	-0.25604200
H	-1.70553200	2.98336300	1.81615000
H	-3.22864800	3.83089900	1.55575400
C	2.95640200	-1.47191500	0.28216200
O	0.82533100	-3.40370800	0.89063100
C	3.05624100	1.30366800	-0.22887700
H	-1.35760600	-3.22045700	-1.59875900
H	-1.73341600	-4.56849500	-0.52104800
C	4.16765500	-0.83213000	0.14724100
H	2.91595800	-2.53287200	0.46968000
C	4.21897600	0.57791300	-0.11407000

H	3.09032900	2.36314700	-0.42827300
C	5.43567400	-1.60035800	0.26576300
C	5.53875200	1.24780600	-0.26298700
H	6.03333700	-1.51780600	-0.64389200
H	5.24964100	-2.65312400	0.45774400
H	6.05945400	-1.20959600	1.07186200
H	6.11587600	0.80620600	-1.07744300
H	5.42800000	2.31014200	-0.46047000
H	6.14605300	1.12776700	0.63604700
C	-3.35796500	-3.10931000	-0.78547900
H	-3.80755100	-3.35483000	-1.74448200
H	-3.93350200	-3.61508000	-0.00942800
Cl	-0.76661700	-0.44595900	-2.47418600
Cl	-1.31462800	0.17975600	2.88929700

51

$[LNi^{III}Cl_2]^-$

Ni	-0.84828200	0.01982700	0.02174300
N	-2.05406600	1.56172900	-0.37925000
N	-2.14103300	-1.42102800	0.50929900
C	-1.21130900	2.72007200	-0.83512500
C	-3.00469700	2.06580800	0.65285200
H	-2.55060400	1.18800700	-1.18315600
N	0.47868400	-1.28485300	0.21835200
N	0.56258300	1.20982600	-0.26659400
C	-1.40379000	-2.72574900	0.52467900
C	-3.40486500	-1.60765300	-0.26123200
H	-2.33249600	-1.14079600	1.46853600
C	-1.76536100	3.93587200	-0.10575900
C	0.27394900	2.48141400	-0.58232600
H	-1.31391000	2.81178100	-1.91795200
C	-2.36427700	3.33941100	1.15743400
H	-3.14864800	1.31496200	1.42680200
H	-3.96503700	2.26799400	0.17412700
C	1.77510700	-0.77054200	0.11564100
C	0.09996200	-2.55691300	0.41203000
C	1.82195700	0.61348100	-0.14156700
C	-2.04233400	-3.52316300	-0.60365100
H	-1.60614700	-3.22451600	1.47698500
H	-4.24038800	-1.19759400	0.30230300

H	-3.31529500	-1.06180300	-1.20029700
H	-0.97951200	4.66650900	0.07512700
H	-2.53921300	4.41783000	-0.70388100
O	1.08028900	3.40177200	-0.72745500
H	-1.58008700	3.08851600	1.87420200
H	-3.07848000	3.99387000	1.65170100
C	2.96454200	-1.48269600	0.24750700
O	0.83794400	-3.54158800	0.49768400
C	3.05714800	1.24506300	-0.26371400
H	-1.61128100	-3.21454400	-1.55819800
H	-1.88230800	-4.59162100	-0.48432600
C	4.19788000	-0.84728300	0.12714700
H	2.91878500	-2.54367400	0.44309200
C	4.24453000	0.52960200	-0.13154000
H	3.08401900	2.30620900	-0.46174600
C	5.46171500	-1.63405100	0.27429300
C	5.55903500	1.23146100	-0.26317500
H	6.08253300	-1.57591800	-0.62267100
H	5.25633800	-2.68478600	0.46777600
H	6.07948000	-1.25955600	1.09376900
H	6.16260200	0.81309800	-1.07191500
H	5.42561500	2.29241200	-0.46382000
H	6.16187500	1.13743500	0.64300300
C	-3.49961900	-3.10173200	-0.52042900
H	-4.06602000	-3.32514000	-1.42200700
H	-3.99113500	-3.60588200	0.31350800
Cl	-1.10671300	-0.42635000	-2.50583700
Cl	-0.83712500	0.41202400	2.59509400

16

ClThioAnisole_ElecRadcat.log

16

Final Energy = -1129.2181236200

C	-0.9974910	0.2794980	-0.0001440
C	-0.4303780	-0.9934890	-0.0002210
C	0.9472870	-1.1509080	-0.0002400
C	1.7639990	-0.0341270	-0.0000560
C	1.2200030	1.2426940	-0.0000690
C	-0.1526500	1.3938180	-0.0001290
H	-1.0526170	-1.8769570	-0.0005030
H	1.3806940	-2.1414260	-0.0004410

H 1.8658050 2.1096650 -0.0001630

H -0.5766190 2.3902030 -0.0003250

S -2.7249920 0.5907090 0.0000930

C -3.4414630 -1.0525570 0.0001710

H -3.1630930 -1.6107150 0.8916710

H -4.5181820 -0.9087350 -0.0000880

H -3.1624090 -1.6110710 -0.8908320

Cl 3.4923790 -0.2301100 0.0001950

19

MeThioAnisole_ElecRadCat.log

19

Final Energy = -708.9420893880

C 0.6074180 -0.2671310 0.0000040

C 0.0717270 1.0170640 0.0000010

C -1.3055030 1.1975860 -0.0000130

C -2.1865590 0.1230910 -0.0000250

C -1.6342960 -1.1598940 -0.0000110

C -0.2679980 -1.3577760 -0.0000060

H 0.7168780 1.8846350 0.0000130

H -1.7008920 2.2071120 -0.0000220

H -2.2917880 -2.0220830 -0.0000160

H 0.1314390 -2.3649710 -0.0000090

S 2.3301030 -0.6256760 0.0000090

C 3.0903750 0.9978030 -0.0000040

H 2.8258200 1.5643540 0.8905600

H 4.1632830 0.8271830 -0.0000270

H 2.8257750 1.5643520 -0.8905550

C -3.6678780 0.3227500 0.0000160

H -4.1335330 -0.1352760 -0.8737520

H -4.1333580 -0.1344030 0.8743420

H -3.9289850 1.3789510 -0.0004570

20

OMeThioAnisole_ElecRadCat.log

20

Final Energy = -784.1801479080

C 1.0078220 -0.2538940 -0.0001220

C 0.5540570 1.0655710 -0.0002750

C -0.8005230 1.3435150 -0.0002110

C -1.7371580 0.3137100 -0.0000810

C -1.2946020 -1.0069750 -0.0000670

C 0.0639040 -1.2793080 -0.0000510
H 1.2532040 1.8901040 -0.0003790
H -1.1492960 2.3681730 -0.0002930
H -1.9969120 -1.8282230 -0.0000600
H 0.3933660 -2.3114530 -0.0000470
S 2.7078980 -0.7190850 -0.0001370
C 3.5642540 0.8560690 0.0004930
H 3.3344640 1.4379530 0.8909670
H 4.6249080 0.6210840 0.0011640
H 3.3355680 1.4381320 -0.8901260
O -3.0430660 0.6875640 -0.0001380
C -4.0133760 -0.3415870 0.0003920
H -3.9304370 -0.9719130 0.8892050
H -4.9813870 0.1501740 0.0010630
H -3.9315960 -0.9717720 -0.8886610

16

RadCatClThioAnisole_ElecNeutral.log

16

Final Energy = -1129.4268823000

C -0.9903110 0.2610480 -0.0001160
C -0.4226140 -1.0310670 -0.0002940
C 0.9370470 -1.1753930 -0.0002330
C 1.7550260 -0.0346500 -0.0000360
C 1.2092460 1.2554890 -0.0001170
C -0.1491220 1.4023810 -0.0001870
H -1.0518860 -1.9082810 -0.0005120
H 1.3884340 -2.1565940 -0.0003570
H 1.8611750 2.1162970 -0.0000850
H -0.5907340 2.3896200 -0.0002400
S -2.6584720 0.5830230 0.0001060
C -3.4555850 -1.0088530 0.0002120
H -3.1908930 -1.5671480 0.8951840
H -4.5213840 -0.8037920 0.0004150
H -3.1913690 -1.5670800 -0.8949580
Cl 3.4429470 -0.2261830 0.0002050

19

RadCatMeThioAnisole_ElecNeutral.log

19

Final Energy = -709.1446387850

C -0.6093260 0.2514970 -0.0000900

C	-0.0677370	-1.0525100	-0.0004230
C	1.2882440	-1.2145640	-0.0007720
C	2.1649620	-0.1067180	-0.0008330
C	1.6137050	1.1859950	-0.0006430
C	0.2592190	1.3726730	-0.0002390
H	-0.7169500	-1.9153260	-0.0005460
H	1.7070590	-2.2124210	-0.0012200
H	2.2738170	2.0428530	-0.0009740
H	-0.1618710	2.3692440	-0.0001790
S	-2.2690000	0.6123600	0.0003410
C	-3.1039940	-0.9610750	0.0002680
H	-2.8522860	-1.5260240	0.8946630
H	-4.1648160	-0.7320350	0.0006070
H	-2.8528210	-1.5256440	-0.8945200
C	3.6275460	-0.3226610	0.0007080
H	4.1846260	0.6087460	-0.0190200
H	3.9265530	-0.8918820	0.8833240
H	3.9249760	-0.9310780	-0.8554540

20

RadCatOMeThioAnisole_ElecNeutral.log

20

Final Energy = -784.3718821410

C	-1.0086400	-0.2342400	-0.0001500
C	-0.5546620	1.1035380	-0.0001390
C	0.7816620	1.3661750	-0.0002220
C	1.7186700	0.3067990	-0.0002070
C	1.2763140	-1.0310970	-0.0002930
C	-0.0628340	-1.2907940	-0.0002540
H	-1.2593610	1.9216730	-0.0001190
H	1.1545200	2.3806720	-0.0002430
H	1.9851370	-1.8450770	-0.0004650
H	-0.4142030	-2.3142130	-0.0003190
S	-2.6441320	-0.7088620	0.0000880
C	-3.5774220	0.8118280	0.0003130
H	-3.3668630	1.3926550	-0.8941200
H	-4.6215990	0.5154250	0.0007280
H	-3.3662260	1.3928580	0.8944720
O	2.9844490	0.6713560	-0.0002280
C	4.0098200	-0.3301660	0.0006210
H	3.9383430	-0.9483950	-0.8927250

H 4.9473010 0.2125840 0.0021850

H 3.9360140 -0.9494890 0.8930020

16

RadCatThioAnisole_ElecNeutral.log

16

Final Energy = -669.8196244500

C 0.1243080 -0.2124880 -0.0000280

C -0.3148910 1.1289980 -0.0000660

C -1.6605280 1.3896510 -0.0000080

C -2.5857910 0.3386970 0.0000570

C -2.1581040 -0.9897490 0.0000150

C -0.8172180 -1.2745460 -0.0000420

H 0.3979160 1.9401400 -0.0001610

H -2.0092880 2.4126110 -0.0000350

H -3.6444890 0.5611140 0.0001430

H -2.8808720 -1.7929450 0.0000360

H -0.4645500 -2.2974350 -0.0000690

S 1.7524740 -0.6973750 0.0000050

C 2.7123050 0.7998660 0.0000590

H 2.5051650 1.3830520 0.8942440

H 3.7510420 0.4857210 -0.0001210

H 2.5050120 1.3831640 -0.8940410

51

SP_Ni_III_Geom_Ni_IV.log

51

Final Energy = -3499.1612678800

Ni -0.8111050 0.0145170 -0.2003400

N -2.0051520 1.5588910 -0.5550000

N -2.1316580 -1.3690030 0.3174050

C -1.1571630 2.7616640 -0.8556050

C -3.0202680 1.9629960 0.4724780

H -2.4677550 1.2664570 -1.4102420

N 0.4921030 -1.2032000 0.2917040

N 0.5823430 1.2265040 -0.1677480

C -1.4127010 -2.6651770 0.4806190

C -3.3322310 -1.6089400 -0.5367240

H -2.3657180 -1.0078810 1.2480960

C -1.8296300 3.9183900 -0.1316650

C 0.2807720 2.5569390 -0.4025800

H -1.1160070 2.9052370 -1.9364610

C	-2.4621450	3.2339800	1.0686270
H	-3.1486530	1.1663780	1.2014560
H	-3.9673820	2.1409790	-0.0384230
C	1.7645000	-0.7423210	0.1921540
C	0.0855560	-2.4980440	0.5705370
C	1.8170070	0.6722110	-0.0656840
C	-1.8945870	-3.5078190	-0.6929580
H	-1.7371700	-3.1260690	1.4174630
H	-4.2178810	-1.2287300	-0.0326700
H	-3.2078640	-1.0634510	-1.4718360
H	-1.1036060	4.6876350	0.1214750
H	-2.5915600	4.3662230	-0.7686180
O	1.0560180	3.4753650	-0.2560420
H	-1.7055320	2.9833630	1.8161500
H	-3.2286480	3.8308990	1.5557540
C	2.9564020	-1.4719150	0.2821620
O	0.8253310	-3.4037080	0.8906310
C	3.0562410	1.3036680	-0.2288770
H	-1.3576060	-3.2204570	-1.5987590
H	-1.7334160	-4.5684950	-0.5210480
C	4.1676550	-0.8321300	0.1472410
H	2.9159580	-2.5328720	0.4696800
C	4.2189760	0.5779130	-0.1140700
H	3.0903290	2.3631470	-0.4282730
C	5.4356740	-1.6003580	0.2657630
C	5.5387520	1.2478060	-0.2629870
H	6.0333370	-1.5178060	-0.6438920
H	5.2496410	-2.6531240	0.4577440
H	6.0594540	-1.2095960	1.0718620
H	6.1158760	0.8062060	-1.0774430
H	5.4280000	2.3101420	-0.4604700
H	6.1460530	1.1277670	0.6360470
C	-3.3579650	-3.1093100	-0.7854790
H	-3.8075510	-3.3548300	-1.7444820
H	-3.9335020	-3.6150800	-0.0094280
Cl	-0.7666170	-0.4459590	-2.4741860
Cl	-1.3146280	0.1797560	2.8892970

51

SP_Ni_III_LD_ClCl_G1.log

51

Final Energy = -3499.0222084000

Ni	-0.8111050	0.0145170	-0.2003400
N	-2.0051520	1.5588910	-0.5550000
N	-2.1316580	-1.3690030	0.3174050
C	-1.1571630	2.7616640	-0.8556050
C	-3.0202680	1.9629960	0.4724780
H	-2.4677550	1.2664570	-1.4102420
N	0.4921030	-1.2032000	0.2917040
N	0.5823430	1.2265040	-0.1677480
C	-1.4127010	-2.6651770	0.4806190
C	-3.3322310	-1.6089400	-0.5367240
H	-2.3657180	-1.0078810	1.2480960
C	-1.8296300	3.9183900	-0.1316650
C	0.2807720	2.5569390	-0.4025800
H	-1.1160070	2.9052370	-1.9364610
C	-2.4621450	3.2339800	1.0686270
H	-3.1486530	1.1663780	1.2014560
H	-3.9673820	2.1409790	-0.0384230
C	1.7645000	-0.7423210	0.1921540
C	0.0855560	-2.4980440	0.5705370
C	1.8170070	0.6722110	-0.0656840
C	-1.8945870	-3.5078190	-0.6929580
H	-1.7371700	-3.1260690	1.4174630
H	-4.2178810	-1.2287300	-0.0326700
H	-3.2078640	-1.0634510	-1.4718360
H	-1.1036060	4.6876350	0.1214750
H	-2.5915600	4.3662230	-0.7686180
O	1.0560180	3.4753650	-0.2560420
H	-1.7055320	2.9833630	1.8161500
H	-3.2286480	3.8308990	1.5557540
C	2.9564020	-1.4719150	0.2821620
O	0.8253310	-3.4037080	0.8906310
C	3.0562410	1.3036680	-0.2288770
H	-1.3576060	-3.2204570	-1.5987590
H	-1.7334160	-4.5684950	-0.5210480
C	4.1676550	-0.8321300	0.1472410
H	2.9159580	-2.5328720	0.4696800
C	4.2189760	0.5779130	-0.1140700
H	3.0903290	2.3631470	-0.4282730
C	5.4356740	-1.6003580	0.2657630

C	5.5387520	1.2478060	-0.2629870
H	6.0333370	-1.5178060	-0.6438920
H	5.2496410	-2.6531240	0.4577440
H	6.0594540	-1.2095960	1.0718620
H	6.1158760	0.8062060	-1.0774430
H	5.4280000	2.3101420	-0.4604700
H	6.1460530	1.1277670	0.6360470
C	-3.3579650	-3.1093100	-0.7854790
H	-3.8075510	-3.3548300	-1.7444820
H	-3.9335020	-3.6150800	-0.0094280
Cl	-0.7666170	-0.4459590	-2.4741860
Cl	-1.3146280	0.1797560	2.8892970

51

SP_Ni_II_LD_ClCl_G1.log

51

Final Energy = -3499.2085120300

Ni	-0.8482820	0.0198270	0.0217430
N	-2.0540660	1.5617290	-0.3792500
N	-2.1410330	-1.4210280	0.5092990
C	-1.2113090	2.7200720	-0.8351250
C	-3.0046970	2.0658080	0.6528520
H	-2.5506040	1.1880070	-1.1831560
N	0.4786840	-1.2848530	0.2183520
N	0.5625830	1.2098260	-0.2665940
C	-1.4037900	-2.7257490	0.5246790
C	-3.4048650	-1.6076530	-0.2612320
H	-2.3324960	-1.1407960	1.4685360
C	-1.7653610	3.9358720	-0.1057590
C	0.2739490	2.4814140	-0.5823260
H	-1.3139100	2.8117810	-1.9179520
C	-2.3642770	3.3394110	1.1574340
H	-3.1486480	1.3149620	1.4268020
H	-3.9650370	2.2679940	0.1741270
C	1.7751070	-0.7705420	0.1156410
C	0.0999620	-2.5569130	0.4120300
C	1.8219570	0.6134810	-0.1415670
C	-2.0423340	-3.5231630	-0.6036510
H	-1.6061470	-3.2245160	1.4769850
H	-4.2403880	-1.1975940	0.3023030
H	-3.3152950	-1.0618030	-1.2002970

H	-0.9795120	4.6665090	0.0751270
H	-2.5392130	4.4178300	-0.7038810
O	1.0802890	3.4017720	-0.7274550
H	-1.5800870	3.0885160	1.8742020
H	-3.0784800	3.9938700	1.6517010
C	2.9645420	-1.4826960	0.2475070
O	0.8379440	-3.5415880	0.4976840
C	3.0571480	1.2450630	-0.2637140
H	-1.6112810	-3.2145440	-1.5581980
H	-1.8823080	-4.5916210	-0.4843260
C	4.1978800	-0.8472830	0.1271470
H	2.9187850	-2.5436740	0.4430920
C	4.2445300	0.5296020	-0.1315400
H	3.0840190	2.3062090	-0.4617460
C	5.4617150	-1.6340510	0.2742930
C	5.5590350	1.2314610	-0.2631750
H	6.0825330	-1.5759180	-0.6226710
H	5.2563380	-2.6847860	0.4677760
H	6.0794800	-1.2595560	1.0937690
H	6.1626020	0.8130980	-1.0719150
H	5.4256150	2.2924120	-0.4638200
H	6.1618750	1.1374350	0.6430030
C	-3.4996190	-3.1017320	-0.5204290
H	-4.0660200	-3.3251400	-1.4220070
H	-3.9911350	-3.6058820	0.3135080
Cl	-1.1067130	-0.4263500	-2.5058370
Cl	-0.8371250	0.4120240	2.5950940

51

SP_Ni_IV_Geom_Ni_III.log

51

Final Energy = -3498.9726111100

Ni	-0.8482820	0.0198270	0.0217430
N	-2.0540660	1.5617290	-0.3792500
N	-2.1410330	-1.4210280	0.5092990
C	-1.2113090	2.7200720	-0.8351250
C	-3.0046970	2.0658080	0.6528520
H	-2.5506040	1.1880070	-1.1831560
N	0.4786840	-1.2848530	0.2183520
N	0.5625830	1.2098260	-0.2665940
C	-1.4037900	-2.7257490	0.5246790

C	-3.4048650	-1.6076530	-0.2612320
H	-2.3324960	-1.1407960	1.4685360
C	-1.7653610	3.9358720	-0.1057590
C	0.2739490	2.4814140	-0.5823260
H	-1.3139100	2.8117810	-1.9179520
C	-2.3642770	3.3394110	1.1574340
H	-3.1486480	1.3149620	1.4268020
H	-3.9650370	2.2679940	0.1741270
C	1.7751070	-0.7705420	0.1156410
C	0.0999620	-2.5569130	0.4120300
C	1.8219570	0.6134810	-0.1415670
C	-2.0423340	-3.5231630	-0.6036510
H	-1.6061470	-3.2245160	1.4769850
H	-4.2403880	-1.1975940	0.3023030
H	-3.3152950	-1.0618030	-1.2002970
H	-0.9795120	4.6665090	0.0751270
H	-2.5392130	4.4178300	-0.7038810
O	1.0802890	3.4017720	-0.7274550
H	-1.5800870	3.0885160	1.8742020
H	-3.0784800	3.9938700	1.6517010
C	2.9645420	-1.4826960	0.2475070
O	0.8379440	-3.5415880	0.4976840
C	3.0571480	1.2450630	-0.2637140
H	-1.6112810	-3.2145440	-1.5581980
H	-1.8823080	-4.5916210	-0.4843260
C	4.1978800	-0.8472830	0.1271470
H	2.9187850	-2.5436740	0.4430920
C	4.2445300	0.5296020	-0.1315400
H	3.0840190	2.3062090	-0.4617460
C	5.4617150	-1.6340510	0.2742930
C	5.5590350	1.2314610	-0.2631750
H	6.0825330	-1.5759180	-0.6226710
H	5.2563380	-2.6847860	0.4677760
H	6.0794800	-1.2595560	1.0937690
H	6.1626020	0.8130980	-1.0719150
H	5.4256150	2.2924120	-0.4638200
H	6.1618750	1.1374350	0.6430030
C	-3.4996190	-3.1017320	-0.5204290
H	-4.0660200	-3.3251400	-1.4220070
H	-3.9911350	-3.6058820	0.3135080

Cl -1.1067130 -0.4263500 -2.5058370

Cl -0.8371250 0.4120240 2.5950940

16

ThioAnisole_ElecRadCat.log

16

Final Energy = -669.6099369780

C	0.1177770	-0.2346910	-0.0000840
C	-0.3204630	1.0884380	-0.0001650
C	-1.6799950	1.3696240	-0.0000460
C	-2.6162340	0.3481890	0.0001020
C	-2.1802280	-0.9713760	0.0001040
C	-0.8288720	-1.2643290	-0.0000310
H	0.3889660	1.9039770	-0.0004450
H	-2.0036360	2.4029520	-0.0001390
H	-3.6740430	0.5748110	0.0003320
H	-2.8984340	-1.7815670	0.0001540
H	-0.4972170	-2.2956990	-0.0001830
S	1.8095130	-0.7128320	-0.0000590
C	2.6853340	0.8518830	0.0001800
H	2.4623470	1.4356820	0.8908610
H	3.7427830	0.6031600	0.0007200
H	2.4631050	1.4355770	-0.8907100

16

ClThioAnisole.log

16

Final Energy = -1129.4308660000

C	-0.9974910	0.2794980	-0.0001440
C	-0.4303780	-0.9934890	-0.0002210
C	0.9472870	-1.1509080	-0.0002400
C	1.7639990	-0.0341270	-0.0000560
C	1.2200030	1.2426940	-0.0000690
C	-0.1526500	1.3938180	-0.0001290
H	-1.0526170	-1.8769570	-0.0005030
H	1.3806940	-2.1414260	-0.0004410
H	1.8658050	2.1096650	-0.0001630
H	-0.5766190	2.3902030	-0.0003250
S	-2.7249920	0.5907090	0.0000930
C	-3.4414630	-1.0525570	0.0001710
H	-3.1630930	-1.6107150	0.8916710

H -4.5181820 -0.9087350 -0.0000880
H -3.1624090 -1.6110710 -0.8908320
Cl 3.4923790 -0.2301100 0.0001950
51
M06L_LS_Ni_II_LD_ClCl_G1.log
51
Final Energy = -3499.1573950700
Ni -0.8482820 0.0198270 0.0217430
N -2.0540660 1.5617290 -0.3792500
N -2.1410330 -1.4210280 0.5092990
C -1.2113090 2.7200720 -0.8351250
C -3.0046970 2.0658080 0.6528520
H -2.5506040 1.1880070 -1.1831560
N 0.4786840 -1.2848530 0.2183520
N 0.5625830 1.2098260 -0.2665940
C -1.4037900 -2.7257490 0.5246790
C -3.4048650 -1.6076530 -0.2612320
H -2.3324960 -1.1407960 1.4685360
C -1.7653610 3.9358720 -0.1057590
C 0.2739490 2.4814140 -0.5823260
H -1.3139100 2.8117810 -1.9179520
C -2.3642770 3.3394110 1.1574340
H -3.1486480 1.3149620 1.4268020
H -3.9650370 2.2679940 0.1741270
C 1.7751070 -0.7705420 0.1156410
C 0.0999620 -2.5569130 0.4120300
C 1.8219570 0.6134810 -0.1415670
C -2.0423340 -3.5231630 -0.6036510
H -1.6061470 -3.2245160 1.4769850
H -4.2403880 -1.1975940 0.3023030
H -3.3152950 -1.0618030 -1.2002970
H -0.9795120 4.6665090 0.0751270
H -2.5392130 4.4178300 -0.7038810
O 1.0802890 3.4017720 -0.7274550
H -1.5800870 3.0885160 1.8742020
H -3.0784800 3.9938700 1.6517010
C 2.9645420 -1.4826960 0.2475070
O 0.8379440 -3.5415880 0.4976840
C 3.0571480 1.2450630 -0.2637140
H -1.6112810 -3.2145440 -1.5581980

H	-1.8823080	-4.5916210	-0.4843260
C	4.1978800	-0.8472830	0.1271470
H	2.9187850	-2.5436740	0.4430920
C	4.2445300	0.5296020	-0.1315400
H	3.0840190	2.3062090	-0.4617460
C	5.4617150	-1.6340510	0.2742930
C	5.5590350	1.2314610	-0.2631750
H	6.0825330	-1.5759180	-0.6226710
H	5.2563380	-2.6847860	0.4677760
H	6.0794800	-1.2595560	1.0937690
H	6.1626020	0.8130980	-1.0719150
H	5.4256150	2.2924120	-0.4638200
H	6.1618750	1.1374350	0.6430030
C	-3.4996190	-3.1017320	-0.5204290
H	-4.0660200	-3.3251400	-1.4220070
H	-3.9911350	-3.6058820	0.3135080
Cl	-1.1067130	-0.4263500	-2.5058370
Cl	-0.8371250	0.4120240	2.5950940

51

M06L_MS_Ni_III_LD_ClCl_G1.log

51

Final Energy = -3498.9718609200

Ni	-0.8111050	0.0145170	-0.2003400
N	-2.0051520	1.5588910	-0.5550000
N	-2.1316580	-1.3690030	0.3174050
C	-1.1571630	2.7616640	-0.8556050
C	-3.0202680	1.9629960	0.4724780
H	-2.4677550	1.2664570	-1.4102420
N	0.4921030	-1.2032000	0.2917040
N	0.5823430	1.2265040	-0.1677480
C	-1.4127010	-2.6651770	0.4806190
C	-3.3322310	-1.6089400	-0.5367240
H	-2.3657180	-1.0078810	1.2480960
C	-1.8296300	3.9183900	-0.1316650
C	0.2807720	2.5569390	-0.4025800
H	-1.1160070	2.9052370	-1.9364610
C	-2.4621450	3.2339800	1.0686270
H	-3.1486530	1.1663780	1.2014560
H	-3.9673820	2.1409790	-0.0384230
C	1.7645000	-0.7423210	0.1921540

C	0.0855560	-2.4980440	0.5705370
C	1.8170070	0.6722110	-0.0656840
C	-1.8945870	-3.5078190	-0.6929580
H	-1.7371700	-3.1260690	1.4174630
H	-4.2178810	-1.2287300	-0.0326700
H	-3.2078640	-1.0634510	-1.4718360
H	-1.1036060	4.6876350	0.1214750
H	-2.5915600	4.3662230	-0.7686180
O	1.0560180	3.4753650	-0.2560420
H	-1.7055320	2.9833630	1.8161500
H	-3.2286480	3.8308990	1.5557540
C	2.9564020	-1.4719150	0.2821620
O	0.8253310	-3.4037080	0.8906310
C	3.0562410	1.3036680	-0.2288770
H	-1.3576060	-3.2204570	-1.5987590
H	-1.7334160	-4.5684950	-0.5210480
C	4.1676550	-0.8321300	0.1472410
H	2.9159580	-2.5328720	0.4696800
C	4.2189760	0.5779130	-0.1140700
H	3.0903290	2.3631470	-0.4282730
C	5.4356740	-1.6003580	0.2657630
C	5.5387520	1.2478060	-0.2629870
H	6.0333370	-1.5178060	-0.6438920
H	5.2496410	-2.6531240	0.4577440
H	6.0594540	-1.2095960	1.0718620
H	6.1158760	0.8062060	-1.0774430
H	5.4280000	2.3101420	-0.4604700
H	6.1460530	1.1277670	0.6360470
C	-3.3579650	-3.1093100	-0.7854790
H	-3.8075510	-3.3548300	-1.7444820
H	-3.9335020	-3.6150800	-0.0094280
Cl	-0.7666170	-0.4459590	-2.4741860
Cl	-1.3146280	0.1797560	2.8892970

19

MeThioAnisole.log

19

Final Energy = -709.1488760360

C	-0.6074180	0.2671310	0.0000040
C	-0.0717270	-1.0170640	0.0000010
C	1.3055030	-1.1975860	-0.0000130

C	2.1865590	-0.1230910	-0.0000250
C	1.6342960	1.1598940	-0.0000110
C	0.2679980	1.3577760	-0.0000060
H	-0.7168780	-1.8846350	0.0000130
H	1.7008920	-2.2071120	-0.0000220
H	2.2917880	2.0220830	-0.0000160
H	-0.1314390	2.3649710	-0.0000090
S	-2.3301030	0.6256760	0.0000090
C	-3.0903750	-0.9978030	-0.0000040
H	-2.8258200	-1.5643540	0.8905600
H	-4.1632830	-0.8271830	-0.0000270
H	-2.8257750	-1.5643520	-0.8905550
C	3.6678780	-0.3227500	0.0000160
H	4.1335330	0.1352760	-0.8737520
H	4.1333580	0.1344030	0.8743420
H	3.9289850	-1.3789510	-0.0004570

20

OMeThioAnisole.log

20

Final Energy = -784.3773811270

C	1.0078220	-0.2538940	-0.0001220
C	0.5540570	1.0655710	-0.0002750
C	-0.8005230	1.3435150	-0.0002110
C	-1.7371580	0.3137100	-0.0000810
C	-1.2946020	-1.0069750	-0.0000670
C	0.0639040	-1.2793080	-0.0000510
H	1.2532040	1.8901040	-0.0003790
H	-1.1492960	2.3681730	-0.0002930
H	-1.9969120	-1.8282230	-0.0000600
H	0.3933660	-2.3114530	-0.0000470
S	2.7078980	-0.7190850	-0.0001370
C	3.5642540	0.8560690	0.0004930
H	3.3344640	1.4379530	0.8909670
H	4.6249080	0.6210840	0.0011640
H	3.3355680	1.4381320	-0.8901260
O	-3.0430660	0.6875640	-0.0001380
C	-4.0133760	-0.3415870	0.0003920
H	-3.9304370	-0.9719130	0.8892050
H	-4.9813870	0.1501740	0.0010630
H	-3.9315960	-0.9717720	-0.8886610

16

RadCatClThioAnisole.log

16

Final Energy = -1129.2220312800

C	-0.9903110	0.2610480	-0.0001160
C	-0.4226140	-1.0310670	-0.0002940
C	0.9370470	-1.1753930	-0.0002330
C	1.7550260	-0.0346500	-0.0000360
C	1.2092460	1.2554890	-0.0001170
C	-0.1491220	1.4023810	-0.0001870
H	-1.0518860	-1.9082810	-0.0005120
H	1.3884340	-2.1565940	-0.0003570
H	1.8611750	2.1162970	-0.0000850
H	-0.5907340	2.3896200	-0.0002400
S	-2.6584720	0.5830230	0.0001060
C	-3.4555850	-1.0088530	0.0002120
H	-3.1908930	-1.5671480	0.8951840
H	-4.5213840	-0.8037920	0.0004150
H	-3.1913690	-1.5670800	-0.8949580
Cl	3.4429470	-0.2261830	0.0002050

19

RadCatMeThioAnisole.log

19

Final Energy = -708.9461536600

C	-0.6093260	0.2514970	-0.0000900
C	-0.0677370	-1.0525100	-0.0004230
C	1.2882440	-1.2145640	-0.0007720
C	2.1649620	-0.1067180	-0.0008330
C	1.6137050	1.1859950	-0.0006430
C	0.2592190	1.3726730	-0.0002390
H	-0.7169500	-1.9153260	-0.0005460
H	1.7070590	-2.2124210	-0.0012200
H	2.2738170	2.0428530	-0.0009740
H	-0.1618710	2.3692440	-0.0001790
S	-2.2690000	0.6123600	0.0003410
C	-3.1039940	-0.9610750	0.0002680
H	-2.8522860	-1.5260240	0.8946630
H	-4.1648160	-0.7320350	0.0006070
H	-2.8528210	-1.5256440	-0.8945200
C	3.6275460	-0.3226610	0.0007080

H	4.1846260	0.6087460	-0.0190200
H	3.9265530	-0.8918820	0.8833240
H	3.9249760	-0.9310780	-0.8554540

20

RadCatOMeThioAnisole.log

20

Final Energy = -784.1854851380

C	1.0086400	-0.2342400	0.0001500
C	0.5546620	1.1035380	0.0001390
C	-0.7816620	1.3661750	0.0002220
C	-1.7186700	0.3067990	0.0002070
C	-1.2763140	-1.0310970	0.0002930
C	0.0628340	-1.2907940	0.0002540
H	1.2593610	1.9216730	0.0001190
H	-1.1545200	2.3806720	0.0002430
H	-1.9851370	-1.8450770	0.0004650
H	0.4142030	-2.3142130	0.0003190
S	2.6441320	-0.7088620	-0.0000880
C	3.5774220	0.8118280	-0.0003130
H	3.3668630	1.3926550	0.8941200
H	4.6215990	0.5154250	-0.0007280
H	3.3662260	1.3928580	-0.8944720
O	-2.9844490	0.6713560	0.0002280
C	-4.0098200	-0.3301660	-0.0006210
H	-3.9383430	-0.9483950	0.8927250
H	-4.9473010	0.2125840	-0.0021850
H	-3.9360140	-0.9494890	-0.8930020

16

RadCatThioAnisole.log

16

Final Energy = -669.6132972100

C	0.1243080	-0.2124880	-0.0000280
C	-0.3148910	1.1289980	-0.0000660
C	-1.6605280	1.3896510	-0.0000080
C	-2.5857910	0.3386970	0.0000570
C	-2.1581040	-0.9897490	0.0000150
C	-0.8172180	-1.2745460	-0.0000420
H	0.3979160	1.9401400	-0.0001610
H	-2.0092880	2.4126110	-0.0000350
H	-3.6444890	0.5611140	0.0001430

H	-2.8808720	-1.7929450	0.0000360
H	-0.4645500	-2.2974350	-0.0000690
S	1.7524740	-0.6973750	0.0000050
C	2.7123050	0.7998660	0.0000590
H	2.5051650	1.3830520	0.8942440
H	3.7510420	0.4857200	-0.0001210
H	2.5050120	1.3831640	-0.8940410

16

ThioAnisole.log

16

Final Energy = -669.8230033920

C	0.1177770	-0.2346910	-0.0000840
C	-0.3204630	1.0884380	-0.0001650
C	-1.6799950	1.3696240	-0.0000460
C	-2.6162340	0.3481890	0.0001020
C	-2.1802280	-0.9713760	0.0001040
C	-0.8288720	-1.2643290	-0.0000310
H	0.3889660	1.9039770	-0.0004450
H	-2.0036360	2.4029520	-0.0001390
H	-3.6740430	0.5748110	0.0003320

H	-2.8984340	-1.7815670	0.0001540
H	-0.4972170	-2.2956990	-0.0001830
S	1.8095130	-0.7128320	-0.0000590
C	2.6853340	0.8518830	0.0001800
H	2.4623470	1.4356820	0.8908610
H	3.7427830	0.6031600	0.0007200
H	2.4631050	1.4355770	-0.8907100

52

Cartesian_M06L_LS_Ni_III_LD_ClOH_G1.log

52

Final Energy = -3114.6279990800

Ni	0.8789510	0.0622290	-0.0789140
N	2.0640840	1.6533290	0.2115290
N	2.2249750	-1.3367860	-0.5989720
C	1.2022120	2.8000330	0.6571620
C	2.8870370	2.1187470	-0.9433130
H	2.6514240	1.3560240	0.9838390
N	-0.4059580	-1.2841680	-0.2733820
N	-0.5581700	1.2071700	0.2587400

C	1.5277550	-2.6620340	-0.6190720	H	2.8099770	3.9911060	-2.0385200
C	3.5158580	-1.4974840	0.1321870	C	-2.8817260	-1.5423740	-0.3184750
H	2.3667070	-1.0186800	-1.5537940	O	-0.6912820	-3.5382630	-0.5410520
C	1.6608260	3.9911370	-0.1710970	C	-3.0471130	1.1773890	0.2365010
C	-0.2829810	2.5009040	0.4917650	H	1.7851700	-3.1807980	1.4533710
H	1.3595220	2.9504320	1.7262170	H	2.0793050	-4.5256780	0.3493830
C	2.1624150	3.3446350	-1.4516020	C	-4.1289450	-0.9394210	-0.1955370
H	2.9804770	1.3280060	-1.6831590	H	-2.8068720	-2.5975360	-0.5333760
H	3.8823120	2.3663020	-0.5707180	C	-4.2120450	0.4330750	0.0847160
C	-1.7140410	-0.8014780	-0.1557810	H	-3.1013380	2.2338810	0.4500650
C	0.0242310	-2.5420820	-0.4785100	C	-5.3714470	-1.7543180	-0.3608940
C	-1.7966980	0.5743610	0.1226730	C	-5.5444760	1.0976910	0.2197530
C	2.2096280	-3.4556950	0.4862790	H	-5.9923620	-1.7290330	0.5371080
H	1.7284020	-3.1385690	-1.5824050	H	-5.1402650	-2.7949270	-0.5766220
H	4.3212020	-1.0650900	-0.4569570	H	-5.9965240	-1.3753950	-1.1722440
H	3.4467650	-0.9552590	1.0745140	H	-6.1400740	0.6476430	1.0167730
H	0.8439910	4.6946550	-0.3165300	H	-5.4410800	2.1578960	0.4391110
H	2.4702980	4.5149450	0.3370560	H	-6.1369740	1.0013020	-0.6925610
O	-1.1181300	3.3895460	0.6337800	C	3.6519830	-2.9906250	0.3802610
H	1.3255250	3.0364380	-2.0799100	H	4.2394020	-3.2025010	1.2704580

H	4.1427660	-3.4747380	-0.4650860
O	0.8257420	0.4481140	-1.8926610
H	-0.0562410	0.2088170	-2.2056330
Cl	1.1286970	-0.3740250	2.1771290

61

Cartesian_M06L_LS_Ni_II_LD_MeCN2_G1.log

61

Final Energy = -2844.0208393300

Ni	-0.9267190	0.0687370	0.0395010
N	-2.0861900	1.6096730	-0.5174690
N	-2.2495800	-1.2182960	0.8264800
C	-1.2303770	2.6161030	-1.2407170
C	-2.8440350	2.3434460	0.5464240
H	-2.7417220	1.2037080	-1.1756550
N	0.3724980	-1.2285830	0.4417400
N	0.5181470	1.1244310	-0.5234410
C	-1.5421600	-2.5209530	1.0843730
C	-3.5055700	-1.5185640	0.0691060
H	-2.4868380	-0.7993590	1.7191480
C	-1.6431630	3.9684340	-0.6806040
C	0.2580830	2.3329150	-1.0541250
H	-1.4270040	2.5333580	-2.3106280
C	-2.0733250	3.6298170	0.7371000
H	-2.9254490	1.7324800	1.4421430
H	-3.8508570	2.5404170	0.1763230
C	1.6819380	-0.7982150	0.1829570
C	-0.0393920	-2.4252320	0.8986810
C	1.7632770	0.5021070	-0.3453670
C	-2.2287690	-3.5128570	0.1548710
H	-1.7282230	-2.8098390	2.1212180
H	-4.3292940	-0.9486950	0.4920630
H	-3.3708850	-1.2024610	-0.9645530
H	-0.8187890	4.6755340	-0.7402000
H	-2.4805460	4.3758580	-1.2467820
O	1.0942660	3.1585110	-1.4102200
H	-1.2008530	3.4582410	1.3708310
H	-2.6771970	4.4024270	1.2057840
C	2.8494210	-1.5242380	0.4029010
O	0.6766790	-3.3911240	1.1529420
C	3.0100790	1.0426570	-0.6481350

H	-1.7966290	-3.4483480	-0.8462700
H	-2.1082790	-4.5351970	0.5022290
C	4.0950080	-0.9801490	0.1035940
H	2.7776630	-2.5220790	0.8081490
C	4.1759260	0.3149700	-0.4277100
H	3.0643780	2.0413440	-1.0538260
C	5.3368270	-1.7762680	0.3489050
C	5.5050110	0.9175260	-0.7540790
H	5.9083070	-1.9257670	-0.5697110
H	5.1076650	-2.7562830	0.7613500
H	6.0096780	-1.2700740	1.0444920
H	6.0498900	0.3189580	-1.4872920
H	5.3993090	1.9221680	-1.1573350
H	6.1486810	0.9769920	0.1262070
C	-3.6667140	-3.0244320	0.1502640
H	-4.2552550	-3.4174660	-0.6751460
H	-4.1642650	-3.3057970	1.0793280
N	-0.6687410	0.8624470	2.1408710
C	-0.1231650	1.1155990	3.1231920
C	0.5537590	1.4352330	4.3464940
H	1.5386150	0.9724570	4.3563760
H	0.6687070	2.5132610	4.4370330
H	-0.0178740	1.0677700	5.1959880
N	-1.1820440	-0.7293980	-1.9611860
C	-1.0218480	-1.1207550	-3.0316600
C	-0.8285950	-1.6132740	-4.3637610
H	0.0741790	-1.1815080	-4.7906480
H	-0.7295620	-2.6966040	-4.3468970
H	-1.6789180	-1.3443460	-4.9867080

55

M06L_HS_Ni_III_LD_ClMeOH_G1.log

55

Final Energy = -3153.8809064400

Ni	-0.9682420	0.0729770	0.1463090
N	-2.0199180	1.7368900	-0.6554650
N	-2.2441300	-1.5687310	0.6210540
C	-1.0762430	2.8719580	-0.8570210
C	-3.1948180	2.2436300	0.0859100
H	-2.2773850	1.3271680	-1.5477480
N	0.4736440	-1.2795050	0.3516130

N	0.6149290	1.2368260	-0.2522470
C	-1.4026910	-2.8044070	0.6246340
C	-3.4741410	-1.8527520	-0.1688860
H	-2.4981050	-1.3460760	1.5758290
C	-1.6187800	4.0147710	0.0174700
C	0.3911590	2.5423350	-0.5680330
H	-1.0867870	3.1669870	-1.9091890
C	-2.6203020	3.3437890	0.9474720
H	-3.6622190	1.4361740	0.6481980
H	-3.9345630	2.6428520	-0.6136420
C	1.7296090	-0.8004650	0.1876960
C	0.0940860	-2.5887450	0.4446020
C	1.8094230	0.6156440	-0.1353340
C	-2.0104970	-3.6913580	-0.4599090
H	-1.5161460	-3.3043010	1.5917480
H	-4.3459610	-1.4693060	0.3574120
H	-3.3933790	-1.3329990	-1.1242810
H	-0.8133690	4.5296280	0.5372040
H	-2.1213670	4.7517530	-0.6074180
O	1.2214880	3.4380380	-0.6497590
H	-2.1222200	2.9118920	1.8157080
H	-3.3830910	4.0265320	1.3139300
C	2.9224540	-1.5310260	0.3084710
O	0.8303180	-3.5650050	0.3943570
C	3.0768920	1.1915170	-0.3291950
H	-1.6100400	-3.4004560	-1.4327820
H	-1.7806410	-4.7410360	-0.2969290
C	4.1515490	-0.9367600	0.1257270
H	2.8604550	-2.5798160	0.5539730
C	4.2302550	0.4512100	-0.2046020
H	3.1304460	2.2402130	-0.5781250
C	5.4011360	-1.7344440	0.2666110
C	5.5612190	1.0878600	-0.4086900
H	5.9803300	-1.7304450	-0.6588820
H	5.1887760	-2.7672850	0.5290460
H	6.0558390	-1.3147200	1.0327690
H	6.1195990	0.5940590	-1.2062070
H	5.4656580	2.1401390	-0.6623720
H	6.1811650	1.0084330	0.4865000
C	-3.4883970	-3.3566930	-0.3747490

H -4.0476460 -3.6506650 -1.2607150

H -3.9403640 -3.8571040 0.4840090

O -1.1907190 0.5430240 1.9729190

C -0.2166630 1.1830730 2.7109860

H 0.1629910 2.0855360 2.2104120

H -0.5888730 1.4553150 3.7028010

H 0.6571870 0.5270630 2.8405390

Cl -1.0483160 -0.6526190 -2.3148370

55

M06L_HS_Ni_III_LD_ClMeOH_G2.log

55

Final Energy = -3153.8802039100

Ni 1.0074410 -0.0355030 0.1034160

N 2.1380600 -1.6845700 -0.6245500

N 2.2210620 1.6842980 0.4842240

C 1.2367840 -2.8594930 -0.8041640

C 3.3122540 -2.1205760 0.1635140

H 2.4030920 -1.3047860 -1.5270110

N -0.4901750 1.2419020 0.3536450

N -0.5193400 -1.2908840 -0.1974470

C 1.3002000 2.8029360 0.8622860

C 3.1908950 2.2061320 -0.5179160

H 2.7182460 1.3834050 1.3129650

C 1.8481070 -3.9752210 0.0545600

C -0.2364400 -2.5941740 -0.4747540

H 1.2294750 -3.1502460 -1.8574640

C 2.7666870 -3.2401400 1.0192940

H 3.7174010 -1.2823940 0.7294610

H 4.0957970 -2.4901230 -0.5036680

C -1.7228420 0.7082120 0.1819760

C -0.1696340 2.5630260 0.4890750

C -1.7400620 -0.7167700 -0.1072650

C 1.8807230 4.0450660 0.2003440

H 1.2915630 2.8938770 1.9520210

C 2.5564540 3.4788360 -1.0372990

H 4.1428230 2.4177630 -0.0263490

H 3.3574790 1.4594780 -1.2926310

H 1.0749320 -4.5663050 0.5404340

H 2.4282510 -4.6509870 -0.5730160

O -1.0233540 -3.5313540 -0.4979700

H	2.2029960	-2.8265080	1.8567290
H	3.5504150	-3.8721820	1.4299520
C	-2.9468520	1.3893010	0.2772690
O	-0.9368980	3.5100970	0.3945540
C	-2.9794920	-1.3502280	-0.2997420
H	1.1056490	4.7782220	-0.0092320
H	2.6160810	4.5097110	0.8578510
H	3.2828080	4.1539980	-1.4840130
H	1.8112460	3.2312300	-1.7961380
C	-4.1482160	0.7393970	0.0978060
H	-2.9322950	2.4455710	0.4969000
C	-4.1648470	-0.6570030	-0.2024590
H	-2.9866320	-2.4059790	-0.5235830
C	-5.4316050	1.4867190	0.2084790
C	-5.4654220	-1.3541880	-0.4048430
H	-5.9987780	1.4409190	-0.7233410
H	-5.2654820	2.5324880	0.4525600
H	-6.0777840	1.0551090	0.9752540
H	-6.0372590	-0.9004490	-1.2164950
H	-5.3226950	-2.4061980	-0.6366560
H	-6.0961410	-1.2840080	0.4835860
O	1.3290490	-0.4162870	1.9435830
C	0.3455500	-0.9165160	2.7687630
H	-0.1383340	-1.8089860	2.3454940
H	0.7423980	-1.1579100	3.7589450
H	-0.4601500	-0.1759160	2.8908860
Cl	0.9614990	0.5299130	-2.3818290

55

M06L_HS_Ni_III_LD_ClMeOH_G3.log

55

Final Energy = -3153.8751157600

Ni	0.9443160	0.0693330	0.3398150
N	2.0292130	1.8711350	0.6227500
N	2.1060910	-1.5815700	-0.2851000
C	3.2323730	2.1836050	-0.1944380
H	2.2954490	1.7919460	1.5963130
N	-0.5270940	-1.3075380	0.0520750
N	-0.6394720	1.2719250	0.3487780
C	3.5009960	-1.9861730	-0.0430050
H	1.9740200	-1.4521390	-1.2899230

C	1.6256550	3.8550070	-0.6598570
C	-0.3902280	2.6148100	0.2701740
C	3.1250220	3.6617900	-0.5203410
H	3.1894620	1.5847970	-1.1044980
H	4.1300620	1.9079460	0.3555670
C	-1.7695340	-0.7851000	-0.0086670
C	-0.1520610	-2.6070130	-0.1646770
C	-1.8338620	0.6584220	0.1590140
C	2.0861600	-3.9400630	-0.4665520
H	4.1735170	-1.3701570	-0.6354410
H	3.7232060	-1.8084060	1.0097750
H	1.2781550	3.4548290	-1.6141790
H	1.3005860	4.8895560	-0.5886620
O	-1.2112250	3.4955960	0.0529530
H	3.6783180	3.9330710	-1.4172530
H	3.5103720	4.2662810	0.3032870
C	-2.9658780	-1.4965870	-0.2073650
O	-0.8543670	-3.5451720	-0.5102080
C	-3.0910660	1.2810810	0.1182730
H	1.8942960	-4.8729990	0.0578870
H	1.7718650	-4.0828070	-1.5012570
C	-4.1852920	-0.8602610	-0.2361950
H	-2.9091070	-2.5666680	-0.3375610
C	-4.2487830	0.5572260	-0.0690820
H	-3.1397660	2.3511710	0.2450640
C	-5.4390620	-1.6386610	-0.4377400
C	-5.5686240	1.2462010	-0.0977850
H	-6.1246660	-1.5118250	0.4023360
H	-5.2358120	-2.6999870	-0.5520770
H	-5.9803270	-1.2981180	-1.3224570
H	-6.2348960	0.8608710	0.6765160
H	-5.4632530	2.3179570	0.0469620
H	-6.0824140	1.0795170	-1.0465950
C	3.5560940	-3.4883490	-0.3834060
H	4.1036230	-4.0350880	0.3817430
H	4.0732710	-3.6605380	-1.3249890
C	1.3292230	-2.7714460	0.1332170
H	1.4181190	-2.8095140	1.2276090
C	1.0666200	3.0056060	0.4806120
H	1.0848240	3.5957000	1.4021900

O 1.2070100 -0.1916300 2.2005050

C 0.2747560 -0.7927210 3.0173940

H -0.1127080 -1.7304770 2.5954720

H 0.6852170 -0.9839750 4.0127880

H -0.6033610 -0.1383460 3.1292760

Cl 0.9607960 0.5302240 -2.2044520

52

M06L_HS_Ni_III_LD_ClOH_G1.log

52

Final Energy = -3114.5643482100

Ni 1.0007610 0.1119360 -0.2879820

N 2.1062750 1.8434980 0.2554590

N 2.2586440 -1.6125060 -0.6058710

C 1.1446850 2.8885470 0.7212210

C 2.9933310 2.4529740 -0.7650000

H 2.6349070 1.4993120 1.0479400

N -0.4514010 -1.2849180 -0.4445030

N -0.5692490 1.2352140 0.1747100

C 1.4110870 -2.8373480 -0.5748400

C 3.4684230 -1.8532600 0.2263890

H 2.5367520 -1.4236350 -1.5599450

C 1.5051360 4.1462490 -0.0735100

C -0.3329170 2.5206420 0.5659060

H 1.2774200 3.0493900 1.7930440

C 2.1646990 3.5873750 -1.3234730

H 3.2757310 1.7061580 -1.5046040

H 3.9017170 2.8344840 -0.2919700

C -1.7006930 -0.7990320 -0.2726120

C -0.0906190 -2.6013760 -0.4791070

C -1.7684860 0.6185580 0.0498450

C 1.9621870 -3.6503540 0.5937410

H 1.5722020 -3.3961960 -1.5021150

H 4.3537790 -1.5243550 -0.3141230

H 3.3798780 -1.2612400 1.1371410

H 0.6279230 4.7603740 -0.2627250

H 2.2173260 4.7506580 0.4886120

O -1.1803900 3.3655830 0.8213390

H 1.4137200 3.1893020 -2.0091690

H 2.7613820 4.3188660 -1.8633020

C -2.9017880 -1.5237460 -0.3774150

O	-0.8336620	-3.5714480	-0.4141570
C	-3.0303080	1.2086140	0.2215750
H	1.5257330	-3.2883250	1.5262880
H	1.7253990	-4.7063410	0.4952730
C	-4.1249070	-0.9197810	-0.2060200
H	-2.8457970	-2.5764860	-0.6065320
C	-4.1904370	0.4749800	0.1005980
H	-3.0782190	2.2610340	0.4525590
C	-5.3818890	-1.7073770	-0.3392390
C	-5.5149160	1.1280400	0.2882580
H	-5.9631790	-1.6850010	0.5846310
H	-5.1778240	-2.7458170	-0.5857330
H	-6.0306310	-1.2936000	-1.1134990
H	-6.0820800	0.6515690	1.0901680
H	-5.4109460	2.1833120	0.5252140
H	-6.1308880	1.0386400	-0.6088720
C	3.4473610	-3.3386140	0.5447480
H	3.9654490	-3.5694410	1.4731900
H	3.9260660	-3.9091350	-0.2533680
O	1.1839600	0.5307310	-2.1046940
H	0.5987950	-0.0260070	-2.6373460
Cl	1.1691310	-0.3888370	2.1810520

52

M06L_HS_Ni_III_LD_ClOH_G2.log

52

Final Energy = -3114.5656738600

Ni	1.0137900	0.0669310	-0.2700580
N	2.1031310	1.7298110	0.5096530
N	2.2143240	-1.6613040	-0.6143360
C	1.2010050	2.9029160	0.6640770
C	3.2939710	2.1562000	-0.2597930
H	2.3411200	1.3382730	1.4152060
N	-0.4838560	-1.2307360	-0.3897770
N	-0.5296980	1.3265390	0.0207330
C	1.2952940	-2.8080720	-0.8871270
C	3.2348870	-2.1134150	0.3719720
H	2.6671220	-1.3922780	-1.4789660
C	1.8506430	4.0253320	-0.1531190
C	-0.2576620	2.6380030	0.2720420
H	1.1463050	3.1754140	1.7208600

C	2.7769370	3.2918970	-1.1114210
H	3.6967050	1.3172590	-0.8253160
H	4.0679460	2.5083130	0.4275580
C	-1.7178530	-0.6964970	-0.2418990
C	-0.1552660	-2.5555930	-0.4612300
C	-1.7447240	0.7420440	-0.0304020
C	1.9210840	-4.0068230	-0.1881570
H	1.2360170	-2.9585460	-1.9683720
C	2.6497260	-3.3693830	0.9829470
H	4.1698770	-2.3301430	-0.1487020
H	3.4187070	-1.3255030	1.1008670
H	1.0988390	4.6410030	-0.6415010
H	2.4293760	4.6743380	0.5033620
O	-1.0435510	3.5738600	0.2455490
H	2.2235800	2.8935790	-1.9640240
H	3.5752880	3.9186150	-1.5011980
C	-2.9365860	-1.3887520	-0.3011680
O	-0.9148040	-3.4953190	-0.2802370
C	-2.9882700	1.3762730	0.1417880
H	1.1653240	-4.7340810	0.0988650
H	2.6275380	-4.5003570	-0.8560790
H	3.4105960	-4.0104990	1.4222440
H	1.9405810	-3.0965410	1.7667340
C	-4.1417590	-0.7379490	-0.1477950
H	-2.9165630	-2.4543180	-0.4693590
C	-4.1678530	0.6723650	0.0860170
H	-3.0002120	2.4417260	0.3141780
C	-5.4195780	-1.4985660	-0.2156520
C	-5.4726180	1.3680940	0.2643110
H	-5.9919450	-1.3956660	0.7082990
H	-5.2467670	-2.5566120	-0.3924100
H	-6.0638420	-1.1198260	-1.0116570
H	-6.0282140	0.9587950	1.1101770
H	-5.3370730	2.4332120	0.4313480
H	-6.1146470	1.2389870	-0.6090890
O	1.3627990	0.4494590	-2.0688750
H	0.7727260	1.1347180	-2.4106370
Cl	0.9784920	-0.4727370	2.2500870

52

M06L_HS_Ni_III_LD_ClOH_G3.log

52

Final Energy = -3114.5582898200

Ni	-0.9748160	0.0923090	-0.4172320
N	-1.9270880	2.0087030	-0.5899730
N	-2.2595470	-1.5285310	0.0923890
C	-3.0460200	2.3913480	0.3152040
H	-2.2714100	1.9321790	-1.5379600
N	0.4227730	-1.3269980	-0.0969890
N	0.7001110	1.2283450	-0.4998650
C	-3.6394030	-1.8928240	-0.2657240
H	-2.1944820	-1.4496240	1.1078360
C	-1.2434540	3.8822920	0.7114840
C	0.5585830	2.5868310	-0.5157070
C	-2.7605310	3.8274370	0.7189450
H	-3.0233300	1.7326410	1.1830450
H	-3.9944910	2.2510570	-0.1991450
C	1.6950930	-0.8785150	-0.0638370
C	-0.0230370	-2.6150040	0.0591350
C	1.8519800	0.5513670	-0.2842710
C	-2.3038970	-3.8986430	0.1609280
H	-4.3434340	-1.2834640	0.2964340
H	-3.7751180	-1.6716690	-1.3248290
H	-0.8396720	3.3871930	1.5966210
H	-0.8330960	4.8874910	0.6665460
O	1.4509090	3.4239130	-0.4907970
H	-3.1942650	4.0786880	1.6845780
H	-3.1664980	4.5221970	-0.0186040
C	2.8420300	-1.6612490	0.1480650
O	0.6345210	-3.5881790	0.3939110
C	3.1496880	1.0930680	-0.2459970
H	-2.1003480	-4.8063840	-0.4021530
H	-2.0602210	-4.1068580	1.2036900
C	4.1002100	-1.1030800	0.1665490
H	2.7168210	-2.7216450	0.3047780
C	4.2563290	0.3038830	-0.0332070
H	3.2659740	2.1544750	-0.3976610
C	5.2985870	-1.9548200	0.4008510
C	5.6182260	0.9065800	-0.0131440
H	6.0026470	-1.8857590	-0.4306410
H	5.0278230	-2.9989930	0.5314010

H	5.8477580	-1.6316490	1.2873590
H	6.2583950	0.4686120	-0.7814390
H	5.5815360	1.9805680	-0.1744800
H	6.1203040	0.7220920	0.9385340
C	-3.7548310	-3.4043660	0.0104940
H	-4.2714750	-3.9075320	-0.8044930
H	-4.3297560	-3.5969730	0.9140790
C	-1.4856470	-2.7186960	-0.3258810
H	-1.4973010	-2.6933670	-1.4240970
C	-0.8856460	3.0712490	-0.5320850
H	-0.9869280	3.7099220	-1.4145690
O	-1.3047830	-0.2370980	-2.2265120
H	-0.6587810	0.2148180	-2.7872960
Cl	-0.9502520	0.4556750	2.0848460

49

M06L_HS_Ni_III_LD_G2.log

49

Final Energy = -2578.1189212300

Ni	-1.2631480	0.0679440	-0.0220380
N	-2.2411640	1.9056860	-0.1352040
N	-2.5102380	-1.5646640	0.2814310
C	-1.3431070	2.9352920	-0.7353200
C	-2.7305460	2.4401900	1.1832110
H	-3.0336750	1.7744190	-0.7520860
N	0.1673220	-1.2913430	0.2013610
N	0.3310280	1.2137410	-0.3349190
C	-1.7165490	-2.7507420	0.7314160
C	-3.3104990	-1.9841060	-0.9205130
H	-3.1436060	-1.2959120	1.0253980
C	-1.5933030	4.2042630	0.0673870
C	0.1309200	2.5282440	-0.7076670
H	-1.5893630	3.0529920	-1.7917460
C	-1.8685720	3.6561410	1.4572710
H	-2.6590610	1.6731440	1.9524740
H	-3.7807310	2.7101780	1.0797460
C	1.4216200	-0.8300140	0.0741020
C	-0.2070550	-2.5671100	0.5791240
C	1.5135370	0.5991080	-0.1837250
C	-2.2321390	-3.9235520	-0.0959710
H	-1.8748920	-2.9030080	1.7999110

C	-2.6417300	-3.2543980	-1.3965590
H	-4.3379900	-2.1728630	-0.6099550
H	-3.3266180	-1.1856920	-1.6604140
H	-0.7485090	4.8861090	0.0145990
H	-2.4691740	4.7189470	-0.3264590
O	1.0027140	3.3143070	-1.0143570
H	-0.9344660	3.3619070	1.9401840
H	-2.3639800	4.3674430	2.1124230
C	2.6106380	-1.5698520	0.1470210
O	0.5536530	-3.4867300	0.7976100
C	2.7821590	1.1962470	-0.1942410
H	-1.4762410	-4.6967110	-0.2068660
H	-3.0972270	-4.3670210	0.3959210
H	-3.3052620	-3.8628040	-2.0050330
H	-1.7623740	-3.0212780	-2.0005130
C	3.8650520	-0.9571860	0.1056000
H	2.5410650	-2.6465750	0.2042700
C	3.9524720	0.4443640	-0.0794090
H	2.8404110	2.2740770	-0.2465260
C	5.0950340	-1.7691680	0.2531950
C	5.2736340	1.1115260	-0.1348010
H	5.7402360	-1.6585980	-0.6207180
H	4.8728010	-2.8222760	0.3908260
H	5.6869060	-1.4251900	1.1038830
H	5.8872690	0.6959330	-0.9361740
H	5.1815690	2.1816370	-0.2891830
H	5.8320870	0.9386660	0.7876630

49

M06L_HS_Ni_III_LD_G3.log

49

Final Energy = -2578.0953858400

Ni	-1.1159510	0.1541030	-0.2012560
N	-2.0232410	2.0088200	-0.3219790
N	-2.4073960	-1.4346300	0.1585140
C	-2.7723660	2.4263350	0.8955530
H	-2.6699750	2.0040730	-1.1026760
N	0.2432810	-1.3204720	-0.0541070
N	0.5543970	1.2240530	-0.2927620
C	-3.8080280	-1.7041640	-0.2445900
H	-2.3894960	-1.4841550	1.1783970

C	-1.2686390	4.1897190	0.4403830
C	0.4388820	2.5866070	-0.5299160
C	-2.6991210	3.9368820	0.8853650
H	-2.2621570	2.0148790	1.7691170
H	-3.7743130	2.0060460	0.8650290
C	1.5154480	-0.9073360	-0.0148520
C	-0.2366230	-2.6265500	0.0645270
C	1.6908070	0.5304110	-0.1189610
C	-2.5818880	-3.8057360	0.0673900
H	-4.4817170	-1.0820840	0.3382250
H	-3.9075850	-1.4219940	-1.2917060
H	-0.5860140	4.0926280	1.2856440
H	-1.1079480	5.1728690	0.0077480
O	1.3631150	3.3539380	-0.6950400
H	-2.9252990	4.3763620	1.8536610
H	-3.4076900	4.3401800	0.1602400
C	2.6578510	-1.7172650	0.0743450
O	0.4008460	-3.5978420	0.3979190
C	2.9884130	1.0522750	-0.0118440
H	-2.4303200	-4.6763480	-0.5648440
H	-2.3506430	-4.1062730	1.0894480
C	3.9500890	-1.1740810	0.1506220
H	2.5290890	-2.7905370	0.0651030
C	4.1172990	0.2238900	0.0978150
H	3.1114490	2.1253850	0.0086450
C	5.1236120	-2.0665160	0.2827190
C	5.4694220	0.8226500	0.1655720
H	5.8128870	-1.9226520	-0.5521310
H	4.8391150	-3.1128430	0.3226860
H	5.6947290	-1.8253640	1.1815880
H	6.1007290	0.4461340	-0.6417860
H	5.4400270	1.9057410	0.1062060
H	5.9720630	0.5364040	1.0919630
C	-4.0022370	-3.2160760	-0.0412460
H	-4.5567800	-3.6461040	-0.8717150
H	-4.5748980	-3.4187400	0.8608690
C	-1.6952540	-2.6473730	-0.3374960
H	-1.6942170	-2.5538900	-1.4308530
C	-0.9988070	3.0739900	-0.5831900
H	-1.1485420	3.4462870	-1.5975770

61

M06L_HS_Ni_III_LD_MeCN2_G1.log

61

Final Energy = -2843.7658622900

Ni	1.0283270	0.1349390	0.0794610
N	1.8805790	2.0849000	0.3792660
N	2.3339600	-1.5287320	-0.1777180
C	0.8255830	3.0549080	0.7884180
C	2.6620130	2.7019410	-0.7309660
H	2.4915770	1.9117960	1.1683080
N	-0.3515240	-1.3208930	-0.1883450
N	-0.6788220	1.1976190	0.2655320
C	1.5842910	-2.6629450	-0.7928340
C	3.0791260	-2.1120420	0.9642770
H	3.0172210	-1.2128780	-0.8586990
C	1.0450400	4.2930450	-0.0823470
C	-0.5983080	2.5146290	0.6587000
H	0.9355690	3.2881750	1.8490130
C	1.7149300	3.7216480	-1.3207110
H	2.9941110	1.9359750	-1.4277530
H	3.5468820	3.1907500	-0.3190170
C	-1.6324720	-0.9300330	-0.0839560
C	0.0782320	-2.5629210	-0.6091490
C	-1.8170590	0.4985160	0.1167300
C	2.1750850	-3.9569440	-0.2052880
H	1.7353350	-2.6209220	-1.8740390
H	3.9120370	-1.4648270	1.2273980
H	2.4053020	-2.1635810	1.8193420
H	0.1126580	4.8174100	-0.2754610
H	1.7161400	4.9841580	0.4263850
O	-1.5440580	3.2351390	0.9170520
H	0.9786150	3.2330270	-1.9627280
H	2.2295040	4.4702130	-1.9171850
C	-2.7713540	-1.7482490	-0.1231840
O	-0.6391100	-3.5130250	-0.8581080
C	-3.1243630	1.0081400	0.0974590
H	1.4764000	-4.3851540	0.5136480
H	2.3434250	-4.7070740	-0.9727840
C	-4.0595520	-1.2221210	-0.0932380
H	-2.6299260	-2.8186880	-0.1383300

C	-4.2390180	0.1790730	0.0174620
H	-3.2544360	2.0801550	0.1051270
C	-5.2357840	-2.1225120	-0.1756250
C	-5.6047090	0.7583020	0.0389120
H	-5.8715330	-2.0126840	0.7051890
H	-4.9430000	-3.1641870	-0.2625700
H	-5.8644770	-1.8676470	-1.0308180
H	-6.1883950	0.3528940	0.8674090
H	-5.5845800	1.8398630	0.1293670
H	-6.1520490	0.4957080	-0.8687030
C	3.4498770	-3.5011060	0.4911580
H	3.7452490	-4.1602760	1.3041510
H	4.2817390	-3.4480140	-0.2130160
N	1.0911760	0.3638700	-2.0137690
C	1.0167950	0.4426650	-3.1589590
C	0.9304150	0.5568640	-4.5842650
H	0.0702740	0.0005730	-4.9505160
H	0.8216250	1.6032270	-4.8629420
H	1.8328130	0.1591370	-5.0436880
N	1.0820800	-0.0336130	2.1749170
C	1.0031730	-0.0376280	3.3224380
C	0.9103170	-0.0513320	4.7520780
H	0.3906100	0.8394240	5.0985640
H	0.3608840	-0.9319490	5.0781360
H	1.9068920	-0.0732620	5.1879020

61

M06L_HS_Ni_III_LD_MeCN2_G2.log

61

Final Energy = -2843.7666800300

Ni	-1.0636880	0.0001650	-0.0001150
N	-2.1514460	1.7728390	-0.5376190
N	-2.1519320	-1.7722360	0.5372310
C	-1.2335990	2.8171450	-1.0698990
C	-3.0138290	2.4071310	0.5003990
H	-2.7270810	1.4209700	-1.2934430
N	0.4958380	-1.2350360	0.3723120
N	0.4961640	1.2350610	-0.3722290
C	-1.2344080	-2.8167690	1.0696270
C	-3.0143030	-2.4062910	-0.5009360
H	-2.7276000	-1.4202530	1.2929770

C	-1.6106860	4.1075150	-0.3396830
C	0.2471150	2.4793770	-0.9035480
H	-1.3744800	2.9121460	-2.1480780
C	-2.2088030	3.5999560	0.9618010
H	-3.2505440	1.6888010	1.2821900
H	-3.9509250	2.7289620	0.0421680
C	1.7156860	-0.7045630	0.1812470
C	0.2464070	-2.4792710	0.9036800
C	1.7158470	0.7043630	-0.1809680
C	-1.6115920	-4.1070090	0.3392210
H	-1.3755700	-2.9118230	2.1477650
C	-2.2094420	-3.5992450	-0.9623050
H	-3.9515340	-2.7279400	-0.0428610
H	-3.2507500	-1.6878730	-1.2827300
H	-0.7523770	4.7628200	-0.2158410
H	-2.3613950	4.6473410	-0.9156800
O	1.0927280	3.2829550	-1.2503090
H	-1.4210100	3.2861550	1.6500240
H	-2.8190780	4.3399260	1.4725970
C	2.9491050	-1.3691080	0.2625290
O	1.0917400	-3.2830420	1.2506690
C	2.9493680	1.3687210	-0.2621040
H	-0.7533650	-4.7624350	0.2154320
H	-2.3624620	-4.6467840	0.9150520
H	-2.8197930	-4.3390710	-1.4732200
H	-1.4215100	-3.2855500	-1.6504190
C	4.1581600	-0.6972780	0.1332010
H	2.9429970	-2.4412190	0.3911580
C	4.1582940	0.6967110	-0.1325900
H	2.9434240	2.4408350	-0.3907720
C	5.4404520	-1.4315000	0.2680760
C	5.4407230	1.4307230	-0.2673170
H	6.0309900	-1.3528220	-0.6469950
H	5.2833200	-2.4829060	0.4880990
H	6.0568150	-1.0007750	1.0591550
H	6.0570930	0.9999050	-1.0583400
H	5.2837950	2.4821590	-0.4873430
H	6.0311510	1.3519300	0.6478130
N	-1.1201180	0.4883980	2.0453530
C	-1.0418510	0.6963450	3.1739870

C	-0.9505240	0.9687410	4.5773580
H	-0.5354650	1.9624520	4.7331270
H	-1.9394170	0.9224220	5.0286190
H	-0.3059800	0.2353290	5.0571430
N	-1.1197850	-0.4880500	-2.0455380
C	-1.0410030	-0.6967930	-3.1739870
C	-0.9489730	-0.9702290	-4.5771070
H	-0.5388520	-1.9661580	-4.7318140
H	-1.9369160	-0.9189530	-5.0299090
H	-0.2998720	-0.2404030	-5.0562120

61

M06L_HS_Ni_III_LD_MeCN2_G3.log

61

Final Energy = -2843.7593070200

Ni	-0.9444150	0.1256150	0.0357270
N	-1.8176220	2.0749780	0.0084470
N	-2.1778740	-1.5836270	0.4455240
C	-2.2477600	2.7611720	1.2500340
H	-2.6309970	2.0018950	-0.5938920
N	0.4526850	-1.3628230	-0.0679710
N	0.7179230	1.1943960	-0.3206620
C	-3.5940810	-1.8904110	0.1505720
H	-2.0581550	-1.6789950	1.4529090
C	-1.0545970	4.3887200	0.0110840
C	0.5842370	2.5400650	-0.6152780
C	-2.3067910	4.2233760	0.8625780
H	-1.4863130	2.5942150	2.0121950
H	-3.1814420	2.3304650	1.6035360
C	1.7190870	-0.9340700	-0.1525100
C	0.0154330	-2.6755450	0.0864270
C	1.8726250	0.5049520	-0.2600770
C	-2.2710710	-3.9495350	0.2328380
H	-4.2397640	-1.3372780	0.8273940
H	-3.7985160	-1.5487100	-0.8639150
H	-0.1903370	4.6175370	0.6345920
H	-1.1348180	5.1841850	-0.7244550
O	1.4957040	3.3006400	-0.8748290
H	-2.3227360	4.8897270	1.7219640
H	-3.2057110	4.4183240	0.2758330
C	2.8713280	-1.7351570	-0.1921620

O	0.6989460	-3.6383330	0.3584370
C	3.1702150	1.0339810	-0.2709680
H	-2.1357690	-4.7836200	-0.4508920
H	-1.9439420	-4.2841770	1.2178370
C	4.1574690	-1.1849390	-0.2331210
H	2.7486860	-2.8088090	-0.2076160
C	4.3084080	0.2148360	-0.2722760
H	3.2887130	2.1072160	-0.2494040
C	5.3469330	-2.0700690	-0.2349640
C	5.6590910	0.8243150	-0.3127200
H	5.9638750	-1.8900980	-1.1175330
H	5.0725480	-3.1199430	-0.2120630
H	5.9882090	-1.8615760	0.6237820
H	6.2123310	0.4863380	-1.1912530
H	5.6158800	1.9087080	-0.3292840
H	6.2514680	0.5119140	0.5497140
C	-3.7175850	-3.4201120	0.2674510
H	-4.3150600	-3.8213880	-0.5480450
H	-4.2140640	-3.7045370	1.1925290
C	-1.4722020	-2.7319890	-0.1818160
H	-1.5763780	-2.5773440	-1.2636910
C	-0.8610580	3.0131730	-0.6527880
H	-1.1101810	3.0511320	-1.7160890
N	-1.4671530	-0.0050540	-1.9841110
C	-1.7481270	-0.0467370	-3.0986680
C	-2.0984440	-0.1000290	-4.4868330
H	-1.4756620	0.5897940	-5.0523440
H	-1.9481240	-1.1077220	-4.8681960
H	-3.1426780	0.1760450	-4.6164110
N	-0.6092300	0.2067890	2.1219500
C	-0.3388780	0.1837980	3.2398510
C	-0.0065590	0.1647640	4.6334260
H	0.6034870	-0.7076330	4.8577500
H	0.5498200	1.0630680	4.8926480
H	-0.9150980	0.1256300	5.2304480

60

M06L_HS_Ni_III_LD_MeCNMeOH_G1.log

60

Final Energy = -2826.2880738900

Ni	1.0101970	0.1207740	-0.2807870

N	2.1158670	1.7294830	0.5686950
N	2.2698830	-1.5302810	-0.7580010
C	1.1722680	2.8339650	0.9331070
C	3.1882710	2.3066770	-0.2875900
H	2.5185640	1.3362400	1.4098030
N	-0.4573680	-1.1849980	-0.6146390
N	-0.5465540	1.2850360	0.1748800
C	1.3931380	-2.7287920	-0.9502290
C	3.3870100	-1.9170750	0.1494260
H	2.6475050	-1.2603730	-1.6577100
C	1.7229990	4.0815510	0.2392110
C	-0.2956610	2.5653750	0.5765770
H	1.1709210	2.9586940	2.0181010
C	2.5345190	3.5154120	-0.9147940
H	3.5358050	1.5639240	-1.0036660
H	4.0350220	2.6050960	0.3344780
C	-1.7046740	-0.6986800	-0.3989550
C	-0.1093800	-2.4743080	-0.9071150
C	-1.7537770	0.6902660	0.0355380
C	1.8451550	-3.7107360	0.1307930
H	1.6005350	-3.1649590	-1.9309300
H	4.3253830	-1.5229750	-0.2342910
H	3.2140820	-1.4682790	1.1284490
H	0.9193490	4.7491090	-0.0620840
H	2.3723320	4.6312450	0.9201580
O	-1.1079630	3.4705920	0.6924060
H	1.8820130	3.2073280	-1.7343800
H	3.2597440	4.2176450	-1.3182360
C	-2.9112510	-1.3999400	-0.5437040
O	-0.8698920	-3.4087450	-1.1151670
C	-3.0047910	1.2695030	0.3038640
H	1.3452160	-3.4816420	1.0749800
H	1.5992580	-4.7361000	-0.1314830
C	-4.1257570	-0.8027670	-0.2838100
H	-2.8726030	-2.4272190	-0.8705450
C	-4.1734570	0.5579890	0.1523530
H	-3.0352780	2.2968100	0.6327700
C	-5.3907590	-1.5683700	-0.4520530
C	-5.4870880	1.1954470	0.4408910
H	-5.9574580	-1.6070100	0.4801420

H	-5.2017800	-2.5871350	-0.7786900
H	-6.0470690	-1.0913170	-1.1825670
H	-6.0238130	0.6580670	1.2250870
H	-5.3696200	2.2285340	0.7560020
H	-6.1375640	1.1766480	-0.4354790
C	3.3328920	-3.4307910	0.2366220
H	3.7793650	-3.8123910	1.1522000
H	3.8616000	-3.8818820	-0.6048160
N	0.9586760	-0.6230890	1.8292210
C	0.6734620	-0.9986490	2.8796830
C	0.3214130	-1.4778490	4.1850070
H	-0.6891850	-1.1628500	4.4364890
H	0.3678180	-2.5648720	4.2040790
H	1.0107200	-1.0813610	4.9273180
O	1.2752580	0.6840540	-2.0750960
C	0.2324350	1.1083680	-2.8673740
H	-0.3693690	1.8854140	-2.3740190
H	0.5914790	1.4796250	-3.8304060
H	-0.4630560	0.2744740	-3.0516430

60

M06L_HS_Ni_III_LD_MeCNMeOH_G2.log

60

Final Energy = -2826.2881675300

Ni	1.0515050	0.0723740	-0.2457100
N	2.2413520	1.6667850	0.5286340
N	2.2182480	-1.6778030	-0.6274600
C	1.3436460	2.8183750	0.8651200
C	3.3203780	2.1734610	-0.3633380
H	2.6441400	1.2959800	1.3795630
N	-0.4781910	-1.1569500	-0.5984280
N	-0.4451750	1.3329310	0.1321670
C	1.2903720	-2.7324500	-1.1504500
C	3.0530390	-2.2949440	0.4438870
H	2.8179600	-1.3743080	-1.3838910
C	1.9447500	4.0236730	0.1400380
C	-0.1342250	2.6072400	0.5126580
H	1.3473250	2.9696140	1.9467180
C	2.7067410	3.3917310	-1.0129840
H	3.6200240	1.3980990	-1.0659910
H	4.1916800	2.4525550	0.2330080

C	-1.6991400	-0.6091760	-0.3836900
C	-0.1951660	-2.4684290	-0.8607640
C	-1.6810280	0.7922720	0.0088150
C	1.7731810	-4.0335450	-0.5250070
H	1.3635540	-2.7494530	-2.2409950
C	2.3226040	-3.5687950	0.8135840
H	4.0431260	-2.5206250	0.0450430
H	3.1758230	-1.6003010	1.2728810
H	1.1715640	4.7256290	-0.1625770
H	2.6327780	4.5517840	0.7999610
O	-0.9056950	3.5494440	0.6126210
H	2.0223370	3.0885440	-1.8086020
H	3.4525350	4.0497750	-1.4520160
C	-2.9387980	-1.2542050	-0.5144390
O	-0.9955180	-3.3889340	-0.9257150
C	-2.9025530	1.4397890	0.2531800
H	0.9669940	-4.7587180	-0.4506280
H	2.5677750	-4.4691810	-1.1309110
H	2.9734950	-4.2946740	1.2946200
H	1.5029360	-3.3520820	1.5030790

C	-4.1235870	-0.5905270	-0.2808710
H	-2.9501400	-2.2921730	-0.8079020
C	-4.1049950	0.7821880	0.1162570
H	-2.8844310	2.4774230	0.5482320
C	-5.4244880	-1.2962570	-0.4374010
C	-5.3861510	1.4928890	0.3786690
H	-5.9992480	-1.2787670	0.4904450
H	-5.2847340	-2.3325230	-0.7323410
H	-6.0504660	-0.8089130	-1.1874990
H	-5.9517330	1.0073090	1.1763170
H	-5.2185160	2.5281280	0.6625170
H	-6.0338250	1.4792770	-0.4998980
N	0.9139010	-0.5511540	1.8816860
C	0.6193390	-0.8701500	2.9478560
C	0.2571440	-1.2836130	4.2726920
H	0.3094290	-2.3680550	4.3467930
H	0.9375450	-0.8462320	5.0000740
H	-0.7573620	-0.9623350	4.4989250
O	1.4058370	0.5567500	-2.0524180
C	0.3819070	0.8451070	-2.9239480

H -0.3048020 1.5974820 -2.5081300

H 0.7635890 1.1889410 -3.8884280

H -0.2372450 -0.0512060 -3.0897710

60

M06L_HS_Ni_III_LD_MeCNMeOH_G3.log

60

Final Energy = -2826.2813170600

Ni -1.0221490 0.0789250 -0.2937490

N -2.0015790 1.9686420 -0.3068150

N -2.2398680 -1.5980970 0.2262610

C -2.6021820 2.6036110 0.8860640

H -2.7343560 1.8678260 -1.0010560

N 0.3991000 -1.3636940 -0.2422750

N 0.6176470 1.2114640 -0.4883620

C -3.6528250 -1.9281230 -0.0424950

H -2.0963990 -1.6696570 1.2322580

C -1.3747660 4.3162860 -0.2010650

C 0.4434840 2.5645950 -0.6064770

C -2.6985530 4.0688500 0.5134920

H -1.9240390 2.4608350 1.7277620

H -3.5456740 2.1186950 1.1263780

C 1.6647910 -0.8942070 -0.1780680

C -0.0226830 -2.6683130 -0.1512230

C 1.7889850 0.5501500 -0.3183160

C -2.2960310 -3.9638410 0.0325860

H -4.2996230 -1.3793460 0.6374730

H -3.8763490 -1.6000580 -1.0577170

H -0.5953450 4.5978910 0.5068980

H -1.4311330 5.1111210 -0.9401830

O 1.3142120 3.4206380 -0.6006140

H -2.8423400 4.7180020 1.3746460

H -3.5417060 4.2258620 -0.1614690

C 2.8288180 -1.6601770 -0.0068340

O 0.6571530 -3.6626500 0.0394820

C 3.0715170 1.1177850 -0.2749210

H -2.1591470 -4.8003630 -0.6482830

H -1.9479360 -4.2899570 1.0135910

C 4.0759310 -1.0755330 0.0278690

H 2.7279460 -2.7297800 0.0977020

C 4.1989570 0.3412630 -0.1086390

H	3.1642650	2.1875130	-0.3779240
C	5.2949240	-1.9100380	0.2079600
C	5.5456650	0.9732730	-0.0687730
H	5.9797440	-1.7961290	-0.6345680
H	5.0479060	-2.9635210	0.3049470
H	5.8557270	-1.6068130	1.0940360
H	6.1931960	0.5747380	-0.8520550
H	5.4857530	2.0510350	-0.1918880
H	6.0529830	0.7649490	0.8752150
C	-3.7504880	-3.4590080	0.0892880
H	-4.3566430	-3.8770140	-0.7116670
H	-4.2263380	-3.7428450	1.0256850
C	-1.5193090	-2.7372290	-0.3998050
H	-1.6468790	-2.5906870	-1.4812240
C	-1.0157570	2.9631340	-0.8360620
H	-1.1315970	2.9901050	-1.9231440
N	-0.7321600	0.2450690	1.8982190
C	-0.3801390	0.2525010	2.9942600
C	0.0557100	0.2673700	4.3607510
H	0.6653630	-0.6100940	4.5657340
H	0.6469940	1.1604270	4.5516510
H	-0.8043740	0.2638470	5.0267830
O	-1.5085350	-0.0786520	-2.1302130
C	-0.5412310	-0.2783210	-3.0866210
H	0.0698120	-1.1647360	-2.8552420
H	-0.9764970	-0.3846100	-4.0828950
H	0.1733370	0.5603290	-3.0927360

57

M06L_HS_Ni_III_LD_MeCNOH_G1.log

57

Final Energy = -2786.9703522500

Ni	-1.0617600	-0.1163750	-0.3586410
N	-2.1388270	-1.9106250	0.0742300
N	-2.2628760	1.6492380	-0.3010870
C	-1.1513280	-2.9891640	0.3945680
C	-3.0479490	-2.4252880	-0.9862170
H	-2.6716220	-1.6897710	0.9056190
N	0.3985940	1.2799190	-0.5266190
N	0.5313720	-1.2722940	-0.0722700
C	-1.4742210	2.7494470	-0.9273850

C	-2.8441230	2.2343670	0.9262440
H	-3.0257070	1.4138210	-0.9275520
C	-1.5333790	-4.1656280	-0.5063600
C	0.3146820	-2.5920440	0.2122580
H	-1.2376580	-3.2573210	1.4495870
C	-2.2256210	-3.4878670	-1.6766870
H	-3.3563210	-1.6107720	-1.6370190
H	-3.9374260	-2.8629930	-0.5276310
C	1.6494500	0.8011700	-0.3659190
C	0.0346040	2.5829100	-0.7302400
C	1.7267070	-0.6348500	-0.1310640
C	-2.0171850	4.0634300	-0.3422630
H	-1.6315130	2.6938680	-2.0077810
H	-3.6661720	1.6164650	1.2792890
H	-2.0714860	2.2459660	1.6947500
H	-0.6616140	-4.7562140	-0.7769420
H	-2.2308030	-4.8215120	0.0147020
O	1.1776410	-3.4466640	0.3451560
H	-1.4939540	-3.0170230	-2.3368960
H	-2.8316600	-4.1650800	-2.2734340
C	2.8434730	1.5441400	-0.4090330
O	0.7714510	3.5525390	-0.8030360
C	2.9910980	-1.2209060	0.0180190
H	-1.2443120	4.5317390	0.2662940
H	-2.2811170	4.7744940	-1.1205080
C	4.0702780	0.9436510	-0.2566770
H	2.7766810	2.6093430	-0.5662580
C	4.1456350	-0.4681600	-0.0385250
H	3.0497290	-2.2863850	0.1731050
C	5.3194000	1.7507500	-0.3163300
C	5.4721620	-1.1188460	0.1310740
H	5.8977420	1.6550350	0.6043920
H	5.1067660	2.8039240	-0.4771790
H	5.9737050	1.4081940	-1.1202430
H	6.0122860	-0.6985120	0.9816890
H	5.3770120	-2.1901770	0.2834040
H	6.1082970	-0.9508940	-0.7400990
C	-3.2117470	3.6434990	0.5095020
H	-3.3791480	4.3031830	1.3580180
H	-4.1280280	3.6286280	-0.0825420

N -0.9910540 0.0112990 1.8846980
C -0.7847440 -0.0782100 3.0138110
C -0.5326880 -0.1829350 4.4223200
H 0.0281320 -1.0909170 4.6336900
H 0.0443140 0.6749800 4.7608600
H -1.4734110 -0.2132160 4.9678210
O -1.4103580 -0.3247170 -2.1770700
H -0.8607940 0.2608540 -2.7168100
57
M06L_HS_Ni_III_LD_MeCNOH_G3.log
57
Final Energy = -2786.9638953500
Ni -1.0561120 0.1477610 -0.4350900
N -1.8180490 2.1364000 -0.2681750
N -2.3943130 -1.4604990 0.0161230
C -2.1978700 2.7897410 1.0028270
H -2.6397980 2.1053150 -0.8627470
N 0.2806730 -1.3633790 -0.3220640
N 0.6815590 1.1846950 -0.6007810
C -3.8052190 -1.7052000 -0.3395730
H -2.3121130 -1.5518580 1.0269800
C -1.0326820 4.4319600 -0.2579730
C 0.6134590 2.5407260 -0.7645380
C -2.2518810 4.2605910 0.6441580
H -1.4102340 2.5967090 1.7309380
H -3.1234990 2.3612480 1.3794380
C 1.5743470 -0.9803090 -0.2600710
C -0.2256950 -2.6407290 -0.2686090
C 1.7999960 0.4509910 -0.4110970
C -2.5703600 -3.8152890 -0.2194700
H -4.4584820 -1.1357230 0.3167360
H -3.9505350 -1.3435640 -1.3572220
H -0.1481590 4.6968190 0.3203080
H -1.1659880 5.2090670 -1.0058170
O 1.5474310 3.3210140 -0.8528070
H -2.2258520 4.9079190 1.5178880
H -3.1731120 4.4763780 0.1011370
C 2.6825120 -1.8238190 -0.0938220
O 0.3979080 -3.6702200 -0.0733890
C 3.1197760 0.9322590 -0.3480460

H	-2.4360660	-4.6527490	-0.8996250
H	-2.3045380	-4.1666210	0.7785120
C	3.9664120	-1.3240210	-0.0501930
H	2.5108280	-2.8848140	0.0001810
C	4.1887520	0.0835500	-0.1765440
H	3.2826610	1.9945760	-0.4408620
C	5.1226160	-2.2408270	0.1369100
C	5.5760400	0.6196160	-0.1209490
H	5.8236230	-2.1659650	-0.6967000
H	4.8040380	-3.2758690	0.2225400
H	5.6920480	-1.9821320	1.0316010
H	6.1985080	0.1889690	-0.9075230
H	5.5914880	1.7005340	-0.2286210
H	6.0623300	0.3628490	0.8220430
C	-3.9953430	-3.2309040	-0.2501740
H	-4.5650050	-3.5984050	-1.1011660
H	-4.5507830	-3.5068260	0.6437710
C	-1.7061370	-2.6276200	-0.5888040
H	-1.7650990	-2.4549060	-1.6718350
C	-0.8238590	3.0500540	-0.8993270
H	-1.0250340	3.0675120	-1.9734600
N	-0.7807830	0.1845470	1.7868220
C	-0.4563710	0.0610580	2.8845230
C	-0.0536860	-0.0864910	4.2533080
H	0.4144040	-1.0575240	4.4001700
H	0.6588080	0.6924790	4.5161750
H	-0.9194720	-0.0086420	4.9072790
O	-1.5992540	0.0425900	-2.2102630
H	-0.9949440	0.4964800	-2.8146210

55

M06L_HS_Ni_II_LD_ClMeOH_G1.log

55

Final Energy = -3153.9390854400

Ni	-0.9328930	0.0645530	0.1568240
N	-2.0357270	1.7261290	-0.5611860
N	-2.2569820	-1.5381730	0.6410340
C	-1.1000030	2.8535020	-0.8552660
C	-3.1184190	2.2296450	0.3112070
H	-2.3899780	1.3288870	-1.4246590
N	0.4602250	-1.3270310	0.2954110

N	0.6250280	1.2372540	-0.2866550
C	-1.4515140	-2.7972100	0.6138830
C	-3.5194380	-1.7737090	-0.1081370
H	-2.4635100	-1.3015610	1.6037090
C	-1.6426370	4.0387530	-0.0486030
C	0.3639660	2.5433890	-0.5477670
H	-1.1469170	3.0751410	-1.9283340
C	-2.4746080	3.3849680	1.0436380
H	-3.4751780	1.4348030	0.9660830
H	-3.9610900	2.5770400	-0.2941090
C	1.7247530	-0.8563050	0.1412340
C	0.0490380	-2.6082240	0.4629370
C	1.8093050	0.6154410	-0.1726510
C	-2.0835470	-3.6157880	-0.5103310
H	-1.6075700	-3.3364550	1.5565540
H	-4.3694700	-1.4155530	0.4706350
H	-3.4773450	-1.2048560	-1.0383770
H	-0.8286170	4.6591290	0.3209610
H	-2.2784580	4.6668750	-0.6747830
O	1.2004480	3.4698860	-0.6471300
H	-1.8347660	3.0019950	1.8402060
H	-3.2039040	4.0546330	1.4945090
C	2.9181330	-1.5776260	0.2481540
O	0.7782920	-3.6159870	0.5169370
C	3.0980880	1.1796290	-0.3411880
H	-1.6886460	-3.2752120	-1.4695200
H	-1.8685540	-4.6767890	-0.4073620
C	4.1600300	-0.9916400	0.0833820
H	2.8516590	-2.6324190	0.4778980
C	4.2463520	0.4458820	-0.2194570
H	3.1527910	2.2338120	-0.5791270
C	5.4057930	-1.7975720	0.2000760
C	5.5845150	1.0759980	-0.3963000
H	5.9980360	-1.7824790	-0.7205540
H	5.1819780	-2.8378310	0.4291930
H	6.0722450	-1.4220450	0.9832240
H	6.1561380	0.6105010	-1.2047900
H	5.4939320	2.1366480	-0.6228710
H	6.2070500	0.9793550	0.4983460
C	-3.5561380	-3.2659870	-0.3930810

H	-4.1313410	-3.5054660	-1.2858820
H	-4.0063160	-3.8051340	0.4433340
O	-1.0884580	0.5165360	2.0034430
C	-0.0261840	1.0873630	2.6712680
H	0.3028560	2.0284380	2.2026180
H	-0.2814730	1.2936030	3.7170230
H	0.8554970	0.4268680	2.6568860
Cl	-1.1842950	-0.5760710	-2.4180060

55

M06L_HS_Ni_II_LD_ClMeOH_G2.log

55

Final Energy = -3153.9382174500

Ni	0.9698560	-0.0341410	0.1221860
N	2.1258030	-1.6666500	-0.6016640
N	2.2287260	1.6439740	0.5231080
C	1.2432790	-2.8579440	-0.7753140
C	3.3031890	-2.0717730	0.1912680
H	2.3817980	-1.2826030	-1.5052980
N	-0.4880870	1.2815620	0.3067560
N	-0.5397120	-1.2972100	-0.2371230
C	1.3317980	2.7903710	0.8839170
C	3.2344080	2.1323640	-0.4547940
H	2.6923180	1.3204800	1.3629350
C	1.8660180	-3.9491060	0.1087110
C	-0.2287540	-2.5966750	-0.4648000
H	1.2760740	-3.1700290	-1.8260490
C	2.7736490	-3.1873080	1.0634370
H	3.6951060	-1.2203910	0.7482090
H	4.0968030	-2.4394370	-0.4668460
C	-1.7260260	0.7571180	0.1442290
C	-0.1332280	2.5789030	0.5028090
C	-1.7515610	-0.7208630	-0.1442500
C	1.9487260	4.0079380	0.2027420
H	1.3473400	2.9056520	1.9739730
C	2.6337350	3.4054230	-1.0120130
H	4.1779430	2.3404900	0.0566350
H	3.4121540	1.3722720	-1.2147910
H	1.0934970	-4.5338620	0.6045320
H	2.4568470	-4.6369620	-0.4978640
O	-1.0222210	-3.5612030	-0.5151310

H	2.2019070	-2.7640120	1.8898920
H	3.5663720	-3.8006160	1.4866050
C	-2.9534310	1.4310850	0.2351840
O	-0.9073370	3.5561650	0.5142040
C	-3.0106630	-1.3381570	-0.3138490
H	1.1877830	4.7482550	-0.0341740
H	2.6837200	4.4783130	0.8583730
H	3.3798800	4.0570150	-1.4625240
H	1.8960570	3.1514150	-1.7758150
C	-4.1652720	0.7932320	0.0728280
H	-2.9310280	2.4909940	0.4491530
C	-4.1923500	-0.6499120	-0.2111700
H	-3.0216510	-2.3971780	-0.5358640
C	-5.4446890	1.5472560	0.1743380
C	-5.5014290	-1.3375160	-0.3892560
H	-6.0317390	1.4928340	-0.7478380
H	-5.2666230	2.5991550	0.3896410
H	-6.0963670	1.1532160	0.9605580
H	-6.0848160	-0.9104430	-1.2106750
H	-5.3646960	-2.3970630	-0.5971180
H	-6.1364300	-1.2528620	0.4979560
O	1.2371080	-0.4272270	1.9709260
C	0.2302310	-1.0278260	2.6955430
H	-0.0672540	-1.9981230	2.2663950
H	0.5331540	-1.1894220	3.7364170
H	-0.6837220	-0.4128120	2.6941620
Cl	1.0562540	0.5140220	-2.4868640

55

M06L_HS_Ni_II_LD_ClMeOH_G3.log

55

Final Energy = -3153.9332677000

Ni	0.9074500	0.0748390	0.3421190
N	2.0364740	1.8512370	0.6252190
N	2.1041220	-1.5401910	-0.2905220
C	3.2350020	2.1494220	-0.2008870
H	2.3038000	1.7364790	1.5947170
N	-0.5307120	-1.3290880	0.0476000
N	-0.6414970	1.2938830	0.3420710
C	3.5187800	-1.8900420	-0.1017790
H	1.9210600	-1.4365440	-1.2903670

C	1.6075620	3.8016130	-0.6776760
C	-0.3801600	2.6247980	0.3930710
C	3.1114370	3.6144420	-0.5821010
H	3.2027780	1.5153580	-1.0880160
H	4.1392490	1.9097690	0.3566100
C	-1.7650720	-0.8128840	-0.0144030
C	-0.1132070	-2.6247210	-0.0861510
C	-1.8423460	0.6815230	0.1478320
C	2.1479930	-3.8904680	-0.4307660
H	4.1406580	-1.2863970	-0.7601750
H	3.7950550	-1.6509090	0.9263540
H	1.2283770	3.3597870	-1.6014440
H	1.2851620	4.8393440	-0.6385200
O	-1.2174190	3.5425640	0.3525160
H	3.6369140	3.8514350	-1.5056800
H	3.5218280	4.2519410	0.2042210
C	-2.9787720	-1.5166230	-0.2369160
O	-0.8241270	-3.6345520	-0.2983570
C	-3.0976820	1.2859700	0.0828840
H	1.9875760	-4.8325410	0.0891980
H	1.8221390	-4.0333580	-1.4630770
C	-4.1933260	-0.8927800	-0.2892190
H	-2.9133670	-2.5885830	-0.3693010
C	-4.2656130	0.5649520	-0.1219100
H	-3.1505350	2.3585480	0.2082150
C	-5.4473670	-1.6645500	-0.5148910
C	-5.5838500	1.2524820	-0.1814940
H	-6.1570230	-1.5457990	0.3092990
H	-5.2423190	-2.7276280	-0.6251500
H	-5.9785170	-1.3325280	-1.4117710
H	-6.2787000	0.8857790	0.5810330
H	-5.4754820	2.3260370	-0.0386710
H	-6.0914720	1.0963930	-1.1389250
C	3.6077520	-3.4077470	-0.3675420
H	4.1591350	-3.9037810	0.4297960
H	4.1381620	-3.6198210	-1.2940780
C	1.3721040	-2.7395320	0.1835020
H	1.5358940	-2.7565110	1.2735820
C	1.0880560	2.9999920	0.5160940
H	1.1823870	3.6222230	1.4141820

O 1.1796830 -0.2033240 2.2054100
C 0.2025020 -0.7982960 2.9734670
H -0.0916090 -1.7854690 2.5858630
H 0.5330430 -0.9175790 4.0113580
H -0.7207790 -0.1977640 2.9748040
Cl 0.9959660 0.5160540 -2.3180700
52
M06L_HS_Ni_II_LD_ClOH_G1.log
52
Final Energy = -3114.6222826900
Ni 0.9943500 0.1616310 -0.1752790
N 2.0588380 1.9573590 0.2434430
N 2.3218180 -1.5325370 -0.1666880
C 1.0589590 3.0266250 0.5468030
C 2.9886690 2.4699670 -0.7862010
H 2.5413280 1.6993170 1.0958440
N -0.3882600 -1.3027970 -0.3512160
N -0.5975740 1.2856010 0.0940470
C 1.5588630 -2.6655210 -0.7812550
C 3.0424410 -2.1099220 0.9830230
H 3.0088650 -1.2234640 -0.8467400
C 1.4251990 4.1864230 -0.3861040
C -0.3940660 2.5949400 0.3851300
H 1.1637180 3.3278140 1.5934570
C 2.1613870 3.4983790 -1.5238580
H 3.3354500 1.6518240 -1.4152400
H 3.8573760 2.9407950 -0.3163860
C -1.6477190 -0.8639910 -0.2087560
C 0.0577730 -2.5747220 -0.5382000
C -1.7794740 0.6196020 0.0139880
C 2.1826240 -3.9553430 -0.2222170
H 1.7219300 -2.5984310 -1.8648200
H 3.8665120 -1.4593150 1.2694740
H 2.3506260 -2.1573970 1.8237700
H 0.5399920 4.7401580 -0.6912720
H 2.0928230 4.8842130 0.1220270
O -1.2842430 3.4474250 0.5472950
H 1.4565200 2.9887580 -2.1841360
H 2.7641760 4.1742920 -2.1266890
C -2.8398380 -1.6359270 -0.2394820

O	-0.6425990	-3.6089510	-0.6115790
C	-3.0624910	1.1584110	0.1042570
H	1.4777250	-4.4195930	0.4678310
H	2.3872670	-4.6848370	-1.0032040
C	-4.0816130	-1.0786840	-0.1303600
H	-2.7335610	-2.7066460	-0.3527510
C	-4.2078410	0.3775460	0.0361990
H	-3.1545760	2.2286300	0.2258210
C	-5.3114550	-1.9182890	-0.1692320
C	-5.5563770	0.9952660	0.1449360
H	-5.9012230	-1.8267450	0.7477340
H	-5.0663170	-2.9703280	-0.3012530
H	-5.9829460	-1.6277550	-0.9825340
H	-6.1282390	0.5972750	0.9893470
H	-5.4878180	2.0739230	0.2724070
H	-6.1717310	0.8047270	-0.7403910
C	3.4376360	-3.4937730	0.5086140
H	3.7266070	-4.1570020	1.3223640
H	4.2856620	-3.4261780	-0.1765960
O	1.3679150	0.3124640	-2.0278490
H	0.8346930	-0.3460040	-2.4924210
Cl	1.0520550	0.0658530	2.4734360

52

M06L_HS_Ni_II_LD_ClOH_G2.log

52

Final Energy = -3114.6254970600

Ni	0.9747490	0.0495540	-0.2719590
N	2.1098210	1.7062050	0.4673150
N	2.1992810	-1.6569170	-0.6433150
C	1.2235280	2.8903950	0.6459210
C	3.2839980	2.1134200	-0.3336560
H	2.3671820	1.3154610	1.3677610
N	-0.4912360	-1.2663360	-0.3495990
N	-0.5416020	1.3317010	0.0546440
C	1.2945020	-2.8204370	-0.8963050
C	3.2484040	-2.0843850	0.3182080
H	2.6233150	-1.3695370	-1.5165540
C	1.9038400	4.0195200	-0.1332190
C	-0.2212850	2.6453520	0.2157940
H	1.1844070	3.1366160	1.7146640

C	2.7777930	3.2880710	-1.1399170
H	3.6365570	1.2784380	-0.9382840
H	4.0975070	2.4241280	0.3288810
C	-1.7313240	-0.7323220	-0.2036040
C	-0.1519010	-2.5753160	-0.4676200
C	-1.7468310	0.7551170	0.0116730
C	1.9409240	-3.9977980	-0.1748130
H	1.2549530	-2.9986670	-1.9764140
C	2.6832100	-3.3289590	0.9696510
H	4.1719170	-2.3117030	-0.2204830
H	3.4510320	-1.2828530	1.0279760
H	1.1638920	4.6801450	-0.5802530
H	2.5256940	4.6200760	0.5326260
O	-1.0143250	3.6157570	0.1884770
H	2.1816620	2.9272430	-1.9805130
H	3.5829360	3.8987250	-1.5428220
C	-2.9526960	-1.3988670	-0.2760380
O	-0.9278780	-3.5403120	-0.3718460
C	-3.0119530	1.3879880	0.1711570
H	1.1923660	-4.7212440	0.1408530
H	2.6426300	-4.5074400	-0.8371470
H	3.4557020	-3.9537900	1.4134240
H	1.9851000	-3.0378010	1.7566480
C	-4.1702390	-0.7455010	-0.1371220
H	-2.9384330	-2.4654630	-0.4535300
C	-4.1890110	0.7031810	0.0986380
H	-3.0134760	2.4537260	0.3597160
C	-5.4499370	-1.5011010	-0.2074930
C	-5.4967880	1.3970300	0.2684870
H	-6.0362280	-1.4108870	0.7125320
H	-5.2734140	-2.5607180	-0.3819640
H	-6.1028740	-1.1379120	-1.0076170
H	-6.0670930	0.9972230	1.1118530
H	-5.3588450	2.4632220	0.4366840
H	-6.1405390	1.2778480	-0.6075670
O	1.2957280	0.4703570	-2.0868830
H	0.7457130	1.2280030	-2.3262290
Cl	1.0710530	-0.4465690	2.3624050

52

M06L_HS_Ni_II_LD_ClOH_G3.log

52

Final Energy = -3114.6163941700

Ni	-0.9763680	0.0883580	-0.3277500
N	-1.9043310	2.0127490	-0.1631900
N	-2.3048040	-1.5259850	0.0850430
C	-2.4115560	2.6619790	1.0593770
H	-2.6792910	1.9072490	-0.8100280
N	0.3772930	-1.3756990	-0.2211000
N	0.6831740	1.2328530	-0.4269630
C	-3.7109520	-1.8233830	-0.2157860
H	-2.1712230	-1.5364610	1.0976810
C	-1.2711400	4.3557510	-0.1460480
C	0.5143650	2.5748160	-0.5558850
C	-2.5383940	4.1211160	0.6697610
H	-1.6705400	2.5186600	1.8458090
H	-3.3332780	2.1811350	1.3809110
C	1.6541570	-0.9448680	-0.1368570
C	-0.1086390	-2.6507870	-0.1415140
C	1.8245680	0.5478000	-0.2335030
C	-2.4092780	-3.8841810	-0.0171930
H	-4.3630250	-1.2573920	0.4468150
H	-3.9118630	-1.4980670	-1.2374010
H	-0.4435410	4.6639210	0.4926150
H	-1.3912770	5.1291170	-0.9019150
O	1.4118440	3.4411060	-0.5844170
H	-2.6217520	4.7855710	1.5281860
H	-3.4299820	4.2664560	0.0559180
C	2.8046360	-1.7318010	0.0043470
O	0.5594810	-3.6857810	0.0442890
C	3.1325850	1.0699080	-0.1117900
H	-2.2517870	-4.7664440	-0.6339780
H	-2.1278460	-4.1518950	1.0031780
C	4.0716610	-1.1876150	0.0923750
H	2.6784550	-2.8057200	0.0417100
C	4.2374850	0.2727010	0.0393770
H	3.2439410	2.1456210	-0.1398990
C	5.2659730	-2.0589500	0.2574300
C	5.6005250	0.8603580	0.1599410
H	5.9882300	-1.9271420	-0.5543680
H	4.9878110	-3.1106290	0.2850800

H 5.8154050 -1.8362090 1.1773840
H 6.2735950 0.5062090 -0.6266450
H 5.5674160 1.9466010 0.1023500
H 6.0838460 0.5926140 1.1044490
C -3.8530010 -3.3535130 -0.0784770
H -4.3989060 -3.7680700 -0.9245380
H -4.4118840 -3.6219150 0.8163330
C -1.5834620 -2.6947770 -0.4708240
H -1.6829230 -2.5913460 -1.5622940
C -0.9407910 2.9862820 -0.7646220
H -1.1293000 2.9889960 -1.8448200
O -1.4734210 0.0758670 -2.1507820
H -0.8236150 0.5819160 -2.6560410
Cl -0.8278440 0.0236010 2.3435650

49

M06L_HS_Ni_II_LD_G2.log

49

Final Energy = -2578.1915424800

Ni -1.2087860 0.0000850 -0.0000300
N -2.3936860 1.6999490 -0.2926830
N -2.3938900 -1.6996040 0.2927450
C -1.5313940 2.8537160 -0.7233270
C -3.1499820 2.1339010 0.9279060
H -3.0531550 1.4976570 -1.0337590
N 0.2738770 -1.2744780 0.2860380
N 0.2740220 1.2744620 -0.2861360
C -1.5317350 -2.8534230 0.7235150
C -3.1502340 -2.1335990 -0.9278080
H -3.0533520 -1.4971850 1.0337920
C -1.9253840 4.0112590 0.1922520
C -0.0378150 2.5533060 -0.6454930
H -1.7477310 3.0831040 -1.7691370
C -2.3521390 3.3005850 1.4643030
H -3.2524690 1.3065870 1.6290340
H -4.1527320 2.4504710 0.6379050
C 1.4959480 -0.7433030 0.1256550
C -0.0381040 -2.5532870 0.6454310
C 1.4960120 0.7431300 -0.1257850
C -1.9260220 -4.0110760 -0.1917790
H -1.7479670 -3.0825500 1.7694040

C	-2.3526650	-3.3005790	-1.4639680
H	-4.1530730	-2.4498620	-0.6377900
H	-3.2524880	-1.3063870	-1.6290900
H	-1.1019460	4.7092170	0.3221820
H	-2.7663430	4.5590300	-0.2342310
O	0.7816770	3.4447450	-0.9021780
H	-1.4784970	2.9437810	2.0146550
H	-2.9345810	3.9233230	2.1384820
C	2.7339300	-1.4032160	0.1153120
O	0.7812600	-3.4447700	0.9022110
C	2.7341260	1.4029200	-0.1154890
H	-1.1027470	-4.7092470	-0.3215910
H	-2.7670850	-4.5585590	0.2348690
H	-2.9352680	-3.9233320	-2.1379940
H	-1.4789590	-2.9440960	-2.0144270
C	3.9508750	-0.7294340	0.0611050
H	2.7235290	-2.4863450	0.1140900
C	3.9509420	0.7290030	-0.0614380
H	2.7238450	2.4860510	-0.1141710
C	5.2346240	-1.4684740	0.1174710
C	5.2347830	1.4678790	-0.1178800
H	5.8428760	-1.2932830	-0.7746610
H	5.0752580	-2.5394140	0.2122650
H	5.8499320	-1.1432510	0.9610650
H	5.8499760	1.1425630	-0.9615190
H	5.0755620	2.5388420	-0.2126480
H	5.8430650	1.2925730	0.7742070

49

M06L_HS_Ni_II_LD_G3.log

49

Final Energy = -2578.2136717000

Ni	-1.0925300	0.1275610	-0.1932230
N	-2.0595510	1.9547550	-0.3480060
N	-2.3643460	-1.4763010	0.2053330
C	-2.8659160	2.3669710	0.8296630
H	-2.6780750	1.9425550	-1.1510180
N	0.2843680	-1.3325560	-0.0996740
N	0.5453430	1.2384150	-0.3174520
C	-3.7780950	-1.7727170	-0.1153190
H	-2.2810220	-1.5362440	1.2210220

C	-1.4500690	4.2007790	0.3597310
C	0.3976690	2.6001370	-0.3550330
C	-2.8795160	3.8788400	0.7671180
H	-2.3584490	2.0193990	1.7324740
H	-3.8419060	1.8893130	0.7888070
C	1.5434860	-0.8776760	-0.1698480
C	-0.1681550	-2.6293860	-0.0023860
C	1.6862690	0.5309340	-0.2937130
C	-2.4901020	-3.8452190	0.1049820
H	-4.4326470	-1.1845420	0.5224420
H	-3.9525230	-1.4684230	-1.1466520
H	-0.7965720	4.2132310	1.2326830
H	-1.3459080	5.1656870	-0.1290100
O	1.2813920	3.4401000	-0.2421370
H	-3.1673190	4.3394780	1.7095430
H	-3.5864240	4.2122250	0.0052920
C	2.7415930	-1.6942030	-0.1190560
O	0.4750420	-3.6308480	0.2758190
C	3.0278290	1.0775020	-0.3913890
H	-2.3457380	-4.7173670	-0.5275790
H	-2.2054380	-4.1340740	1.1175500
C	4.0267160	-1.0830460	0.1528500
H	2.6510700	-2.7680800	-0.1713710
C	4.1677280	0.2779090	0.0313780
H	3.1734030	2.0797640	-0.7632200
C	5.1162290	-2.0164560	0.5385850
C	5.4238570	1.0311920	0.2708230
H	6.0777610	-1.5353020	0.6809300
H	5.2305590	-2.7942200	-0.2197430
H	4.8587360	-2.5397370	1.4628370
H	5.6928060	1.6048270	-0.6197400
H	5.2777950	1.7704070	1.0626180
H	6.2679620	0.4068870	0.5430550
C	-3.9279270	-3.2946920	0.0584340
H	-4.4972360	-3.7200510	-0.7651840
H	-4.4681540	-3.5347970	0.9717280
C	-1.6489630	-2.6673200	-0.3404390
H	-1.7187490	-2.5654220	-1.4310350
C	-1.0456610	3.0442820	-0.5685220
H	-1.1170820	3.3516020	-1.6142060

61

M06L_HS_Ni_II_LD_MeCN2_G3.log

61

Final Energy = -2843.8536378700

Ni	-0.9445560	0.1036970	0.0123960
N	-1.8872450	2.0177100	-0.1443740
N	-2.1558600	-1.6016360	0.5202740
C	-2.4940400	2.6995530	1.0197680
H	-2.6169210	1.9166750	-0.8418680
N	0.4793320	-1.3695460	-0.0309090
N	0.7060040	1.2048190	-0.3695800
C	-3.5673180	-1.9570430	0.2774410
H	-2.0053680	-1.6519420	1.5269180
C	-1.2283090	4.3612620	-0.0938240
C	0.5635110	2.5644200	-0.4305200
C	-2.5666800	4.1543650	0.6051520
H	-1.8285230	2.5733280	1.8747040
H	-3.4471700	2.2349970	1.2637510
C	1.7195930	-0.9072540	-0.2310700
C	0.0657390	-2.6634950	0.1730530
C	1.8432330	0.4961030	-0.4168140
C	-2.1906410	-3.9733180	0.4105870
H	-4.2141940	-1.3951530	0.9470470
H	-3.8076870	-1.6620460	-0.7445930
H	-0.4544360	4.6426860	0.6203830
H	-1.2582240	5.1414450	-0.8503820
O	1.4516620	3.4045410	-0.3206530
H	-2.7166260	4.8311880	1.4439450
H	-3.3968700	4.3012800	-0.0884210
C	2.9195320	-1.7213930	-0.2855000
O	0.7476280	-3.6323500	0.4905650
C	3.1644530	1.0395040	-0.6713780
H	-2.0480000	-4.8367830	-0.2350170
H	-1.8332970	-4.2542550	1.4023220
C	4.2294440	-1.1063900	-0.2084230
H	2.8256900	-2.7965090	-0.2857200
C	4.3510990	0.2473390	-0.3895670
H	3.2637550	2.0459720	-1.0474190
C	5.3617880	-2.0331720	0.0556890
C	5.6299540	1.0024870	-0.3472690

H	6.3341890	-1.5523750	0.0491810
H	5.3728760	-2.8359850	-0.6850900
H	5.2344280	-2.5249330	1.0234460
H	5.7767400	1.5486560	-1.2825090
H	5.5968930	1.7640610	0.4361100
H	6.5028520	0.3814760	-0.1760260
C	-3.6502690	-3.4842760	0.4541330
H	-4.2519970	-3.9325450	-0.3340530
H	-4.1238150	-3.7463630	1.3982660
C	-1.4293340	-2.7567220	-0.0750300
H	-1.5710870	-2.6515350	-1.1584860
C	-0.8823330	2.9891430	-0.6925230
H	-0.9956330	2.9929060	-1.7801450
N	-1.4389580	-0.1517390	-2.0284440
C	-1.6618320	-0.2545760	-3.1526600
C	-1.9394710	-0.3866700	-4.5537620
H	-1.4959330	0.4427050	-5.1005010
H	-1.5228620	-1.3191510	-4.9286440
H	-3.0142150	-0.3866510	-4.7222860
N	-0.6199540	0.3352820	2.1040420
C	-0.2942510	0.3960330	3.2061770
C	0.1095860	0.4781540	4.5801230
H	0.7559760	-0.3608450	4.8286450
H	0.6527140	1.4049680	4.7523150
H	-0.7639610	0.4551460	5.2279890

57

M06L_HS_Ni_II_LD_MeCNOH_G1.log

57

Final Energy = -2787.0376501500

Ni	-1.0534130	-0.1042040	-0.3855920
N	-2.1663000	-1.8765660	0.0342880
N	-2.2916700	1.6166800	-0.2032100
C	-1.1947420	-2.9678480	0.3671080
C	-3.0571830	-2.3684900	-1.0472840
H	-2.7101610	-1.6509640	0.8572300
N	0.3703570	1.3259050	-0.5082050
N	0.5107320	-1.2751500	-0.1109330
C	-1.5541670	2.7433950	-0.8509690
C	-2.7984340	2.1687130	1.0675570
H	-3.0888490	1.3864090	-0.7886620

C	-1.5566870	-4.1212960	-0.5747420
C	0.2696570	-2.5660020	0.2371890
H	-1.3332120	-3.2659270	1.4107070
C	-2.2190820	-3.4143450	-1.7451680
H	-3.3528750	-1.5407300	-1.6883110
H	-3.9557220	-2.8196240	-0.6178990
C	1.6187670	0.8516560	-0.3809260
C	-0.0432090	2.6121190	-0.6754800
C	1.7088470	-0.6394730	-0.1757560
C	-2.1191590	4.0362400	-0.2353990
H	-1.7716040	2.6876120	-1.9241440
H	-3.5763880	1.5255790	1.4738110
H	-1.9710060	2.1893860	1.7776480
H	-0.6771880	-4.7044080	-0.8383110
H	-2.2685830	-4.7934910	-0.0931470
O	1.1381910	-3.4242230	0.4672140
H	-1.4714320	-2.9230220	-2.3716540
H	-2.8091780	-4.0746260	-2.3767230
C	2.8294170	1.5903680	-0.4019260
O	0.6836970	3.6230330	-0.7647400

C	2.9778870	-1.2139690	-0.0901940
H	-1.3247470	4.5432050	0.3112890
H	-2.4737480	4.7299750	-0.9946580
C	4.0563530	0.9979500	-0.3012080
H	2.7519450	2.6651270	-0.5004130
C	4.1419170	-0.4642350	-0.1495240
H	3.0406160	-2.2883480	0.0123900
C	5.3085590	1.8027470	-0.3377050
C	5.4731780	-1.1186620	-0.0499860
H	5.9014720	1.6822470	0.5736620
H	5.0924930	2.8626730	-0.4551710
H	5.9653780	1.5032700	-1.1595050
H	6.0547540	-0.7447910	0.7984220
H	5.3769320	-2.1964720	0.0640000
H	6.0912410	-0.9313840	-0.9336940
C	-3.2326440	3.5736950	0.7020550
H	-3.3547430	4.2191240	1.5699030
H	-4.1921110	3.5398690	0.1824230
N	-0.9500410	-0.0776160	1.9705920
C	-0.5931230	-0.2579210	3.0514310

C -0.1444990 -0.4856190 4.3969690
H 0.5067410 -1.3567790 4.4300090
H 0.4078800 0.3792320 4.7582370
H -0.9954680 -0.6576930 5.0525160
O -1.4462970 -0.2045930 -2.2221430
H -0.9130810 0.4516050 -2.6900140
49
M06L_HS_Ni_II_Me2_OPD_G2.log
49
Final Energy = -2578.4083973400
Ni -1.1461900 -0.0000030 -0.0000070
N -2.3971750 1.6645120 -0.2633840
N -2.3971720 -1.6645180 0.2633850
C -1.5525610 2.8358280 -0.6814280
C -3.1560240 2.0631790 0.9622980
H -3.0540120 1.4513220 -1.0032290
N 0.2771160 -1.2758220 0.2996670
N 0.2771130 1.2758210 -0.2996700
C -1.5525540 -2.8358320 0.6814290
C -3.1560240 -2.0631890 -0.9622940
H -3.0540070 -1.4513260 1.0032310
C -1.9743850 3.9745760 0.2454200
C -0.0526390 2.5644630 -0.5926300
H -1.7773910 3.0753370 -1.7247480
C -2.3805870 3.2401220 1.5111300
H -3.2335430 1.2241850 1.6528150
H -4.1688610 2.3644950 0.6881570
C 1.5167800 -0.7320630 0.1743370
C -0.0526330 -2.5644610 0.5926400
C 1.5167780 0.7320660 -0.1743390
C -1.9743680 -3.9745790 -0.2454260
H -1.7773890 -3.0753480 1.7247460
C -2.3805780 -3.2401220 -1.5111330
H -4.1688560 -2.3645170 -0.6881480
H -3.2335560 -1.2241930 -1.6528070
H -1.1655930 4.6887500 0.3808640
H -2.8307290 4.5089000 -0.1694100
O 0.7359480 3.5006460 -0.8131700
H -1.4952430 2.8923810 2.0489310
H -2.9710140 3.8408380 2.1989430

C	2.7543270	-1.3766460	0.3248740
O	0.7359560	-3.5006360	0.8132060
C	2.7543230	1.3766510	-0.3248760
H	-1.1655700	-4.6887440	-0.3808750
H	-2.8307070	-4.5089130	0.1694000
H	-2.9710000	-3.8408390	-2.1989480
H	-1.4952370	-2.8923700	-2.0489330
C	3.9525120	-0.7172450	0.1687340
H	2.7493690	-2.4295440	0.5717270
C	3.9525100	0.7172520	-0.1687380
H	2.7493630	2.4295490	-0.5717280
C	5.2477010	-1.4310110	0.3342940
C	5.2476980	1.4310210	-0.3343000
H	5.8584940	-1.3934060	-0.5730260
H	5.0919130	-2.4785500	0.5846060
H	5.8652080	-0.9890120	1.1222500
H	5.8652040	0.9890230	-1.1222570
H	5.0919070	2.4785600	-0.5846110
H	5.8584920	1.3934160	0.5730190

61

M06L_HS_Ni_II_Me2_OPD_MeCN2_G2.log

61

Final Energy = -2844.0407461300

Ni	-0.9952380	0.0000140	0.0000600
N	-2.2044830	-1.6509440	0.6172680
N	-2.2044320	1.6511080	-0.6169350
C	-1.2932650	-2.7256440	1.1433500
C	-3.1128300	-2.2709230	-0.3838820
H	-2.7568050	-1.2927830	1.3856420
N	0.5124860	1.2532260	-0.4315400
N	0.5124380	-1.2533030	0.4315050
C	-1.2931920	2.7256770	-1.1432390
C	-3.1124930	2.2712370	0.3843810
H	-2.7569650	1.2929870	-1.3851760
C	-1.8007360	-4.0172030	0.5035840
C	0.1950110	-2.4920130	0.8699980
H	-1.4038410	-2.7671730	2.2328600
C	-2.3953290	-3.5337660	-0.8078220
H	-3.3051340	-1.5757830	-1.2008470
H	-4.0717080	-2.5174260	0.0791020

C	1.7354930	0.7156670	-0.2430550
C	0.1951130	2.4919100	-0.8701360
C	1.7354610	-0.7158390	0.2428860
C	-1.8004000	4.0173250	-0.5034420
H	-1.4039490	2.7671590	-2.2327300
C	-2.3947520	3.5340110	0.8081190
H	-4.0714370	2.5178370	-0.0784110
H	-3.3047090	1.5761530	1.2014140
H	-0.9947810	-4.7387470	0.3920720
H	-2.5779580	-4.4726100	1.1202990
O	0.9857930	-3.4225020	1.1443290
H	-1.5997950	-3.2947220	-1.5174740
H	-3.0600030	-4.2527640	-1.2821240
C	2.9849270	1.3374280	-0.4535850
O	0.9859280	3.4223200	-1.1446280
C	2.9848720	-1.3377100	0.4532720
H	-0.9943550	4.7388020	-0.3921480
H	-2.5777170	4.4727670	-1.1200120
H	-3.0592370	4.2531030	1.2825430
H	-1.5990860	3.2949000	1.5176000
C	4.1795120	0.6980940	-0.2363200
H	2.9794440	2.3629050	-0.7984820
C	4.1794810	-0.6984770	0.2358730
H	2.9793400	-2.3631880	0.7981650
C	5.4765720	1.3929290	-0.4704460
C	5.4765110	-1.3934160	0.4698580
H	6.0919200	1.4404030	0.4336220
H	5.3192190	2.4135920	-0.8149660
H	6.0927520	0.8830650	-1.2179360
H	6.0928080	-0.8836030	1.2172850
H	5.3191150	-2.4140680	0.8143890
H	6.0917580	-1.4409310	-0.4342760
N	-1.0488470	-0.6306210	-2.0727190
C	-0.8857490	-0.9030710	-3.1799420
C	-0.6839580	-1.2577380	-4.5567030
H	-0.5926810	-2.3378840	-4.6532030
H	-1.5260900	-0.9215860	-5.1577530
H	0.2246430	-0.7934870	-4.9343130
N	-1.0485220	0.6307380	2.0729190
C	-0.8849140	0.9032740	3.1800470

C -0.6825070 1.2580430 4.5566910
H -0.5920060 2.3382550 4.6531800
H -1.5239920 0.9212130 5.1582710
H 0.2266990 0.7944870 4.9336960
61
M06L_HS_Ni_II_Me2_OPD_MeCN2_G3.log
61
Final Energy = -2844.0361823000
Ni 1.0218830 0.1253460 0.0220460
N 1.9104580 2.0221780 -0.2456140
N 2.1957140 -1.4496940 -0.7622180
C 2.1485240 2.5776940 -1.5936760
H 2.8017270 2.0292390 0.2409010
N -0.3138260 -1.3940180 0.2138280
N -0.5987580 1.2293490 0.4457300
C 3.6420970 -1.7133010 -0.8513430
H 1.8115350 -1.4782910 -1.7068640
C 1.2120150 4.3507190 -0.3235480
C -0.4408650 2.5734210 0.4810910
C 2.2961650 4.0671460 -1.3597460
H 1.2707660 2.3606130 -2.2043760
H 3.0060490 2.0895840 -2.0536480
C -1.5841830 -0.9732800 0.3447130
C 0.1562700 -2.6523650 -0.0090550
C -1.7518830 0.5228940 0.4815720
C 2.3856650 -3.8067520 -0.7352460
H 4.0810390 -1.1258520 -1.6551650
H 4.0992940 -1.3861620 0.0837660
H 0.2706970 4.6229850 -0.8002380
H 1.4747420 5.1693340 0.3432760
O -1.3322110 3.4352630 0.6158550
H 2.1823850 4.6566540 -2.2679280
H 3.2882270 4.2795790 -0.9557480
C -2.7541820 -1.7684190 0.3244910
O -0.5008620 -3.7128110 -0.0950200
C -3.0612790 1.0272060 0.6011530
H 2.4145630 -4.7030960 -0.1193420
H 1.8560090 -4.0676900 -1.6535350
C -4.0121080 -1.2374400 0.4348290
H -2.6278740 -2.8373510 0.2128050

C	-4.1739510	0.2197480	0.5890070
H	-3.1784540	2.0974270	0.7059620
C	-5.2200870	-2.1092250	0.4116800
C	-5.5409690	0.7947870	0.7271240
H	-5.8088340	-2.0240580	1.3302620
H	-4.9480040	-3.1560900	0.2900300
H	-5.9041130	-1.8472730	-0.4012240
H	-6.0732490	0.3961830	1.5965470
H	-5.5034580	1.8775760	0.8322110
H	-6.1760980	0.5677120	-0.1349840
C	3.7825120	-3.2372300	-1.0374340
H	4.5357620	-3.6389930	-0.3615080
H	4.1038690	-3.4869980	-2.0470940
C	1.6717470	-2.6530270	-0.0575780
H	2.0535210	-2.5612280	0.9705660
C	1.0206960	3.0220460	0.4322790
H	1.3684340	3.1079210	1.4672550
N	1.8611790	0.0017740	1.9150780
C	2.3022410	-0.0615960	2.9759250
C	2.8502340	-0.1426010	4.2997290
H	2.3579070	0.5760530	4.9514490
H	2.7028350	-1.1420870	4.7032870
H	3.9159860	0.0747150	4.2771910
N	0.1077100	0.1253860	-2.3374180
C	-1.0185970	0.1206400	-2.5977870
C	-2.4246310	0.1110180	-2.8941690
H	-2.9541200	-0.4433710	-2.1177200
H	-2.8118490	1.1281070	-2.9224670
H	-2.6073420	-0.3617770	-3.8574230

57

M06L_HS_Ni_II_Me2_OPD_MeCNOH_G2.log

57

Final Energy = -2787.2059822000

Ni	-1.0935920	0.0969480	-0.4698810
N	-2.3578210	-1.5037300	0.1275690
N	-2.2182320	1.8996240	-0.4029420
C	-1.5226270	-2.7303660	0.3211260
C	-3.4328920	-1.8268410	-0.8308110
H	-2.7353920	-1.2211760	1.0250540
N	0.4617780	1.3636490	-0.4806910

N	0.3527130	-1.2513720	-0.1456130
C	-1.2876140	3.0551450	-0.5779010
C	-3.1109190	2.1841240	0.7344560
H	-2.7194360	1.7117370	-1.2642970
C	-2.0158440	-3.7150920	-0.7567270
C	-0.0195730	-2.4840960	0.2667200
H	-1.7201350	-3.1481860	1.3143920
C	-2.7750430	-2.8390010	-1.7413430
H	-3.7538240	-0.9218850	-1.3463720
H	-4.2980270	-2.2643660	-0.3200030
C	1.6665210	0.7509330	-0.4358800
C	0.1960080	2.6823300	-0.6007000
C	1.6029660	-0.7461740	-0.2314460
C	-1.6181630	3.9957800	0.5909870
H	-1.4984200	3.5536280	-1.5302890
C	-2.2605840	3.0733390	1.6143670
H	-4.0123550	2.7169060	0.4128870
H	-3.4245160	1.2559880	1.2148140
H	-1.1885180	-4.2649890	-1.2019800
H	-2.6899650	-4.4506500	-0.3136300
O	0.7334130	-3.4221240	0.6231110
H	-2.0909150	-2.3079730	-2.4052820
H	-3.4901110	-3.3915360	-2.3481090
C	2.9395920	1.3533510	-0.4916780
O	1.0189030	3.6145140	-0.7453890
C	2.8231800	-1.4404200	-0.0917890
H	-0.7288190	4.5119930	0.9462050
H	-2.3370720	4.7558030	0.2789330
H	-2.8350280	3.5948890	2.3770390
H	-1.5000890	2.4715750	2.1192840
C	4.1074350	0.6396560	-0.3734040
H	2.9789070	2.4247060	-0.6385790
C	4.0453570	-0.8190670	-0.1641730
H	2.7759840	-2.5096520	0.0671550
C	5.4333990	1.3134080	-0.4583900
C	5.3106820	-1.5962350	-0.0435580
H	6.0343950	1.1668950	0.4449910
H	5.3201380	2.3860180	-0.6072660
H	6.0445630	0.9292930	-1.2817840
H	5.9292680	-1.2572190	0.7936650

H 5.1090450 -2.6558180 0.1048590
H 5.9434940 -1.5016790 -0.9320030
N -1.5789040 -0.5477860 2.9518290
C -0.4299000 -0.6802040 2.9582890
C 1.0001830 -0.8378230 2.9907210
H 1.4814110 0.0196850 2.5213630
H 1.3461190 -0.9182470 4.0194000
H 1.2913430 -1.7340150 2.4385870
O -1.5281580 0.0477790 -2.4200170
H -0.9344610 0.6931970 -2.8182680
55
M06L_LS_Ni_III_LD_ClMeOH_G1.log
55
Final Energy = -3153.9057565000
Ni -0.8843210 0.0427540 -0.0040230
N -2.0716560 1.6323990 -0.3339990
N -2.2269320 -1.3395090 0.5608580
C -1.2069940 2.7731600 -0.7877070
C -2.9134440 2.1163460 0.7964210
H -2.6437510 1.3157330 -1.1101510
N 0.3931710 -1.3088870 0.1714720
N 0.5458610 1.1705180 -0.3975130
C -1.5369670 -2.6683440 0.5950530
C -3.5297260 -1.5071560 -0.1482630
H -2.3593210 -1.0089140 1.5127510
C -1.6593490 3.9732810 0.0320010
C 0.2774950 2.4680160 -0.6290300
H -1.3663460 2.9185680 -1.8573090
C -2.1871960 3.3397020 1.3086460
H -3.0305650 1.3334340 1.5417070
H -3.8983710 2.3721710 0.4019690
C 1.6997120 -0.8342650 0.0381160
C -0.0355790 -2.5584210 0.4252600
C 1.7845240 0.5380590 -0.2639260
C -2.2408210 -3.4789880 -0.4841360
H -1.7237010 -3.1263960 1.5700090
H -4.3233830 -1.0574400 0.4439720
H -3.4706080 -0.9845740 -1.1021900
H -0.8353690 4.6667240 0.1854260
H -2.4552640 4.5065900 -0.4873800

O	1.1160080	3.3521550	-0.7774200
H	-1.3635090	3.0334110	1.9555120
H	-2.8387330	3.9960160	1.8800520
C	2.8671660	-1.5705630	0.2246740
O	0.6759300	-3.5571380	0.4990150
C	3.0356270	1.1396290	-0.3778150
H	-1.8310930	-3.2238610	-1.4630110
H	-2.1137200	-4.5469950	-0.3295030
C	4.1146430	-0.9709410	0.0931000
H	2.7912080	-2.6209010	0.4618250
C	4.1995420	0.3971370	-0.2117170
H	3.0914560	2.1931180	-0.6051710
C	5.3565760	-1.7828420	0.2743600
C	5.5328990	1.0572490	-0.3564040
H	5.9749490	-1.7793770	-0.6257850
H	5.1251590	-2.8177680	0.5155250
H	5.9842530	-1.3846760	1.0743710
H	6.1289440	0.5923560	-1.1445470
H	5.4314270	2.1136150	-0.5942820
H	6.1240600	0.9756180	0.5582380
C	-3.6788110	-3.0038900	-0.3641070
H	-4.2817170	-3.2301290	-1.2403770
H	-4.1585440	-3.4686240	0.4983770
O	-0.8633540	0.4568330	1.8302710
C	0.3175600	0.6368590	2.5274950
H	0.8992040	1.5078800	2.1980850
H	0.0292030	0.8174500	3.5709300
H	0.9741930	-0.2421110	2.5277220
Cl	-1.1877640	-0.4428610	-2.2821720

55

M06L_LS_Ni_III_LD_ClMeOH_G2.log

55

Final Energy = -3153.9049087500

Ni	-0.9204370	0.0351000	-0.0385320
N	-2.1433720	1.6082570	-0.3257610
N	-2.2468180	-1.4010640	0.4499640
C	-1.3024020	2.7886410	-0.7187200
C	-3.0171380	2.0263740	0.8054120
H	-2.6912370	1.3079430	-1.1252760
N	0.3875240	-1.2847920	0.1184800

N	0.4841940	1.2187030	-0.3537720	O	1.0049790	3.4210070	-0.6802240
C	-1.4751900	-2.6238970	0.8553880	H	-1.5028140	2.9187200	2.0291140
C	-3.3291630	-1.8311070	-0.4789750	H	-2.9974580	3.8537850	1.9777830
H	-2.6292260	-0.9677050	1.2841550	C	2.8690300	-1.4932970	0.1396040
C	-1.7931170	3.9407670	0.1472660	O	0.7430940	-3.4866860	0.6231450
C	0.1867130	2.5138760	-0.5570330	C	2.9728240	1.2468920	-0.3237590
H	-1.4537000	2.9774860	-1.7826360	H	-1.4589550	-4.5727710	-0.0817860
C	-2.3244070	3.2385380	1.3860000	H	-2.9250740	-4.2217110	0.8268500
H	-3.1371690	1.2102760	1.5141070	H	-3.6375290	-3.7400670	-1.4647970
H	-3.9983160	2.2829480	0.4023690	H	-2.1011950	-2.9526800	-1.8262890
C	1.6835200	-0.7762480	-0.0059930	C	4.1017470	-0.8583740	0.0383850
C	0.0032510	-2.5143830	0.5073660	H	2.8192540	-2.5556600	0.3228430
C	1.7364840	0.6099880	-0.2447970	C	4.1541820	0.5253620	-0.1947690
C	-2.1732410	-3.7907100	0.1661900	H	3.0025440	2.3115930	-0.4974610
H	-1.5131690	-2.7125330	1.9423920	C	5.3625130	-1.6495440	0.1764520
C	-2.8337250	-3.1437750	-1.0404760	C	5.4713030	1.2230220	-0.3075610
H	-4.2420130	-1.9698640	0.1035670	H	5.9843800	-1.5761640	-0.7182350
H	-3.5096490	-1.0641730	-1.2284930	H	5.1554200	-2.7025260	0.3529830
H	-0.9873510	4.6447650	0.3435470	H	5.9770220	-1.2863600	1.0029820
H	-2.5937110	4.4793680	-0.3593130	H	6.0693700	0.8240150	-1.1296410

H 5.3445960 2.2900860 -0.4752450
H 6.0730520 1.0945950 0.5946400
O -0.9565290 0.3612780 1.8113680
C 0.2029300 0.5044190 2.5511770
H 0.8224140 1.3578030 2.2455440
H -0.1172470 0.6964840 3.5832950
H 0.8327220 -0.3944540 2.5741620
Cl -1.1466160 -0.3228090 -2.3526990
55
M06L_LS_Ni_III_LD_ClMeOH_G3.log
55
Final Energy = -3153.8953214500
Ni 0.8362600 0.0511330 0.0890810
N 2.0336530 1.5871390 0.5526030
N 2.0852910 -1.4703230 -0.2486850
C 3.2819840 1.8705520 -0.2145740
H 2.2393890 1.2997290 1.5056490
N -0.4836620 -1.2621100 -0.2135720
N -0.5752610 1.2614800 0.2358720
C 3.4668250 -1.7107190 0.2101210
H 2.0712770 -1.4743060 -1.2699640
C 1.7986790 3.7006990 -0.5236070
C -0.2770430 2.5602570 0.4210910
C 3.2791170 3.3729180 -0.4367560
H 3.2275020 1.3377130 -1.1628190
H 4.1380870 1.4994190 0.3438540
C -1.7717850 -0.7242560 -0.2033260
C -0.1059470 -2.5595440 -0.1489090
C -1.8226720 0.6596900 0.0512860
C 2.2416770 -3.8100590 -0.1770030
H 4.1645920 -1.1283750 -0.3851690
H 3.5339770 -1.3791390 1.2462670
H 1.3935540 3.3953850 -1.4902380
H 1.5675960 4.7519480 -0.3751960
O -1.0885720 3.4818700 0.4439950
H 3.8356090 3.6534900 -1.3278710
H 3.7305250 3.8863010 0.4134580
C -2.9558220 -1.4387210 -0.3661180
O -0.8341850 -3.5349610 -0.2999380
C -3.0604750 1.2913680 0.1482370

H	2.0402870	-4.7159970	0.3882830
H	2.0925630	-4.0420210	-1.2321530
C	-4.1886530	-0.8002940	-0.2906340
H	-2.9029110	-2.4992970	-0.5610820
C	-4.2408520	0.5784480	-0.0303020
H	-3.0913060	2.3507270	0.3524560
C	-5.4475630	-1.5810220	-0.4903940
C	-5.5569330	1.2828960	0.0485980
H	-6.1020870	-1.5194530	0.3815550
H	-5.2396880	-2.6319730	-0.6776460
H	-6.0285080	-1.1997160	-1.3328370
H	-6.1962240	0.8551980	0.8237820
H	-5.4307350	2.3409420	0.2662570
H	-6.1159800	1.1983400	-0.8857420
C	3.6511120	-3.2385630	0.0809970
H	4.0820550	-3.6425230	0.9943030
H	4.3328880	-3.4837080	-0.7296420
C	1.3518250	-2.6609190	0.2374540
H	1.3684000	-2.5749060	1.3304490
C	1.2042400	2.8340220	0.5784390
H	1.3514190	3.3231060	1.5450120
O	0.9358840	-0.2945260	1.9344310
C	-0.2056350	-0.5709920	2.6689540
H	-0.6475860	-1.5524920	2.4508430
H	0.0994940	-0.5766860	3.7227020
H	-0.9888010	0.1908050	2.5734400
Cl	0.9812760	0.5022850	-2.2173510

52

M06L_LS_Ni_III_LD_ClOH_G1.log

52

Final Energy = -3114.5948955900

Ni	0.8789510	0.0622290	-0.0789140
N	2.0640840	1.6533290	0.2115290
N	2.2249750	-1.3367860	-0.5989720
C	1.2022120	2.8000330	0.6571620
C	2.8870370	2.1187470	-0.9433130
H	2.6514240	1.3560240	0.9838390
N	-0.4059580	-1.2841680	-0.2733820
N	-0.5581700	1.2071700	0.2587400
C	1.5277550	-2.6620340	-0.6190720

C	3.5158580	-1.4974840	0.1321870
H	2.3667070	-1.0186800	-1.5537940
C	1.6608260	3.9911370	-0.1710970
C	-0.2829810	2.5009040	0.4917650
H	1.3595220	2.9504320	1.7262170
C	2.1624150	3.3446350	-1.4516020
H	2.9804770	1.3280060	-1.6831590
H	3.8823120	2.3663020	-0.5707180
C	-1.7140410	-0.8014780	-0.1557810
C	0.0242310	-2.5420820	-0.4785100
C	-1.7966980	0.5743610	0.1226730
C	2.2096280	-3.4556950	0.4862790
H	1.7284020	-3.1385690	-1.5824050
H	4.3212020	-1.0650900	-0.4569570
H	3.4467650	-0.9552590	1.0745140
H	0.8439910	4.6946550	-0.3165300
H	2.4702980	4.5149450	0.3370560
O	-1.1181300	3.3895460	0.6337800
H	1.3255250	3.0364380	-2.0799100
H	2.8099770	3.9911060	-2.0385200
C	-2.8817260	-1.5423740	-0.3184750
O	-0.6912820	-3.5382630	-0.5410520
C	-3.0471130	1.1773890	0.2365010
H	1.7851700	-3.1807980	1.4533710
H	2.0793050	-4.5256780	0.3493830
C	-4.1289450	-0.9394210	-0.1955370
H	-2.8068720	-2.5975360	-0.5333760
C	-4.2120450	0.4330750	0.0847160
H	-3.1013380	2.2338810	0.4500650
C	-5.3714470	-1.7543180	-0.3608940
C	-5.5444760	1.0976910	0.2197530
H	-5.9923620	-1.7290330	0.5371080
H	-5.1402650	-2.7949270	-0.5766220
H	-5.9965240	-1.3753950	-1.1722440
H	-6.1400740	0.6476430	1.0167730
H	-5.4410800	2.1578960	0.4391110
H	-6.1369740	1.0013020	-0.6925610
C	3.6519830	-2.9906250	0.3802610
H	4.2394020	-3.2025010	1.2704580
H	4.1427660	-3.4747380	-0.4650860

O 0.8257420 0.4481140 -1.8926610

H -0.0562410 0.2088170 -2.2056330

Cl 1.1286970 -0.3740250 2.1771290

52

M06L_LS_Ni_III_LD_ClOH_G2.log

52

Final Energy = -3114.5937731200

Ni	0.9152820	0.0506340	-0.0548870
N	2.1407040	1.6178080	0.2213900
N	2.2413330	-1.4009580	-0.5060640
C	1.3016950	2.8035480	0.6033900
C	3.0132420	2.0221390	-0.9181860
H	2.6921120	1.3282090	1.0222470
N	-0.4020930	-1.2600530	-0.2427910
N	-0.4934180	1.2479820	0.2127110
C	1.4698480	-2.6308480	-0.8902030
C	3.3153310	-1.8122950	0.4406400
H	2.6267930	-0.9835600	-1.3468300
C	1.8091920	3.9499250	-0.2596800
C	-0.1874720	2.5383520	0.4248250
H	1.4424400	2.9924310	1.6686860
C	2.3334130	3.2418820	-1.4980100
H	3.1186350	1.2026870	-1.6248940
H	3.9998640	2.2648780	-0.5203200
C	-1.6982690	-0.7459930	-0.1224190
C	-0.0128740	-2.5062260	-0.5666960
C	-1.7471830	0.6413240	0.1023450
C	2.1552330	-3.7828340	-0.1648900
H	1.5225850	-2.7462380	-1.9738080
C	2.8119410	-3.1100400	1.0294310
H	4.2320910	-1.9665200	-0.1317070
H	3.4935000	-1.0289620	1.1736870
H	1.0127170	4.6641490	-0.4568950
H	2.6151530	4.4774800	0.2501690
O	-1.0011640	3.4503030	0.5427070
H	1.5087400	2.9270460	-2.1388560
H	3.0134730	3.8497280	-2.0894180
C	-2.8846970	-1.4645630	-0.2470270
O	-0.7544880	-3.4800560	-0.6518650
C	-2.9818970	1.2802160	0.1886030

H	1.4338710	-4.5533180	0.0984300
H	2.9082620	-4.2360440	-0.8089640
H	3.6117270	-3.6989420	1.4712260
H	2.0760890	-2.8987780	1.8066270
C	-4.1161940	-0.8264980	-0.1456270
H	-2.8371080	-2.5292330	-0.4172740
C	-4.1651290	0.5588550	0.0733260
H	-3.0090920	2.3461070	0.3554460
C	-5.3787750	-1.6175690	-0.2690230
C	-5.4806270	1.2599460	0.1867730
H	-5.9970380	-1.5322530	0.6270890
H	-5.1738770	-2.6729850	-0.4332580
H	-5.9959870	-1.2640050	-1.0977170
H	-6.0757480	0.8682100	1.0144370
H	-5.3512300	2.3279960	0.3459770
H	-6.0865630	1.1262280	-0.7118530
O	0.9306370	0.3713560	-1.8800840
H	0.0340200	0.2194090	-2.2049950
Cl	1.0829680	-0.2860980	2.2336100

52

M06L_LS_Ni_III_LD_ClOH_G3.log

52

Final Energy = -3114.5847375700

Ni	0.8346410	-0.0355530	-0.1840840
N	2.0314570	-1.5913140	-0.5869790
N	2.0823610	1.4841910	0.1589240
C	3.2520320	-1.8781840	0.2207980
H	2.2631660	-1.3120410	-1.5365030
N	-0.4861620	1.2982520	0.0421700
N	-0.5898550	-1.2287640	-0.3906980
C	3.4827210	1.7019660	-0.2536730
H	2.0320160	1.5157920	1.1783600
C	1.7423160	-3.6913460	0.5021840
C	-0.2945450	-2.5367150	-0.5089650
C	3.2279480	-3.3785900	0.4548570
H	3.1730750	-1.3374080	1.1629050
H	4.1282650	-1.5188030	-0.3135610
C	-1.7768930	0.7651230	0.0442860
C	-0.0931800	2.5882170	-0.0421510
C	-1.8356280	-0.6182620	-0.1991940

C	2.2610090	3.8203890	0.0306730
H	4.1523910	1.1281490	0.3808580
H	3.5867670	1.3466530	-1.2784800
H	1.3127470	-3.3756180	1.4547360
H	1.5055230	-4.7414450	0.3550940
O	-1.1133280	-3.4514110	-0.5189790
H	3.7560020	-3.6569300	1.3637420
H	3.6985780	-3.9030830	-0.3779530
C	-2.9546800	1.4829530	0.2325290
O	-0.8181780	3.5721960	0.0669310
C	-3.0754690	-1.2489830	-0.2638150
H	2.0912450	4.7098650	-0.5700630
H	2.0729740	4.0873800	1.0712070
C	-4.1908790	0.8476530	0.1816230
H	-2.8945460	2.5441600	0.4226420
C	-4.2512290	-0.5312020	-0.0699400
H	-3.1130820	-2.3098160	-0.4586950
C	-5.4439920	1.6337710	0.3982860
C	-5.5703380	-1.2331140	-0.1233130
H	-6.1117920	1.5726710	-0.4635950
H	-5.2294460	2.6844780	0.5794860
H	-6.0136030	1.2570290	1.2504510
H	-6.2200530	-0.8109220	-0.8928720
H	-5.4498590	-2.2933480	-0.3336860
H	-6.1154560	-1.1393910	0.8183490
C	3.6747750	3.2305900	-0.1529830
H	4.1488520	3.6082120	-1.0560360
H	4.3231560	3.4891770	0.6805490
C	1.3760330	2.6681400	-0.3846090
H	1.4227220	2.5462160	-1.4724440
C	1.1875570	-2.8278600	-0.6226540
H	1.3549670	-3.3268020	-1.5809550
O	0.9537270	0.2963160	-2.0062290
H	0.0734500	0.1403300	-2.3720180
Cl	0.8784860	-0.4558620	2.0930290

49

M06L_LS_Ni_III_LD_G1.log

49

Final Energy = -2578.1649380800

Ni	-1.0379380	0.0990120	-0.0734240

N	-2.1133320	1.7244650	-0.2309490
N	-2.4019800	-1.2785210	0.2101500
C	-1.2507070	2.8539780	-0.7180470
C	-2.7918490	2.1967850	1.0380150
H	-2.8256800	1.5519210	-0.9316200
N	0.2209740	-1.2405050	0.1251330
N	0.4487830	1.1741210	-0.2880870
C	-1.7469940	-2.5712420	0.6157640
C	-3.2714400	-1.6109390	-0.9621430
H	-3.0103500	-0.9922070	0.9714190
C	-1.7080420	4.0761220	0.0596930
C	0.2204690	2.5550040	-0.5054130
H	-1.3832740	2.9611680	-1.7956300
C	-2.0804480	3.4813980	1.4070930
H	-2.7324090	1.4305900	1.8071500
H	-3.8424670	2.3682590	0.8099970
C	1.4941060	-0.8704370	0.0665000
C	-0.2588730	-2.5533710	0.3620300
C	1.6317680	0.5785460	-0.1889500
C	-2.4776040	-3.6918910	-0.1387140
H	-1.8618780	-2.6788080	1.6957080
H	-4.0649910	-0.8735250	-1.0459370
H	-2.6564440	-1.5652330	-1.8614470
H	-0.9221970	4.8260630	0.1060520
H	-2.5754480	4.5207180	-0.4260880
O	1.0820710	3.3904910	-0.5327000
H	-1.1829670	3.2663950	1.9902920
H	-2.7109740	4.1323760	2.0061710
C	2.6465540	-1.6576570	0.2389510
O	0.4293160	-3.5370750	0.3679080
C	2.9135470	1.1457340	-0.2895950
H	-1.8508510	-4.0538450	-0.9533120
H	-2.6842690	-4.5376330	0.5100050
C	3.8797510	-1.0769420	0.1559790
H	2.5468990	-2.7122700	0.4353350
C	4.0164940	0.3564330	-0.1239940
H	3.0164250	2.2001880	-0.4844310
C	5.1019480	-1.8865810	0.3494180
C	5.3719190	0.9397960	-0.2160000
H	5.7508440	-1.8297150	-0.5263170

H 4.8678200 -2.9281220 0.5430330

H 5.6934200 -1.5004130 1.1818960

H 5.9518160 0.4512680 -1.0013900

H 5.3390930 2.0051210 -0.4189420

H 5.9289930 0.7758270 0.7083740

C -3.7329960 -3.0226150 -0.6784180

H -4.1230210 -3.5148510 -1.5654390

H -4.5236930 -3.0125860 0.0726100

49

M06L_LS_Ni_III_LD_G2.log

49

Final Energy = -2578.1643703400

Ni -1.0666980 0.0000050 -0.0000180

N -2.3035460 -1.4878050 0.2809990

N -2.3035360 1.4878250 -0.2810240

C -1.5417860 -2.7073410 0.7251050

C -3.1214180 -1.8895310 -0.9251200

H -2.9369300 -1.2190450 1.0261180

N 0.3085510 1.2059680 -0.2384760

N 0.3085420 -1.2059650 0.2384530

C -1.5417680 2.7073680 -0.7250970

C -3.1214110 1.8895270 0.9251010

H -2.9369170 1.2190810 -1.0261500

C -2.1300810 -3.8656390 -0.0663770

C -0.0515340 -2.5527310 0.4880380

H -1.6622430 -2.8278480 1.8025460

C -2.5199890 -3.2016250 -1.3756820

H -3.0880740 -1.1088560 -1.6803070

H -4.1556640 -2.0113940 -0.6068980

C 1.5426290 0.7248910 -0.1439550

C -0.0515140 2.5527380 -0.4880630

C 1.5426240 -0.7248970 0.1439400

C -2.1300460 3.8656500 0.0664210

H -1.6622310 2.8279060 -1.8025340

C -2.5199730 3.2016030 1.3757030

H -4.1556550 2.0114030 0.6068780

H -3.0880730 1.1088320 1.6802690

H -1.4078440 -4.6714150 -0.1719280

H -3.0058960 -4.2593270 0.4470300

O 0.7247740 -3.4678340 0.5185340

H	-1.6368430	-3.0237990	-1.9929640
H	-3.2208240	-3.7862260	-1.9648750
C	2.7648490	1.4031380	-0.2896700
O	0.7247840	3.4678540	-0.5184340
C	2.7648370	-1.4031530	0.2896610
H	-1.4077970	4.6714110	0.1719990
H	-3.0058530	4.2593700	-0.4469770
H	-3.2208070	3.7861960	1.9649060
H	-1.6368350	3.0237520	1.9929900
C	3.9384470	0.7178160	-0.1506900
H	2.7668180	2.4589710	-0.5035580
C	3.9384410	-0.7178420	0.1506840
H	2.7667960	-2.4589860	0.5035500
C	5.2333930	1.4162640	-0.2959140
C	5.2333800	-1.4163000	0.2959100
H	5.8209630	1.3358690	0.6208640
H	5.0999770	2.4667570	-0.5325120
H	5.8397750	0.9538110	-1.0768770
H	5.8397640	-0.9538540	1.0768750
H	5.0999550	-2.4667930	0.5325040
H	5.8209540	-1.3359070	-0.6208670

49

M06L_LS_Ni_III_LD_G3.log

49

Final Energy = -2578.1571976600

Ni	-0.9647090	0.0535110	-0.1502140
N	-2.0919600	1.6303560	-0.2800770
N	-2.2246220	-1.4090420	0.1596870
C	-2.8460560	2.0133950	0.9541710
H	-2.7659970	1.4911620	-1.0280040
N	0.3552450	-1.2552870	0.0306160
N	0.4854140	1.1964540	-0.2179650
C	-3.6197920	-1.6274620	-0.3062180
H	-2.2422620	-1.4704950	1.1796310
C	-1.8314790	4.0156020	0.1606320
C	0.2082330	2.5804600	-0.3563820
C	-3.1310520	3.4847250	0.7534010
H	-2.1943700	1.8437750	1.8116760
H	-3.7217350	1.3788630	1.0569680
C	1.6069450	-0.8262090	0.0390250

C -0.0872180 -2.6067390 0.0798930
C 1.6874130 0.6440880 -0.0983320
C -2.4540380 -3.7688620 0.0187140
H -4.3017540 -0.9988380 0.2582080
H -3.6650070 -1.3320080 -1.3529990
H -1.1324330 4.3019410 0.9454450
H -1.9759000 4.8844740 -0.4741710
O 1.0321580 3.4501040 -0.2822440
H -3.4021210 3.9820770 1.6809010
H -3.9589020 3.6108040 0.0550990
C 2.7871220 -1.5841700 0.1422370
O 0.5913100 -3.5525060 0.3681330
C 2.9469840 1.2670690 -0.1002960
H -2.3074680 -4.6309010 -0.6254340
H -2.2622120 -4.0902980 1.0420770
C 3.9969940 -0.9527580 0.1339960
H 2.7226280 -2.6562160 0.2363110
C 4.0775310 0.5077070 0.0138180
H 3.0121620 2.3382470 -0.1950940
C 5.2491160 -1.7316130 0.2476940
C 5.4091230 1.1502250 0.0127340
H 5.8934290 -1.5603310 -0.6166800
H 5.0539450 -2.7957150 0.3305410
H 5.8277380 -1.4132040 1.1168790
H 6.0256050 0.7663750 -0.8022640
H 5.3356100 2.2283790 -0.0834900
H 5.9527120 0.9163540 0.9302530
C -3.8541250 -3.1357810 -0.1241250
H -4.3950910 -3.5382500 -0.9764230
H -4.4574840 -3.3305540 0.7588780
C -1.5303320 -2.6294030 -0.3455230
H -1.5027950 -2.5139460 -1.4365280
C -1.2569850 2.8302260 -0.6305990
H -1.3330300 2.9888390 -1.7077590
60
M06L_LS_Ni_III_LD_MeCNMeOH_G1.log
60
Final Energy = -2826.3083518400
Ni 0.9083830 0.1296290 -0.2626590
N 2.1381880 1.5861460 0.3006390

N	2.2280730	-1.2660000	-0.7561610
C	1.3376440	2.6936860	0.9211290
C	2.9921630	2.1888250	-0.7658910
H	2.7016790	1.1471930	1.0229280
N	-0.3647200	-1.1981620	-0.3241460
N	-0.4593130	1.1705080	0.3843480
C	1.5216980	-2.5822070	-0.9049270
C	3.4306670	-1.4872260	0.1024050
H	2.5023410	-0.9382140	-1.6780970
C	1.7774460	3.9692440	0.2104630
C	-0.1566680	2.4403070	0.8243710
H	1.5584000	2.7225490	1.9894790
C	2.2683050	3.4627350	-1.1359330
H	3.1029250	1.4926400	-1.5935900
H	3.9775560	2.3963440	-0.3457820
C	-1.6504570	-0.7290100	-0.2376660
C	0.0354560	-2.4775810	-0.6409080
C	-1.7055920	0.6408260	0.1674150
C	2.2413270	-3.5278770	0.0547820
H	1.6460340	-2.9281210	-1.9329580

H	4.2578820	-0.8883220	-0.2702660
H	3.1924620	-1.1567320	1.1139520
H	0.9595060	4.6834820	0.1485360
H	2.5902600	4.4410720	0.7613160
O	-0.9711900	3.2660720	1.1815130
H	1.4281920	3.2258170	-1.7915160
H	2.9133100	4.1686600	-1.6524640
C	-2.8329860	-1.4165730	-0.5233450
O	-0.6898460	-3.4509580	-0.6587140
C	-2.9461060	1.2756180	0.2870030
H	1.7960710	-3.4580540	1.0497780
H	2.1616750	-4.5613060	-0.2699780
C	-4.0520700	-0.7840570	-0.3864720
H	-2.7851920	-2.4475890	-0.8372390
C	-4.1089310	0.5787260	0.0272490
H	-2.9856900	2.3095460	0.5910940
C	-5.3135690	-1.5208910	-0.6771850
C	-5.4304120	1.2514910	0.1683510
H	-5.9716100	-1.5394590	0.1934230
H	-5.1175720	-2.5466250	-0.9770220

H -5.8807160 -1.0358210 -1.4738220
H -6.0585900 0.7401660 0.9001430
H -5.3211420 2.2867250 0.4793620
H -5.9862380 1.2342870 -0.7708600
C 3.6591370 -2.9844700 0.0690020
H 4.2427940 -3.3308660 0.9182370
H 4.1879980 -3.2703460 -0.8411990
N 1.4605300 -0.2637140 2.5704590
C 0.6274450 -0.9152680 3.0345330
C -0.4238720 -1.7263190 3.5848210
H -1.3504600 -1.1568930 3.6297340
H -0.5815910 -2.6034750 2.9590220
H -0.1646050 -2.0550120 4.5889230
O 0.9271000 0.6730170 -2.1125970
C -0.2419370 0.5803650 -2.8301940
H -1.1426870 0.8492640 -2.2501950
H -0.2100270 1.3164750 -3.6453820
H -0.4176270 -0.4155300 -3.2627270
60
M06L_LS_Ni_III_LD_MeCNMeOH_G2.log
60
Final Energy = -2826.3090434300
Ni 0.9486680 0.1008190 -0.2617140
N 2.2176420 1.5454510 0.2480020
N 2.2322770 -1.3562300 -0.6940780
C 1.4503280 2.6917720 0.8403280
C 3.0716420 2.0936890 -0.8465930
H 2.7798130 1.1200280 0.9792240
N -0.3628620 -1.1848540 -0.3079620
N -0.3865310 1.1943090 0.3667500
C 1.4496880 -2.5645540 -1.1141940
C 3.2297760 -1.7952000 0.3337370
H 2.6974330 -0.9701000 -1.5091160
C 1.8891870 3.9285480 0.0606550
C -0.0489560 2.4611960 0.7901850
H 1.7004820 2.7659580 1.8996560
C 2.3533470 3.3542020 -1.2679180
H 3.1758860 1.3601930 -1.6422570
H 4.0595770 2.3146010 -0.4396410
C -1.6341360 -0.6804370 -0.2139120

C	-0.0030490	-2.4646730	-0.6698720
C	-1.6488620	0.6962340	0.1685550
C	2.1657840	-3.7471280	-0.4811600
H	1.4306040	-2.6129530	-2.2041710
C	2.7294940	-3.1456820	0.7958290
H	4.2047630	-1.8772780	-0.1469800
H	3.3001980	-1.0573030	1.1298980
H	1.0772210	4.6474290	-0.0219450
H	2.7154570	4.4183940	0.5744020
O	-0.8394470	3.3046220	1.1595950
H	1.5010850	3.0933220	-1.8985050
H	2.9958710	4.0288260	-1.8275570
C	-2.8365910	-1.3402420	-0.4827940
O	-0.7419450	-3.4260470	-0.6431190
C	-2.8705550	1.3678150	0.2810800
H	1.4783230	-4.5742780	-0.3205250
H	2.9685140	-4.0926800	-1.1313320
H	3.5178340	-3.7416780	1.2473490
H	1.9411200	-3.0242990	1.5422130
C	-4.0364420	-0.6715530	-0.3530970
H	-2.8190400	-2.3778420	-0.7778730
C	-4.0532710	0.6998410	0.0368390
H	-2.8800050	2.4075500	0.5670970
C	-5.3194250	-1.3772980	-0.6264100
C	-5.3547620	1.4118590	0.1703950
H	-5.9751620	-1.3600980	0.2458980
H	-5.1538420	-2.4137890	-0.9070070
H	-5.8745960	-0.8914590	-1.4309800
H	-5.9938510	0.9320060	0.9140030
H	-5.2152270	2.4492300	0.4615840
H	-5.9149260	1.3928180	-0.7661760
N	1.5298700	-0.1387010	2.6271280
C	0.6648440	-0.7452740	3.0937070
C	-0.4249240	-1.5025550	3.6467210
H	-0.6251360	-2.3741110	3.0247600
H	-0.1821920	-1.8389680	4.6523860
H	-1.3230770	-0.8891100	3.6888540
O	0.9952020	0.5364910	-2.1380480
C	-0.1850700	0.5024940	-2.8434800
H	-1.0566580	0.8664640	-2.2713800

H -0.1031310 1.1933300 -3.6939180

H -0.4415650 -0.4958060 -3.2278790

60

M06L_LS_Ni_III_LD_MeCNMeOH_G3.log

60

Final Energy = -2826.3005509200

Ni -0.8435200 0.1012110 -0.3911580

N -1.9734540 1.7111960 -0.5031030

N -2.1893180 -1.3117400 -0.0796720

C -2.8958820 2.0140030 0.6249970

H -2.5068440 1.5775340 -1.3577400

N 0.3848050 -1.2140500 0.0479080

N 0.5832460 1.2445730 -0.2018590

C -3.5220870 -1.5052100 -0.6883150

H -2.2848930 -1.3070440 0.9421580

C -1.6932230 4.0142020 0.2133770

C 0.3497800 2.5988920 -0.3319130

C -3.0919810 3.5121750 0.5421660

H -2.4002840 1.7302930 1.5547470

H -3.8011180 1.4211070 0.5283590

C 1.6839980 -0.7941580 0.0207090

C -0.0768790 -2.5219320 0.1033870

C 1.8022560 0.6217130 -0.1340360

C -2.4660310 -3.6508960 -0.1118140

H -4.2511760 -0.8595550 -0.2067120

H -3.4477400 -1.2213460 -1.7375650

H -1.0980120 4.1156420 1.1215030

H -1.6857260 4.9791360 -0.2850890

O 1.1811110 3.4671650 -0.1732060

H -3.4872530 3.9322920 1.4636290

H -3.7912070 3.7519910 -0.2597870

C 2.8355990 -1.5857940 0.0876430

O 0.5545920 -3.4799860 0.4912680

C 3.0707260 1.2019180 -0.2250610

H -2.2577920 -4.5768660 -0.6406330

H -2.4321180 -3.8671340 0.9566250

C 4.0814730 -0.9994980 0.0205070

H 2.7376030 -2.6540640 0.2061190

C 4.2003550 0.4136990 -0.1393820

H 3.1590380 2.2700650 -0.3466980

C	5.3094290	-1.8380440	0.1090530
C	5.5514830	1.0356970	-0.2157800
H	5.9258200	-1.7340910	-0.7857390
H	5.0670690	-2.8897880	0.2333240
H	5.9386770	-1.5317870	0.9466890
H	6.1299200	0.6266330	-1.0460780
H	5.4891450	2.1127800	-0.3436180
H	6.1324330	0.8328300	0.6858620
C	-3.8104160	-3.0109100	-0.5164640
H	-4.1877020	-3.4301820	-1.4462170
H	-4.5704250	-3.1805720	0.2422200
C	-1.4784090	-2.5594760	-0.4481510
H	-1.3514520	-2.4943690	-1.5377370
C	-1.0930360	2.9138330	-0.6727570
H	-1.1113270	3.1974060	-1.7265560
N	-1.5461260	-1.0767500	2.8378850
C	-0.7211830	-0.3771930	3.2414350
C	0.3086240	0.5047950	3.7194410
H	1.2887780	0.0650850	3.5439990
H	0.2533240	1.4563140	3.1918850
H	0.1900750	0.6867370	4.7852370
O	-0.9640890	-0.1296180	-2.2973940
C	0.2100650	-0.0145520	-3.0118520
H	1.0741880	-0.5046130	-2.5302920
H	0.0846550	-0.5360710	-3.9703940
H	0.4979600	1.0250670	-3.2256660

57

M06L_LS_Ni_III_LD_MeCNOH_G1.log

57

Final Energy = -2786.9945227500

Ni	0.9206190	0.1236250	-0.3742550
N	2.1752430	1.5331460	0.2498150
N	2.2095010	-1.3309110	-0.7515290
C	1.3859800	2.6929750	0.7801900
C	3.1363570	2.0654180	-0.7613700
H	2.6576610	1.0854070	1.0237350
N	-0.3992830	-1.1548370	-0.4671520
N	-0.4405710	1.2230010	0.1833490
C	1.4652180	-2.6233640	-0.9092080
C	3.3573230	-1.5715260	0.1727500

H	2.5311190	-1.0238260	-1.6660420
C	1.9317840	3.9263430	0.0689890
C	-0.1084110	2.4991120	0.5974450
H	1.5353990	2.7445510	1.8603360
C	2.5023500	3.3581530	-1.2200640
H	3.2791720	1.3411570	-1.5592820
H	4.0942340	2.2401390	-0.2687870
C	-1.6761560	-0.6584710	-0.3900560
C	-0.0257600	-2.4613740	-0.7155420
C	-1.6996440	0.7204530	-0.0217170
C	2.1029110	-3.5719700	0.1045590
H	1.6266430	-2.9959310	-1.9224230
H	4.2221350	-1.0073010	-0.1672540
H	3.0775430	-1.2130490	1.1641670
H	1.1519880	4.6703700	-0.0770890
H	2.7186720	4.3828810	0.6680830
O	-0.9071410	3.3663560	0.8785680
H	1.7057400	3.1354450	-1.9314910
H	3.2169950	4.0196030	-1.7026420
C	-2.8733020	-1.3310340	-0.6460870
O	-0.7817490	-3.4095860	-0.7174070
C	-2.9238730	1.3853160	0.0896670
H	1.6112330	-3.4662370	1.0742570
H	2.0038770	-4.6086960	-0.2038320
C	-4.0779610	-0.6699340	-0.5160040
H	-2.8498010	-2.3708240	-0.9316660
C	-4.1030280	0.7054550	-0.1410240
H	-2.9405240	2.4278040	0.3643520
C	-5.3569040	-1.3890230	-0.7719870
C	-5.4089760	1.4086870	-0.0061690
H	-6.0046270	-1.3714040	0.1063500
H	-5.1847140	-2.4260090	-1.0466700
H	-5.9240180	-0.9137640	-1.5745580
H	-6.0410950	0.9293060	0.7436740
H	-5.2758340	2.4491440	0.2768260
H	-5.9733890	1.3787630	-0.9399530
C	3.5360790	-3.0758850	0.1802950
H	4.0639780	-3.4230640	1.0648830
H	4.0999420	-3.3982400	-0.6959720
N	1.2751840	-0.2325990	2.5117650

C	0.3759440	-0.8339990	2.9163580
C	-0.7574880	-1.5805070	3.3902410
H	-1.6661840	-0.9928120	3.2719220
H	-0.8600100	-2.5025330	2.8197650
H	-0.6351680	-1.8315450	4.4415690
O	1.1013470	0.6155230	-2.2076580
H	0.2468680	0.4672560	-2.6324840

57

M06L_LS_Ni_III_LD_MeCNOH_G2.log

57

Final Energy = -2786.9952554400

Ni	0.9763330	0.0837580	-0.3705410
N	2.2846950	1.4436360	0.2607910
N	2.1979210	-1.4542410	-0.6678160
C	1.5440560	2.6565090	0.7407640
C	3.3030180	1.9013080	-0.7294170
H	2.7183770	0.9916300	1.0605620
N	-0.4015750	-1.1256890	-0.4767480
N	-0.3385430	1.2586820	0.1454040
C	1.3810220	-2.6257820	-1.1206570
C	3.0866470	-1.9195870	0.4442580
H	2.7387680	-1.1094170	-1.4545030
C	2.1512270	3.8387740	-0.0094380
C	0.0449230	2.5225000	0.5526910
H	1.6885290	2.7433150	1.8192050
C	2.7351550	3.1978570	-1.2578180
H	3.4494560	1.1431540	-1.4943230
H	4.2475060	2.0609130	-0.2068260
C	-1.6552880	-0.5755870	-0.3958130
C	-0.0855500	-2.4400760	-0.7600130
C	-1.6187470	0.8084090	-0.0511280
C	1.9939570	-3.8320280	-0.4282520
H	1.4255330	-2.6898540	-2.2084550
C	2.4943890	-3.2418440	0.8800350
H	4.0932980	-2.0464270	0.0460980
H	3.1229980	-1.1749460	1.2365490
H	1.4029140	4.6026750	-0.2079660
H	2.9379740	4.2937450	0.5908360
O	-0.7182090	3.4252130	0.8210530
H	1.9518560	2.9772120	-1.9843020

H	3.4896180	3.8116220	-1.7428190
C	-2.8808060	-1.2027140	-0.6347530
O	-0.8706000	-3.3620880	-0.7172860
C	-2.8134770	1.5268670	0.0486380
H	1.2578540	-4.6234620	-0.3082390
H	2.8219920	-4.2224330	-1.0184350
H	3.2221220	-3.8669440	1.3901610
H	1.6625960	-3.0779470	1.5692840
C	-4.0557150	-0.4896500	-0.5125680
H	-2.9021190	-2.2483850	-0.8987220
C	-4.0209600	0.8932150	-0.1662010
H	-2.7853720	2.5743010	0.3025760
C	-5.3655560	-1.1599260	-0.7441090
C	-5.2952630	1.6545600	-0.0445150
H	-5.9979720	-1.1108220	0.1442910
H	-5.2381560	-2.2050150	-1.0124000
H	-5.9265770	-0.6690340	-1.5413240
H	-5.9531220	1.2096010	0.7042080
H	-5.1178440	2.6904320	0.2309440
H	-5.8540080	1.6424340	-0.9822340
N	1.2016870	-0.1852170	2.5456630
C	0.2567220	-0.7271010	2.9297010
C	-0.9267230	-1.4097990	3.3777640
H	-1.0775840	-2.3146260	2.7902620
H	-0.8319390	-1.6858320	4.4257430
H	-1.7977270	-0.7674330	3.2606590
O	1.2483710	0.4945110	-2.2102780
H	0.3897540	0.4496080	-2.6496750

57

M06L_LS_Ni_III_LD_MeCNOH_G3.log

57

Final Energy = -2786.9873340300

Ni	-0.9029210	0.0097820	-0.5283170
N	-2.0093960	1.6249940	-0.7223140
N	-2.2241150	-1.3300190	0.1059470
C	-3.0570100	1.9214630	0.2990480
H	-2.4262030	1.4248080	-1.6286240
N	0.3441090	-1.3008200	-0.1083800
N	0.5578590	1.1279100	-0.5114520
C	-3.6408180	-1.5268590	-0.2692470

H	-2.1545260	-1.2068970	1.1192320
C	-1.6095360	3.7892970	0.2518980
C	0.3390510	2.4851950	-0.6870010
C	-3.0772880	3.4322330	0.4002360
H	-2.7516180	1.4683610	1.2433980
H	-3.9975380	1.4743830	-0.0101940
C	1.6461600	-0.8954580	-0.1580740
C	-0.1359130	-2.5862730	0.1021770
C	1.7746790	0.5043650	-0.4100230
C	-2.5461200	-3.6599540	0.2811450
H	-4.2708770	-0.8503330	0.3008190
H	-3.7384440	-1.2846640	-1.3269430
H	-1.0737520	3.5943680	1.1837530
H	-1.4265610	4.8246070	-0.0208770
O	1.2001860	3.3364420	-0.6558990
H	-3.5088370	3.7764660	1.3367150
H	-3.6612110	3.8597970	-0.4157620
C	2.7907350	-1.6876490	-0.0237570
O	0.5155380	-3.5313720	0.4894280
C	3.0472800	1.0699620	-0.5336330
H	-2.4242100	-4.6256600	-0.2017110
H	-2.3737170	-3.8024260	1.3482590
C	4.0408860	-1.1142200	-0.1208730
H	2.6853530	-2.7442070	0.1671690
C	4.1706550	0.2834720	-0.3809910
H	3.1447500	2.1252450	-0.7319480
C	5.2625990	-1.9501570	0.0408090
C	5.5266320	0.8896590	-0.4888850
H	5.8938120	-1.9046920	-0.8484280
H	5.0134520	-2.9908890	0.2279440
H	5.8796760	-1.5929580	0.8673450
H	6.1050260	0.4246350	-1.2893930
H	5.4733490	1.9569240	-0.6848340
H	6.1023810	0.7385900	0.4260460
C	-3.9204830	-3.0181580	0.0107440
H	-4.4085760	-3.4747630	-0.8470730
H	-4.5876100	-3.1405160	0.8602300
C	-1.5962000	-2.6206450	-0.2621600
H	-1.6171280	-2.6300570	-1.3605210
C	-1.1247450	2.8299110	-0.8369670

H	-1.2661630	3.2733270	-1.8237690
N	-0.9789480	0.0705650	2.4925730
C	-0.0394730	0.6178580	2.8809940
C	1.1361490	1.3011370	3.3480300
H	2.0169520	0.6814180	3.1891700
H	1.2640660	2.2371080	2.8062160
H	1.0489370	1.5215730	4.4097340
O	-1.3288210	-0.3981930	-2.3471990
H	-0.5042330	-0.4019420	-2.8491780

55

M06L_LS_Ni_II_LD_ClMeOH_G1.log

55

Final Energy = -3154.0686493300

Ni	0.8613540	0.0277390	-0.1114730
N	2.0661760	1.5406800	0.3659320
N	2.1516870	-1.3978900	-0.6339730
C	1.2327170	2.7109030	0.7994020
C	3.0212390	2.0161810	-0.6673800
H	2.5363510	1.1479870	1.1787450
N	-0.4528150	-1.2920920	-0.2523630
N	-0.5426110	1.1737270	0.3236670
C	1.4327350	-2.7070270	-0.6328860
C	3.4506770	-1.5737600	0.0746060
H	2.2703610	-1.0772120	-1.5929660
C	1.7486960	3.8978480	-0.0065990
C	-0.2589560	2.4533330	0.6127430
H	1.3752970	2.8601340	1.8717540
C	2.3538210	3.2461850	-1.2394740
H	3.1901600	1.2356210	-1.4054550
H	3.9716020	2.2668630	-0.1894960
C	-1.7516570	-0.7991350	-0.0921760
C	-0.0722960	-2.5540620	-0.5078990
C	-1.8016380	0.5764910	0.2140530
C	2.0800370	-3.4693120	0.5146460
H	1.6398910	-3.2272890	-1.5729920
H	4.2648320	-1.2107880	-0.5499200
H	3.4227080	-0.9825990	0.9904860
H	0.9435770	4.5982330	-0.2202270
H	2.5150590	4.4336740	0.5543560
O	-1.0672940	3.3694550	0.7805200

H	1.5715930	2.9340590	-1.9333300
H	3.0495440	3.8892670	-1.7738340
C	-2.9391750	-1.5158720	-0.2158440
O	-0.8026340	-3.5429900	-0.6169890
C	-3.0381880	1.1933570	0.3876000
H	1.6623350	-3.1197290	1.4613810
H	1.9142080	-4.5408220	0.4362760
C	-4.1737430	-0.8950690	-0.0402600
H	-2.8913680	-2.5692960	-0.4492090
C	-4.2235940	0.4724360	0.2641470
H	-3.0679530	2.2471620	0.6216180
C	-5.4358220	-1.6867130	-0.1767960
C	-5.5395930	1.1580900	0.4546700
H	-6.0266430	-1.6699250	0.7420180
H	-5.2291280	-2.7271840	-0.4185990
H	-6.0841080	-1.2855810	-0.9593050
H	-6.1182710	0.7074330	1.2643070
H	-5.4089540	2.2127610	0.6879510
H	-6.1661700	1.0900170	-0.4377200
C	3.5378130	-3.0568630	0.4003400
H	4.1099740	-3.2430300	1.3070220
H	4.0207040	-3.6016060	-0.4129220
O	0.9812890	0.4334550	-2.1251050
C	-0.1695610	0.8214960	-2.7656850
H	-0.5451650	1.8131830	-2.4478700
H	-0.0038650	0.8936950	-3.8532000
H	-1.0126000	0.1190360	-2.6340600
Cl	1.4005340	-0.4677740	2.6860550

55

M06L_LS_Ni_II_LD_ClMeOH_G2.log

55

Final Energy = -3154.0674211600

Ni	-0.8883840	0.0024270	-0.1171640
N	-2.1485490	-1.4642160	0.3621090
N	-2.1386800	1.4990450	-0.5315260
C	-1.3620940	-2.6978750	0.6924500
C	-3.1887400	-1.8369920	-0.6263130
H	-2.5379460	-1.0833850	1.2235610
N	0.4734900	1.2676160	-0.2381630
N	0.4693360	-1.2138130	0.2587610

C	-1.3304620	2.7172700	-0.8740400
C	-3.1959980	1.9055470	0.4304020
H	-2.5256540	1.1143210	-1.3880210
C	-1.9364690	-3.7992480	-0.1969690
C	0.1367920	-2.4886340	0.5198700
H	-1.5055960	-2.9276080	1.7504990
C	-2.5998970	-3.0365860	-1.3330010
H	-3.3926880	-1.0000240	-1.2912790
H	-4.1101020	-2.0991910	-0.0998210
C	1.7535200	0.7254010	-0.0760790
C	0.1529580	2.5280730	-0.5757990
C	1.7509450	-0.6613050	0.1784340
C	-1.9649810	3.8588610	-0.0856260
H	-1.3970910	2.8877440	-1.9500510
C	-2.6338590	3.1491500	1.0803530
H	-4.1151360	2.1253020	-0.1179770
H	-3.3905730	1.0999340	1.1367190
H	-1.1548710	-4.4845810	-0.5193680
H	-2.6771980	-4.3806150	0.3525620
O	0.9105490	-3.4366900	0.6692790
H	-1.8584490	-2.6910600	-2.0541330
H	-3.3499970	-3.6189960	-1.8634420
C	2.9669200	1.4037180	-0.1590360
O	0.9267250	3.4803150	-0.6908900
C	2.9628030	-1.3299310	0.3338630
H	-1.2149490	4.5908340	0.2073170
H	-2.7094870	4.3723820	-0.6948130
H	-3.3996810	3.7453950	1.5711570
H	-1.8988290	2.8540640	1.8321710
C	4.1763490	0.7320560	0.0019470
H	2.9599200	2.4666330	-0.3493980
C	4.1742770	-0.6480200	0.2490300
H	2.9523570	-2.3927790	0.5240280
C	5.4669440	1.4831360	-0.0887590
C	5.4627870	-1.3879360	0.4229930
H	6.0491330	1.4021710	0.8320990
H	5.2994040	2.5405020	-0.2827780
H	6.1069380	1.0961150	-0.8850750
H	6.0430100	-0.9999280	1.2634230
H	5.2925240	-2.4478900	0.5996800

H 6.1057560 -1.2958350 -0.4553650
O -1.0986430 -0.3063800 -2.1303950
C 0.0051890 -0.7722950 -2.8014840
H 0.2822230 -1.8125680 -2.5414970
H -0.1715860 -0.7661850 -3.8894110
H 0.9137190 -0.1644590 -2.6385180
Cl -1.3529730 0.2480600 2.8654520
55
M06L_LS_Ni_II_LD_ClMeOH_G3.log
55
Final Energy = -3154.0610539300
Ni 0.8172970 0.0721160 0.2387670
N 1.9570690 1.6416410 0.6216790
N 2.0759880 -1.3892480 -0.2293690
C 3.1945450 1.9014830 -0.1663610
H 2.1590480 1.3850440 1.5862340
N -0.4865800 -1.2505560 -0.0792410
N -0.6252810 1.2586920 0.3007990
C 3.4852780 -1.6352340 0.1141230
H 1.9847270 -1.3231310 -1.2487390
C 1.6866370 3.6885690 -0.5591550
C -0.3686710 2.5693950 0.4468810
C 3.1730490 3.3903870 -0.4648520
H 3.1315880 1.3238240 -1.0891030
H 4.0642530 1.5692930 0.3970480
C -1.7871090 -0.7453200 -0.1184200
C -0.0883860 -2.5360860 -0.0812780
C -1.8671000 0.6451910 0.0998290
C 2.2687170 -3.7344420 -0.2681450
H 4.1339400 -1.0148390 -0.4997860
H 3.6269190 -1.3511710 1.1574290
H 1.2853320 3.3151470 -1.5040000
H 1.4365400 4.7430120 -0.4748670
O -1.1888440 3.4906680 0.4493540
H 3.7205400 3.6344930 -1.3729150
H 3.6206970 3.9556690 0.3547520
C -2.9538140 -1.4750020 -0.3304960
O -0.7847950 -3.5418840 -0.2364400
C -3.1153840 1.2625940 0.1080770
H 2.1228480 -4.6633860 0.2778140

H	2.0465580	-3.9361130	-1.3174310
C	-4.1998140	-0.8523530	-0.3252020
H	-2.8800480	-2.5394870	-0.4997350
C	-4.2809820	0.5293390	-0.1028450
H	-3.1702450	2.3274120	0.2793580
C	-5.4398480	-1.6566320	-0.5563790
C	-5.6093590	1.2176590	-0.0933350
H	-6.1305080	-1.5893310	0.2873800
H	-5.2090690	-2.7083320	-0.7126390
H	-5.9947720	-1.3046690	-1.4291240
H	-6.2719620	0.8107440	0.6740950
H	-5.5036220	2.2845670	0.0918980
H	-6.1366090	1.0956450	-1.0423360
C	3.6855430	-3.1512740	-0.0979660
H	4.1963280	-3.5896890	0.7571800
H	4.3037480	-3.3493980	-0.9709800
C	1.3903610	-2.6128320	0.2413250
H	1.4576120	-2.5641600	1.3359810
C	1.1109230	2.8738700	0.5919230
H	1.2531830	3.4209660	1.5284100
O	1.1028100	-0.2807420	2.2343840
C	-0.0030620	-0.6672720	2.9549490
H	-0.3348490	-1.7017210	2.7432750
H	0.2038210	-0.6284050	4.0365610
H	-0.8855390	-0.0238890	2.7904040
Cl	1.1596660	0.4213170	-2.6501840

52

M06L_LS_Ni_II_LD_ClOH_G1.log

52

Final Energy = -3114.7593658800

Ni	0.8567560	0.0500550	-0.2131170
N	2.0613160	1.5755580	0.2063010
N	2.1427700	-1.4017870	-0.6729240
C	1.2314880	2.7495640	0.6372210
C	2.9854180	2.0336100	-0.8645240
H	2.5579980	1.2077770	1.0143340
N	-0.4624690	-1.2705530	-0.3275080
N	-0.5497360	1.2138350	0.1771580
C	1.4218550	-2.7066120	-0.6288700
C	3.4454950	-1.5567740	0.0297560

H	2.2394820	-1.0905440	-1.6397470
C	1.7464160	3.9293400	-0.1782550
C	-0.2619160	2.4942590	0.4572880
H	1.3786340	2.9023840	1.7082980
C	2.3138790	3.2679630	-1.4235580
H	3.1211100	1.2450040	-1.6007670
H	3.9542150	2.2752010	-0.4207450
C	-1.7613970	-0.7675030	-0.1923080
C	-0.0835850	-2.5451390	-0.5196570
C	-1.8091380	0.6160270	0.0758940
C	2.0621900	-3.4264470	0.5492920
H	1.6329020	-3.2622780	-1.5480130
H	4.2572620	-1.2320410	-0.6186850
H	3.4344100	-0.9231800	0.9172910
H	0.9468500	4.6411100	-0.3742620
H	2.5326170	4.4533620	0.3664350
O	-1.0675120	3.4128010	0.6246950
H	1.5108230	2.9564730	-2.0933090
H	3.0011280	3.9023210	-1.9789250
C	-2.9501420	-1.4850220	-0.2996110

O	-0.8171350	-3.5348140	-0.5894080
C	-3.0454070	1.2388780	0.2321610
H	1.6431050	-3.0376510	1.4798860
H	1.8925130	-4.4996180	0.5125540
C	-4.1838980	-0.8576450	-0.1445070
H	-2.9037150	-2.5446110	-0.5032010
C	-4.2316840	0.5174670	0.1245430
H	-3.0733890	2.2982160	0.4401790
C	-5.4468670	-1.6504110	-0.2654070
C	-5.5466350	1.2105780	0.2940660
H	-6.0439040	-1.6046610	0.6483310
H	-5.2407120	-2.6981260	-0.4741350
H	-6.0889170	-1.2722560	-1.0643510
H	-6.1283000	0.7821440	1.1135280
H	-5.4141920	2.2705930	0.5004800
H	-6.1710340	1.1210000	-0.5979270
C	3.5223250	-3.0249580	0.4222960
H	4.0903220	-3.1739440	1.3384210
H	4.0054330	-3.6075810	-0.3640540
O	0.9611290	0.4144980	-2.2277140

H 0.1043030 0.1303880 -2.5644140

Cl 1.3745000 -0.3543130 2.5760820

52

M06L_LS_Ni_II_LD_ClOH_G2.log

52

Final Energy = -3114.7579017700

Ni	0.8887410	0.0321540	-0.2177230
N	2.1375480	1.5166390	0.2210090
N	2.1468200	-1.4782110	-0.5719320
C	1.3423730	2.7462220	0.5481660
C	3.1551590	1.8849860	-0.7945330
H	2.5501130	1.1561200	1.0792460
N	-0.4689210	-1.2417760	-0.3341260
N	-0.4821200	1.2527930	0.1128840
C	1.3475980	-2.7099720	-0.8761830
C	3.2205390	-1.8491030	0.3834110
H	2.5028910	-1.0937540	-1.4438840
C	1.9120970	3.8474750	-0.3431030
C	-0.1559130	2.5284340	0.3745090
H	1.4830610	2.9777180	1.6060140
C	2.5521880	3.0837880	-1.4915670
H	3.3416910	1.0445890	-1.4599820
H	4.0885700	2.1463500	-0.2896720
C	-1.7527860	-0.7019530	-0.1871150
C	-0.1357010	-2.5222280	-0.5724530
C	-1.7592130	0.6894610	0.0389890
C	2.0022970	-3.8281500	-0.0725090
H	1.4041330	-2.9047010	-1.9493420
C	2.6889120	-3.0898560	1.0654810
H	4.1369170	-2.0630270	-0.1720440
H	3.4097450	-1.0267590	1.0717400
H	1.1315720	4.5408690	-0.6501740
H	2.6657710	4.4193830	0.1990890
O	-0.9345570	3.4723130	0.5270490
H	1.7959870	2.7326130	-2.1945490
H	3.2920690	3.6649610	-2.0375600
C	-2.9617620	-1.3892810	-0.2605850
O	-0.9001090	-3.4876900	-0.6109250
C	-2.9761130	1.3522710	0.1824840
H	1.2611400	-4.5565100	0.2506570

H 2.7378220 -4.3521880 -0.6835330

H 3.4737870 -3.6688980 1.5468910

H 1.9677310 -2.7934760 1.8296110

C -4.1758410 -0.7227720 -0.1153220

H -2.9472640 -2.4556430 -0.4301370

C -4.1828610 0.6613180 0.1081910

H -2.9726170 2.4183180 0.3545260

C -5.4615220 -1.4832280 -0.1974100

C -5.4765020 1.3955080 0.2676360

H -6.0491450 -1.3870000 0.7184960

H -5.2868000 -2.5432860 -0.3691530

H -6.0994400 -1.1162600 -1.0048480

H -6.0560920 1.0168010 1.1127240

H -5.3136180 2.4592040 0.4279840

H -6.1167560 1.2852870 -0.6106310

O 1.1035070 0.3414340 -2.2239500

H 0.3112260 -0.0495480 -2.6088950

Cl 1.2835910 -0.1802100 2.7013480

52

M06L_LS_Ni_II_LD_ClOH_G3.log

52

Final Energy = -3114.7510677100

Ni -0.7298050 0.1666220 -0.5138230

N -1.7972420 1.8174730 -0.6800350

N -2.0810720 -1.2256500 -0.1485970

C -2.9984120 2.0135130 0.1816960

H -2.0352810 1.6906740 -1.6619390

N 0.4871770 -1.2248350 -0.1770900

N 0.7540750 1.2832820 -0.3520600

C -3.4813810 -1.3818090 -0.5713590

H -2.0630730 -1.2018760 0.8858940

C -1.4382310 3.7075400 0.7237940

C 0.5629170 2.6177310 -0.3930680

C -2.9333900 3.4626950 0.6259470

H -2.9201100 1.3398090 1.0387170

H -3.8950350 1.7539720 -0.3768540

C 1.8086970 -0.7939850 -0.0456130

C 0.0155370 -2.4903090 -0.1984050

C 1.9630230 0.6011270 -0.1618760

C -2.4084770 -3.5526930 -0.1538330

H	-4.1225190	-0.7352140	0.0233940
H	-3.5595260	-1.0780110	-1.6154740
H	-1.0342900	3.2310260	1.6210680
H	-1.1560320	4.7566640	0.7485140
O	1.4251520	3.4901680	-0.2998830
H	-3.4610280	3.6304550	1.5622560
H	-3.3751010	4.1169190	-0.1277540
C	2.9279500	-1.5940300	0.1678420
O	0.6459010	-3.5275670	0.0024930
C	3.2364560	1.1573620	-0.0758380
H	-2.2901870	-4.4837670	-0.7027580
H	-2.2493740	-3.7736770	0.9031110
C	4.1991290	-1.0337330	0.2582970
H	2.7973340	-2.6621950	0.2621350
C	4.3549440	0.3546950	0.1329680
H	3.3488420	2.2271710	-0.1690930
C	5.3890630	-1.9106340	0.4868920
C	5.7131610	0.9747780	0.2250220
H	6.1181470	-1.8232430	-0.3218600
H	5.1036210	-2.9575100	0.5646860
H	5.9202690	-1.6411230	1.4025190
H	6.3915000	0.5799330	-0.5347190
H	5.6671730	2.0545270	0.1000140
H	6.1873800	0.7715410	1.1879320
C	-3.7789860	-2.8852750	-0.3839880
H	-4.2764710	-3.2877520	-1.2642300
H	-4.4453170	-3.0502600	0.4603030
C	-1.4428570	-2.4790840	-0.6044620
H	-1.4423590	-2.4150380	-1.7002370
C	-0.8997280	3.0024930	-0.5165060
H	-1.0128620	3.6545150	-1.3860840
O	-0.9927550	0.0045670	-2.4980880
H	-0.1150840	0.0776510	-2.8884630
Cl	-1.9146790	-0.4913030	2.9446580

49

M06L_LS_Ni_II_LD_G1.log

49

Final Energy = -2578.3709919700

Ni	-1.0314290	0.0723550	-0.0194880
N	-2.1876340	1.6267810	-0.3423370

N	-2.3597390	-1.3427670	0.2638740
C	-1.3361640	2.8243760	-0.6786910
C	-3.0699980	2.0205700	0.8078550
H	-2.7658480	1.4036210	-1.1450310
N	0.2561350	-1.2275340	0.2308300
N	0.4010640	1.2049050	-0.2510150
C	-1.6582920	-2.6473510	0.5435230
C	-3.3046460	-1.5907490	-0.8705650
H	-2.9102900	-1.1125030	1.0843030
C	-1.8190880	3.9401430	0.2408290
C	0.1459780	2.5313640	-0.5000060
H	-1.4741730	3.0738850	-1.7311440
C	-2.3444370	3.1811110	1.4473260
H	-3.2280290	1.1751230	1.4733390
H	-4.0404160	2.3303590	0.4181200
C	1.5471740	-0.7885320	0.1318860
C	-0.1521620	-2.5172350	0.4739710
C	1.6312150	0.6177740	-0.1410780
C	-2.2283790	-3.6509070	-0.4621430
H	-1.9081710	-2.9521270	1.5608010
H	-4.1742490	-0.9461680	-0.7664530
H	-2.7968600	-1.3350650	-1.8012180
H	-1.0131240	4.6351820	0.4642920
H	-2.6230310	4.5000960	-0.2357720
O	0.9768880	3.4150240	-0.5946900
H	-1.5187810	2.8179290	2.0632950
H	-2.9970610	3.7730990	2.0832660
C	2.7213980	-1.5369800	0.2633590
O	0.5703690	-3.4856170	0.6211450
C	2.8859910	1.2208210	-0.2754280
H	-1.5963450	-3.6880710	-1.3510400
H	-2.2648900	-4.6540670	-0.0470840
C	3.9525840	-0.9299850	0.1288180
H	2.6545950	-2.5936250	0.4677950
C	4.0362550	0.4706870	-0.1454980
H	2.9457050	2.2777770	-0.4807350
C	5.2006560	-1.7299140	0.2661290
C	5.3710290	1.1140430	-0.2896590
H	5.8047430	-1.6807810	-0.6418480
H	4.9852100	-2.7742500	0.4744820

H	5.8323760	-1.3447830	1.0688180
H	5.9459770	0.6569080	-1.0972720
H	5.2826190	2.1772250	-0.4950360
H	5.9707230	0.9894290	0.6140120
C	-3.5909090	-3.0747090	-0.8070100
H	-3.9943110	-3.4597110	-1.7403490
H	-4.3124160	-3.2875120	-0.0166920

49

M06L_LS_Ni_II_LD_G2.log

49

Final Energy = -2578.3705871100

Ni	-1.0866680	0.0000080	-0.0000120
N	-2.3306720	-1.4949310	0.2625950
N	-2.3306490	1.4949690	-0.2625960
C	-1.5633770	-2.7118640	0.7063830
C	-3.1053610	-1.8830320	-0.9701380
H	-2.9872660	-1.2430100	0.9924280
N	0.2734870	1.2137090	-0.2611760
N	0.2734690	-1.2137130	0.2611470
C	-1.5633410	2.7118940	-0.7063830
C	-3.1053080	1.8830710	0.9701550
H	-2.9872600	1.2430640	-0.9924200
C	-2.0862930	-3.8550650	-0.1506360
C	-0.0622360	-2.5148680	0.5448390
H	-1.7411180	-2.8735150	1.7698860
C	-2.4434910	-3.1548390	-1.4505880
H	-3.0876500	-1.0749470	-1.6977260
H	-4.1425160	-2.0613770	-0.6871000
C	1.5365900	0.7021500	-0.1474170
C	-0.0621980	2.5148630	-0.5449000
C	1.5365790	-0.7021710	0.1474030
C	-2.0862080	3.8550950	0.1506680
H	-1.7411060	2.8735660	-1.7698780
C	-2.4434000	3.1548550	1.4506150
H	-4.1424650	2.0614430	0.6871380
H	-3.0876020	1.0749740	1.6977300
H	-1.3376100	-4.6368390	-0.2548600
H	-2.9733810	-4.2941170	0.3052100
O	0.7138160	-3.4415150	0.6832770
H	-1.5410980	-2.9224160	-2.0202870

H	-3.0980760	-3.7369690	-2.0936910
C	2.7530210	1.3771650	-0.2898740
O	0.7138570	3.4415220	-0.6832390
C	2.7529980	-1.3772050	0.2898670
H	-1.3375020	4.6368480	0.2548910
H	-2.9732940	4.2941780	-0.3051510
H	-3.0979590	3.7369900	2.0937380
H	-1.5410020	2.9224010	2.0202940
C	3.9460260	0.6995530	-0.1471580
H	2.7493500	2.4327870	-0.5100010
C	3.9460140	-0.6996110	0.1471600
H	2.7493090	-2.4328260	0.5099930
C	5.2396680	1.4210920	-0.2958680
C	5.2396440	-1.4211700	0.2958760
H	5.8353270	1.3579460	0.6169120
H	5.0872910	2.4712030	-0.5294200
H	5.8514970	0.9804120	-1.0851770
H	5.8514770	-0.9805000	1.0851880
H	5.0872500	-2.4712790	0.5294260
H	5.8353090	-1.3580320	-0.6169020

49

M06L_LS_Ni_II_LD_G3.log

49

Final Energy = -2578.3622901800

Ni	-0.9747010	0.0662450	-0.1460290
N	-2.0910770	1.6591030	-0.2643180
N	-2.2479130	-1.3894710	0.1746380
C	-2.8072020	2.0537530	0.9844910
H	-2.7865620	1.5306750	-0.9931400
N	0.3152140	-1.2723140	-0.0143700
N	0.4749650	1.2003880	-0.2606370
C	-3.6655260	-1.5984220	-0.2077770
H	-2.2060910	-1.4675460	1.1923060
C	-1.7348320	4.0228980	0.2095130
C	0.2349160	2.5417910	-0.4468160
C	-3.0465100	3.5371590	0.8116470
H	-2.1450940	1.8559120	1.8282980
H	-3.7015070	1.4477020	1.1025330
C	1.6013970	-0.8220220	0.0374010
C	-0.1108940	-2.5820420	-0.0312280

C	1.6959440	0.6030520	-0.1097540
C	-2.4884980	-3.7412090	0.0490370
H	-4.3140370	-0.9851350	0.4112490
H	-3.7784890	-1.2779140	-1.2422980
H	-1.0078940	4.2402220	0.9922030
H	-1.8446030	4.9259600	-0.3842270
O	1.0721940	3.4233000	-0.4848930
H	-3.2907400	4.0318200	1.7485210
H	-3.8769220	3.7024190	0.1237700
C	2.7655670	-1.5821220	0.1867360
O	0.5679230	-3.5784550	0.1261190
C	2.9548740	1.2121250	-0.0992010
H	-2.3694530	-4.6183070	-0.5810330
H	-2.2560660	-4.0422300	1.0709330
C	4.0003980	-0.9682200	0.1962940
H	2.6857930	-2.6525670	0.2971430
C	4.0958580	0.4514540	0.0515980
H	3.0254440	2.2823790	-0.2108500
C	5.2393520	-1.7775740	0.3580880
C	5.4336040	1.1048120	0.0647540
H	5.9069860	-1.6547290	-0.4968950
H	5.0148780	-2.8352960	0.4639250
H	5.8095710	-1.4599890	1.2330640
H	6.0733620	0.7150960	-0.7292620
H	5.3537610	2.1810800	-0.0605470
H	5.9614750	0.9088620	1.0000580
C	-3.8940010	-3.1128960	-0.0489490
H	-4.4501700	-3.5022330	-0.8983230
H	-4.4809780	-3.3281220	0.8407650
C	-1.5840320	-2.6060090	-0.3731620
H	-1.6322320	-2.4934530	-1.4627290
C	-1.2398070	2.8414270	-0.6378110
H	-1.3816880	3.0301230	-1.7028350

61

M06L_LS_Ni_II_LD_MeCN2_G1.log

61

Final Energy = -2843.9861801700

Ni	-0.9267190	0.0687370	0.0395010
N	-2.0861900	1.6096730	-0.5174690
N	-2.2495800	-1.2182960	0.8264800

C	-1.2303770	2.6161030	-1.2407170	H	-3.3708850	-1.2024610	-0.9645530
C	-2.8440350	2.3434460	0.5464240	H	-0.8187890	4.6755340	-0.7402000
H	-2.7417220	1.2037080	-1.1756550	H	-2.4805460	4.3758580	-1.2467820
N	0.3724980	-1.2285830	0.4417400	O	1.0942660	3.1585110	-1.4102200
N	0.5181470	1.1244310	-0.5234410	H	-1.2008530	3.4582410	1.3708310
C	-1.5421600	-2.5209530	1.0843730	H	-2.6771970	4.4024270	1.2057840
C	-3.5055700	-1.5185640	0.0691060	C	2.8494210	-1.5242380	0.4029010
H	-2.4868380	-0.7993590	1.7191480	O	0.6766790	-3.3911240	1.1529420
C	-1.6431630	3.9684340	-0.6806040	C	3.0100790	1.0426570	-0.6481350
C	0.2580830	2.3329150	-1.0541250	H	-1.7966290	-3.4483480	-0.8462700
H	-1.4270040	2.5333580	-2.3106280	H	-2.1082790	-4.5351970	0.5022290
C	-2.0733250	3.6298170	0.7371000	C	4.0950080	-0.9801490	0.1035940
H	-2.9254490	1.7324800	1.4421430	H	2.7776630	-2.5220790	0.8081490
H	-3.8508570	2.5404170	0.1763230	C	4.1759260	0.3149700	-0.4277100
C	1.6819380	-0.7982150	0.1829570	H	3.0643780	2.0413440	-1.0538260
C	-0.0393920	-2.4252320	0.8986810	C	5.3368270	-1.7762680	0.3489050
C	1.7632770	0.5021070	-0.3453670	C	5.5050110	0.9175260	-0.7540790
C	-2.2287690	-3.5128570	0.1548710	H	5.9083070	-1.9257670	-0.5697110
H	-1.7282230	-2.8098390	2.1212180	H	5.1076650	-2.7562830	0.7613500
H	-4.3292940	-0.9486950	0.4920630	H	6.0096780	-1.2700740	1.0444920

H	6.0498900	0.3189580	-1.4872920
H	5.3993090	1.9221680	-1.1573350
H	6.1486810	0.9769920	0.1262070
C	-3.6667140	-3.0244320	0.1502640
H	-4.2552550	-3.4174660	-0.6751460
H	-4.1642650	-3.3057970	1.0793280
N	-0.6687410	0.8624470	2.1408710
C	-0.1231650	1.1155990	3.1231920
C	0.5537590	1.4352330	4.3464940
H	1.5386150	0.9724570	4.3563760
H	0.6687070	2.5132610	4.4370330
H	-0.0178740	1.0677700	5.1959880
N	-1.1820440	-0.7293980	-1.9611860
C	-1.0218480	-1.1207550	-3.0316600
C	-0.8285950	-1.6132740	-4.3637610
H	0.0741790	-1.1815080	-4.7906480
H	-0.7295620	-2.6966040	-4.3468970
H	-1.6789180	-1.3443460	-4.9867080

61

M06L_LS_Ni_II_LD_MeCN2_G2.log

61

Final Energy = -2843.9861309600

Ni	0.9678920	-0.0000190	-0.0000280
N	2.2234060	1.4753350	0.5365890
N	2.2234150	-1.4752840	-0.5368640
C	1.4150440	2.5989740	1.1311980
C	3.1117770	2.0671460	-0.5133260
H	2.7944590	1.0629030	1.2652780
N	-0.4105200	-1.2124470	-0.3916430
N	-0.4105310	1.2123440	0.3917530
C	1.4150340	-2.5989650	-1.1313660
C	3.1119710	-2.0670500	0.5129170
H	2.7943360	-1.0628090	-1.2656320
C	1.9628300	3.8680280	0.4943070
C	-0.0803160	2.4181220	0.8874860
H	1.5508420	2.5890450	2.2136240
C	2.4546560	3.3833640	-0.8593680
H	3.2163140	1.3833120	-1.3521470
H	4.0996730	2.2262930	-0.0797820
C	-1.6925050	-0.6724380	-0.2051930

C	-0.0803070	-2.4181740	-0.8875040
C	-1.6925120	0.6722920	0.2054020
C	1.9629380	-3.8679860	-0.4945020
H	1.5507260	-2.5890580	-2.2138040
C	2.4549310	-3.3832710	0.8590930
H	4.0997980	-2.2261950	0.0792110
H	3.2166380	-1.3831930	1.3517030
H	1.1926450	4.6342790	0.4414980
H	2.7914410	4.2624820	1.0820970
O	-0.8643980	3.3174360	1.1789920
H	1.6137270	3.2205690	-1.5365430
H	3.1434050	4.0699990	-1.3444960
C	-2.9039200	-1.3309200	-0.3979440
O	-0.8643890	-3.3174670	-1.1790730
C	-2.9039350	1.3307340	0.3982460
H	1.1927820	-4.6342570	-0.4415680
H	2.7914810	-4.2624390	-1.0823900
H	3.1437660	-4.0698760	1.3441440
H	1.6140930	-3.2204780	1.5363800
C	-4.1138400	-0.6719290	-0.2000920
H	-2.8952920	-2.3645790	-0.7082690
C	-4.1138480	0.6717070	0.2004750
H	-2.8953160	2.3643940	0.7085680
C	-5.4036250	-1.3969030	-0.4170160
C	-5.4036390	1.3966470	0.4174740
H	-6.0166570	-1.4086030	0.4867330
H	-5.2357540	-2.4283080	-0.7189100
H	-6.0110230	-0.9179900	-1.1881120
H	-6.0109790	0.9177170	1.1886050
H	-5.2357790	2.4280560	0.7193590
H	-6.0167250	1.4083310	-0.4862390
N	0.9670220	0.5958700	-2.1296180
C	0.6212460	0.8190720	-3.2047990
C	0.1951280	1.1048680	-4.5436530
H	-0.2771270	2.0843450	-4.5799600
H	1.0499260	1.0989000	-5.2164950
H	-0.5206070	0.3547650	-4.8733690
N	0.9673930	-0.5958810	2.1295120
C	0.6219130	-0.8188750	3.2048300
C	0.1962170	-1.1044170	4.5438720

H -0.2761920 -2.0838140 4.5804710

H 1.0512680 -1.0985520 5.2163930

H -0.5192800 -0.3541640 4.8737500

61

M06L_LS_Ni_II_LD_MeCN2_G3.log

61

Final Energy = -2843.9814953200

Ni	0.8817430	0.1409020	0.2781800
N	1.8898140	1.8444780	0.3115850
N	2.1561370	-1.0955500	-0.6288300
C	2.6950410	2.1724220	-0.9036210
H	2.5170880	1.7825470	1.1062210
N	-0.3425440	-1.2512860	-0.0222110
N	-0.6459870	1.1807340	0.4738270
C	3.6272380	-1.2448820	-0.5623420
H	1.8922770	-0.9261120	-1.6049740
C	1.2041360	3.9747480	-0.6012490
C	-0.5037780	2.5344170	0.5832670
C	2.6380630	3.6809370	-1.0024520
H	2.2186700	1.7051710	-1.7666210
H	3.6937100	1.7583160	-0.7972580
C	-1.6698860	-0.8909290	0.1280860
C	0.1501410	-2.4689310	-0.3920960
C	-1.8445000	0.4794160	0.4327460
C	2.5233140	-3.3678050	-1.1178590
H	4.1027420	-0.4938880	-1.1869440
H	3.9385570	-1.0756570	0.4681310
H	0.5284930	3.7651230	-1.4336080
H	1.0302320	5.0011360	-0.2908970
O	-1.4114860	3.3464010	0.6633510
H	2.8872790	4.0402600	-1.9978600
H	3.3378700	4.1326560	-0.2978440
C	-2.7848330	-1.7213530	0.0133490
O	-0.4946580	-3.4519100	-0.7229440
C	-3.1308870	0.9827130	0.6291490
H	2.5214480	-4.3918200	-0.7541940
H	2.1584740	-3.3837930	-2.1461540
C	-4.0612980	-1.2131840	0.1996530
H	-2.6438130	-2.7647260	-0.2234140
C	-4.2361170	0.1538820	0.5123500

H	-3.2608170	2.0273570	0.8642540
C	-5.2484740	-2.1096630	0.0766580
C	-5.6085140	0.7044260	0.7181250
H	-5.8223800	-2.1420880	1.0049950
H	-4.9570520	-3.1266970	-0.1734180
H	-5.9383000	-1.7570100	-0.6925900
H	-6.1280690	0.1915590	1.5300020
H	-5.5816860	1.7653390	0.9540650
H	-6.2304370	0.5725630	-0.1695550
C	3.9029410	-2.6932630	-1.0192380
H	4.5365960	-3.1998890	-0.2944670
H	4.4273730	-2.7152170	-1.9712590
C	1.6578960	-2.4515920	-0.2860350
H	1.8928450	-2.5951640	0.7760570
C	0.9393580	2.9884960	0.5391900
H	1.1791840	3.4445310	1.5007280
N	1.5748720	-0.3737690	2.2355150
C	1.8360510	-0.6318700	3.3264320
C	2.1624260	-0.9566080	4.6844690
H	1.5880090	-0.3312840	5.3645100
H	1.9291710	-2.0007510	4.8819990
H	3.2228810	-0.7911090	4.8619580
N	0.5114730	-0.2784700	-3.0565670
C	-0.5049160	0.2692650	-3.0559670
C	-1.7726830	0.9496340	-3.0492570
H	-2.5528630	0.2853430	-2.6811170
H	-1.7255110	1.8239310	-2.4014580
H	-2.0322850	1.2735590	-4.0549580

60

M06L_LS_Ni_II_LD_MeCNMeOH_G1.log

60

Final Energy = -2826.4851628900

Ni	0.9762370	0.0715650	-0.2093280
N	2.2273220	1.4381350	0.5292900
N	2.2250890	-1.4251200	-0.5774810
C	1.4360170	2.6279260	0.9972300
C	3.2881960	1.9333330	-0.3921550
H	2.6350170	0.9761130	1.3360920
N	-0.3894960	-1.1677640	-0.4659420
N	-0.3820950	1.2526160	0.2453690

C	1.4325060	-2.6790230	-0.8071320
C	3.3219420	-1.7429190	0.3796280
H	2.5895790	-1.1018690	-1.4696990
C	2.0652590	3.8333120	0.3052350
C	-0.0518890	2.4736550	0.7081930
H	1.5219910	2.6995570	2.0831150
C	2.7234260	3.2293060	-0.9246180
H	3.4817420	1.1952720	-1.1666030
H	4.2043360	2.1038250	0.1776730
C	-1.6712510	-0.6144320	-0.3582210
C	-0.0650930	-2.4475380	-0.7348840
C	-1.6660100	0.7395060	0.0321410
C	1.9553650	-3.6637870	0.2339430
H	1.6622900	-3.0517280	-1.8080820
H	4.2347570	-1.2338610	0.0781020
H	3.0347530	-1.3736990	1.3657670
H	1.3134530	4.5883650	0.0847070
H	2.8153690	4.2908790	0.9503830
O	-0.8237060	3.4004130	0.9478250
H	1.9814660	3.0065150	-1.6930610
H	3.4875840	3.8664340	-1.3633970
C	-2.8840960	-1.2610120	-0.5802280
O	-0.8432660	-3.3881140	-0.8911230
C	-2.8761330	1.4097360	0.1933510
H	1.4216080	-3.5272850	1.1780850
H	1.8116320	-4.6946630	-0.0779850
C	-4.0917160	-0.5885230	-0.4161540
H	-2.8786100	-2.2992840	-0.8764430
C	-4.0874640	0.7591530	-0.0249050
H	-2.8651650	2.4469700	0.4926880
C	-5.3840710	-1.3003020	-0.6608090
C	-5.3756330	1.4981940	0.1511680
H	-6.0217180	-1.2978000	0.2259330
H	-5.2200680	-2.3360880	-0.9498900
H	-5.9655280	-0.8215690	-1.4519250
H	-6.0128470	1.0266190	0.9025660
H	-5.2057840	2.5278850	0.4580310
H	-5.9601670	1.5170210	-0.7713350
C	3.4103840	-3.2565110	0.3874780
H	3.8743210	-3.6396420	1.2934500

H 3.9959880 -3.6093770 -0.4631380
N 1.0664980 -0.4641950 2.6722830
C 0.0314360 -0.9675710 2.7730430
C -1.2590610 -1.5944670 2.8802440
H -2.0462690 -0.8653750 2.6946220
H -1.3472770 -2.3923770 2.1438480
H -1.3938160 -2.0158630 3.8742670
O 1.3350570 0.5439700 -2.1429290
C 0.2652590 1.0386150 -2.8531730
H -0.0518890 2.0484430 -2.5339710
H 0.5217760 1.1175590 -3.9209640
H -0.6333030 0.3994670 -2.8027260

60

M06L_LS_Ni_II_LD_MeCNMeOH_G2.log

60

Final Energy = -2826.4849766600

Ni -0.9644680 0.0676350 -0.2652160
N -2.2887380 -1.3486380 0.2205730
N -2.1620080 1.6392270 -0.4894060
C -1.5545730 -2.6044750 0.5989290
C -3.2883640 -1.7074320 -0.8228020
H -2.7443660 -0.9873810 1.0543520
N 0.4405900 1.2752540 -0.3405460
N 0.3348260 -1.1863880 0.1623070
C -1.3214450 2.8437640 -0.8049400
C -3.0638770 1.9979120 0.6405020
H -2.6804800 1.3675740 -1.3181010
C -2.1197080 -3.6972020 -0.3074300
C -0.0462490 -2.4422510 0.4786840
H -1.7516550 -2.8194200 1.6508100
C -2.6873010 -2.9238260 -1.4865810
H -3.4354540 -0.8702560 -1.5007920
H -4.2381020 -1.9481430 -0.3398120
C 1.7019190 0.6768240 -0.2385050
C 0.1668380 2.5628010 -0.6331390
C 1.6433450 -0.7036800 0.0354200
C -1.8126720 3.9335400 0.1432040
H -1.4685180 3.1141980 -1.8509940
C -2.3482980 3.1406360 1.3241570
H -4.0284720 2.3214270 0.2447320

H	-3.2336060	1.1363320	1.2844800
H	-1.3495750	-4.4175340	-0.5754270
H	-2.9129450	-4.2392260	0.2074680
O	0.6955810	-3.3957480	0.7033550
H	-1.8921030	-2.6046080	-2.1617190
H	-3.4175120	-3.4882210	-2.0616600
C	2.9409120	1.2981080	-0.3726880
O	0.9829860	3.4713780	-0.7677710
C	2.8253080	-1.4271260	0.1703810
H	-1.0092990	4.6230920	0.3924370
H	-2.6152420	4.5078600	-0.3198640
H	-3.0045720	3.7127950	1.9748320
H	-1.5267680	2.7563130	1.9346090
C	4.1206180	0.5717280	-0.2385750
H	2.9778240	2.3563920	-0.5826280
C	4.0624640	-0.8030760	0.0363310
H	2.7732720	-2.4844800	0.3815540
C	5.4413970	1.2567770	-0.3887830
C	5.3202050	-1.5985750	0.1833860
H	6.0491210	1.1657360	0.5142180
H	5.3188750	2.3164730	-0.6015540
H	6.0333690	0.8204720	-1.1963150
H	5.9496900	-1.2144170	0.9890850
H	5.1078130	-2.6437950	0.3971280
H	5.9309970	-1.5605480	-0.7212900
N	-1.8202810	-0.3449600	3.0121510
C	-0.6883170	-0.1343710	3.1105640
C	0.7194220	0.1349300	3.2321390
H	1.0166450	0.8907500	2.5056720
H	0.9506080	0.4950470	4.2323700
H	1.2930000	-0.7708240	3.0402810
O	-1.2345030	-0.1386200	-2.2604060
C	-0.1361040	-0.5597040	-2.9733590
H	0.1861230	-1.5889420	-2.7298480
H	-0.3610540	-0.5545920	-4.0512070
H	0.7511360	0.0847830	-2.8451120

60

M06L_LS_Ni_II_LD_MeCNMeOH_G3.log

60

Final Energy = -2826.4777578400

Ni	-0.9475990	0.0426550	-0.3379660
N	-2.0128240	1.6801950	-0.5839290
N	-2.2467390	-1.2766620	0.3951400
C	-3.0523510	2.0469550	0.4183590
H	-2.4259050	1.4380140	-1.4818040
N	0.2880590	-1.3315170	-0.0149630
N	0.5524740	1.1351920	-0.5204930
C	-3.6989920	-1.4448060	0.1946770
H	-2.0637530	-1.1881190	1.3964010
C	-1.5001330	3.8277150	0.3488370
C	0.3695500	2.4594000	-0.6950120
C	-2.9848830	3.5581640	0.5167640
H	-2.7891870	1.5839970	1.3709870
H	-4.0166860	1.6550600	0.1043700
C	1.6178990	-0.9112940	-0.0677100
C	-0.1901280	-2.5844900	0.1380250
C	1.7695360	0.4567470	-0.3668040
C	-2.5918680	-3.6012190	0.5914530
H	-4.2472870	-0.7530980	0.8289680
H	-3.9148260	-1.2018910	-0.8457720
H	-0.9645130	3.5880660	1.2710570
H	-1.2598780	4.8549220	0.0884430
O	1.2451130	3.3201960	-0.7717930
H	-3.3885860	3.9304140	1.4555300
H	-3.5495950	4.0184670	-0.2957000
C	2.7471910	-1.6993180	0.1400120
O	0.4517720	-3.6131570	0.3432090
C	3.0493120	0.9970010	-0.4663150
H	-2.5563750	-4.5661940	0.0921130
H	-2.2888570	-3.7604140	1.6275030
C	4.0243840	-1.1533290	0.0501710
H	2.6190880	-2.7465400	0.3721560
C	4.1766840	0.2072070	-0.2583870
H	3.1595480	2.0457080	-0.6984680
C	5.2249030	-2.0159510	0.2760830
C	5.5413740	0.8100550	-0.3649120
H	5.8740590	-2.0428080	-0.6019700
H	4.9418320	-3.0396200	0.5111110
H	5.8420820	-1.6432230	1.0964970
H	6.1430310	0.3158590	-1.1309450

H	5.4911070	1.8679430	-0.6133350
H	6.1013070	0.7130580	0.5679120
C	-3.9763440	-2.9300650	0.5077250
H	-4.5859920	-3.3735770	-0.2768220
H	-4.5283160	-3.0408800	1.4384210
C	-1.6919390	-2.5764210	-0.0597990
H	-1.8563780	-2.5776500	-1.1447290
C	-1.0944190	2.8538170	-0.7540550
H	-1.2865160	3.3035840	-1.7309090
N	-0.7555910	0.3919320	2.5469180
C	0.2944200	0.8486400	2.7003780
C	1.6019200	1.4219790	2.8761300
H	2.3675880	0.6655660	2.7122280
H	1.7538500	2.2267850	2.1578760
H	1.7075970	1.8220560	3.8825070
O	-1.5152470	-0.3750830	-2.2275430
C	-0.5192170	-0.7979510	-3.0809810
H	-0.1212230	-1.7991840	-2.8372950
H	-0.9041400	-0.8604790	-4.1102970
H	0.3475520	-0.1168100	-3.1204960

57

M06L_LS_Ni_II_LD_MeCNOH_G1.log

57

Final Energy = -2787.1756062700

Ni	0.9866670	0.1030130	-0.3302600
N	2.2381850	1.4902660	0.3566950
N	2.2300780	-1.4168660	-0.6269600
C	1.4487940	2.6861140	0.8120360
C	3.2772770	1.9682790	-0.5998790
H	2.6659960	1.0504780	1.1653970
N	-0.3832020	-1.1410400	-0.5554520
N	-0.3732280	1.3031730	0.0800020
C	1.4363230	-2.6773810	-0.7857290
C	3.3508150	-1.6875920	0.3157200
H	2.5562060	-1.1077200	-1.5409140
C	2.0725300	3.8803220	0.0970420
C	-0.0411640	2.5267800	0.5351800
H	1.5416890	2.7731050	1.8961220
C	2.7029070	3.2580380	-1.1379920
H	3.4494070	1.2168930	-1.3662800

H	4.2075110	2.1432120	-0.0550890
C	-1.6649650	-0.5827320	-0.4584630
C	-0.0610310	-2.4371140	-0.7402300
C	-1.6580490	0.7817970	-0.1093060
C	1.9480700	-3.5913780	0.3221070
H	1.6741850	-3.1170040	-1.7576840
H	4.2633560	-1.2234280	-0.0516600
H	3.1054950	-1.2415020	1.2810500
H	1.3216280	4.6379980	-0.1174240
H	2.8371970	4.3391540	0.7241450
O	-0.8133020	3.4521100	0.7780180
H	1.9434910	3.0193670	-1.8842410
H	3.4580780	3.8873000	-1.6029050
C	-2.8786400	-1.2374180	-0.6499270
O	-0.8425870	-3.3818920	-0.8417180
C	-2.8680090	1.4544470	0.0443550
H	1.4239010	-3.3736100	1.2564040
H	1.7839540	-4.6399640	0.0892940
C	-4.0858080	-0.5624190	-0.4944570
H	-2.8742170	-2.2842010	-0.9143160
C	-4.0800740	0.7961060	-0.1429700
H	-2.8560280	2.4999500	0.3132670
C	-5.3791870	-1.2830620	-0.7052510
C	-5.3678990	1.5379620	0.0233430
H	-6.0057720	-1.2596110	0.1890750
H	-5.2161800	-2.3254900	-0.9700180
H	-5.9715480	-0.8256040	-1.5008080
H	-5.9995730	1.0842100	0.7901770
H	-5.1974540	2.5751670	0.3033680
H	-5.9583120	1.5331690	-0.8956260
C	3.4110770	-3.1989000	0.4335060
H	3.8772710	-3.5230220	1.3610880
H	3.9815940	-3.6246080	-0.3935550
N	1.0519780	-0.3861760	2.5024040
C	0.0135640	-0.8699450	2.6530430
C	-1.2819080	-1.4733320	2.8172650
H	-2.0635090	-0.7407500	2.6219550
H	-1.3990080	-2.2982260	2.1153020
H	-1.3972260	-1.8533670	3.8302430
O	1.3532280	0.5265720	-2.2600480

H	0.5280480	0.3424710	-2.7222780

57

M06L_LS_Ni_II_LD_MeCNOH_G3.log

57

Final Energy = -2787.1684952400

Ni	-0.9636500	0.0186040	-0.4712030
N	-2.0283150	1.6699790	-0.6373880
N	-2.2594210	-1.3221510	0.2294850
C	-3.0674010	2.0054580	0.3745060
H	-2.4335910	1.4420250	-1.5437140
N	0.2776690	-1.3640360	-0.1964850
N	0.5374990	1.1180530	-0.6192860
C	-3.7107350	-1.4868570	0.0191240
H	-2.0797140	-1.2561570	1.2329170
C	-1.5150200	3.7884290	0.3566200
C	0.3528380	2.4503350	-0.7282920
C	-2.9995100	3.5132460	0.5184390
H	-2.8057870	1.5138440	1.3130850
H	-4.0319090	1.6242950	0.0479970
C	1.6061400	-0.9370210	-0.2145620
C	-0.2005330	-2.6184180	-0.0487150
C	1.7560270	0.4395110	-0.4701100
C	-2.6019070	-3.6512750	0.3658790
H	-4.2621840	-0.8125040	0.6691190
H	-3.9237550	-1.2175010	-1.0151030
H	-0.9777800	3.5233040	1.2708730
H	-1.2759350	4.8228820	0.1255230
O	1.2288830	3.3128090	-0.7586340
H	-3.4021660	3.8573530	1.4683240
H	-3.5652020	3.9973580	-0.2793440
C	2.7354490	-1.7264100	-0.0117800
O	0.4423210	-3.6453010	0.1606380
C	3.0347270	0.9879230	-0.5326600
H	-2.5632560	-4.6028820	-0.1583760
H	-2.3036630	-3.8374890	1.3988970
C	4.0112100	-1.1727010	-0.0641750
H	2.6082750	-2.7806240	0.1865810
C	4.1621570	0.1971230	-0.3294860
H	3.1441790	2.0434980	-0.7312450
C	5.2118060	-2.0366270	0.1563020

C	5.5254840	0.8088100	-0.3948540
H	5.8777780	-2.0314330	-0.7094110
H	4.9297390	-3.0687770	0.3521560
H	5.8111000	-1.6883130	1.0003740
H	6.1383270	0.3485620	-1.1731300
H	5.4738940	1.8757070	-0.6008580
H	6.0744490	0.6755320	0.5399420
C	-3.9870000	-2.9799440	0.2932640
H	-4.5926600	-3.4041640	-0.5048840
H	-4.5427810	-3.1150530	1.2184720
C	-1.7005800	-2.6095490	-0.2548120
H	-1.8551770	-2.5784440	-1.3405290
C	-1.1108510	2.8467110	-0.7743930
H	-1.3036760	3.3246490	-1.7377720
N	-0.7465670	0.2859930	2.4059010
C	0.2974770	0.7463740	2.5868390
C	1.5974610	1.3258260	2.7946190
H	2.3709090	0.5757430	2.6385240
H	1.7584980	2.1378130	2.0863630
H	1.6805780	1.7170650	3.8065470
O	-1.5538100	-0.3305200	-2.3525210
H	-0.7549290	-0.3636360	-2.8896150

49

M06L_LS_Ni_II_Me2_OPD_G1.log

49

Final Energy = -2578.5405760200

Ni	-1.0317960	0.0907740	-0.0252870
N	-2.1860660	1.6515010	-0.3790130
N	-2.3793120	-1.3142470	0.2578830
C	-1.3164340	2.8585050	-0.6325720
C	-3.1187120	2.0072350	0.7316200
H	-2.7197690	1.4488140	-1.2167300
N	0.2317700	-1.2322280	0.2436810
N	0.3983630	1.2258800	-0.2756740
C	-1.6823340	-2.6231240	0.5331120
C	-3.3207180	-1.5499930	-0.8770790
H	-2.9321570	-1.0883740	1.0775820
C	-1.7483950	3.8923000	0.4073590
C	0.1672220	2.5235040	-0.5306110
H	-1.4927880	3.2183570	-1.6470950

C	-2.3523110	3.0455210	1.5154600
H	-3.3881160	1.1211940	1.3030360
H	-4.0356640	2.4336940	0.3186600
C	1.5515590	-0.7773290	0.1348490
C	-0.1709960	-2.4908460	0.4843320
C	1.6450710	0.5996240	-0.1543040
C	-2.2439330	-3.6099290	-0.4919130
H	-1.9540220	-2.9421210	1.5413930
H	-4.1907930	-0.9053160	-0.7707340
H	-2.8118360	-1.2816450	-1.8042370
H	-0.9054960	4.5046030	0.7200060
H	-2.5055090	4.5579050	-0.0078080
O	0.9989640	3.4214270	-0.6867360
H	-1.5681810	2.5646840	2.1049620
H	-2.9868610	3.6065730	2.1969090
C	2.7165870	-1.5261380	0.2798460
O	0.5303000	-3.4918460	0.6554600
C	2.9006910	1.1850880	-0.2945850
H	-1.6067690	-3.6258010	-1.3786810
H	-2.2779060	-4.6228790	-0.0997990
C	3.9708090	-0.9362350	0.1410340
H	2.6369180	-2.5804120	0.5009260
C	4.0637330	0.4322460	-0.1501340
H	2.9643400	2.2396980	-0.5186920
C	5.2081910	-1.7619510	0.3013500
C	5.4016670	1.0838950	-0.3048270
H	5.8250980	-1.7489330	-0.6002260
H	4.9681050	-2.7997440	0.5232670
H	5.8445720	-1.3884610	1.1070250
H	5.9886700	0.6221490	-1.1021660
H	5.3044330	2.1427400	-0.5356730
H	6.0042350	0.9956940	0.6022670
C	-3.6078070	-3.0352280	-0.8362510
H	-4.0101520	-3.4105550	-1.7745830
H	-4.3307920	-3.2594080	-0.0496360

49

M06L_LS_Ni_II_Me2_OPD_G2.log

49

Final Energy = -2578.5403465000

Ni	-1.0948930	0.0000110	-0.0000200

N	-2.3481660	1.4983480	-0.2639780
N	-2.3481920	-1.4983000	0.2639570
C	-1.5735830	2.7202130	-0.6870350
C	-3.1186700	1.8660520	0.9705310
H	-3.0038920	1.2597260	-0.9985030
N	0.2524110	-1.2290980	0.2708600
N	0.2524330	1.2290970	-0.2708960
C	-1.5736310	-2.7201680	0.6870480
C	-3.1186970	-1.8660190	-0.9705470
H	-3.0039190	-1.2596550	0.9984750
C	-2.0562970	3.8378810	0.2271800
C	-0.0672680	2.4973450	-0.5737940
H	-1.7876340	2.9278650	-1.7361810
C	-2.4117980	3.0922570	1.5021810
H	-3.1376000	1.0301540	1.6670470
H	-4.1481150	2.1006870	0.6952950
C	1.5387610	-0.6892170	0.1489460
C	-0.0673130	-2.4973410	0.5737540
C	1.5387740	0.6891960	-0.1489660
C	-2.0563910	-3.8378560	-0.2271170
H	-1.7876670	-2.9277730	1.7362060
C	-2.4118690	-3.0922710	-1.5021470
H	-4.1481540	-2.1006050	-0.6953120
H	-3.1375900	-1.0301450	-1.6670930
H	-1.2872920	4.5969670	0.3516620
H	-2.9420380	4.3194430	-0.1887640
O	0.7011640	3.4410530	-0.7755890
H	-1.5055460	2.8027310	2.0391790
H	-3.0346170	3.6630840	2.1864640
C	2.7517020	-1.3578750	0.2917300
O	0.7011080	-3.4410350	0.7756600
C	2.7517270	1.3578320	-0.2917420
H	-1.2874140	-4.5969740	-0.3515710
H	-2.9421470	-4.3193670	0.1888540
H	-3.0347090	-3.6631040	-2.1864060
H	-1.5056070	-2.8027980	-2.0391590
C	3.9630340	-0.6856110	0.1470870
H	2.7432950	-2.4138540	0.5185940
C	3.9630470	0.6855470	-0.1470890
H	2.7433410	2.4138110	-0.5186080

C	5.2537050	-1.4256090	0.3052290
C	5.2537320	1.4255220	-0.3052220
H	5.8636880	-1.3765180	-0.5998420
H	5.0849940	-2.4758710	0.5340950
H	5.8677450	-1.0053990	1.1051780
H	5.8677710	1.0053010	-1.1051650
H	5.0850410	2.4757870	-0.5340910
H	5.8637070	1.3764230	0.5998540

49

M06L_LS_Ni_II_Me2_OPD_G3.log

49

Final Energy = -2578.5312125700

Ni	-0.9795630	0.0715700	-0.1425890
N	-2.0963700	1.6721800	-0.2610950
N	-2.2615260	-1.3856070	0.1767230
C	-2.7926530	2.0726980	0.9923270
H	-2.8041500	1.5531380	-0.9791030
N	0.2910540	-1.2883480	-0.0220250
N	0.4647170	1.2167060	-0.2748060
C	-3.6871440	-1.5979760	-0.1571770
H	-2.1855620	-1.4699640	1.1913060
C	-1.7055860	4.0278750	0.2168560
C	0.2427110	2.5261470	-0.4765760
C	-3.0191150	3.5598420	0.8292980
H	-2.1230840	1.8674570	1.8290160
H	-3.6918200	1.4756480	1.1236590
C	1.6049810	-0.8151780	0.0364920
C	-0.1191220	-2.5681130	-0.0890260
C	1.7042560	0.5836680	-0.1152850
C	-2.4967170	-3.7330120	0.0781130
H	-4.3192140	-0.9954510	0.4901060
H	-3.8416540	-1.2657650	-1.1832110
H	-0.9675220	4.2260300	0.9949850
H	-1.8063960	4.9410620	-0.3640910
O	1.0763660	3.4322000	-0.5536480
H	-3.2530350	4.0564820	1.7684240
H	-3.8525990	3.7357540	0.1467840
C	2.7613250	-1.5708980	0.2054650
O	0.5540150	-3.5983680	-0.0109910
C	2.9608690	1.1827310	-0.0993210

H	-2.3850980	-4.6270160	-0.5300600
H	-2.2451170	-4.0073070	1.1039060
C	4.0166470	-0.9669570	0.2236650
H	2.6737540	-2.6418140	0.3215300
C	4.1168020	0.4230590	0.0689380
H	3.0311400	2.2538300	-0.2202370
C	5.2467030	-1.7985140	0.4073820
C	5.4554100	1.0913750	0.0855420
H	5.9309240	-1.7036610	-0.4390760
H	5.0019160	-2.8528320	0.5185460
H	5.8150490	-1.4940590	1.2893300
H	6.1091550	0.7110570	-0.7028670
H	5.3648760	2.1672250	-0.0495510
H	5.9849190	0.9179190	1.0253210
C	-3.9075080	-3.1168910	-0.0091800
H	-4.4612420	-3.5047580	-0.8616460
H	-4.4941480	-3.3448520	0.8781410
C	-1.6082430	-2.5997640	-0.3858870
H	-1.7042760	-2.4991340	-1.4733390
C	-1.2361020	2.8462190	-0.6441910
H	-1.4023200	3.0440070	-1.7044400

61

M06L_LS_Ni_II_Me2_OPD_MeCN2_G1.log

61

Final Energy = -2844.1589167200

Ni	-0.9095880	0.0672120	-0.0108690
N	-2.0916260	1.6312440	-0.1927590
N	-2.2338290	-1.3763080	0.1290440
C	-1.2479340	2.8727830	-0.3302310
C	-3.0217800	1.8514610	0.9509840
H	-2.6066350	1.4976790	-1.0586750
N	0.3792700	-1.2538960	0.1181020
N	0.5008420	1.2482220	-0.1375710
C	-1.5144010	-2.6960870	0.2173930
C	-3.2243470	-1.4762540	-0.9846730
H	-2.7236940	-1.2426150	1.0099220
C	-1.6731950	3.7781920	0.8276680
C	0.2425680	2.5582090	-0.2995030
H	-1.4552100	3.3350670	-1.2962670
C	-2.2611820	2.8075690	1.8388980

H	-3.2714310	0.9047850	1.4279980	H	2.8088690	-2.5766560	0.2545450
H	-3.9472020	2.3079660	0.5920770	C	4.1803230	0.5126860	-0.0872690
C	1.6909640	-0.7661250	0.0671540	H	3.0478460	2.3279940	-0.2755460
C	-0.0059610	-2.5341370	0.2679790	C	5.3650860	-1.6939120	0.1445130
C	1.7591800	0.6354640	-0.0811030	C	5.5061090	1.2005150	-0.1708080
C	-1.9966770	-3.4874540	-0.9964020	H	5.9816480	-1.5785070	-0.7498870
H	-1.8319480	-3.1968940	1.1349790	H	5.1451110	-2.7530160	0.2611380
H	-4.1323890	-0.9380590	-0.7193900	H	5.9935910	-1.3899590	0.9847380
H	-2.7971660	-0.9982370	-1.8684390	H	6.1007090	0.8347980	-1.0110850
H	-0.8323240	4.3584310	1.2012910	H	5.3901120	2.2757370	-0.2896320
H	-2.4386980	4.4811620	0.4984070	H	6.1098080	1.0277630	0.7231390
O	1.0588430	3.4748940	-0.4188610	C	-3.4044020	-2.9617300	-1.2173710
H	-1.4679790	2.2724130	2.3675260	H	-3.8003290	-3.1843900	-2.2057480
H	-2.8947550	3.2831530	2.5835060	H	-4.0896830	-3.3837880	-0.4799900
C	2.8695370	-1.5042790	0.1400440	N	-2.0233520	-0.9333220	3.1422440
O	0.7121070	-3.5265370	0.4131000	C	-0.8958810	-0.6882100	3.2066030
C	3.0040480	1.2551470	-0.1590630	C	0.5071190	-0.3802930	3.2985400
H	-1.3684160	-3.2560680	-1.8609050	H	1.1010830	-1.2577170	3.0472660
H	-1.9414410	-4.5597740	-0.8285560	H	0.7569220	0.4144300	2.5953260
C	4.1124980	-0.8800000	0.0645780	H	0.7588590	-0.0584960	4.3071690

N -1.8019570 1.3085460 -3.2334440
C -0.7816560 0.7660920 -3.2481240
C 0.4838060 0.0811010 -3.2749120
H 1.2786080 0.7468540 -2.9411010
H 0.4506230 -0.7748520 -2.5996950
H 0.7079990 -0.2638330 -4.2823920
55
M06L_MS_Ni_III_LD_ClMeOH_G1.log
55
Final Energy = -3153.9025114400
Ni -0.8968630 0.0305740 -0.0059620
N -2.0664400 1.5957010 -0.3507620
N -2.2108500 -1.3597930 0.5670110
C -1.2168710 2.7537580 -0.7911220
C -2.9429040 2.0666390 0.7606320
H -2.6114730 1.2671250 -1.1429180
N 0.4163260 -1.2827770 0.2163780
N 0.5445620 1.1653170 -0.3474200
C -1.5026300 -2.6739490 0.6288740
C -3.4940210 -1.5572560 -0.1692510
H -2.3581290 -1.0216180 1.5147790
C -1.7061550 3.9448080 0.0230750
C 0.2701810 2.4826100 -0.5996670
H -1.3556200 2.9004150 -1.8634850
C -2.2403140 3.2957390 1.2895520
H -3.0573760 1.2800070 1.5021060
H -3.9234140 2.3142810 0.3501740
C 1.6950250 -0.8264690 0.0851900
C 0.0035100 -2.5482150 0.5440850
C 1.7691340 0.5706810 -0.2412730
C -2.1210820 -3.4740680 -0.5106330
H -1.7372870 -3.1584970 1.5800420
H -4.3187710 -1.1691330 0.4244560
H -3.4409700 -0.9952280 -1.1014230
H -0.9002160 4.6561640 0.1898250
H -2.5053850 4.4609830 -0.5081490
O 1.0954030 3.3735840 -0.6971750
H -1.4200160 2.9955220 1.9443580
H -2.9051450 3.9397870 1.8595270
C 2.8789600 -1.5613050 0.2347450

O	0.7316140	-3.5083910	0.7291880
C	3.0213580	1.1701090	-0.4164070
H	-1.6747720	-3.1640560	-1.4575550
H	-1.9619210	-4.5418200	-0.3860090
C	4.1047600	-0.9556520	0.0609100
H	2.8175310	-2.6095290	0.4808310
C	4.1774260	0.4315880	-0.2707120
H	3.0721680	2.2189470	-0.6625850
C	5.3590450	-1.7440730	0.2149950
C	5.5066380	1.0775680	-0.4540620
H	5.9477880	-1.7358620	-0.7042840
H	5.1513320	-2.7783870	0.4750910
H	6.0018080	-1.3200030	0.9886770
H	6.0813220	0.5857100	-1.2410770
H	5.4082860	2.1286000	-0.7113450
H	6.1117660	1.0037210	0.4516420
C	-3.5783640	-3.0500570	-0.4453950
H	-4.1275410	-3.2649630	-1.3591980
H	-4.0831390	-3.5642460	0.3737490
O	-0.8729550	0.3197330	2.0777100
C	0.2651380	0.8057620	2.6638350
H	0.5086070	1.8444310	2.3722040
H	0.1772890	0.7908210	3.7603310
H	1.1693480	0.2110950	2.4277730
Cl	-1.2273290	-0.4522450	-2.5005330

55

M06L_MS_Ni_III_LD_ClMeOH_G2.log

55

Final Energy = -3153.9006001200

Ni	-0.9122370	0.0102340	-0.0214670
N	-2.1500770	1.5338660	-0.3808980
N	-2.1646270	-1.4486620	0.4856630
C	-1.3341670	2.7457090	-0.7239960
C	-3.1316620	1.9267510	0.6715370
H	-2.6158410	1.2080110	-1.2230990
N	0.4542120	-1.2434510	0.1818660
N	0.4820120	1.2262010	-0.2759110
C	-1.3709290	-2.6716230	0.8459130
C	-3.2586290	-1.8704190	-0.4335690
H	-2.5255290	-1.0294030	1.3385980

C	-1.9577960	3.8927730	0.0576910
C	0.1461410	2.5462800	-0.4293850
H	-1.3906850	2.9040390	-1.8029580
C	-2.5633720	3.1967500	1.2653860
H	-3.2403810	1.1251700	1.3979950
H	-4.0974820	2.1050130	0.1956580
C	1.7141040	-0.7340650	0.0463370
C	0.1014840	-2.5327330	0.4841450
C	1.7285610	0.6834300	-0.1869130
C	-2.0648490	-3.8330860	0.1444660
H	-1.3874770	-2.7877000	1.9316260
C	-2.7564480	-3.1645300	-1.0323860
H	-4.1665160	-2.0280480	0.1518690
H	-3.4472360	-1.0929730	-1.1710720
H	-1.2105370	4.6441960	0.3039320
H	-2.7331980	4.3733720	-0.5384070
O	0.9249010	3.4818950	-0.3967050
H	-1.7887820	2.9372020	1.9882290
H	-3.3205850	3.7910200	1.7710090
C	2.9259760	-1.4265200	0.1438210
O	0.8600140	-3.4842320	0.5299010
C	2.9558520	1.3474230	-0.3172600
H	-1.3470660	-4.6006160	-0.1368030
H	-2.7968530	-4.2888820	0.8104620
H	-3.5590740	-3.7620260	-1.4573370
H	-2.0404440	-2.9407160	-1.8253550
C	4.1259610	-0.7580820	0.0146950
H	2.9110760	-2.4908750	0.3176390
C	4.1408710	0.6508010	-0.2223940
H	2.9609040	2.4117040	-0.4923450
C	5.4117070	-1.5018060	0.1193680
C	5.4421260	1.3610770	-0.3655160
H	6.0063800	-1.3953430	-0.7898970
H	5.2475800	-2.5612850	0.2957690
H	6.0298360	-1.1126180	0.9307100
H	6.0259610	0.9557520	-1.1940520
H	5.2998020	2.4241750	-0.5391980
H	6.0600720	1.2408800	0.5263040
O	-1.0147530	0.4993070	2.0080770
C	0.0853610	0.2396580	2.7842260

H	1.0210820	0.6692970	2.3774250
H	-0.0340110	0.6783250	3.7861820
H	0.2898990	-0.8375420	2.9274510
Cl	-1.1271730	-0.3449920	-2.5695000

55

M06L_MS_Ni_III_LD_ClMeOH_G3.log

55

Final Energy = -3153.8936480200

Ni	0.8411160	0.0697580	0.1114130
N	1.9905230	1.6155130	0.5715040
N	2.0917830	-1.4173110	-0.2709200
C	3.2240840	1.9104930	-0.2140860
H	2.1967110	1.3239700	1.5250710
N	-0.4898810	-1.2439120	-0.1367610
N	-0.6089690	1.2396420	0.2728160
C	3.4829110	-1.6546810	0.1574380
H	2.0536970	-1.4014150	-1.2936690
C	1.6927250	3.6976190	-0.5433550
C	-0.3346430	2.5652860	0.4963590
C	3.1818400	3.4051000	-0.4812060
H	3.1706800	1.3487330	-1.1463870
H	4.0970980	1.5800660	0.3442190
C	-1.7589170	-0.7575510	-0.1497350
C	-0.0792410	-2.5598440	-0.1516200
C	-1.8296300	0.6594360	0.0920780
C	2.2756050	-3.7702950	-0.1788970
H	4.1640540	-1.0516590	-0.4368820
H	3.5611710	-1.3411680	1.1985310
H	1.2739570	3.3469380	-1.4887750
H	1.4383500	4.7471530	-0.4227330
O	-1.1610340	3.4558090	0.5887670
H	3.7089930	3.6715230	-1.3943560
H	3.6406310	3.9564940	0.3409320
C	-2.9391960	-1.4867930	-0.3402980
O	-0.7799420	-3.5355750	-0.3447980
C	-3.0820020	1.2849240	0.1279250
H	2.1079010	-4.6569480	0.4268330
H	2.0871100	-4.0449810	-1.2172710
C	-4.1631640	-0.8556830	-0.3006210
H	-2.8754990	-2.5484930	-0.5221420

C	-4.2348690	0.5534100	-0.0635940
H	-3.1333600	2.3469760	0.3079160
C	-5.4157400	-1.6333220	-0.5072630
C	-5.5623860	1.2261110	-0.0244440
H	-6.0836020	-1.5444970	0.3515300
H	-5.2092550	-2.6874620	-0.6700800
H	-5.9769460	-1.2591630	-1.3655960
H	-6.1965020	0.8051420	0.7582010
H	-5.4646270	2.2931760	0.1548200
H	-6.1056690	1.0850320	-0.9606540
C	3.6874190	-3.1748120	-0.0010330
H	4.1867750	-3.5824430	0.8750000
H	4.3176270	-3.3973610	-0.8588850
C	1.3820090	-2.6200260	0.2251420
H	1.3953840	-2.5070790	1.3179060
C	1.1485150	2.8501900	0.6005710
H	1.3175220	3.3683040	1.5481000
O	1.0328550	-0.2677400	2.1568830
C	-0.0708850	-0.7179930	2.8412350
H	-0.3339990	-1.7697680	2.6202570
H	0.0954320	-0.6595680	3.9271070
H	-0.9849800	-0.1292480	2.6435960
Cl	0.9855440	0.5086840	-2.4384130

52

M06L_MS_Ni_III_LD_ClOH_G1.log

52

Final Energy = -3114.5914680000

Ni	0.8784640	0.0445980	-0.1444440
N	2.0745300	1.5826250	0.2672470
N	2.1623510	-1.4002230	-0.6125850
C	1.2315240	2.7558380	0.6737120
C	3.0189460	2.0432490	-0.7919140
H	2.5687350	1.2362980	1.0848680
N	-0.4623970	-1.2478030	-0.3428970
N	-0.5503600	1.2118010	0.1496820
C	1.4342360	-2.7027410	-0.6341070
C	3.4342480	-1.5875490	0.1403070
H	2.3003270	-1.0660210	-1.5672070
C	1.7998670	3.9467200	-0.0862030
C	-0.2489400	2.5282320	0.4012450

H	1.3088480	2.8804720	1.7555260
C	2.3855670	3.3049430	-1.3330280
H	3.1423020	1.2664250	-1.5413110
H	3.9855190	2.2490090	-0.3283580
C	-1.7322510	-0.7702970	-0.2189000
C	-0.0688700	-2.5506530	-0.5357920
C	-1.7814920	0.6422330	0.0520420
C	2.0560620	-3.4925490	0.5109090
H	1.6466350	-3.2099230	-1.5793860
H	4.2662170	-1.2020760	-0.4449220
H	3.3676080	-1.0196050	1.0685750
H	1.0260190	4.6854330	-0.2836140
H	2.5810660	4.4278100	0.5019230
O	-1.0549450	3.4379240	0.4523310
H	1.5944660	3.0335080	-2.0339510
H	3.1010610	3.9389070	-1.8508820
C	-2.9274870	-1.4923420	-0.3234620
O	-0.8126780	-3.5116890	-0.6116290
C	-3.0241870	1.2696520	0.2130550
H	1.6181540	-3.1688360	1.4569980
H	1.8900250	-4.5607880	0.4007730
C	-4.1414070	-0.8589990	-0.1676220
H	-2.8865870	-2.5507790	-0.5255860
C	-4.1904240	0.5450780	0.1066440
H	-3.0561180	2.3281850	0.4168510
C	-5.4089290	-1.6309210	-0.2817410
C	-5.5085180	1.2165390	0.2733740
H	-5.9936670	-1.5691790	0.6380230
H	-5.2208320	-2.6794350	-0.4951630
H	-6.0469570	-1.2300000	-1.0715560
H	-6.0801900	0.7691920	1.0885920
H	-5.3935750	2.2768700	0.4802330
H	-6.1242240	1.1077790	-0.6214850
C	3.5151440	-3.0776360	0.4300560
H	4.0706470	-3.2866540	1.3413590
H	4.0106930	-3.6012750	-0.3888100
O	1.0281870	0.4423300	-2.1580380
H	0.1848370	0.1989720	-2.5553470
Cl	1.0958880	-0.4123030	2.4020820

52

M06L_MS_Ni_III_LD_ClOH_G3.log

52

Final Energy = -3114.5840118900

Ni	-0.8385920	0.0540810	-0.2686990
N	-1.9720670	1.6359410	-0.6075070
N	-2.0962670	-1.4262840	0.1602260
C	-3.1794420	1.9095270	0.2208450
H	-2.1930010	1.3578690	-1.5648910
N	0.4854140	-1.2797660	-0.0823210
N	0.6285920	1.2108240	-0.4193630
C	-3.5029020	-1.6467640	-0.2224510
H	-2.0211200	-1.4137210	1.1807900
C	-1.6335690	3.6816370	0.5628280
C	0.3642790	2.5550110	-0.5387640
C	-3.1251040	3.3965160	0.5262210
H	-3.0994270	1.3230340	1.1362670
H	-4.0692820	1.5949740	-0.3199260
C	1.7568220	-0.8066500	-0.0440040
C	0.0550000	-2.5898150	-0.0334650
C	1.8420290	0.6172490	-0.2396660
C	-2.3074240	-3.7770010	0.0574000
H	-4.1576150	-1.0431510	0.4003980
H	-3.6138190	-1.3227520	-1.2570410
H	-1.1959040	3.3072150	1.4903050
H	-1.3776040	4.7328530	0.4621810
O	1.1985520	3.4408330	-0.5712480
H	-3.6306300	3.6411040	1.4575020
H	-3.5998390	3.9704150	-0.2710850
C	2.9285740	-1.5505720	0.1477530
O	0.7481500	-3.5701880	0.1618600
C	3.0986210	1.2346980	-0.2281970
H	-2.1688340	-4.6602720	-0.5607360
H	-2.0896690	-4.0632070	1.0870010
C	4.1563660	-0.9274820	0.1529540
H	2.8539430	-2.6168170	0.2951390
C	4.2422010	0.4890520	-0.0374810
H	3.1617260	2.3013620	-0.3730530
C	5.3994260	-1.7198970	0.3598150
C	5.5743840	1.1529980	-0.0285090
H	6.0821810	-1.6132070	-0.4851690

H 5.1824870 -2.7763780 0.4903630
H 5.9487980 -1.3731860 1.2370330
H 6.2244400 0.7472740 -0.8060180
H 5.4877160 2.2248950 -0.1827280
H 6.0939910 0.9847520 0.9166620
C -3.7180640 -3.1662080 -0.0704860
H -4.2490080 -3.5612750 -0.9336640
H -4.3231600 -3.3902460 0.8049650
C -1.4156510 -2.6330880 -0.3662470
H -1.4539200 -2.5026910 -1.4562840
C -1.1170600 2.8595800 -0.6125250
H -1.2926730 3.4060830 -1.5431350
O -1.1948040 -0.2734770 -2.2588860
H -0.3498170 -0.2321750 -2.7195260
Cl -0.8538350 0.4251700 2.3171210
49
M06L_MS_Ni_III_LD_G1.log
49
Final Energy = -2578.1518594300
Ni -1.0589680 0.0937380 -0.0373770
N -2.1320640 1.7575000 -0.1724840
N -2.4384430 -1.3110280 0.2659100
C -1.2705440 2.8682060 -0.7073970
C -2.7591030 2.2536860 1.1100950
H -2.8664940 1.5631400 -0.8454720
N 0.1996950 -1.2688490 0.1806600
N 0.4260260 1.1696830 -0.3729070
C -1.7669790 -2.6328410 0.5382470
C -3.4279160 -1.5394190 -0.8405410
H -2.9482260 -1.0403210 1.1013450
C -1.6137890 4.0851780 0.1363190
C 0.2044760 2.5137890 -0.6254170
H -1.4957750 3.0072770 -1.7647220
C -1.9467500 3.4712760 1.4854260
H -2.7647060 1.4704880 1.8647430
H -3.7939570 2.5124320 0.8892810
C 1.4987740 -0.8644560 0.0717630
C -0.2618590 -2.5501750 0.4449990
C 1.6297460 0.5421540 -0.2207250
C -2.3807930 -3.6221990 -0.4545260

H	-2.0076810	-2.9221250	1.5616690
H	-4.2760000	-0.8734240	-0.7064630
H	-2.9452060	-1.3001820	-1.7884700
H	-0.7818960	4.7850660	0.1583740
H	-2.4784900	4.5980710	-0.2817800
O	1.0794340	3.3278820	-0.8007810
H	-1.0362420	3.1778800	2.0113820
H	-2.5021010	4.1383270	2.1384980
C	2.6432000	-1.6454570	0.2421970
O	0.4493290	-3.5160210	0.5916030
C	2.8989320	1.1136670	-0.3160200
H	-1.7727560	-3.6725370	-1.3586190
H	-2.4263190	-4.6222730	-0.0340050
C	3.8939430	-1.0692240	0.1504260
H	2.5421760	-2.6954810	0.4611540
C	4.0240580	0.3355460	-0.1285350
H	2.9945390	2.1650280	-0.5309600
C	5.1108410	-1.8927120	0.3501570
C	5.3731750	0.9460050	-0.2048530
H	5.7656170	-1.8428080	-0.5215760
H	4.8664410	-2.9330780	0.5403000
H	5.7010220	-1.5172490	1.1885080
H	5.9739530	0.4709080	-0.9826760
H	5.3228980	2.0108260	-0.4096440
H	5.9210020	0.7973600	0.7276570
C	-3.7393310	-3.0169760	-0.7601830
H	-4.1736260	-3.3908420	-1.6836000
H	-4.4441610	-3.2134470	0.0482000

61

M06L_MS_Ni_III_LD_MeCN2_G1.log

61

Final Energy = -2843.7983940800

Ni	-0.9068350	0.0469920	0.0313670
N	-2.0806470	1.5447340	-0.6025210
N	-2.2134710	-1.2966370	0.7426510
C	-1.2278760	2.5711800	-1.3004790
C	-2.9180040	2.2711150	0.4107590
H	-2.6857240	1.1044790	-1.2873360
N	0.4339660	-1.2091950	0.4636960
N	0.5450580	1.1227990	-0.5012050

C	-1.4749830	-2.5721440	1.0499860
C	-3.4085400	-1.6505910	-0.0914730
H	-2.5283200	-0.8911050	1.6179300
C	-1.7195800	3.9177040	-0.7909370
C	0.2574820	2.3585600	-1.0390780
H	-1.3591930	2.4583180	-2.3777480
C	-2.2072170	3.5894610	0.6102540
H	-3.0239250	1.6730580	1.3120060
H	-3.9100270	2.4185030	-0.0163810
C	1.7066550	-0.7678460	0.2599220
C	0.0276950	-2.4466560	0.9168520
C	1.7705790	0.5639170	-0.2931490
C	-2.0905270	-3.6080150	0.1149580
H	-1.6854200	-2.8446380	2.0858770
H	-4.2689810	-1.0846200	0.2566800
H	-3.2074660	-1.3651450	-1.1225560
H	-0.9222500	4.6563700	-0.8251620
H	-2.5403280	4.2759030	-1.4105160
O	1.0848640	3.1977800	-1.3207300
H	-1.3626640	3.4693910	1.2918250
H	-2.8644700	4.3466220	1.0285080
C	2.8913290	-1.4630060	0.5242620
O	0.7710870	-3.3701690	1.1713370
C	3.0160800	1.1341220	-0.5747500
H	-1.6120590	-3.5612480	-0.8653950
H	-1.9602270	-4.6150290	0.5002920
C	4.1108760	-0.8863040	0.2473800
H	2.8389100	-2.4555540	0.9408350
C	4.1741040	0.4342520	-0.3165300
H	3.0606950	2.1275140	-0.9904050
C	5.3684280	-1.6253460	0.5283420
C	5.4959060	1.0415460	-0.6161720
H	5.9630960	-1.7483410	-0.3786110
H	5.1714920	-2.6083000	0.9456230
H	5.9994750	-1.0742860	1.2281170
H	6.0586810	0.4264520	-1.3207790
H	5.3936180	2.0375150	-1.0363970
H	6.1112510	1.1078020	0.2827930
C	-3.5371570	-3.1558130	0.0293800
H	-4.0724710	-3.5866300	-0.8126570

H	-4.0727430	-3.4222300	0.9409850
N	-0.7667760	0.8384120	2.0398660
C	-0.4728480	1.1532670	3.1069880
C	-0.1155740	1.5539700	4.4345610
H	0.4222210	0.7489900	4.9310980
H	0.5206390	2.4356180	4.3952560
H	-1.0123760	1.7874200	5.0046050
N	-1.0220940	-0.7664880	-1.9660080
C	-0.8251630	-1.1458080	-3.0346540
C	-0.5883540	-1.6247480	-4.3631190
H	0.1187320	-0.9716740	-4.8702760
H	-0.1774980	-2.6314200	-4.3236920
H	-1.5210320	-1.6426760	-4.9228470

61

M06L_MS_Ni_III_LD_MeCN2_G2.log

61

Final Energy = -2843.7983286300

Ni	0.9397350	0.0000790	-0.0000090
N	2.1843980	-1.4731690	-0.5587350
N	2.1840810	1.4738400	0.5580500
C	1.3746330	-2.5933750	-1.1567170
C	3.0895880	-2.0772220	0.4756500
H	2.7466160	-1.0524630	-1.2901720
N	-0.4594450	1.1948250	0.4073440
N	-0.4591930	-1.1950990	-0.4069610
C	1.3741790	2.5936920	1.1565100
C	3.0883280	2.0783090	-0.4769280
H	2.7469830	1.0534220	1.2891240
C	1.9568510	-3.8706320	-0.5698700
C	-0.1136560	-2.4466880	-0.8685450
H	1.4710100	-2.5514470	-2.2427700
C	2.4643260	-3.4166130	0.7884800
H	3.1839630	-1.4133990	1.3308260
H	4.0765160	-2.1970710	0.0290420
C	-1.7103230	0.6844800	0.2277910
C	-0.1141810	2.4464810	0.8689420
C	-1.7101740	-0.6850660	-0.2272820
C	1.9556070	3.8711960	0.5694340
H	1.4710820	2.5517310	2.2425140
C	2.4622960	3.4174450	-0.7892980

H	4.0755080	2.1985610	-0.0309880
H	3.1824040	1.4145320	-1.3321710
H	1.2022690	-4.6525340	-0.5267820
H	2.7788230	-4.2269360	-1.1889720
O	-0.8976550	-3.3477710	-1.0726850
H	1.6346570	-3.2971570	1.4882020
H	3.1772410	-4.1034770	1.2359390
C	-2.9270010	1.3373900	0.4500470
O	-0.8984090	3.3473010	1.0733710
C	-2.9267080	-1.3382770	-0.4494540
H	1.2007560	4.6528690	0.5269350
H	2.7778940	4.2276770	1.1880190
H	3.1746070	4.1046140	-1.2372510
H	1.6321680	3.2976600	-1.4884250
C	-4.1173100	0.6801100	0.2308010
H	-2.9230050	2.3600000	0.7902320
C	-4.1171610	-0.6812930	-0.2301260
H	-2.9224800	-2.3608840	-0.7896470
C	-5.4089630	1.3744490	0.4668020
C	-5.4086630	-1.3759370	-0.4660660
H	-6.0099080	1.4055950	-0.4438350
H	-5.2598100	2.3923060	0.8145030
H	-6.0112410	0.8422260	1.2053250
H	-6.0110470	-0.8439090	-1.2046450
H	-5.2592870	-2.3937920	-0.8136790
H	-6.0096100	-1.4071320	0.4445650
N	0.9321470	-0.6303630	2.0653690
C	0.6998970	-0.8701460	3.1666420
C	0.4189340	-1.1799750	4.5362020
H	-0.2136720	-2.0634450	4.5913410
H	1.3469100	-1.3728700	5.0703190
H	-0.0953420	-0.3444050	5.0062720
N	0.9310530	0.6304950	-2.0655750
C	0.6981280	0.8704370	-3.1666720
C	0.4164730	1.1804440	-4.5360430
H	-0.2163890	2.0637610	-4.5907390
H	1.3441760	1.3737190	-5.0705010
H	-0.0977720	0.3448310	-5.0060640

61

M06L_MS_Ni_III_LD_MeCN2_G3.log

61

Final Energy = -2843.7880138300

Ni	-0.8434460	0.0565190	0.0183400
N	-1.9631520	1.7164040	-0.0251190
N	-2.0573790	-1.4589460	0.4952120
C	-2.5373840	2.2999230	1.2188070
H	-2.7324510	1.4918520	-0.6497100
N	0.4864640	-1.3082100	-0.0377010
N	0.6103630	1.2012670	-0.3408640
C	-3.5051520	-1.6681090	0.2580900
H	-1.8990420	-1.5274320	1.5011160
C	-1.5690210	4.1150760	0.0392140
C	0.3322910	2.5300620	-0.5839940
C	-2.8125330	3.7371570	0.8341600
H	-1.7816270	2.2437300	1.9995130
H	-3.4013420	1.7190210	1.5298300
C	1.7586100	-0.8342630	-0.1042850
C	0.0557270	-2.6228240	0.0160510
C	1.8315030	0.5957080	-0.2872990
C	-2.2862340	-3.8040080	0.3787450
H	-4.0847470	-1.0573800	0.9441230
H	-3.7220440	-1.3461570	-0.7595030
H	-0.7682900	4.4408130	0.7028690
H	-1.7420130	4.9154150	-0.6740480
O	1.1678530	3.3898430	-0.7605040
H	-2.9676130	4.3725220	1.7023500
H	-3.7075490	3.7961540	0.2138880
C	2.9361200	-1.5864470	-0.0383650
O	0.7683460	-3.5992490	0.0948470
C	3.0816470	1.2097340	-0.4037270
H	-2.2275090	-4.6764920	-0.2656080
H	-1.9419220	-4.1047150	1.3681410
C	4.1606330	-0.9667660	-0.1475710
H	2.8718350	-2.6535900	0.1023640
C	4.2340390	0.4566240	-0.3346320
H	3.1353040	2.2772960	-0.5420240
C	5.4124010	-1.7625140	-0.0660800
C	5.5612040	1.1139950	-0.4461800
H	6.0118700	-1.6461730	-0.9707340
H	5.2071440	-2.8193630	0.0756030

H	6.0420180	-1.4206670	0.7575240
H	6.1319380	0.7063970	-1.2824970
H	5.4670880	2.1866960	-0.5854930
H	6.1647560	0.9339390	0.4453380
C	-3.7000120	-3.1862920	0.4336490
H	-4.3394020	-3.5754040	-0.3545920
H	-4.1859540	-3.4079200	1.3803360
C	-1.4462380	-2.6598300	-0.1385180
H	-1.6274350	-2.5396460	-1.2132140
C	-1.1555370	2.8149280	-0.6611330
H	-1.4051550	2.8345310	-1.7234830
N	-1.3315170	-0.1645120	-2.0526260
C	-1.4860850	-0.2378640	-3.1903550
C	-1.6814040	-0.3341790	-4.6053920
H	-1.1946650	0.5020790	-5.1027240
H	-1.2541460	-1.2645360	-4.9737020
H	-2.7448650	-0.3151400	-4.8340060
N	-0.4331170	0.2225920	2.1576730
C	-0.0235990	0.2181520	3.2332680
C	0.4764240	0.2214520	4.5753880
H	1.0819440	-0.6658960	4.7472650
H	1.0881510	1.1062060	4.7387670
H	-0.3534050	0.2281180	5.2789730

60

M06L_MS_Ni_III_LD_MeCNMeOH_G1.log

60

Final Energy = -2826.3069689900

Ni	0.9160640	0.1007990	-0.1504160
N	2.0699820	1.6535440	0.3171510
N	2.2508020	-1.2348480	-0.7943820
C	1.2083950	2.7563300	0.8711160
C	2.9000840	2.2348870	-0.7845570
H	2.6729340	1.3060050	1.0546260
N	-0.3872380	-1.2070500	-0.4698640
N	-0.5375210	1.1822230	0.3137900
C	1.5427090	-2.5329440	-1.0330380
C	3.4849640	-1.5181810	-0.0020710
H	2.4667770	-0.8139660	-1.6939030
C	1.6674010	4.0214700	0.1593470
C	-0.2774210	2.4798140	0.6715090

H	1.3602210	2.8113390	1.9503310
C	2.1663160	3.4959290	-1.1764930
H	2.9935550	1.5170320	-1.5949340
H	3.8910830	2.4578360	-0.3870710
C	-1.6692570	-0.7782470	-0.2900380
C	0.0351560	-2.4214650	-0.9513890
C	-1.7562690	0.5823150	0.1695360
C	2.1416420	-3.4768930	0.0032670
H	1.7917830	-2.8891380	-2.0346350
H	4.3360300	-1.0214060	-0.4617730
H	3.3550560	-1.1093880	0.9997420
H	0.8526390	4.7379890	0.0831800
H	2.4791530	4.4911930	0.7138810
O	-1.1135670	3.3453710	0.8496990
H	1.3285160	3.2473270	-1.8308330
H	2.8063090	4.1968450	-1.7058880
C	-2.8452810	-1.5100250	-0.5019510
O	-0.6879840	-3.3513720	-1.2578270
C	-3.0121440	1.1456000	0.4161710
H	1.6611940	-3.3202510	0.9717350
H	2.0016780	-4.5177600	-0.2748170
C	-4.0754240	-0.9391520	-0.2593310
H	-2.7741210	-2.5275900	-0.8514710
C	-4.1605380	0.4095320	0.2102040
H	-3.0732550	2.1651110	0.7622280
C	-5.3218600	-1.7213170	-0.4828930
C	-5.4942770	1.0158840	0.4697000
H	-5.9075840	-1.8041120	0.4344900
H	-5.1058730	-2.7242000	-0.8402310
H	-5.9705040	-1.2311770	-1.2112900
H	-6.0507230	0.4423190	1.2133400
H	-5.4070940	2.0391710	0.8237220
H	-6.1101110	1.0167700	-0.4315970
C	3.5929830	-3.0320210	0.0457210
H	4.1232660	-3.3768640	0.9298980
H	4.1260210	-3.4052940	-0.8295700
N	1.1552270	-0.5761570	2.0166400
C	0.9116730	-0.9441140	3.0810490
C	0.6154310	-1.4081560	4.4056350
H	-0.1635450	-0.7927700	4.8505570

H 0.2722240 -2.4399380 4.3683280

H 1.5063950 -1.3548640 5.0275080

O 0.8749080 0.5336160 -2.1729370

C -0.2702030 1.0567590 -2.7172750

H -0.5341820 2.0543440 -2.3226200

H -0.1647380 1.1588880 -3.8063720

H -1.1630210 0.4232470 -2.5550620

60

M06L_MS_Ni_III_LD_MeCNMeOH_G2.log

60

Final Energy = -2826.3057836000

Ni 0.9480620 0.0348900 -0.1549200

N 2.1993830 1.5317980 0.3032950

N 2.1891100 -1.4194820 -0.6786670

C 1.3890040 2.7187230 0.7465680

C 3.1456830 2.0039610 -0.7575530

H 2.7249940 1.1838290 1.0974080

N -0.4322120 -1.1970400 -0.4161140

N -0.4386180 1.2432840 0.1933380

C 1.3895310 -2.6057710 -1.1406670

C 3.2025520 -1.9099510 0.3018850

H 2.6272440 -0.9832710 -1.4850230

C 2.0058460 3.9186440 0.0449800

C -0.0942240 2.5478060 0.4443890

H 1.4551750 2.7975380 1.8334870

C 2.5596300 3.3137220 -1.2338240

H 3.2272680 1.2559280 -1.5413950

H 4.1256020 2.1489250 -0.3011080

C -1.6875000 -0.6859150 -0.2483920

C -0.0907910 -2.4669630 -0.8094730

C -1.6898170 0.7176600 0.0696330

C 2.0371270 -3.8149980 -0.4786720

H 1.4416520 -2.6568010 -2.2296250

C 2.6418690 -3.2281760 0.7862140

H 4.1508170 -2.0520440 -0.2179390

H 3.3527220 -1.1748270 1.0897220

H 1.2623440 4.6966550 -0.1136540

H 2.8076010 4.3366570 0.6529950

O -0.8681160 3.4848270 0.4878040

H 1.7557930 3.1130580 -1.9437830

H	3.3002120	3.9410960	-1.7232810
C	-2.9039530	-1.3626720	-0.3826490
O	-0.8623980	-3.3974580	-0.9410770
C	-2.9106090	1.3841160	0.2364990
H	1.3044230	-4.5990490	-0.3017570
H	2.8167750	-4.2222980	-1.1212400
H	3.4046010	-3.8589730	1.2348350
H	1.8681120	-3.0551880	1.5368330
C	-4.0976710	-0.6925050	-0.2144460
H	-2.8979900	-2.4145640	-0.6197780
C	-4.1007220	0.7036670	0.1014900
H	-2.9069650	2.4366960	0.4708670
C	-5.3891140	-1.4169750	-0.3586290
C	-5.3954650	1.4154420	0.2793830
H	-5.9716000	-1.3716970	0.5636570
H	-5.2359620	-2.4619080	-0.6131250
H	-6.0132390	-0.9634900	-1.1307640
H	-5.9959290	0.9550770	1.0659800
H	-5.2454720	2.4612730	0.5322030
H	-6.0000360	1.3670720	-0.6283300
N	1.0662870	-0.4759920	2.1007050
C	0.7246840	-0.6540350	3.1869140
C	0.3053530	-0.8818590	4.5400460
H	0.0518670	-1.9307420	4.6784510
H	1.1060340	-0.6185990	5.2278350
H	-0.5684180	-0.2744880	4.7664180
O	1.0534600	0.6191550	-2.1236650
C	-0.0455160	0.4085990	-2.9189700
H	-0.9763220	0.8414840	-2.5069500
H	0.1006280	0.8788370	-3.9016520
H	-0.2642710	-0.6584180	-3.1042480

60

M06L_MS_Ni_III_LD_MeCNMeOH_G3.log

60

Final Energy = -2826.2976390100

Ni	-0.9440090	0.0150220	-0.2537140
N	-2.0117290	1.6304230	-0.5771540
N	-2.2323600	-1.3421340	0.4115350
C	-3.0745890	2.0399180	0.3877820
H	-2.4038700	1.3308320	-1.4692410

N	0.3186370	-1.3679000	-0.0189030
N	0.5668840	1.0828190	-0.5479350
C	-3.6797050	-1.5156270	0.1631690
H	-2.0865010	-1.2762150	1.4199850
C	-1.4935270	3.8042930	0.2885510
C	0.3656930	2.4282010	-0.7627770
C	-2.9815180	3.5522720	0.4539340
H	-2.8501570	1.5920400	1.3560130
H	-4.0350540	1.6605400	0.0488530
C	1.6129930	-0.9615870	-0.0769910
C	-0.1705840	-2.6482220	0.1551370
C	1.7587530	0.4418160	-0.3781120
C	-2.5731020	-3.6759150	0.5546450
H	-4.2483050	-0.8201860	0.7741280
H	-3.8572550	-1.2814970	-0.8860770
H	-0.9604820	3.5815990	1.2156840
H	-1.2428100	4.8213140	0.0008330
O	1.2400040	3.2612230	-0.9001900
H	-3.3826410	3.9470690	1.3840100
H	-3.5361880	4.0048440	-0.3689740
C	2.7539000	-1.7493000	0.1199900
O	0.4869910	-3.6458520	0.3728450
C	3.0410630	0.9961440	-0.4567460
H	-2.5317530	-4.6222130	0.0221180
H	-2.2818160	-3.8698600	1.5874640
C	4.0087140	-1.1891240	0.0340140
H	2.6351040	-2.7979880	0.3432690
C	4.1545410	0.2081150	-0.2579490
H	3.1486790	2.0454080	-0.6800190
C	5.2200520	-2.0255000	0.2443510
C	5.5152490	0.8007940	-0.3481360
H	5.8668360	-2.0098130	-0.6347880
H	4.9612180	-3.0582260	0.4590580
H	5.8246440	-1.6441370	1.0692190
H	6.1082820	0.3102000	-1.1222130
H	5.4756770	1.8631990	-0.5704750
H	6.0669200	0.6649850	0.5839470
C	-3.9565940	-2.9989700	0.4771410
H	-4.5746080	-3.4396510	-0.3015750
H	-4.4989630	-3.1061860	1.4134200

C -1.6649460 -2.6346330 -0.0558300

H -1.7975290 -2.6092580 -1.1446190

C -1.1015470 2.8027400 -0.7938800

H -1.3123560 3.2243520 -1.7792490

N -0.6642810 0.5658630 2.1438600

C 0.2374290 1.0407670 2.6871460

C 1.3799220 1.6435300 3.3122550

H 2.1787290 0.9106950 3.4126700

H 1.7352090 2.4679330 2.6946190

H 1.1220460 2.0250000 4.2977660

O -1.4538060 -0.4617880 -2.1598830

C -0.4549250 -0.4139880 -3.1091820

H 0.4049880 -1.0641450 -2.8776980

H -0.8488490 -0.7579590 -4.0760620

H -0.0561560 0.6001910 -3.2811430

57

M06L_MS_Ni_III_LD_MeCNOH_G1.log

57

Final Energy = -2786.9960147900

Ni 0.9071190 0.1190280 -0.2822850

N 2.0952450 1.6299610 0.2595550

N 2.2022600 -1.2854520 -0.8201060

C 1.2423710 2.7720390 0.7391980

C 3.0315410 2.1681130 -0.7770980

H 2.6228600 1.2631460 1.0439770

N -0.4302310 -1.1606550 -0.5866800

N -0.5302020 1.2523550 0.1108310

C 1.4730100 -2.5785580 -1.0021180

C 3.4283480 -1.5502840 -0.0138390

H 2.4019710 -0.8786740 -1.7335670

C 1.8038730 4.0121950 0.0581010

C -0.2359870 2.5536850 0.4453700

H 1.3166710 2.8310740 1.8267170

C 2.3851430 3.4561770 -1.2309320

H 3.1571570 1.4412420 -1.5743410

H 3.9975640 2.3503290 -0.3038760

C -1.7021800 -0.6999830 -0.4258390

C -0.0321860 -2.4360080 -0.9173710

C -1.7581040 0.6870390 -0.0357030

C 2.0687690 -3.4970450 0.0585960

H	1.7059090	-2.9734690	-1.9937690
H	4.2854640	-1.0735460	-0.4834130
H	3.2951930	-1.1107950	0.9752520
H	1.0260300	4.7587970	-0.0860020
H	2.5857690	4.4554420	0.6740600
O	-1.0470610	3.4521740	0.5480390
H	1.5927010	3.2249330	-1.9444240
H	3.0942150	4.1270090	-1.7092060
C	-2.8928870	-1.4157940	-0.5951040
O	-0.7743290	-3.3793950	-1.1111970
C	-3.0029710	1.2937960	0.1754680
H	1.5946180	-3.3098720	1.0244950
H	1.9198930	-4.5443740	-0.1886960
C	-4.1092710	-0.8022850	-0.3897800
H	-2.8468860	-2.4521180	-0.8888650
C	-4.1650120	0.5754660	0.0054410
H	-3.0401750	2.3311480	0.4675780
C	-5.3728200	-1.5636940	-0.5778770
C	-5.4857400	1.2224790	0.2274330
H	-5.9685240	-1.5724230	0.3366990
H	-5.1810940	-2.5918720	-0.8709900
H	-6.0013060	-1.1011220	-1.3412660
H	-6.0522800	0.7058350	1.0044070
H	-5.3775540	2.2631420	0.5191490
H	-6.1022020	1.1805720	-0.6723320
C	3.5240720	-3.0628850	0.0812780
H	4.0551670	-3.3845620	0.9736300
H	4.0504260	-3.4668790	-0.7843610
N	1.0283920	-0.5354570	1.9411070
C	0.7006580	-0.9118810	2.9799890
C	0.3005340	-1.3858910	4.2736460
H	-0.7124270	-1.0560680	4.4944390
H	0.3307260	-2.4732870	4.2954700
H	0.9722650	-0.9986930	5.0367700
O	1.0578970	0.6695050	-2.2294920
H	0.2212240	0.4586720	-2.6584820

57

M06L_MS_Ni_III_LD_MeCNOH_G2.log

57

Final Energy = -2786.9953462100

Ni	0.9592370	0.0618690	-0.2823590
N	2.2046890	1.5447180	0.2142420
N	2.2059320	-1.4448820	-0.6701200
C	1.3952060	2.7449450	0.6233920
C	3.1844560	1.9939640	-0.8245500
H	2.7017150	1.1955590	1.0261070
N	-0.4268520	-1.1601260	-0.5728300
N	-0.4318090	1.2801630	0.0219030
C	1.4085090	-2.6171310	-1.1625190
C	3.1485360	-1.9399270	0.3732640
H	2.6854290	-1.0238080	-1.4606500
C	2.0214140	3.9257020	-0.1058590
C	-0.0866420	2.5783480	0.3150440
H	1.4552000	2.8533520	1.7079430
C	2.6013790	3.2831970	-1.3545150
H	3.2974710	1.2247620	-1.5831950
H	4.1462180	2.1619910	-0.3378010
C	-1.6801810	-0.6467730	-0.4210590
C	-0.0821300	-2.4511080	-0.9013500
C	-1.6814360	0.7574110	-0.0953760
C	2.0006020	-3.8323040	-0.4615320
H	1.5089960	-2.6751770	-2.2474860
C	2.5420930	-3.2473050	0.8326870
H	4.1258680	-2.0978180	-0.0845970
H	3.2584790	-1.2009010	1.1641160
H	1.2789410	4.6962930	-0.3011500
H	2.8106130	4.3654610	0.5031560
O	-0.8592700	3.5141910	0.3730270
H	1.8128130	3.0510200	-2.0720040
H	3.3469570	3.8993800	-1.8504840
C	-2.8980570	-1.3229550	-0.5556600
O	-0.8609400	-3.3758280	-1.0209920
C	-2.9011600	1.4215910	0.0875670
H	1.2485640	-4.6056550	-0.3225990
H	2.8090880	-4.2523290	-1.0587250
H	3.2675740	-3.8867930	1.3284240
H	1.7289000	-3.0579730	1.5365620
C	-4.0892530	-0.6539700	-0.3765980
H	-2.8937090	-2.3732800	-0.7988280
C	-4.0908020	0.7422620	-0.0481790

H	-2.8970620	2.4724050	0.3292850
C	-5.3815820	-1.3760150	-0.5184680
C	-5.3847790	1.4512410	0.1398800
H	-5.9579840	-1.3370410	0.4078780
H	-5.2309650	-2.4188750	-0.7823450
H	-6.0098480	-0.9150240	-1.2827230
H	-5.9817110	0.9833380	0.9247100
H	-5.2349160	2.4950800	0.4003910
H	-5.9924000	1.4085810	-0.7660460
N	0.9529410	-0.4148280	2.0032350
C	0.5671650	-0.6037900	3.0727020
C	0.0943950	-0.8465520	4.4056050
H	-0.5110680	-1.7502060	4.4256180
H	0.9366640	-0.9712930	5.0828140
H	-0.5107880	-0.0087360	4.7451220
O	1.2069340	0.4842380	-2.2459100
H	0.3878330	0.2542640	-2.6983260

57

M06L_MS_Ni_III_LD_MeCNOH_G3.log

57

Final Energy = -2786.9885403900

Ni	0.8886660	0.0214870	-0.3910620
N	2.0120750	-1.5542380	-0.7864010
N	2.1538860	1.4495610	0.1848190
C	3.1416330	-1.9332500	0.1094790
H	2.3223740	-1.2101250	-1.6954210
N	-0.4250920	1.3537940	-0.1158260
N	-0.5907700	-1.0992280	-0.6699100
C	3.5670250	1.6737570	-0.1875490
H	2.0919810	1.4160380	1.2025170
C	1.5727020	-3.7157400	0.1272140
C	-0.3421780	-2.4269580	-0.9357300
C	3.0633540	-3.4445400	0.2284700
H	2.9891100	-1.4572370	1.0783020
H	4.0747190	-1.5624230	-0.3071690
C	-1.7021280	0.8955740	-0.1388910
C	0.0196830	2.6554480	0.0261810
C	-1.8006580	-0.5074750	-0.4589460
C	2.4001110	3.7993660	0.2187640
H	4.2140970	1.0273680	0.3987200

H	3.6661300	1.4052650	-1.2388300
H	1.0710990	-3.4648080	1.0644400
H	1.3219980	-4.7438610	-0.1183350
O	-1.1886760	-3.2822870	-1.1074170
H	3.5037600	-3.8062130	1.1542730
H	3.5899850	-3.9199720	-0.5998730
C	-2.8673150	1.6365110	0.0966650
O	-0.6671790	3.6259860	0.2736730
C	-3.0629180	-1.1083960	-0.5184140
H	2.2726610	4.7232910	-0.3390050
H	2.1866310	4.0183560	1.2657790
C	-4.1012960	1.0306970	0.0291920
H	-2.7827970	2.6855130	0.3339660
C	-4.2001660	-0.3663950	-0.2812890
H	-3.1362240	-2.1573610	-0.7565470
C	-5.3385320	1.8170630	0.2800560
C	-5.5392120	-1.0100190	-0.3463230
H	-6.0029700	1.7928650	-0.5856340
H	-5.1135860	2.8549440	0.5080190
H	-5.9106150	1.3992440	1.1103780
H	-6.1732310	-0.5272260	-1.0922500
H	-5.4649430	-2.0653480	-0.5922890
H	-6.0682800	-0.9155420	0.6038960
C	3.8019300	3.1791570	0.0479020
H	4.3312900	3.6141970	-0.7965360
H	4.4157910	3.3468120	0.9296040
C	1.4954520	2.6987140	-0.2807370
H	1.5493140	2.6270210	-1.3754270
C	1.1364810	-2.7528770	-0.9727590
H	1.3526790	-3.1927240	-1.9490070
N	0.7680950	-0.5728060	1.8728400
C	0.3362580	-0.9661190	2.8665380
C	-0.1944640	-1.4651440	4.1025640
H	-1.2586310	-1.2479310	4.1660870
H	-0.0497980	-2.5421630	4.1589980
H	0.3125800	-0.9962030	4.9430700
O	1.3045760	0.4861330	-2.3139860
H	0.4805290	0.4821130	-2.8131240

55

M06L_MS_Ni_II_LD_ClMeOH_G1.log

55

Final Energy = -3154.0503132500

Ni	-0.9869490	0.0584730	-0.0088540
N	-2.0606060	1.8375340	-0.4713570
N	-2.2510830	-1.5561840	0.6084230
C	-1.0781750	2.9261250	-0.7375700
C	-3.0482570	2.3281280	0.5106370
H	-2.5004550	1.5471100	-1.3367010
N	0.4512450	-1.3269450	0.2204010
N	0.5943860	1.1993320	-0.3687670
C	-1.4229150	-2.7825190	0.6550170
C	-3.5351000	-1.8770260	-0.0595180
H	-2.3774170	-1.1886580	1.5454950
C	-1.5000810	4.0804370	0.1759910
C	0.3921730	2.5291710	-0.5354460
H	-1.1404420	3.2262900	-1.7874810
C	-2.2900040	3.3891060	1.2759790
H	-3.3895530	1.5033820	1.1364620
H	-3.9167500	2.7620990	0.0044330
C	1.7110500	-0.8438570	0.0772010
C	0.0880510	-2.5635240	0.6238510
C	1.7919630	0.5661520	-0.2547690
C	-1.9217230	-3.5735770	-0.5499030
H	-1.6355950	-3.3555830	1.5652520
H	-4.3741000	-1.7078280	0.6159830
H	-3.6554570	-1.2126300	-0.9172200
H	-0.6362440	4.6404370	0.5280000
H	-2.1423700	4.7743190	-0.3679580
O	1.2415000	3.4196920	-0.5611530
H	-1.6176050	2.9037300	1.9848550
H	-2.9433270	4.0616380	1.8281880
C	2.9090120	-1.5665660	0.2184130
O	0.8190430	-3.5102140	0.9283400
C	3.0629670	1.1406300	-0.4319550
H	-1.4970870	-3.1429520	-1.4594660
H	-1.6393890	-4.6228070	-0.4976560
C	4.1421190	-0.9751740	0.0388180
H	2.8435620	-2.6148610	0.4688380
C	4.2208730	0.4042330	-0.2952760
H	3.1193180	2.1892940	-0.6818630

C	5.3922400	-1.7765410	0.1881440
C	5.5529980	1.0468480	-0.4963470
H	5.9815370	-1.7757570	-0.7313730
H	5.1745880	-2.8100020	0.4467150
H	6.0429740	-1.3644730	0.9624000
H	6.1188070	0.5583780	-1.2925900
H	5.4526280	2.0988200	-0.7526470
H	6.1730980	0.9764010	0.3999820
C	-3.4212730	-3.3321150	-0.5020890
H	-3.9151080	-3.5158250	-1.4548880
H	-3.8802820	-3.9934510	0.2351490
O	-1.0804370	0.3947250	2.0221270
C	0.0569340	0.8388910	2.6364540
H	0.3535090	1.8657580	2.3325480
H	-0.0483120	0.8617730	3.7351290
H	0.9526430	0.2169760	2.4323430
Cl	-1.4625970	-0.4577370	-2.5267780

55

M06L_MS_Ni_II_LD_ClMeOH_G2.log

55

Final Energy = -3154.0480255700

Ni	-0.9893160	0.0747390	0.1323640
N	-2.1470930	1.7790510	-0.4302060
N	-2.1880830	-1.7030260	0.3622990
C	-1.2150320	2.9063210	-0.7226470
C	-3.1879550	2.2650720	0.4983320
H	-2.5408500	1.4339020	-1.2976920
N	0.5036770	-1.2429960	0.2987190
N	0.5334320	1.3103330	-0.1785840
C	-1.2747370	-2.8497120	0.6531240
C	-3.1638550	-2.1375730	-0.6678070
H	-2.6780280	-1.4431290	1.2090740
C	-1.7204550	4.0758630	0.1273600
C	0.2678780	2.5989980	-0.4766550
H	-1.2686690	3.1541330	-1.7861110
C	-2.5003490	3.3910170	1.2378190
H	-3.5089060	1.4523870	1.1488110
H	-4.0561930	2.6387050	-0.0542560
C	1.7474840	-0.6915220	0.1773690
C	0.2118670	-2.5571770	0.3944690

C	1.7639360	0.7292490	-0.0756070
C	-1.8044550	-4.0109060	-0.1839590
H	-1.3268230	-3.0838250	1.7205640
C	-2.4909550	-3.3115210	-1.3446180
H	-4.0979420	-2.4495860	-0.1918080
H	-3.3762290	-1.3128510	-1.3469960
H	-0.8979780	4.6985150	0.4728480
H	-2.3856510	4.7088080	-0.4618060
O	1.0727000	3.5270630	-0.5929050
H	-1.8212250	2.9637500	1.9777440
H	-3.1975010	4.0510470	1.7501660
C	2.9734850	-1.3676750	0.2762010
O	0.9928530	-3.5108480	0.3474380
C	3.0055370	1.3673490	-0.2287370
H	-0.9996920	-4.6817090	-0.4762030
H	-2.5292700	-4.5893900	0.3909070
H	-3.1937560	-3.9449710	-1.8821640
H	-1.7524030	-2.9385020	-2.0569800
C	4.1827990	-0.7133370	0.1372170
H	2.9569250	-2.4298960	0.4682180
C	4.1988660	0.6787740	-0.1244920
H	3.0123830	2.4295180	-0.4214830
C	5.4663080	-1.4662470	0.2622820
C	5.4996220	1.3960420	-0.2785540
H	6.0654570	-1.3953890	-0.6482680
H	5.2913430	-2.5201220	0.4660450
H	6.0910220	-1.0678750	1.0648490
H	6.0913710	0.9848850	-1.0994280
H	5.3492850	2.4555200	-0.4726380
H	6.1202680	1.3029860	0.6154490
O	-1.3401250	0.4623150	2.0392030
C	-1.0824150	-0.4729710	3.0057980
H	-0.1219840	-1.0036050	2.8656150
H	-1.0512460	-0.0195380	4.0098540
H	-1.8493910	-1.2732790	3.0767980
Cl	-1.0944630	-0.2367790	-2.5567150

55

M06L_MS_Ni_II_LD_ClMeOH_G3.log

55

Final Energy = -3154.0449771300

Ni	0.9540750	0.0952330	0.1514300
N	1.9742970	1.9087450	0.5980800
N	2.1543740	-1.6165940	-0.3260810
C	3.1995120	2.3459780	-0.1118700
H	2.1552420	1.6178300	1.5543000
N	-0.4946370	-1.2916410	-0.0589640
N	-0.6638310	1.2731890	0.2649000
C	3.5369970	-2.0189910	-0.0418720
H	2.0175060	-1.6039610	-1.3371000
C	1.4465750	3.8708190	-0.6053890
C	-0.4747000	2.6001350	0.4652910
C	2.9591050	3.8003960	-0.4900360
H	3.3191790	1.7299410	-1.0055880
H	4.0780180	2.2020780	0.5166460
C	-1.7550830	-0.7985130	-0.0936910
C	-0.1198200	-2.6018880	-0.0584450
C	-1.8504660	0.6390420	0.0839470
C	2.0967980	-3.9875160	-0.1735100
H	4.2243470	-1.4963460	-0.7046390
H	3.7635130	-1.7141370	0.9813360
H	1.1161870	3.4057220	-1.5369940
H	1.0400740	4.8784340	-0.5610990
O	-1.3322810	3.4800840	0.5580240
H	3.4762690	4.0858310	-1.4045370
H	3.3033310	4.4665790	0.3038330
C	-2.9402810	-1.5362940	-0.2652580
O	-0.8261960	-3.5969520	-0.2003520
C	-3.1264360	1.2293790	0.0592110
H	1.9025270	-4.8246110	0.4939270
H	1.7654300	-4.2910520	-1.1680810
C	-4.1773370	-0.9293260	-0.2800020
H	-2.8611430	-2.6056040	-0.3938490
C	-4.2713370	0.4811930	-0.1172120
H	-3.1960540	2.2989640	0.1867780
C	-5.4154000	-1.7403870	-0.4698460
C	-5.6083030	1.1436100	-0.1380830
H	-6.0987060	-1.6329290	0.3752710
H	-5.1856040	-2.7969800	-0.5838450
H	-5.9744200	-1.4194650	-1.3513780
H	-6.2621160	0.7533630	0.6448020

H -5.5222150 2.2185850 0.0004080
H -6.1292930 0.9656260 -1.0812690
C 3.5736130 -3.5531320 -0.1843620
H 4.1370840 -4.0071250 0.6293670
H 4.0642370 -3.8523880 -1.1091550
C 1.3631250 -2.7250140 0.2446800
H 1.4471530 -2.5902920 1.3324190
C 0.9899640 3.0150490 0.5726500
H 1.0705530 3.6133350 1.4880030
O 1.2999690 -0.2184920 2.1380590
C 0.2294560 -0.5991890 2.9054490
H -0.1019080 -1.6443730 2.7276310
H 0.4558710 -0.5390160 3.9847510
H -0.6770560 0.0194240 2.7537630
Cl 1.1822710 0.5324090 -2.4174720
52
M06L_MS_Ni_II_LD_ClOH_G1.log
52
Final Energy = -3114.7413115100
Ni 0.9864320 0.0892850 -0.1506910
N 2.0792680 1.8439780 0.3414720
N 2.2385380 -1.5949080 -0.6041720
C 1.1087370 2.9422510 0.6144580
C 3.0674110 2.3270140 -0.6463030
H 2.5199080 1.5515220 1.2053600
N -0.4667010 -1.2773440 -0.3762190
N -0.5852500 1.2533670 0.1742420
C 1.3885690 -2.8028990 -0.6518000
C 3.4875370 -1.9153790 0.1236660
H 2.4023060 -1.2285260 -1.5382150
C 1.5677230 4.1095270 -0.2616780
C -0.3632410 2.5754320 0.3726600
H 1.1535300 3.2119550 1.6732830
C 2.3270170 3.4206240 -1.3838030
H 3.3748050 1.5044410 -1.2912250
H 3.9525450 2.7282540 -0.1424490
C -1.7247420 -0.7820260 -0.2322830
C -0.1181530 -2.5550360 -0.6549200
C -1.7901040 0.6328650 0.0727550
C 1.8430600 -3.5876020 0.5753170

H	1.6077630	-3.3953510	-1.5485520
H	4.3580080	-1.7503770	-0.5119130
H	3.5686230	-1.2492170	0.9849660
H	0.7236900	4.7113300	-0.5915070
H	2.2376380	4.7609900	0.3015120
O	-1.1989380	3.4785820	0.3970290
H	1.6348600	2.9565170	-2.0888880
H	2.9900210	4.0862520	-1.9329690
C	-2.9281750	-1.5006390	-0.3309260
O	-0.8625890	-3.5119220	-0.8797560
C	-3.0531610	1.2195660	0.2669400
H	1.4001470	-3.1401000	1.4679460
H	1.5455220	-4.6326740	0.5277260
C	-4.1544420	-0.8968870	-0.1414460
H	-2.8754020	-2.5545270	-0.5593120
C	-4.2181840	0.4890740	0.1660210
H	-3.0969700	2.2732230	0.4977720
C	-5.4119830	-1.6926290	-0.2541100
C	-5.5421950	1.1452810	0.3769980
H	-5.9842700	-1.6724650	0.6758950
H	-5.2053580	-2.7318040	-0.4984890
H	-6.0744980	-1.2900300	-1.0233430
H	-6.0986940	0.6748320	1.1904120
H	-5.4299920	2.2007200	0.6133570
H	-6.1769810	1.0642310	-0.5080760
C	3.3463700	-3.3679010	0.5649580
H	3.8146000	-3.5576950	1.5293930
H	3.8136710	-4.0358320	-0.1612540
O	1.2442530	0.4420290	-2.1303220
H	0.5256480	-0.0378610	-2.5561760
Cl	1.3166910	-0.3739270	2.4236580

52

M06L_MS_Ni_II_LD_ClOH_G2.log

52

Final Energy = -3114.7404797900

Ni	1.0190650	0.0734510	-0.1649110
N	2.1635020	1.7944230	0.3488900
N	2.2252350	-1.6836290	-0.5005730
C	1.2257550	2.9278320	0.5909970
C	3.1976060	2.2407770	-0.6077480

H	2.5643470	1.4880180	1.2272780
N	-0.4730580	-1.2467660	-0.3986460
N	-0.5146270	1.2947770	0.1211850
C	1.3225060	-2.8082940	-0.8711970
C	3.2472350	-2.1894880	0.4349540
H	2.6272090	-1.2737580	-1.3370960
C	1.7343530	4.0654970	-0.2984980
C	-0.2535850	2.6064020	0.3369550
H	1.2657080	3.2151930	1.6453170
C	2.5054620	3.3365990	-1.3867340
H	3.5114720	1.4023010	-1.2280510
H	4.0714370	2.6337640	-0.0779970
C	-1.7156210	-0.7124130	-0.2442130
C	-0.1702940	-2.5479430	-0.6078810
C	-1.7383080	0.7077490	0.0351370
C	1.8548400	-4.0196480	-0.1047900
H	1.3809280	-2.9810620	-1.9502250
C	2.5874850	-3.3936610	1.0707420
H	4.1528790	-2.4864230	-0.1042510
H	3.5103820	-1.4124900	1.1529300
H	0.9139560	4.6806080	-0.6617960
H	2.4054940	4.7131960	0.2674690
O	-1.0613640	3.5349530	0.3649170
H	1.8215890	2.8762760	-2.1023080
H	3.1991730	3.9752120	-1.9299920
C	-2.9408410	-1.3947350	-0.3290550
O	-0.9463450	-3.5042040	-0.6571550
C	-2.9818890	1.3354220	0.2251470
H	1.0485710	-4.6941190	0.1750030
H	2.5541890	-4.5800600	-0.7269330
H	3.2990680	-4.0647730	1.5477320
H	1.8775290	-3.0545410	1.8279850
C	-4.1485080	-0.7506420	-0.1497120
H	-2.9220990	-2.4534010	-0.5387690
C	-4.1695910	0.6400260	0.1374900
H	-2.9926040	2.3937440	0.4375300
C	-5.4297390	-1.5092540	-0.2527510
C	-5.4722850	1.3409540	0.3380350
H	-6.0019590	-1.4592390	0.6762050
H	-5.2547330	-2.5575280	-0.4826830

H -6.0792610 -1.0974200 -1.0281900
H -6.0419200 0.9032910 1.1606310
H -5.3267730 2.3965260 0.5550070
H -6.1109110 1.2639390 -0.5446530
O 1.4018190 0.3670200 -2.1215300
H 0.7806590 -0.2060710 -2.5843040
Cl 1.1955360 -0.3072150 2.4502670
52
M06L_MS_Ni_II_LD_ClOH_G3.log
52
Final Energy = -3114.7374236600
Ni 0.9573960 -0.0874190 -0.4025860
N 1.9870200 -1.9193850 -0.6861590
N 2.1303820 1.6338420 0.1802150
C 3.1967810 -2.2826350 0.0912470
H 2.2074450 -1.7434410 -1.6595570
N -0.4937660 1.3298990 -0.2260260
N -0.6523040 -1.2554890 -0.4145180
C 3.5300530 2.0431990 0.0186700
H 1.9232710 1.5640670 1.1774650
C 1.4769760 -3.8206050 0.6366660
C -0.4578440 -2.5979100 -0.4655640
C 2.9881670 -3.7259200 0.5215000
H 3.2564300 -1.6258680 0.9614900
H 4.0923460 -2.1296350 -0.5097130
C -1.7526160 0.8257820 -0.1369290
C -0.1236290 2.6328410 -0.1505750
C -1.8415580 -0.6156060 -0.2361960
C 2.0806230 4.0037670 0.1744230
H 4.1631380 1.4867710 0.7077510
H 3.8391510 1.7862030 -0.9959990
H 1.1357280 -3.3252720 1.5484030
H 1.0902380 -4.8367670 0.6329020
O -1.3128690 -3.4833480 -0.4358220
H 3.5080700 -3.9663660 1.4472800
H 3.3489870 -4.4125320 -0.2474060
C -2.9356180 1.5593000 0.0519760
O -0.8326200 3.6251890 0.0167600
C -3.1081090 -1.2156750 -0.1260920
H 1.9204540 4.8881840 -0.4387480

H	1.6931020	4.2318390	1.1688610
C	-4.1671160	0.9437360	0.1487240
H	-2.8622540	2.6341610	0.1255220
C	-4.2546250	-0.4706650	0.0586000
H	-3.1716900	-2.2906570	-0.1967730
C	-5.4029210	1.7559110	0.3500290
C	-5.5829810	-1.1438790	0.1625200
H	-6.1229830	1.5984100	-0.4558530
H	-5.1780670	2.8187440	0.3967650
H	-5.9188220	1.4796920	1.2721820
H	-6.2697820	-0.8025270	-0.6150680
H	-5.4893820	-2.2238420	0.0766600
H	-6.0720090	-0.9230950	1.1138230
C	3.5558060	3.5696790	0.2356350
H	4.1540910	4.0609180	-0.5302950
H	4.0030380	3.8270800	1.1942790
C	1.3754140	2.7742040	-0.3725320
H	1.5275640	2.7247150	-1.4594150
C	1.0029040	-3.0232270	-0.5761700
H	1.0654310	-3.6655090	-1.4619750
O	1.4057860	0.1361520	-2.3257040
H	0.8919780	0.8659090	-2.6847800
Cl	1.0645610	-0.4466630	2.2572090

49

M06L_MS_Ni_II_LD_G2.log

49

Final Energy = -2578.3305755300

Ni	1.2635990	0.0000920	-0.0000190
N	2.3977390	-1.7363560	-0.1056070
N	2.3974410	1.7367290	0.1055630
C	1.5733040	-2.8434490	-0.6832190
C	2.9668410	-2.2142540	1.1949420
H	3.1565540	-1.5231730	-0.7423060
N	-0.2116110	1.2231220	0.3818020
N	-0.2114220	-1.2232200	-0.3817560
C	1.5728200	2.8436690	0.6832260
C	2.9664750	2.2147690	-1.1949580
H	3.1562790	1.5236150	0.7422610
C	1.8545130	-4.0517200	0.2042010
C	0.0777290	-2.5180720	-0.7199590

H	1.8754030	-3.0154340	-1.7170110
C	2.1163900	-3.4119820	1.5576190
H	2.9439410	-1.4109450	1.9291860
H	4.0074510	-2.5055130	1.0477850
C	-1.4545190	0.6903590	0.2163550
C	0.0773080	2.5180120	0.7200210
C	-1.4544230	-0.6906310	-0.2163190
C	1.8537770	4.0519920	-0.2042070
H	1.8749210	3.0157060	1.7170100
C	2.1157880	3.4123220	-1.5576350
H	4.0070180	2.5062630	-1.0477850
H	2.9437590	1.4114700	-1.9292170
H	1.0243100	-4.7533980	0.1932260
H	2.7434570	-4.5753920	-0.1474930
O	-0.7169130	-3.3902870	-1.0298380
H	1.1779390	-3.0894530	2.0144150
H	2.6150070	-4.0722510	2.2624310
C	-2.6804030	1.3388100	0.4090770
O	-0.7175160	3.3900560	1.0299140
C	-2.6802440	-1.3392080	-0.4090570
H	1.0234310	4.7535000	-0.1932250
H	2.7426110	4.5758500	0.1474860
H	2.6142620	4.0726920	-2.2624560
H	1.1774060	3.0895880	-2.0144260
C	-3.8772230	0.6818260	0.2072120
H	-2.6774440	2.3704240	0.7237830
C	-3.8771420	-0.6823440	-0.2072250
H	-2.6771800	-2.3708160	-0.7237600
C	-5.1688280	1.3898670	0.4232270
C	-5.1686660	-1.3905190	-0.4232720
H	-5.7776370	1.3886890	-0.4828690
H	-5.0138510	2.4211860	0.7280350
H	-5.7690190	0.8943760	1.1888980
H	-5.7688870	-0.8950930	-1.1889620
H	-5.0135690	-2.4218220	-0.7280750
H	-5.7775020	-1.3894040	0.4828060

61

M06L_MS_Ni_II_LD_MeCN2_G1.log

61

Final Energy = -2843.9848783000

Ni	-0.9801220	0.0726520	0.0470050	C	-2.0259010	-3.6526430	0.0494350
N	-2.0202920	1.8152850	-0.6143450	H	-1.6030460	-3.0628700	2.0651110
N	-2.2552570	-1.4286470	0.8748840	H	-4.3685480	-1.3763290	0.6466970
C	-1.0313680	2.7371710	-1.2637700	H	-3.4614180	-1.3786350	-0.8585440
C	-2.7957250	2.6092550	0.3820450	H	-0.5107770	4.7795670	-0.7460920
H	-2.6546380	1.4747490	-1.3249990	H	-2.1450350	4.5838470	-1.3749670
N	0.4525630	-1.2847510	0.4416750	O	1.3020490	3.2081390	-1.2887570
N	0.6332520	1.1209250	-0.5325280	H	-1.1348020	3.5989340	1.3307430
C	-1.4384510	-2.6688120	1.0584320	H	-2.5145640	4.6572700	1.0590200
C	-3.5023730	-1.8032240	0.1452610	C	2.8984480	-1.5804490	0.4317820
H	-2.5033560	-1.0714750	1.7885240	O	0.7877310	-3.4643470	1.1711100
C	-1.3807330	4.1276990	-0.7450970	C	3.0947830	1.0127050	-0.6031120
C	0.4422530	2.3800250	-1.0104120	H	-1.6140950	-3.4561750	-0.9435710
H	-1.1507710	2.6730040	-2.3478680	H	-1.7905510	-4.6817540	0.3076050
C	-1.9439970	3.8344240	0.6350740	C	4.1355670	-1.0439160	0.1598410
H	-2.9870380	2.0104940	1.2713760	H	2.8181230	-2.5800700	0.8300480
H	-3.7590890	2.8954780	-0.0443770	C	4.2354390	0.2794710	-0.3718610
C	1.7133470	-0.8572060	0.2047760	H	3.1675180	2.0129390	-1.0008910
C	0.0705120	-2.5058910	0.9031530	C	5.3730930	-1.8331630	0.4152460
C	1.8151590	0.4945930	-0.3319870	C	5.5759270	0.8569860	-0.6704540

H	5.9566150	-1.9655110	-0.4979860
H	5.1434890	-2.8167890	0.8162070
H	6.0315890	-1.3231820	1.1211950
H	6.1205320	0.2461430	-1.3930950
H	5.4961030	1.8643540	-1.0703200
H	6.2013930	0.8946210	0.2235920
C	-3.5061130	-3.3203400	0.0878950
H	-4.0584370	-3.7037870	-0.7674150
H	-3.9587490	-3.7373160	0.9892040
N	-0.8007960	0.8569280	2.0350720
C	-0.5351160	1.2368590	3.0885560
C	-0.2049030	1.7236600	4.3970780
H	0.7866940	1.3794050	4.6833780
H	-0.2154830	2.8118410	4.3997860
H	-0.9281800	1.3617040	5.1245760
N	-1.2603280	-0.6972300	-1.9259420
C	-1.2561170	-1.0791590	-3.0117970
C	-1.2541120	-1.5626490	-4.3625340
H	-1.2586800	-0.7260620	-5.0579780
H	-0.3653470	-2.1643800	-4.5405300
H	-2.1359660	-2.1745520	-4.5403960

61

M06L_MS_Ni_II_LD_MeCN2_G2.log

61

Final Energy = -2843.9847414100

Ni	-1.0143010	0.0004580	0.0003040
N	-2.1801240	1.6721050	-0.6514320
N	-2.1808980	-1.6706960	0.6520350
C	-1.2493770	2.7168950	-1.1917360
C	-3.0903610	2.3377550	0.3241950
H	-2.7316580	1.3068500	-1.4168570
N	0.5176970	-1.2244620	0.4395780
N	0.5182950	1.2243390	-0.4397330
C	-1.2505470	-2.7162370	1.1916030
C	-3.0921190	-2.3355630	-0.3232330
H	-2.7318040	-1.3053290	1.4178590
C	-1.7533440	4.0379800	-0.6200420
C	0.2356650	2.4814030	-0.8748120
H	-1.3048730	2.7078630	-2.2827390
C	-2.3698940	3.6134540	0.7018050

H	-3.2890030	1.6758490	1.1655180
H	-4.0435740	2.5664360	-0.1565380
C	1.7432420	-0.6887340	0.2394760
C	0.2344200	-2.4817830	0.8735330
C	1.7435930	0.6879570	-0.2397700
C	-1.7559730	-4.0368960	0.6202870
H	-1.3052050	-2.7071300	2.2826520
C	-2.3729450	-3.6118850	-0.7012060
H	-4.0453190	-2.5634030	0.1579200
H	-3.2905430	-1.6734730	-1.1644600
H	-0.9467550	4.7610770	-0.5288810
H	-2.5165450	4.4644270	-1.2716140
O	1.0278860	3.3964960	-1.0691640
H	-1.5867200	3.4048980	1.4348100
H	-3.0367390	4.3563530	1.1328230
C	2.9800660	-1.3204430	0.4648700
O	1.0260640	-3.3977490	1.0660520
C	2.9807440	1.3190000	-0.4652040
H	-0.9500440	-4.7606660	0.5286580
H	-2.5191490	-4.4626600	1.2723380
H	-3.0406700	-4.3542300	-1.1318170
H	-1.5900240	-3.4040260	-1.4346830
C	4.1733150	-0.6744460	0.2396390
H	2.9760700	-2.3389450	0.8209120
C	4.1736620	0.6723700	-0.2399760
H	2.9772750	2.3375010	-0.8212520
C	5.4665530	-1.3707610	0.4895800
C	5.4672620	1.3680180	-0.4898830
H	6.0696800	-1.4286700	-0.4186710
H	5.3107430	-2.3815240	0.8572700
H	6.0739130	-0.8324530	1.2198180
H	6.0743540	0.8294030	-1.2201180
H	5.3119840	2.3788680	-0.8575600
H	6.0704040	1.4255940	0.4183790
N	-1.0698830	0.6685470	2.0311150
C	-0.9430790	0.9855950	3.1302440
C	-0.7876960	1.3935900	4.4968490
H	-0.7007620	2.4768740	4.5514610
H	-1.6496580	1.0789860	5.0811910
H	0.1087140	0.9457290	4.9203850

N	-1.0709410	-0.6678590	-2.0305770
C	-0.9448830	-0.9855330	-3.1296110
C	-0.7905180	-1.3944100	-4.4960670
H	-0.7116710	-2.4783120	-4.5507610
H	-1.6492020	-1.0732240	-5.0816400
H	0.1098780	-0.9532500	-4.9181750

61

M06L_MS_Ni_II_LD_MeCN2_G3.log

61

Final Energy = -2843.9785804000

Ni	-0.9472740	0.1066540	0.0078360
N	-1.8988160	2.0125580	-0.0875560
N	-2.1463970	-1.5968930	0.5320340
C	-2.4534550	2.6832280	1.1087340
H	-2.6581150	1.9179600	-0.7532740
N	0.4743160	-1.3605290	-0.0493650
N	0.7028070	1.2132070	-0.3592530
C	-3.5666240	-1.9480990	0.3425410
H	-1.9540220	-1.6610620	1.5305120
C	-1.2102780	4.3464360	-0.0240200
C	0.5455150	2.5561830	-0.5246260
C	-2.5241550	4.1453300	0.7200320
H	-1.7580020	2.5368620	1.9358530
H	-3.4018710	2.2254530	1.3821470
C	1.7429560	-0.9066550	-0.1250570
C	0.0611500	-2.6596530	0.0530150
C	1.8721840	0.5352810	-0.3004020
C	-2.1879400	-3.9665290	0.3826420
H	-4.1856900	-1.3951590	1.0450840
H	-3.8480280	-1.6391590	-0.6649120
H	-0.4062390	4.5957650	0.6687780
H	-1.2517950	5.1467150	-0.7585130
O	1.4237530	3.4060420	-0.6143900
H	-2.6342370	4.8101500	1.5744720
H	-3.3768780	4.3140760	0.0595730
C	2.9128890	-1.6871270	-0.0750680
O	0.7455340	-3.6642070	0.1949790
C	3.1646140	1.0805200	-0.4092980
H	-2.0751050	-4.8157680	-0.2870470
H	-1.7887650	-4.2694540	1.3516790

C	4.1620960	-1.1229900	-0.1833720
H	2.8094590	-2.7537740	0.0544340
C	4.2901060	0.2910200	-0.3540410
H	3.2604280	2.1471280	-0.5397250
C	5.3833330	-1.9742260	-0.1233920
C	5.6440610	0.9017030	-0.4711870
H	5.9776160	-1.8820680	-1.0346250
H	5.1324630	-3.0228630	0.0124860
H	6.0395340	-1.6723350	0.6952700
H	6.1961210	0.4872600	-1.3169870
H	5.5856630	1.9792100	-0.6001420
H	6.2510150	0.6976510	0.4131410
C	-3.6434850	-3.4775530	0.5012530
H	-4.2824090	-3.9147090	-0.2635580
H	-4.0722050	-3.7521760	1.4630920
C	-1.4475830	-2.7387000	-0.1106110
H	-1.6318580	-2.6182970	-1.1861270
C	-0.9167580	2.9823980	-0.6710390
H	-1.0919160	3.0164540	-1.7494320
N	-1.4899250	-0.1178680	-2.0205280
C	-1.7435590	-0.2010400	-3.1398920
C	-2.0574550	-0.3084430	-4.5355230
H	-1.6689570	0.5548610	-5.0715600
H	-1.6103490	-1.2102810	-4.9483060
H	-3.1356010	-0.3537040	-4.6731130
N	-0.5879270	0.3034860	2.1220770
C	-0.2185290	0.3272730	3.2120540
C	0.2397460	0.3617010	4.5712150
H	0.8655600	-0.5046760	4.7746880
H	0.8210530	1.2648180	4.7445950
H	-0.6088650	0.3520490	5.2518020

60

M06L_MS_Ni_II_LD_MeCNMeOH_G1.log

60

Final Energy = -2826.4658087700

Ni	0.9480040	0.1810040	-0.1322820
N	1.9170950	2.0340190	0.3321260
N	2.2734420	-1.2818800	-0.9927090
C	0.8741190	3.0095670	0.7760090
C	2.6965000	2.6553600	-0.7681740

H	2.5310950	1.8278550	1.1085430
N	-0.4130170	-1.2847700	-0.3960510
N	-0.6844570	1.1695080	0.4369910
C	1.5063200	-2.5388220	-1.1325730
C	3.6023020	-1.6018590	-0.4224500
H	2.3325280	-0.8123380	-1.8898400
C	1.1094280	4.2609340	-0.0698870
C	-0.5729530	2.5013540	0.6658860
H	1.0138100	3.2271300	1.8380860
C	1.7671170	3.7099860	-1.3243880
H	2.9719280	1.8957340	-1.4986900
H	3.6091180	3.1117100	-0.3745250
C	-1.6879850	-0.9018940	-0.1409850
C	-0.0123380	-2.3855870	-1.0737180
C	-1.8400900	0.4586880	0.3417200
C	2.0378720	-3.3828840	0.0212820
H	1.7413330	-3.0359470	-2.0800100
H	4.3899500	-1.4427140	-1.1595460
H	3.8041910	-0.9280390	0.4127860
H	0.1792920	4.7957360	-0.2479670

H	1.7904580	4.9400140	0.4448450
O	-1.4838210	3.3154040	0.8094140
H	1.0263080	3.2337540	-1.9686670
H	2.2896350	4.4649620	-1.9080270
C	-2.8421750	-1.6920200	-0.2913770
O	-0.7152470	-3.2554360	-1.5903160
C	-3.1320870	0.9167300	0.6524690
H	1.5635280	-3.0597860	0.9501420
H	1.8195680	-4.4401970	-0.1108340
C	-4.0965090	-1.2150530	0.0218560
H	-2.7244630	-2.7012210	-0.6567880
C	-4.2442480	0.1149850	0.5054910
H	-3.2429070	1.9275820	1.0143100
C	-5.2974180	-2.0858570	-0.1380360
C	-5.5998120	0.6342290	0.8504860
H	-5.8267180	-2.2174660	0.8080340
H	-5.0285250	-3.0703770	-0.5128810
H	-6.0207210	-1.6475290	-0.8288500
H	-6.0784640	0.0258490	1.6207670
H	-5.5540290	1.6589070	1.2110920

H -6.2699840 0.6096540 -0.0114530

C 3.5250640 -3.0614460 0.0261930

H 3.9888890 -3.2216180 0.9981600

H 4.0441570 -3.7034540 -0.6859850

N 1.5411020 -0.4655630 1.8474470

C 1.8643360 -0.9074360 2.8608770

C 2.2697570 -1.4795630 4.1138780

H 1.4802470 -1.3640120 4.8533960

H 2.4775410 -2.5403120 3.9841620

H 3.1687480 -0.9873220 4.4786020

O 0.8024430 0.6393940 -2.1327980

C -0.4104090 1.0195220 -2.6369310

H -0.7838040 1.9784230 -2.2213110

H -0.3779130 1.1464700 -3.7320730

H -1.2258100 0.2927430 -2.4423780

60

M06L_MS_Ni_II_LD_MeCNMeOH_G2.log

60

Final Energy = -2826.4663051500

Ni -1.1308290 -0.0770880 -0.3166050

N -2.3833320 -1.4659650 0.6798120

N -2.1154080 1.8124070 -0.3198450

C -1.5926790 -2.6988900 0.9792920

C -3.6016120 -1.8715900 -0.0591280

H -2.6211400 -1.0021280 1.5479580

N 0.4773310 1.0699300 -0.6030510

N 0.2673010 -1.4192720 0.0883030

C -1.1386120 2.8513340 -0.7758680

C -2.7822990 2.3237390 0.9152110

H -2.8155800 1.6859860 -1.0406110

C -2.3240500 -3.8338630 0.2512550

C -0.1104550 -2.6095030 0.6054670

H -1.5945140 -2.8765580 2.0573040

C -3.1561570 -3.1130400 -0.7974950

H -3.9225640 -1.0627280 -0.7147350

H -4.4145500 -2.1011800 0.6360040

C 1.6660110 0.3958360 -0.4985090

C 0.3226290 2.4122380 -0.5825380

C 1.5481330 -0.9904070 -0.1250290

C -1.4844560 4.0988100 0.0198340

H	-1.2656320	3.0015400	-1.8508710
C	-1.9543780	3.5199000	1.3433610
H	-3.8040910	2.6234550	0.6776320
H	-2.8273850	1.5404000	1.6704180
H	-1.6238480	-4.5595240	-0.1568650
H	-2.9745890	-4.3649890	0.9466220
O	0.5972100	-3.5939680	0.8182800
H	-2.5391840	-2.8177510	-1.6475130
H	-3.9902590	-3.7051830	-1.1673730
C	2.9439570	0.9334960	-0.7002450
O	1.2005810	3.2657490	-0.4477360
C	2.7221780	-1.7391280	0.0448900
H	-0.6273570	4.7618910	0.1058700
H	-2.2934400	4.6444860	-0.4671420
H	-2.5223120	4.2188850	1.9529090
H	-1.0954040	3.1941410	1.9348730
C	4.0865480	0.1704290	-0.5485770
H	3.0267080	1.9718840	-0.9847000
C	3.9729920	-1.1881050	-0.1621470
H	2.6305520	-2.7764890	0.3289740

C	5.4316270	0.7680880	-0.7964280
C	5.1999640	-2.0220770	0.0032660
H	6.0695390	0.7036660	0.0876080
H	5.3564940	1.8149900	-1.0805340
H	5.9646950	0.2413260	-1.5908900
H	5.8700450	-1.6049940	0.7580480
H	4.9538580	-3.0392560	0.2983080
H	5.7804450	-2.0703710	-0.9205420
N	-0.6627860	0.3722330	2.4737910
C	0.3749400	0.8734240	2.5600660
C	1.6655170	1.5038320	2.6437760
H	1.7767030	2.2253440	1.8322480
H	1.7748070	2.0195170	3.5958070
H	2.4539120	0.7582990	2.5526330
O	-1.6950750	-0.5336850	-2.0948670
C	-1.5374410	0.3879260	-3.1021200
H	-0.5452530	0.8719100	-3.0905420
H	-1.6606560	-0.0763600	-4.0908730
H	-2.2744220	1.2134730	-3.0636010

60

M06L_MS_Ni_II_LD_MeCNMeOH_G3.log

60

Final Energy = -2826.4602255300

Ni	0.9490280	0.0125450	-0.2794460
N	2.0115480	-1.7380680	-0.8936160
N	2.1116430	1.7397210	0.2959030
C	3.2439320	-2.2180610	-0.2240250
H	2.1922730	-1.3610770	-1.8186920
N	-0.5423350	1.3244450	0.0848350
N	-0.6448120	-1.2106260	-0.4681580
C	3.4596780	2.2065450	-0.0647400
H	2.0473670	1.7358170	1.3124210
C	1.5306070	-3.8326070	0.0959690
C	-0.4252510	-2.4994500	-0.8232160
C	3.0406270	-3.7098870	-0.0051740
H	3.3557090	-1.6966800	0.7297620
H	4.1214170	-1.9843270	-0.8253950
C	-1.7855180	0.7896940	0.0975460
C	-0.2109000	2.6476630	0.1617290
C	-1.8441190	-0.6285000	-0.2153170
C	1.9608100	4.1135370	0.2218300
H	4.2102040	1.6824230	0.5235240
H	3.6174540	1.9516890	-1.1135850
H	1.1819690	-3.4976680	1.0764080
H	1.1517690	-4.8391040	-0.0629950
O	-1.2602550	-3.3844570	-1.0136650
H	3.5663170	-4.0812580	0.8724600
H	3.4002030	-4.2746000	-0.8673140
C	-2.9878640	1.4737070	0.3549110
O	-0.9432360	3.6020760	0.4012040
C	-3.1036590	-1.2532410	-0.2359340
H	1.7028140	4.9607510	-0.4099260
H	1.6714470	4.3775530	1.2404510
C	-4.2066090	0.8333450	0.3267940
H	-2.9359260	2.5275020	0.5843870
C	-4.2647960	-0.5581230	0.0269830
H	-3.1468780	-2.3062410	-0.4687880
C	-5.4628570	1.5855850	0.6114000
C	-5.5829000	-1.2555090	-0.0008170
H	-6.1543110	1.5436390	-0.2327310

H	-5.2602420	2.6313800	0.8285310
H	-6.0002480	1.1611470	1.4619180
H	-6.2587090	-0.8051190	-0.7307630
H	-5.4718520	-2.3081970	-0.2484240
H	-6.0922670	-1.1865070	0.9627240
C	3.4508590	3.7323790	0.1425530
H	3.9586640	4.2401030	-0.6756920
H	3.9753540	4.0086820	1.0557550
C	1.2514430	2.8393780	-0.1988690
H	1.2922340	2.7358680	-1.2920640
C	1.0492330	-2.8638110	-0.9811990
H	1.1383330	-3.3585430	-1.9547990
N	1.1545200	-0.6177590	1.8151170
C	1.1435180	-1.0461470	2.8843480
C	1.1308000	-1.5966070	4.2107370
H	0.2195880	-1.3056660	4.7289910
H	1.1746210	-2.6829910	4.1604830
H	1.9876780	-1.2361580	4.7757820
O	1.2036280	0.4862730	-2.2351820
C	0.0963880	0.8841390	-2.9415350
H	-0.2735060	1.8924470	-2.6602510
H	0.2974580	0.9299670	-4.0253330
H	-0.7772950	0.2145770	-2.8236110

57

M06L_MS_Ni_II_LD_MeCNOH_G1.log

57

Final Energy = -2787.1566889000

Ni	0.9753240	0.2252360	-0.3085760
N	1.9832770	2.0189120	0.2730460
N	2.3064320	-1.3665720	-0.8753160
C	0.9540610	3.0201550	0.6900330
C	2.8407910	2.6416680	-0.7673920
H	2.5458200	1.7666100	1.0745900
N	-0.4114310	-1.2104880	-0.5823630
N	-0.6534060	1.2662220	0.1627980
C	1.5150880	-2.6109540	-1.0192520
C	3.5679220	-1.6808100	-0.1655590
H	2.4585760	-0.9342010	-1.7820150
C	1.2804170	4.2849400	-0.1046040
C	-0.5025370	2.5749410	0.4873840

H	1.0416290	3.2015690	1.7643380
C	1.9738100	3.7392490	-1.3415160
H	3.1289420	1.8911660	-1.5012500
H	3.7439420	3.0585650	-0.3127970
C	-1.6891540	-0.7954260	-0.3797060
C	-0.0018590	-2.4538840	-0.9308270
C	-1.8241670	0.5879580	0.0358400
C	2.0717500	-3.5138050	0.0785170
H	1.7189270	-3.0794280	-1.9888370
H	4.4272650	-1.3355620	-0.7392050
H	3.5804680	-1.1503960	0.7898950
H	0.3835270	4.8650260	-0.3091820
H	1.9641150	4.9185920	0.4621280
O	-1.3890650	3.4107710	0.6508820
H	1.2489640	3.2940190	-2.0257590
H	2.5465890	4.4879720	-1.8847640
C	-2.8544300	-1.5681400	-0.5221530
O	-0.6977110	-3.4448580	-1.1557890
C	-3.1139280	1.0882060	0.2895330
H	1.6340290	-3.2353560	1.0406820
H	1.8413720	-4.5612950	-0.0990390
C	-4.1070950	-1.0476020	-0.2743570
H	-2.7493690	-2.5958410	-0.8350230
C	-4.2391270	0.3061510	0.1427630
H	-3.2108190	2.1172770	0.6000650
C	-5.3227780	-1.8958220	-0.4412240
C	-5.5925610	0.8717930	0.4154760
H	-5.8918780	-1.9680030	0.4879630
H	-5.0655350	-2.9035230	-0.7579720
H	-6.0068240	-1.4735610	-1.1804040
H	-6.1063720	0.3188260	1.2046980
H	-5.5348420	1.9143340	0.7184810
H	-6.2376030	0.8105480	-0.4635050
C	3.5546870	-3.1875490	0.0502150
H	4.0822770	-3.4874850	0.9536910
H	4.0283770	-3.6971990	-0.7907870
N	1.2507380	-0.4076230	1.8125390
C	1.2147460	-0.8524190	2.8746290
C	1.1723060	-1.4225980	4.1922570
H	0.2332860	-1.1704000	4.6802680

H 1.2555670 -2.5060160 4.1293000

H 1.9947720 -1.0414570 4.7936620

O 1.1457300 0.7040080 -2.2558830

H 0.4471820 0.2097770 -2.6980410

57

M06L_MS_Ni_II_LD_MeCNOH_G2.log

57

Final Energy = -2787.1565115300

Ni 1.0412990 0.1497850 -0.3440280

N 2.1644470 1.8844810 0.2230440

N 2.2724290 -1.5818210 -0.7339400

C 1.1998350 2.9538060 0.6255220

C 3.0666570 2.4438440 -0.8156470

H 2.7035750 1.6019020 1.0306340

N -0.4330160 -1.1954080 -0.6000190

N -0.5158390 1.3145130 0.0684450

C 1.3865460 -2.6992520 -1.1677680

C 3.2613390 -2.1131200 0.2263270

H 2.7079450 -1.1540290 -1.5430000

C 1.6047170 4.1848880 -0.1871000

C -0.2813260 2.6017300 0.4239480

H 1.2969780 3.1447580 1.6971800

C 2.2657120 3.5779690 -1.4128440

H 3.3208570 1.6684270 -1.5352450

H 3.9852490 2.8197420 -0.3562030

C -1.6825230 -0.6932730 -0.4064400

C -0.1087850 -2.4796310 -0.8784400

C -1.7291260 0.7075740 -0.0380750

C 1.9371090 -3.9395280 -0.4665160

H 1.4453190 -2.8072790 -2.2542570

C 2.6053330 -3.3642110 0.7714050

H 4.1963550 -2.3650510 -0.2823110

H 3.4856590 -1.3665580 0.9889370

H 0.7459600 4.8165190 -0.4024850

H 2.3256520 4.7837670 0.3712300

O -1.1120430 3.4882470 0.6152070

H 1.5159670 3.1647710 -2.0906870

H 2.8808790 4.2819080 -1.9693370

C -2.8950150 -1.3927560 -0.5274660

O -0.8671210 -3.4457540 -0.9631590

C	-2.9833490	1.2978960	0.1967410
H	1.1480330	-4.6583540	-0.2586500
H	2.6792610	-4.4306360	-1.0970140
H	3.3146450	-4.0420370	1.2411070
H	1.8529980	-3.0998730	1.5183370
C	-4.1125490	-0.7844560	-0.3028050
H	-2.8573950	-2.4351060	-0.8047820
C	-4.1575400	0.5865060	0.0701610
H	-3.0133890	2.3408700	0.4722020
C	-5.3802680	-1.5571860	-0.4496410
C	-5.4721550	1.2485180	0.3152520
H	-5.9535940	-1.5669740	0.4797630
H	-5.1881950	-2.5878010	-0.7377210
H	-6.0352850	-1.1124250	-1.2018380
H	-6.0269850	0.7544760	1.1157320
H	-5.3471720	2.2934790	0.5883230
H	-6.1142700	1.2045630	-0.5669770
N	1.1588230	-0.3626330	1.8484020
C	0.9722770	-0.6652180	2.9442540
C	0.7431920	-1.0576360	4.3068340
H	0.9435310	-2.1207990	4.4245460
H	1.3979560	-0.5009020	4.9736730
H	-0.2903310	-0.8620560	4.5846610
O	1.3558760	0.5240120	-2.2777640
H	0.7413310	-0.0440570	-2.7550370

57

M06L_MS_Ni_II_LD_MeCNOH_G3.log

57

Final Energy = -2787.1520067700

Ni	0.9627210	0.0287660	-0.4164260
N	2.0056270	-1.7761610	-0.8951560
N	2.1292160	1.7563080	0.1692610
C	3.2058910	-2.2587150	-0.1749860
H	2.2148540	-1.4099160	-1.8203260
N	-0.5246980	1.3759710	-0.1279570
N	-0.6504370	-1.1704660	-0.5994020
C	3.4935780	2.1957330	-0.1631830
H	2.0358040	1.7750340	1.1832020
C	1.4584750	-3.8392880	0.1242430
C	-0.4447560	-2.4854090	-0.8697030

C	2.9726080	-3.7429630	0.0643780
H	3.2930200	-1.7215570	0.7729740
H	4.1073460	-2.0491170	-0.7493600
C	-1.7721390	0.8549000	-0.0907760
C	-0.1724110	2.6922980	-0.0401340
C	-1.8445410	-0.5718420	-0.3563950
C	2.0155200	4.1300980	0.0288020
H	4.2192220	1.6756120	0.4590720
H	3.6788250	1.9145300	-1.2005290
H	1.0880110	-3.4802750	1.0881040
H	1.0673550	-4.8422620	-0.0265570
O	-1.2922520	-3.3645740	-1.0202080
H	3.4668230	-4.1073030	0.9630150
H	3.3469980	-4.3284870	-0.7773840
C	-2.9662180	1.5552610	0.1617970
O	-0.8923710	3.6581990	0.1928440
C	-3.1079450	-1.1892210	-0.3355400
H	1.7907230	4.9615750	-0.6359620
H	1.6993620	4.4303040	1.0293360
C	-4.1892040	0.9227640	0.1708830

H	-2.9038900	2.6155970	0.3559030
C	-4.2606290	-0.4778140	-0.0806230
H	-3.1618680	-2.2491380	-0.5312800
C	-5.4366080	1.6929730	0.4459650
C	-5.5836560	-1.1664670	-0.0685240
H	-6.1423240	1.6213520	-0.3841890
H	-5.2250030	2.7453280	0.6180860
H	-5.9617500	1.3058600	1.3216090
H	-6.2607210	-0.7458660	-0.8150200
H	-5.4811400	-2.2301220	-0.2684060
H	-6.0869710	-1.0493310	0.8934480
C	3.5013840	3.7257080	0.0081460
H	4.0444880	4.2066810	-0.8035820
H	3.9990740	4.0156210	0.9321690
C	1.2983040	2.8557420	-0.3752150
H	1.3569330	2.7109670	-1.4631990
C	1.0248250	-2.8824020	-0.9838730
H	1.1260550	-3.3992630	-1.9451200
N	1.0595780	-0.5204960	1.7243640
C	1.0072290	-0.9346450	2.7980080

C	0.9442390	-1.4666200	4.1305800
H	0.0062610	-1.1842380	4.6037350
H	1.0082650	-2.5526470	4.0985960
H	1.7689540	-1.0835950	4.7276690
O	1.3631010	0.4422070	-2.3488610
H	0.5226090	0.4459000	-2.8171680

49

M06L_MS_Ni_II_Me2_OPD_G1.log

49

Final Energy = -2578.5137955600

Ni	-1.1193930	0.1174290	-0.0460180
N	-2.2011190	1.8686080	-0.4867100
N	-2.4640460	-1.4380010	0.3167890
C	-1.2158370	3.0003710	-0.5979550
C	-3.2084200	2.2507590	0.5357550
H	-2.6545230	1.7262220	-1.3808490
N	0.2388050	-1.2738890	0.2389770
N	0.4428720	1.2787500	-0.2674490
C	-1.6702810	-2.6987070	0.5439610
C	-3.4216980	-1.7095690	-0.7897580
H	-3.0001080	-1.2561480	1.1561450
C	-1.5970660	3.9654480	0.5336440
C	0.2584370	2.5763570	-0.5092690
H	-1.3322410	3.4845320	-1.5690450
C	-2.4087530	3.1060090	1.4895550
H	-3.6485360	1.3617380	0.9858980
H	-4.0154360	2.8328850	0.0827260
C	1.5432800	-0.7942400	0.1252550
C	-0.1508510	-2.5225810	0.4958750
C	1.6548180	0.5983110	-0.1482690
C	-2.1787850	-3.6883180	-0.5085340
H	-1.9132380	-3.0732750	1.5409220
H	-4.3367960	-1.1414010	-0.6349850
H	-2.9768070	-1.3690680	-1.7280940
H	-0.7150210	4.4175430	0.9812820
H	-2.2182070	4.7748610	0.1487960
O	1.1051520	3.4692460	-0.6595420
H	-1.7529070	2.4760890	2.0956830
H	-3.0349080	3.6812360	2.1672810
C	2.7098640	-1.5476020	0.2561890

O 0.5395050 -3.5343890 0.6885320

C 2.9267670 1.1566340 -0.2757350

H -1.5637950 -3.6183850 -1.4090770

H -2.1204530 -4.7153520 -0.1578150

C 3.9737050 -0.9771500 0.1277910

H 2.6153900 -2.6042470 0.4633080

C 4.0835470 0.3931060 -0.1419360

H 3.0025100 2.2144220 -0.4838610

C 5.1991480 -1.8229190 0.2773690

C 5.4281430 1.0343660 -0.2848510

H 5.8202300 -1.8029770 -0.6214040

H 4.9436950 -2.8612410 0.4796620

H 5.8383500 -1.4738820 1.0920030

H 6.0107930 0.5844140 -1.0923230

H 5.3409460 2.0988190 -0.4937940

H 6.0313730 0.9223210 0.6193590

C -3.5910460 -3.2141330 -0.8028180

H -3.9862120 -3.5878030 -1.7454200

H -4.2737490 -3.5233210 -0.0086690

49

M06L_MS_Ni_II_Me2_OPD_G2.log

49

Final Energy = -2578.5019617100

Ni -1.1540710 -0.0002020 0.0000230

N -2.3681750 -1.6466380 0.4624220

N -2.3683270 1.6463220 -0.4613490

C -1.4770390 -2.8471980 0.6049870

C -3.3916380 -1.9700750 -0.5644620

H -2.8171840 -1.4536480 1.3492170

N 0.3136040 1.2534110 -0.2784810

N 0.3136670 -1.2538230 0.2781270

C -1.4771760 2.8466800 -0.6057420

C -3.3903390 1.9703380 0.5668760

H -2.8186590 1.4530740 -1.3474140

C -1.9068720 -3.7957300 -0.5246540

C 0.0239640 -2.5382280 0.5421820

H -1.6466650 -3.3125310 1.5774610

C -2.6461520 -2.8926540 -1.4990370

H -3.7607450 -1.0576360 -1.0320080

H -4.2437140 -2.4861260 -0.1131800

C	1.5761030	0.6965560	-0.1538990
C	0.0238530	2.5377560	-0.5427480
C	1.5761390	-0.6968850	0.1536410
C	-1.9067610	3.7967820	0.5225810
H	-1.6469100	3.3105640	-1.5788870
C	-2.6440910	2.8945730	1.4992090
H	-4.2434770	2.4852530	0.1163210
H	-3.7580970	1.0582230	1.0361110
H	-1.0527620	-4.3117500	-0.9572190
H	-2.5856400	-4.5570000	-0.1394810
O	0.8007590	-3.4800050	0.7261520
H	-1.9411740	-2.3178850	-2.1047270
H	-3.3051370	-3.4317480	-2.1753590
C	2.8000060	1.3541970	-0.2995630
O	0.8006240	3.4795330	-0.7268150
C	2.8000750	-1.3544250	0.2994870
H	-1.0526960	4.3144100	0.9533050
H	-2.5867560	4.5566490	0.1367850
H	-3.3024860	3.4341210	2.1757440
H	-1.9378900	2.3210580	2.1046620
C	4.0089610	0.6864780	-0.1521800
H	2.7934040	2.4088590	-0.5330100
C	4.0089960	-0.6865910	0.1523380
H	2.7935270	-2.4090860	0.5329390
C	5.3000090	1.4232000	-0.3155580
C	5.3000840	-1.4231780	0.3160060
H	5.9090430	1.3801690	0.5904520
H	5.1327540	2.4715920	-0.5534720
H	5.9140080	0.9944780	-1.1109800
H	5.9138400	-0.9944080	1.1115900
H	5.1328860	-2.4715930	0.5538560
H	5.9093350	-1.3800570	-0.5898520

49

M06L_MS_Ni_II_Me2_OPD_G3.log

49

Final Energy = -2578.5065973900

Ni	-1.0629660	0.1306480	-0.3679630
N	-2.0850860	1.9420950	-0.4821280
N	-2.3680710	-1.4551580	0.1226610
C	-3.0049950	2.2185880	0.6505090

H	-2.6263950	1.9969340	-1.3365100
N	0.2783430	-1.3100690	-0.0867550
N	0.5308990	1.2795860	-0.2647960
C	-3.7765990	-1.8060910	-0.1489470
H	-2.2716920	-1.4224410	1.1375860
C	-1.5306640	4.0543490	0.5702940
C	0.3748370	2.6060340	-0.3026240
C	-2.9980590	3.7254980	0.7916530
H	-2.5968730	1.7476370	1.5482510
H	-3.9772380	1.7717470	0.4539090
C	1.5844510	-0.8375050	-0.0086640
C	-0.1380410	-2.5805080	-0.0006920
C	1.7245420	0.5758530	-0.1148460
C	-2.4439860	-3.8172530	0.2576820
H	-4.4382350	-1.1789010	0.4442420
H	-3.9752490	-1.5971980	-1.2000010
H	-0.9642530	3.8879260	1.4887450
H	-1.3534940	5.0838950	0.2699870
O	1.2315110	3.4942930	-0.1965770
H	-3.3745210	4.0653990	1.7545700
H	-3.6186470	4.1761770	0.0143530
C	2.7329400	-1.6170790	0.1350390
O	0.5097080	-3.6079580	0.2351250
C	3.0060960	1.1266360	-0.0775580
H	-2.2799570	-4.7514950	-0.2739110
H	-2.1538410	-3.9867480	1.2961940
C	4.0053750	-1.0539600	0.1697100
H	2.6174220	-2.6888420	0.2176870
C	4.1438540	0.3368220	0.0607230
H	3.1049380	2.1996370	-0.1598270
C	5.2111960	-1.9270740	0.3191770
C	5.4991770	0.9702930	0.0909230
H	5.8965560	-1.8216470	-0.5253140
H	4.9358730	-2.9773200	0.3923120
H	5.7907340	-1.6726440	1.2098090
H	6.1398050	0.5999290	-0.7130820
H	5.4346980	2.0519620	-0.0097700
H	6.0300040	0.7549550	1.0214110
C	-3.8946770	-3.3115710	0.1564160
H	-4.4458650	-3.8214610	-0.6316490

H -4.4394230 -3.4856440 1.0824820
C -1.6300580 -2.6722350 -0.3132650
H -1.7183310 -2.6852940 -1.4070740
C -1.0745910 3.0566200 -0.5043240
H -1.1200980 3.5249150 -1.4899090
61
M06L_MS_Ni_II_Me2_OPD_MeCN2_G1.log
61
Final Energy = -2844.1403379300
Ni -0.9833550 0.1081260 0.0539000
N -2.0244280 1.8351030 -0.6596040
N -2.3050250 -1.3670010 0.8686760
C -1.0211240 2.7327210 -1.3276790
C -2.7873710 2.6498960 0.3217690
H -2.6601370 1.4792990 -1.3612110
N 0.4015950 -1.2748960 0.4804190
N 0.6188130 1.1453070 -0.5402870
C -1.5008570 -2.6168050 1.0704110
C -3.5460710 -1.7275070 0.1299470
H -2.5575320 -1.0005700 1.7770270
C -1.3175330 4.1276860 -0.7833010
C 0.4526700 2.3266150 -1.1201690
H -1.1806770 2.6917550 -2.4079220
C -1.8924620 3.8398340 0.5933710
H -3.0151780 2.0565140 1.2073850
H -3.7339840 2.9794250 -0.1143210
C 1.6985270 -0.8397710 0.2300720
C 0.0160670 -2.4683890 0.9125520
C 1.8170390 0.4752110 -0.3136700
C -2.1106160 -3.6079670 0.0840920
H -1.6748200 -2.9890660 2.0853620
H -4.4125280 -1.2701200 0.6049830
H -3.4794060 -1.3275250 -0.8835700
H -0.4214510 4.7439440 -0.7765450
H -2.0643200 4.6299810 -1.4007330
O 1.3108880 3.1183390 -1.5544350
H -1.0946630 3.5613370 1.2856820
H -2.4323690 4.6779640 1.0288720
C 2.8657610 -1.5678690 0.4745450
O 0.7021570 -3.4724680 1.1867980

C	3.0959340	0.9755340	-0.5718750
H	-1.6901190	-3.4443700	-0.9118160
H	-1.9017370	-4.6375270	0.3639500
C	4.1329090	-1.0536340	0.2108810
H	2.7650490	-2.5640010	0.8823750
C	4.2495850	0.2371060	-0.3195710
H	3.1766300	1.9716570	-0.9837730
C	5.3528820	-1.8737410	0.4930990
C	5.5964690	0.8201850	-0.6136280
H	5.9498280	-2.0418220	-0.4068500
H	5.0913450	-2.8480260	0.9018900
H	6.0190730	-1.3831030	1.2072170
H	6.1547510	0.2169800	-1.3340370
H	5.5139730	1.8265550	-1.0200410
H	6.2226170	0.8746280	0.2805140
C	-3.5846840	-3.2455570	0.1036770
H	-4.1425940	-3.6360750	-0.7456190
H	-4.0521720	-3.6320200	1.0116720
N	-0.8374190	0.9166950	2.0842000
C	-0.5228690	1.2804840	3.1307060
C	-0.1298050	1.7462720	4.4312850
H	0.8761560	1.4029390	4.6633590
H	-0.1443120	2.8340580	4.4564180
H	-0.8135090	1.3682650	5.1883170
N	-1.2599240	-0.7174150	-1.9657470
C	-1.1603620	-1.1904760	-3.0112570
C	-1.0382250	-1.7937810	-4.3089280
H	-0.0704580	-1.5541960	-4.7442700
H	-1.1271840	-2.8751300	-4.2247950
H	-1.8211440	-1.4270770	-4.9692150

61

M06L_MS_Ni_II_Me2_OPD_MeCN2_G2.log

61

Final Energy = -2844.1401075200

Ni	-1.0375330	0.0019520	0.0013250
N	-2.2287370	1.7037050	-0.5295540
N	-2.2317160	-1.6962450	0.5375890
C	-1.2998960	2.7739440	-1.0305820
C	-3.0975470	2.3038030	0.5153840
H	-2.8032690	1.3845830	-1.2984240

N	0.4650390	-1.2677750	0.3620200
N	0.4673510	1.2674130	-0.3643310
C	-1.3040180	-2.7689310	1.0355510
C	-3.1066140	-2.2945630	-0.5033280
H	-2.8021250	-1.3749640	1.3086290
C	-1.7289410	4.0431200	-0.2965960
C	0.2003360	2.4763480	-0.8406530
H	-1.4370780	2.8816240	-2.1090930
C	-2.3170990	3.5069320	0.9974270
H	-3.3093300	1.5732470	1.2958990
H	-4.0505230	2.6192800	0.0823480
C	1.7189490	-0.6883700	0.1928330
C	0.1963210	-2.4756600	0.8400530
C	1.7201990	0.6846190	-0.1991600
C	-1.7393820	-4.0372160	0.3037590
H	-1.4374550	-2.8758460	2.1146160
C	-2.3314350	-3.5000040	-0.9880460
H	-4.0585540	-2.6073100	-0.0660620
H	-3.3198510	-1.5638340	-1.2832820
H	-0.8890120	4.7207720	-0.1616130
H	-2.4973760	4.5726280	-0.8625380
O	0.9867180	3.3790570	-1.1861910
H	-1.5199180	3.1907280	1.6738990
H	-2.9392100	4.2243550	1.5283170
C	2.9469810	-1.3313180	0.3701610
O	0.9813430	-3.3804170	1.1833230
C	2.9493950	1.3242880	-0.3803440
H	-0.9017490	-4.7170690	0.1655980
H	-2.5068460	-4.5645490	0.8730330
H	-2.9577120	-4.2159750	-1.5159960
H	-1.5362040	-3.1863460	-1.6679840
C	4.1629670	-0.6792010	0.1825360
H	2.9359460	-2.3710800	0.6651930
C	4.1641950	0.6688700	-0.1966250
H	2.9401830	2.3641240	-0.6751950
C	5.4514500	-1.4135690	0.3853670
C	5.4540370	1.3997080	-0.4035500
H	6.0628770	-1.4239520	-0.5204670
H	5.2782040	-2.4474510	0.6782480
H	6.0691870	-0.9494880	1.1583580

H	6.0680860	0.9338780	-1.1784230
H	5.2827010	2.4340360	-0.6959750
H	6.0683200	1.4084900	0.5003680
N	-1.0958400	0.5062510	2.1355300
C	-0.8609850	0.6841780	3.2490060
C	-0.5645870	0.9135470	4.6355510
H	-0.4084680	1.9758170	4.8121210
H	-1.3885470	0.5709570	5.2576660
H	0.3371650	0.3746800	4.9187240
N	-1.1068410	-0.5023850	-2.1317950
C	-0.8853260	-0.6854960	-3.2471390
C	-0.6058700	-0.9217460	-4.6360240
H	-0.4527440	-1.9850040	-4.8093370
H	-1.4368550	-0.5812310	-5.2498650
H	0.2930190	-0.3853420	-4.9326280

61

M06L_MS_Ni_II_Me2_OPD_MeCN2_G3.log

61

Final Energy = -2844.1398097100

Ni	0.9855660	0.1073360	0.4332420
N	1.8301970	2.0466150	0.5119240
N	2.2505020	-1.0997700	-0.7641220
C	2.7031390	2.4387310	-0.6241910
H	2.3738770	2.1118490	1.3629460
N	-0.2978310	-1.3703280	-0.0349250
N	-0.7184030	1.1166910	0.6469610
C	3.7035720	-1.3182750	-0.8643940
H	1.9161850	-0.7824780	-1.6757510
C	0.9625810	4.0214690	-0.5685350
C	-0.6872670	2.4470070	0.5482800
C	2.4570540	3.9207500	-0.8176940
H	2.3898170	1.8799440	-1.5094900
H	3.7344340	2.1676710	-0.4069090
C	-1.6318190	-1.0214920	0.1268680
C	0.1840460	-2.4790790	-0.6061570
C	-1.8630370	0.3258310	0.5191920
C	2.4764850	-3.3099650	-1.5719900
H	4.1714370	-0.4899570	-1.3921950
H	4.1116220	-1.3375300	0.1474800
H	0.4102270	3.6869550	-1.4505110

H	0.6181440	5.0262370	-0.3381810
O	-1.6369030	3.2343510	0.3943660
H	2.7561060	4.2765510	-1.8019820
H	3.0107680	4.4967950	-0.0731140
C	-2.7312440	-1.8662090	-0.0440550
O	-0.4225170	-3.4398540	-1.1020490
C	-3.1739340	0.7450820	0.7444370
H	2.4804060	-4.3682560	-1.3207330
H	2.0055800	-3.2165990	-2.5525090
C	-4.0356790	-1.4312030	0.1705440
H	-2.5503050	-2.8864090	-0.3524410
C	-4.2607670	-0.1082400	0.5770860
H	-3.3427400	1.7694490	1.0451600
C	-5.1841040	-2.3709050	-0.0232970
C	-5.6516770	0.3834700	0.8278690
H	-5.7652060	-2.4982780	0.8932650
H	-4.8439140	-3.3553720	-0.3385530
H	-5.8868560	-2.0055710	-0.7760020
H	-6.1529350	-0.1921440	1.6098790
H	-5.6552180	1.4280710	1.1331500
H	-6.2820470	0.2954840	-0.0603670
C	3.8798560	-2.6818690	-1.5633960
H	4.5928460	-3.3011820	-1.0213590
H	4.2709340	-2.5651690	-2.5724660
C	1.7106140	-2.4701210	-0.5684480
H	2.0113830	-2.7618700	0.4466110
C	0.7161940	3.0508710	0.5909200
H	0.8080920	3.5866610	1.5394510
N	1.7275980	-0.4798240	2.2144460
C	2.1435290	-0.8532430	3.2203750
C	2.6579810	-1.3214500	4.4751190
H	2.1735240	-0.7970530	5.2961190
H	2.4701790	-2.3880420	4.5795610
H	3.7303360	-1.1459440	4.5289310
N	0.4016170	0.5772550	-2.6243090
C	-0.6783290	0.9860960	-2.5862650
C	-2.0191440	1.5053970	-2.5424840
H	-2.7325520	0.6890800	-2.4423730
H	-2.1319090	2.1672690	-1.6808030
H	-2.2367880	2.0580520	-3.4546590

60

M06L_MS_Ni_II_Me2_OPD_MeCNMeOH_G3.log

60

Final Energy = -2826.6106822400

Ni	1.0429210	0.0499400	0.5466470
N	1.9799550	1.9370660	0.6917400
N	2.3058900	-1.3966220	-0.4235400
C	3.0797570	2.3307100	-0.2162890
H	2.3189410	1.8183820	1.6395990
N	-0.3071460	-1.3830200	0.0982780
N	-0.6244770	1.1751270	0.5846560
C	3.7384130	-1.7217490	-0.3882350
H	2.0439540	-1.2156340	-1.3925510
C	1.3758180	3.9369800	-0.4748560
C	-0.4980790	2.4937560	0.4718950
C	2.8893530	3.8160000	-0.4608480
H	2.9765520	1.7753860	-1.1527200
H	4.0414830	2.0627020	0.2188880
C	-1.6220040	-0.9441190	0.1236480
C	0.1373940	-2.6098230	-0.1726800
C	-1.7974120	0.4457880	0.3944320
C	2.4205030	-3.7390800	-0.7792510
H	4.2974400	-1.0311010	-1.0172790
H	4.0858170	-1.5870150	0.6377300
H	0.9767920	3.5769200	-1.4274210
H	1.0092650	4.9506790	-0.3346480
O	-1.3736450	3.3479320	0.2206830
H	3.3625620	4.1610650	-1.3788860
H	3.3076540	4.3945880	0.3659490
C	-2.7588690	-1.7294280	-0.0888090
O	-0.4926840	-3.6400250	-0.4738920
C	-3.0950150	0.9556280	0.4550540
H	2.3478210	-4.7190700	-0.3116940
H	2.0036290	-3.8363820	-1.7836530
C	-4.0470660	-1.2022800	-0.0358570
H	-2.6180870	-2.7805260	-0.3009590
C	-4.2186620	0.1592440	0.2461120
H	-3.2212160	2.0094120	0.6609740
C	-5.2328120	-2.0852880	-0.2706460
C	-5.5898810	0.7549480	0.3227050

H	-5.9034550	-2.1070630	0.5921170
H	-4.9304310	-3.1100070	-0.4787100
H	-5.8388350	-1.7413850	-1.1126280
H	-6.2016690	0.2769440	1.0920380
H	-5.5484300	1.8188530	0.5492510
H	-6.1409100	0.6378610	-0.6139210
C	3.8586240	-3.1965310	-0.8224230
H	4.5178590	-3.7452720	-0.1509030
H	4.2870480	-3.2847610	-1.8197580
C	1.6568250	-2.6676550	-0.0203150
H	1.8653660	-2.7636610	1.0539840
C	0.9349800	3.0031630	0.6522890
H	0.9591090	3.5558120	1.5978680
N	0.9101580	0.4851560	-2.5590000
C	-0.1521980	0.9377270	-2.6042680
C	-1.4717150	1.5078190	-2.6735630
H	-2.2217020	0.7234690	-2.5843870
H	-1.6184080	2.2141820	-1.8529840
H	-1.6073540	2.0220440	-3.6233310
O	1.6742680	-0.2930060	2.4009090
C	0.7912230	-0.9513460	3.2166020
H	0.5810170	-1.9955060	2.9039830
H	1.1610680	-1.0227620	4.2561690
H	-0.2038920	-0.4664390	3.2870350

57

M06L_MS_Ni_II_Me2_OPD_MeCNOH_G3.log

57

Final Energy = -2787.3034346400

Ni	-1.0564490	0.0359230	-0.7384050
N	-2.0003820	1.9261520	-0.7901430
N	-2.3123470	-1.4375000	0.2121030
C	-3.1213290	2.2243240	0.1293350
H	-2.3266750	1.8864320	-1.7477510
N	0.3040250	-1.4081910	-0.3234350
N	0.6051850	1.1761190	-0.6664770
C	-3.7428030	-1.7646060	0.1512160
H	-2.0633060	-1.2645960	1.1859930
C	-1.4362150	3.8127730	0.5657150
C	0.4653520	2.4856460	-0.4797160
C	-2.9481210	3.6810360	0.5172380

H	-3.0324430	1.5817920	1.0101160
H	-4.0720550	1.9926390	-0.3486890
C	1.6146640	-0.9532110	-0.2889650
C	-0.1368450	-2.6284070	-0.0123290
C	1.7820140	0.4486830	-0.4904050
C	-2.4236590	-3.7807970	0.5446490
H	-4.3108860	-1.0848930	0.7842540
H	-4.0742510	-1.6109500	-0.8773900
H	-1.0483470	3.3657240	1.4856620
H	-1.0762320	4.8377490	0.5288870
O	1.3296510	3.3299630	-0.1658300
H	-3.4376130	3.9328110	1.4567600
H	-3.3600640	4.3325230	-0.2567900
C	2.7540320	-1.7366810	-0.0844310
O	0.4938530	-3.6355390	0.3553430
C	3.0755330	0.9725140	-0.5002130
H	-2.3349860	-4.7594830	0.0768930
H	-2.0273820	-3.8767410	1.5575150
C	4.0377180	-1.1966050	-0.0850930
H	2.6194000	-2.7971310	0.0784440
C	4.2020560	0.1775760	-0.3029170
H	3.1958380	2.0357710	-0.6539180
C	5.2267910	-2.0784140	0.1366810
C	5.5689760	0.7873500	-0.3231560
H	5.9160640	-2.0557900	-0.7112270
H	4.9303920	-3.1140400	0.2927940
H	5.8109150	-1.7663500	1.0060790
H	6.1996450	0.3520250	-1.1025320
H	5.5227420	1.8606610	-0.4984810
H	6.1024990	0.6283770	0.6173880
C	-3.8658520	-3.2474390	0.5578510
H	-4.5017240	-3.7887870	-0.1417250
H	-4.3217420	-3.3559650	1.5408650
C	-1.6529570	-2.7011100	-0.1938780
H	-1.8464440	-2.7908130	-1.2718060
C	-0.9700280	2.9945690	-0.6391870
H	-0.9872800	3.6338770	-1.5284180
N	-0.9335750	0.2812340	2.4649240
C	0.1171170	0.7578800	2.5289480
C	1.4209210	1.3586400	2.6274800

H	2.1915810	0.6027070	2.4857720
H	1.5437330	2.1191680	1.8527670
H	1.5478310	1.8157900	3.6072040
O	-1.7335130	-0.2136600	-2.5925740
H	-1.3430910	-1.0114770	-2.9605670

[LNi^{III}Cl$_2$]

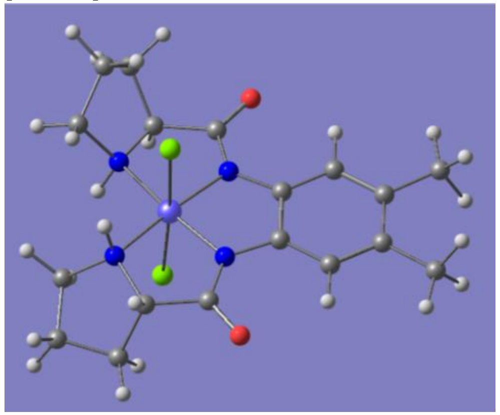

28	0.871380000	0.066373000	-0.147910000
7	2.041767000	1.617845000	0.197114000
7	2.203418000	-1.307086000	-0.618003000
6	1.192686000	2.785887000	0.647594000
6	2.973682000	2.110216000	-0.872374000
1	2.571496000	1.275470000	1.005057000
7	-0.410029000	-1.280118000	-0.307386000
7	-0.561671000	1.221900000	0.133430000
6	1.522607000	-2.653196000	-0.660941000
6	3.466705000	-1.443827000	0.181068000
1	2.408439000	-1.018458000	-1.582130000
6	1.701194000	3.998363000	-0.146029000
6	-0.300513000	2.504700000	0.451001000
1	1.336289000	2.919155000	1.729340000
6	2.304305000	3.370651000	-1.408269000
1	3.109116000	1.335678000	-1.634407000
1	3.942457000	2.340896000	-0.405154000
6	-1.724937000	-0.808932000	-0.182540000
6	0.009149000	-2.540860000	-0.527045000
6	-1.810861000	0.587245000	0.048210000
6	2.207482000	-3.464400000	0.447104000
1	1.736807000	-3.115414000	-1.637455000
1	4.288363000	-0.957443000	-0.357271000
1	3.313719000	-0.943274000	1.145528000
1	0.882716000	4.700365000	-0.348500000
1	2.475688000	4.527081000	0.427474000

8	-1.142271000	3.408351000	0.634984000
1	1.513544000	3.086364000	-2.117460000
1	3.017003000	4.029433000	-1.919863000
6	-2.899931000	-1.568105000	-0.266097000
8	-0.709312000	-3.559105000	-0.627292000
6	-3.070651000	1.187455000	0.178539000
1	1.754331000	-3.220365000	1.419248000
1	2.115694000	-4.542552000	0.273271000
6	-4.158262000	-0.965250000	-0.132503000
1	-2.821302000	-2.639526000	-0.435743000
6	-4.244539000	0.426165000	0.089040000
1	-3.126428000	2.260012000	0.349762000
6	-5.403879000	-1.808181000	-0.226891000
6	-5.585546000	1.099765000	0.228854000
1	-6.008935000	-1.738634000	0.691358000
1	-5.151177000	-2.863742000	-0.390613000
1	-6.054087000	-1.479984000	-1.053970000
1	-6.159319000	0.685340000	1.073395000
1	-5.468159000	2.178852000	0.392394000
1	-6.205286000	0.954544000	-0.670583000
6	3.651287000	-2.950763000	0.387166000
1	4.222845000	-3.169937000	1.298127000
1	4.178209000	-3.397495000	-0.469317000
17	0.784056000	0.400257000	-2.714411000
17	1.109798000	-0.341685000	2.360329000

[LNi^{III}Cl$_2$] (3Cl)

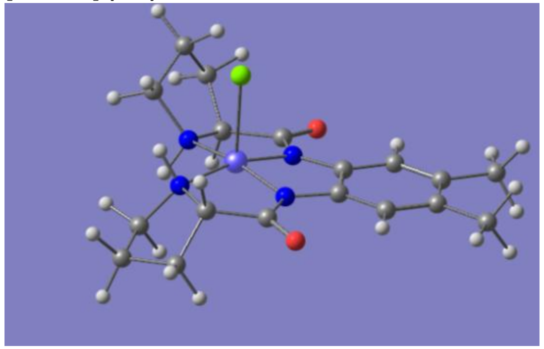

28	0.867157000	0.115697000	-0.372731000
7	2.058476000	1.621066000	0.081306000
7	2.216684000	-1.283341000	-0.550679000
6	1.236532000	2.788952000	0.581564000
6	2.975163000	2.141760000	-0.993658000
1	2.622053000	1.268025000	0.859559000
7	-0.384707000	-1.246043000	-0.322575000

7	-0.529699000	1.206264000	0.130704000
6	1.542018000	-2.619287000	-0.763523000
6	3.175511000	-1.445183000	0.588909000
1	2.738045000	-1.055254000	-1.403185000
6	1.675964000	4.004009000	-0.254242000
6	-0.259762000	2.494275000	0.493447000
1	1.454654000	2.937957000	1.648963000
6	2.257857000	3.375072000	-1.525657000
1	3.135438000	1.367698000	-1.751297000
1	3.935339000	2.409166000	-0.528436000
6	-1.700785000	-0.800726000	-0.188357000
6	0.035891000	-2.521860000	-0.558263000
6	-1.784856000	0.595543000	0.065100000
6	2.225156000	-3.602013000	0.214711000
1	1.714202000	-2.920038000	-1.806289000
1	4.011340000	-0.747986000	0.460109000
1	2.646373000	-1.204931000	1.522227000
1	0.830211000	4.677054000	-0.440513000
1	2.450946000	4.566415000	0.284383000
8	-1.098311000	3.356125000	0.791477000
1	1.456726000	3.065098000	-2.212090000
1	2.939979000	4.046385000	-2.061546000
6	-2.874942000	-1.562597000	-0.276269000
8	-0.681376000	-3.532424000	-0.598215000
6	-3.041245000	1.197875000	0.224019000
1	1.612899000	-3.706064000	1.122096000
1	2.343842000	-4.595233000	-0.232178000
6	-4.125004000	-0.959419000	-0.112782000
1	-2.803129000	-2.630076000	-0.468417000
6	-4.209194000	0.434753000	0.138549000
1	-3.098970000	2.266531000	0.413830000
6	-5.373803000	-1.793830000	-0.206556000
6	-5.549367000	1.097857000	0.309724000
1	-5.965928000	-1.733743000	0.720329000
1	-5.128978000	-2.847145000	-0.391902000
1	-6.030582000	-1.444415000	-1.019230000
1	-6.106741000	0.661005000	1.153599000
1	-5.435707000	2.173794000	0.492250000
1	-6.179541000	0.963055000	-0.583649000
6	3.560034000	-2.922020000	0.548832000

1	3.985549000	-3.266114000	1.499794000
1	4.298308000	-3.098370000	-0.247647000
17	0.832299000	0.336062000	-2.798169000
